# Leveraging Immunogenic Cell Death to Enhance the Immune Response against Malignant Pleural Mesothelioma Tumors

**DOI:** 10.1021/jacs.4c17966

**Published:** 2025-02-24

**Authors:** Meng Rui Chang, Egor M. Matnurov, Chengnan Wu, Jemma Arakelyan, Ho-Jung Choe, Vladimir Kushnarev, Jian Yu Yap, Xiu Xuan Soo, Mun Juinn Chow, Walter Berger, Wee Han Ang, Maria V. Babak

**Affiliations:** a Department of Chemistry, National University of Singapore, 4 Science Drive 2, Singapore 117543, Singapore; b Drug Discovery Lab, Department of Chemistry, City University of Hong Kong, 83 Tat Chee Avenue, Hong Kong SAR 999077, People’s Republic of China; c NUS Graduate School - Integrated Science and Engineering Programme (ISEP), National University of Singapore, Singapore 119077, Singapore; d Center for Cancer Research and Comprehensive Cancer Center, Medical University of Vienna, Borschkegasse 8A, Vienna 1090, Austria

## Abstract

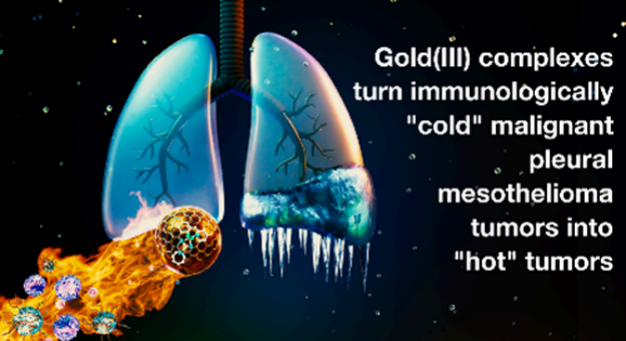

Although various metal-based compounds have exhibited excellent immunogenic cell death (ICD)-inducing properties both *in vitro* and *in vivo,* the majority of these compounds have been discovered serendipitously. In this work, we have successfully synthesized and characterized 35 cyclometalated Au(III) complexes containing dithiocarbamate ligands, with 25 of these complexes being previously unreported. Their ability to induce phagocytosis *in vitro* against immunologically “cold” malignant pleural mesothelioma (MPM) cells was strongly dependent on the cyclometalated scaffold and the overall lipophilicity of the complexes. We elucidated the role of cell death mechanisms in the observed ICD effects and identified correlations between the ability of the complexes to induce necrotic cell death and ICD, both *in vitro* and *in vivo*. Complex **2G**, with its high phagocytosis rates and low necrosis rates, was recognized as a *bona fide* ICD inducer, demonstrating a remarkably long-lasting immune response in vaccinated mice. In contrast, complex **1C**, characterized by high phagocytosis rates and high necrosis rates, failed to elicit a sustained immune response upon following vaccination; however, it triggered selective activation of calreticulin in tumors upon direct *in vivo* administration. Overall, this study offers a framework for predicting ICD effects *in vivo* for structurally similar Au(III) complexes, with the potential for extension to other series of metal complexes.

## Introduction

Malignant pleural mesothelioma (MPM) is a very rare disease typically associated with exposure to asbestos, accounting for approximately 0.2% of the total new cancer cases (30,870 cases) and 0.3% of the total cancer-related deaths (26,278 deaths) worldwide in 2020.^1^ Patients are often diagnosed with MPM at advanced stages, leading to a median overall survival of approximately 1-year postdiagnosis. The 5-year overall survival rate generally remains below 10%, highlighting the aggressive nature of the disease and the challenges in achieving long-term survival outcomes.^2^ The standard-of-care treatment approach for patients with MPM usually consists of a trimodal therapy involving surgery, radiation therapy, and chemotherapy.^3^ Surgery is recommended for patients at stages 1–3 who are deemed suitable for an operative intervention.^4^ Radiation therapy can be employed either as part of a multimodal treatment regimen or independently for palliative purposes.^5^ Chemotherapy plays a crucial role in the integrated treatment of MPM, with the cisplatin-pemetrexed regimen emerging as the preferred first-line option, adaptable for both neo-adjuvant and adjuvant settings.^6^

In 2020, the United States Food and Drug Administration approved the immune checkpoint blockade (ICB) combination treatment (nivolumab [anti-PD1] + ipilimumab [anti-CTLA4]) for patients with unresectable MPM. This approval was based on the findings of a Phase III CheckMate 743 clinical trial that involved 605 patients with advanced, previously untreated MPM.^7^ The results showed that patients in the nivolumab-ipilimumab group had a median overall survival of 18 months relative to 14 months for patients receiving platinum-pemetrexed treatment.^7^ Due to its generally low response rates to ICB, MPM could be classified as an immunologically “cold” cancer. More precisely, most MPM tumor microenvironments exhibit an intermediate inflammatory state, rendering this cancer type suitable for therapeutic strategies aimed at boosting its immunogenicity.^8^

One effective strategy to activate the tumor immune microenvironment and boost the efficacy of ICB treatment involves triggering ICD in tumor cells. ICD is a process wherein dying cells release molecules and signals, called danger-associated molecular patterns (DAMPs), that attract immune cells to the site of cell death, facilitating the activation of an antitumor immune response.^9^ The exposure and subsequent release of DAMPs are believed to occur as a consequence of the induction of endoplasmic reticulum (ER) stress. Type I ICD inducers are known to trigger indirect ER stress, whereas Type II ICD inducers preferentially target the ER, leading to a more effective ICD induction. While the standard-of-care MPM treatment agents, cisplatin and pemetrexed, can initiate the ER stress-dependent death of MPM cells under specific conditions,^10,11^ this process is insufficient to trigger ICD.^11,12^ In contrast, pemetrexed has been found to effectively trigger ICD in other cancer cell types,^13^ further highlighting the comparatively low immunogenicity of MPM cells.

**Figure 1:**
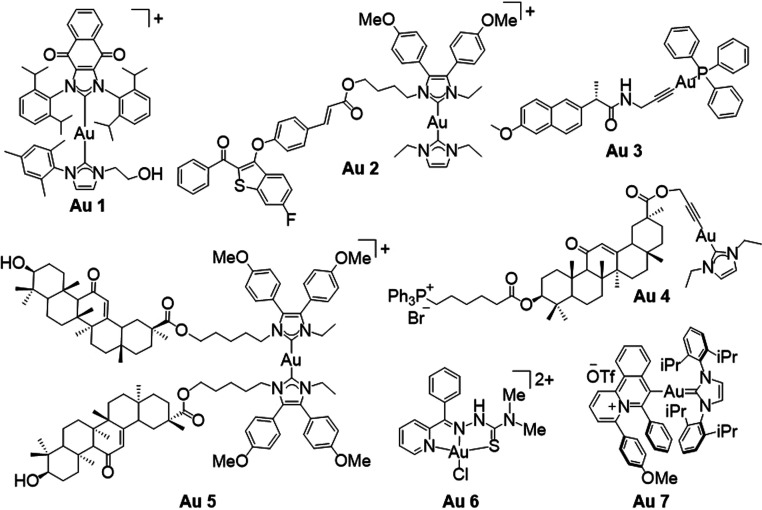
Selected Au complexes with immunogenic properties.

In recent years, gold (Au) complexes have emerged as promising ICD inducers due to their ability to initiate targeted ER stress-mediated cell death (Figure 1).^14−16^ Arambula and Sessler et al. reported a rationally designed redox-active Au(I)-N-heterocyclic carbene (NHC) complex, **Au1**, which demonstrated the ability to induce ICD in lung cancer cells *in vitro* and in colon cancer cells *in vivo.*(15) Upon vaccination of the immunocompetent mice with **Au1**, mice remained tumor-free for more than 42 days. The authors suggested that the observed ICD effects were related to the dual targeting of the cancer antioxidant network through thioredoxin reductase (TrxR) inhibition by the Au(I) fragment and redox cycling via the naphthoquinone ligand moiety.^15^ Several Au(I) complexes targeting the Trx–ROS–ER–ICD axis have been reported by Liu et al., including an Au(I)-NHC complex that incorporates a steroidal selective estrogen receptor degrader (SERD) moiety, **Au2**; an Au(I)-alkynyl complex with naproxen, **Au3**; and Au(I)-NHC complexes with a liver-targeting 18β-glycyrrhetinic acid moiety, **Au4** and **Au5.**(17−20) Mice that were vaccinated with **Au4** and **Au5** remained free from hepatocellular carcinoma tumors for more than 30 days.^19,20^ Besides Au(I) complexes, an Au(III) 2-benzoylpyridine thiosemicarbazone complex **Au6** developed by Liang and Yang et al. was shown to induce ICD in ovarian cancer cells *in vitro*.^21^ Additionally, Senthilkumar, Kumar and Patil et al. identified the complex **Au7** as a potent ICD inducer in lung cancer cells *in vitro.*(22)

While several Au-based ICD inducers have been documented, no complex has yet been investigated for its efficacy against immunologically “cold” tumors. Furthermore, it remains uncertain whether it is feasible to pinpoint distinct characteristics of Au complexes that could facilitate the identification of the most potent ICD inducers from libraries of Au(I) and Au(III) complexes.

In this work, we prepared a library of 35 cyclometalated Au(III) complexes and evaluated their ICD properties against immunologically “cold” MPM cells. The Au(III) complex **2G** emerged as the most effective ICD inducer against MPM, leading to tumor-free conditions in immunocompetent mice for a period exceeding 6 months postvaccination. Importantly, we established direct correlations between the abilities of Au(III) complexes to elicit necrosis and promote phagocytosis in an *in vitro* setting and demonstrated the consistency of these correlations in an *in vivo* context. These findings hold the potential to enhance our understanding of the drug properties that can be used for the identification of novel effective ICD inducers for immunologically “cold” cancers.

## Results

### Synthesis, Characterization, and Stability

We designed a combinatorial library of 40 cyclometalated Au(III) complexes with five cyclometalated scaffolds and eight dithiocarbamate (DTC) ligands. The library was acquired via the reactions of eight DTC salts (**A**–**H**) with cyclometalated Au(III)-dichlorido scaffolds **1**–**5** (Scheme 1). Among the 40 proposed complexes, 35 compounds were successfully obtained with yields up to 79% and high purity (>95%), as validated using reversed-phase high-performance liquid chromatography (RP-HPLC) analysis (Supplementary Figures 1–35). Notably, 25 of these complexes are novel and have not been previously documented. The synthesis of complexes **3C**–**3F** and **3H** with benzoyl scaffold **3** was unsuccessful due to the electron-withdrawing carbonyl group causing scaffold decoordination from the Au(III) center, leading to the formation of thermodynamically favored structures.^23^ The structures of all 35 complexes were verified using ^1^H and ^13^C NMR, as well as ESI-MS analysis (Supplementary Figures 36–110). The ^1^H NMR spectra of all Au(III)–DTC complexes were characterized by eight aromatic signals of cyclometalated ligands between approximately 6.5 and 8.5 ppm. These signals partially overlapped with the aromatic signals of DTC ligands **D** and **E**. All other complexes were also characterized by the aliphatic peaks of DTC ligands, which were shifted downfield in comparison with DTC salts, indicating a successful coordination of the DTC motif to the Au(III) metal center. The complexes with the asymmetric DTC ligand **E** were characterized by the presence of geometric isomers in their NMR spectra. The ESI-MS of all complexes corroborated the presence of the parent molecular [M]^+^ at their predicted *m*/*z* (Supplementary Figures 86–110).

**Scheme 1:**
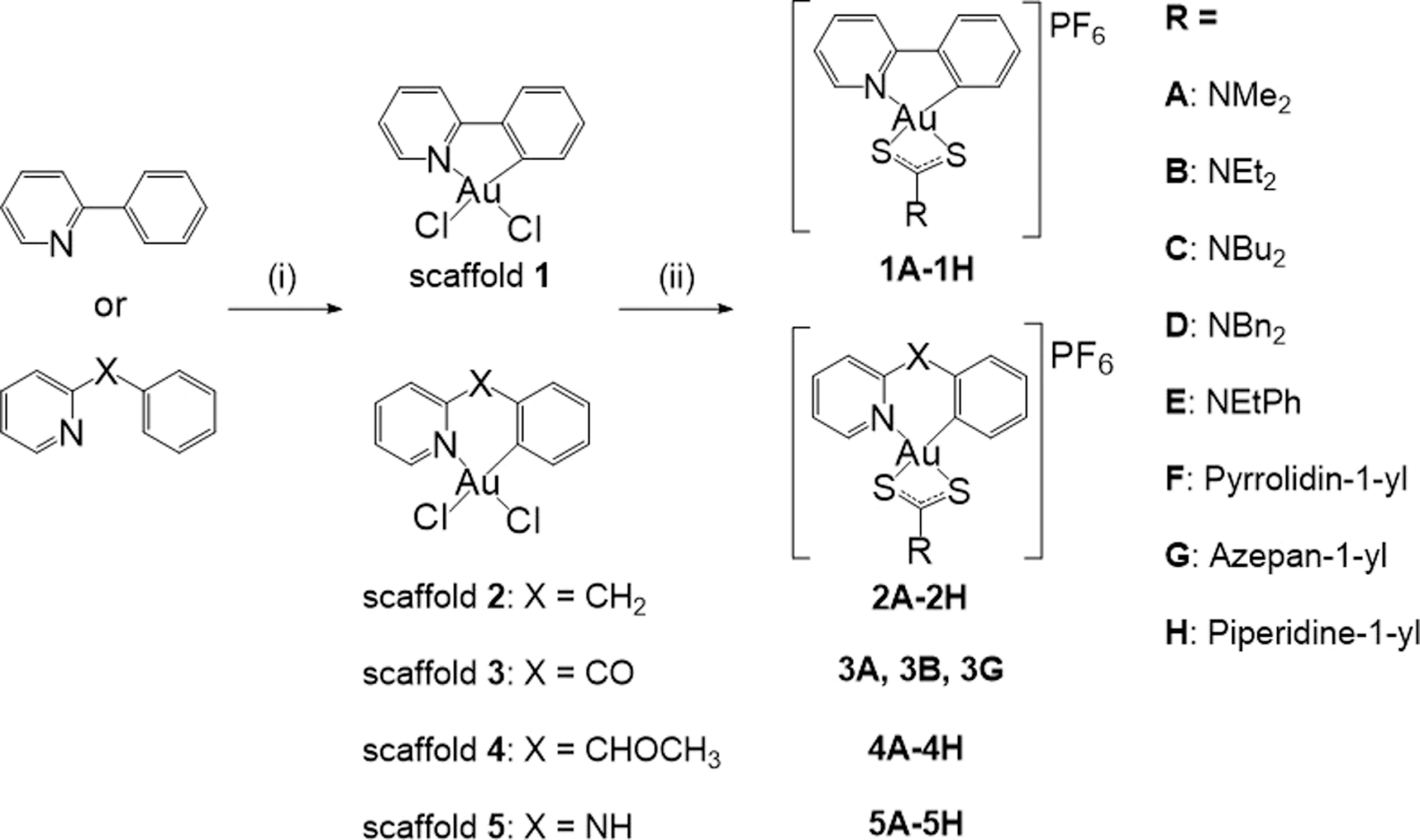
Synthesis Route of Cyclometalated Au(III)–DTC Complexes Reagents and conditions: (i) Scaffolds **1** and **3**: KAuCl_4_, 2-phenylpyridine or 2-benzoylpyridine, H_2_O/EtOH 5:1, 1 h, RT, followed by AgOTf, ACN, overnight reflux. Scaffold **2**: KAuCl_4_, 2-benzylpyridine, H_2_O, 72 h, reflux. Scaffold **4**: KAuCl_4_, 2-(methoxyphenylmethyl)pyridine, H_2_O/ACN 4:1, 1 h, RT, followed by H_2_O/ACN 5:1, 24 h, reflux. Scaffold **5**: KAuCl_4_, 2-anilinopyridine, H_2_O/ACN 1:1, 24 h, RT; (ii) DTC salt, MeOH, 12 h, RT, followed by the addition of NH_4_PF_6_, 1 h, RT.

The molecular structure of **1H** was confirmed by X-ray diffraction analysis (Supplementary Figure 111), and the crystallographic details are summarized in Supplementary Table 1. The crystals suitable for analysis were grown by the slow diffusion of diethyl ether into a chloroform solution. This complex demonstrated a distorted square planar geometry around Au(III) due to the nonequivalent coordination environment. The Au–N(1) and Au–C(1) bond lengths were 2.060(5) and 2.040(6) Å, respectively, while the Au–S(1) and Au–S(2) bond lengths were 2.399(16) and 2.273(16) Å, respectively. The C(2)–S(1/2) bond lengths were 1.731(7)–1.733(7) Å, which lies between the expected C–S and C=S bond lengths of 1.82 and 1.60 Å, respectively. At the same time, the C(2)–N(2) bond length decreased to 1.312(8) Å, relative to the typical C–N and C=N bond lengths of 1.47 and 1.29 Å, respectively. These data indicate the occurrence of thioureide resonance within the DTCs scaffold in Au(III)–DTC complexes, characterized by electronic delocalization across the DTC–NCS_2_ motif.

The aqueous stability of the Au(III)–DTC complexes was assessed by monitoring their RP-HPLC profiles in a 1:1 DMSO:H_2_O mixture at room temperature over a 3-day period (Supplementary Figures 112–146). All but **2E**, **2F**, **4C**, and **4E** complexes displayed consistent RP-HPLC chromatograms over the entire 72-h period. After 24 h, complexes **2E** and **2F** exhibited a minor peak at 3.8 min, accounting for 4% and 5% of the total area, respectively (Supplementary Figures 124 and 125). This proportion increased to 9% for **2F** after 72 h, while it remained unchanged for **2E**. Similarly, **4C** and **4E** exhibited a minor peak at 4.4 min after 24 h, representing 2% and 5% of the total area, respectively. Subsequently, these peaks increased to 8% and 7% after 72 h for **4C** and **4E**, respectively (Supplementary Figures 133 and 135). These findings suggest that, except for **2E**, **2F**, **4C**, and **4E**, which underwent less than 10% hydrolysis over the 72-h period, all other complexes maintained stability in a DMSO-containing aqueous environment.

### *In Vitro* Cytotoxicity and *In Vivo* Efficacy

The cytotoxicity of 35 Au(III)–DTC complexes in comparison with standard-of-care drugs for MPM treatment, i.e., cisplatin and pemetrexed,^24^ was evaluated using a colorimetric MTT assay over a 72-h period against a panel of seven MPM cell lines comprising one murine malignant mesothelioma cell line, AB12; five human malignant mesothelioma cell lines, JU77, LO68, VMC23, Meso84, and Meso92; and one human nonmalignant mesothelioma cell line, Met5A (Supplementary Table 2, Figure 147–153). The VMC23, Meso84, and Meso92 cell lines were derived from surgical specimens obtained from patients with MPM by us and authenticated using short tandem repeat analysis.^25^ These cell lines correspond to three distinct MPM histological subtypes: VMC23 representing the epithelioid type, Meso84 the sarcomatoid type, and Meso92 the mixed biphasic type.

In general, all complexes demonstrated excellent cytotoxicity within the range of nanomolar to low micromolar concentrations of 0.053–8.4 μM. In comparison, cisplatin exhibited cytotoxicity within the 2–20 μM range, while pemetrexed fell within the 0.023–0.3 μM range. Among all of the tested complexes, the most cytotoxic complexes were identified as **5A** (Meso92), **5B** (AB12, LO68, VMC23, and Meso84), and **5C** (JU77). In addition, these complexes demonstrated better selectivity for MPM cells than Met5A. Analysis of the IC_50_ values across all MPM cell lines indicated that the influence of DTC ligands on the cytotoxicity of Au(III) complexes was modest and dependent on the individual cell line. For example, among scaffold **1** and **5** complexes, ligands **A**–**C** with relatively small *N*-alkyl substituents exhibited the highest cytotoxicity, while ligands **D** and **E** with bulkier -NBn_2_ and -NEtPh substituents showed the lowest cytotoxicity. For scaffold **2**, **2A** (-NMe_2_) and **2F** (pyrrolidin-1-yl) were the most cytotoxic, while **2B**–**2D** were the least cytotoxic. For scaffold **4**, **4A** (-NMe_2_) and **4H** (piperidine-1-yl) were the most cytotoxic, while **4B**, **4D**, and **4E** were the least cytotoxic. In contrast, the variation in cyclometalated scaffolds revealed a consistent trend for the Au(III) complexes, irrespective of the cell subtype: **5** < **1** < **3** < **2** ≈ **4**. This suggests that, on average, complexes featuring the 2-anilinopyridine scaffold **5** were the most cytotoxic, whereas those with the 2-(methoxyphenylmethyl)pyridine scaffold **4** and the 2-benzylpyridine scaffold **2** exhibited the least cytotoxicity. The lowest activity of complexes with scaffolds **2** and **4** was attributed to their reduced stability in aqueous environments. Additionally, the cytotoxicity of selected complexes **1G**, **5A**, **5B** and **5G** toward M0-differentiated THP-1 cells was assessed in comparison with AB12 cells (Supplementary Figure 154). The results revealed that THP-1-derived macrophages were highly sensitive to the tested Au complexes. Specifically, in the case of **5G**, it was observed that these macrophages, despite the lack of proliferation subsequent to differentiation, exhibited increased sensitivity in comparison to the malignant mesothelioma cells, suggesting potential macrophage toxicity of the tested complexes.

**Figure 2:**
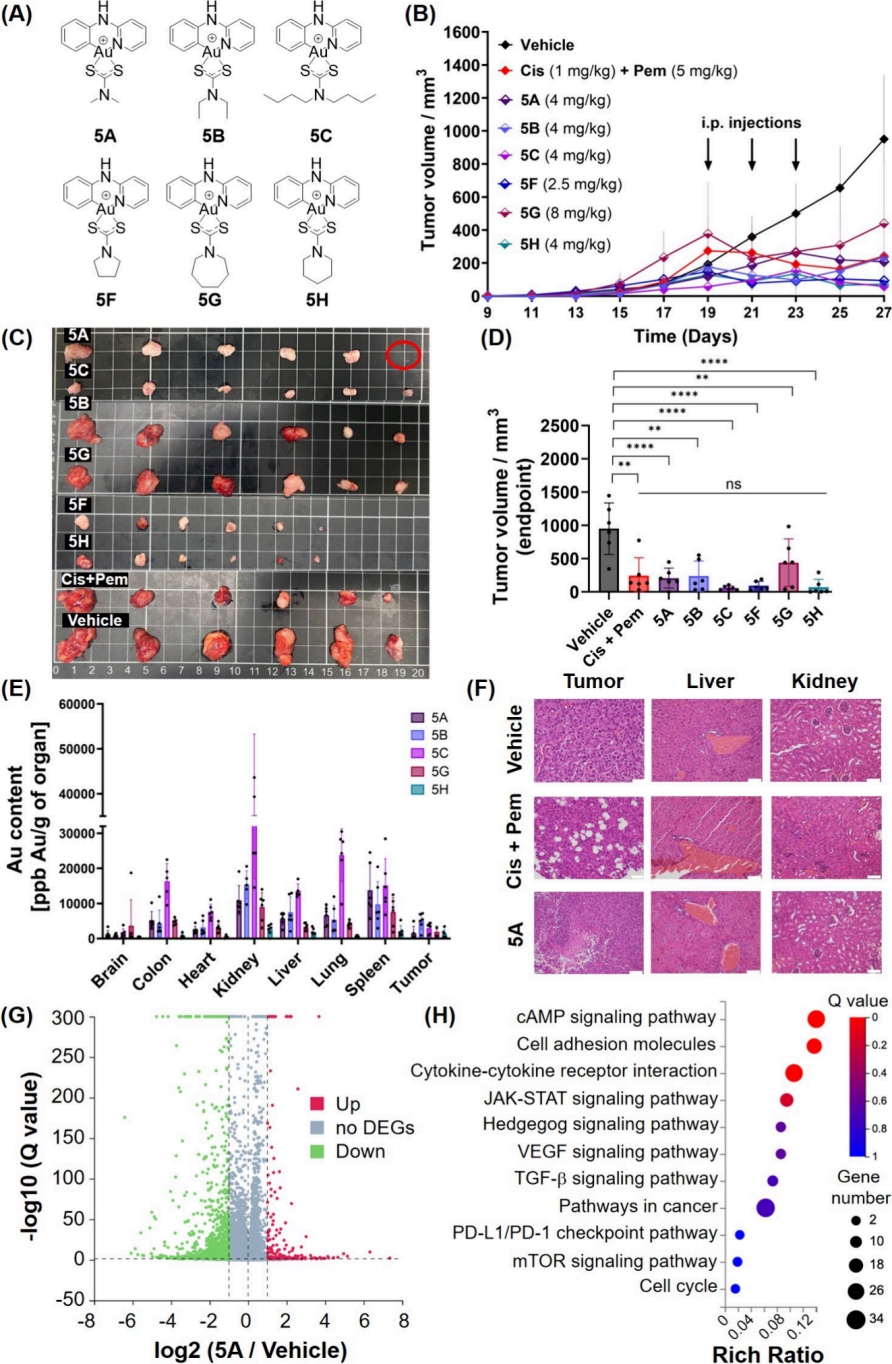
*In vivo* efficacy of Au(III)–DTC complexes with scaffold 5 against MPM tumors. (**A**) Chemical structures of the most cytotoxic Au(III)–DTC complexes. Counterions were omitted for clarity. (**B**) Time-dependent growth of AB12 tumors reflected by changes in the tumor volume. BALB/c mice were subcutaneously inoculated with AB12 cells and treated with complexes **5A**–**5C**, **5F**–**5H**, or cisplatin + pemetrexed at the indicated doses or with the corresponding vehicle on Days 19, 21, and 23 via the intraperitoneal route. Tumor growth was monitored using calipers. (**C**) Corresponding tumor images. (**D**) Tumor volume at the experimental end point (Day 27). (**E**) Au accumulation in mouse organs and tumors obtained from Au-treated mice at the end point (Day 27) and quantified using ICP-MS. (**F**) Representative H&E-stained tumor, liver, and kidney tissues from mice treated with cisplatin + pemetrexed, **5A,** or the corresponding vehicle. (**G**) Volcano plot of DEGs between vehicle- and **5A**-treated mouse AB12 tumors. (**H**) KEGG pathway enrichment bubble plot for all downregulated DEGs in **5A**-treated mouse AB12 tumors relative to vehicle-treated mouse AB12 tumors. Mean ± SD values were obtained from individual tumors (*n* = 6). (**G** and **H**) DEGs were defined as genes with a log base 2-fold change value less than −1 or greater than 1 and an FDR-adjusted p value (Q value) less than 0.05. Rich factor = DEGs enriched in the pathway/all genes in the background gene set. The size and color of the bubble represent the number of DEGs enriched in the pathway and the enrichment significance (Q value), respectively. Statistical analysis was performed using one-way ANOVA with Dunnett’s multiple comparisons.

For the *in vivo* studies, we selected complexes featuring scaffold **5** due to their superior activity to complexes featuring other scaffolds, as observed in patient-derived cell lines and the murine AB12 cell line. To explore the potential impact of the DTC ligand structure on the *in vivo* efficacy of the respective Au(III)–DTC complexes, we chose six complexes, specifically **5A**–**5C** and **5F**–**5H** (Figure 2A). The toxicity study was conducted using the maximum soluble doses in 4% DMSO/4% Cremophore EL in saline, specifically **5G** at 8 mg/kg; **5A,5B, 5C** and **5H** at 4 mg/kg; and **5F** at 2.5 mg/kg. Compounds **5A**, **5H**, and **5F** were administered to mice daily (total of 12 injections), while the remaining compounds were administered every second day (total of six injections), with continuous monitoring of their body weights (Supplementary Figure 155). Mice receiving injections every second day showed no signs of toxicity; however, daily injections led to toxicity in mice, including a reduction in general activity and the death of two mice in group **5H** (Days 7 and 11) and of one mouse in group **5F** (Day 11). Consequently, for the tumor allograft study, AB12 tumor-bearing mice were injected with complexes **5G** at 8 mg/kg; **5A, 5B, 5C** and **5H** at 4 mg/kg; **5F** at 2.5 mg/kg; or the corresponding vehicle every second day on Days 19, 21, and 23 and then sacrificed on Day 27 (Figure 2B). Body weight changes are shown in Supplementary Figure 156. Additionally, we compared the efficacy of Au(III) complexes with that of the standard-of-care MPM regimen, consisting of cisplatin (1 mg/kg) and pemetrexed (5 mg/kg), with doses selected to ensure efficacy while minimizing potential toxicity, based on prior published data.^26^

Importantly, all complexes and the cisplatin-pemetrexed combination, independent of the structure of the DTC ligand, led to a notable reduction in tumor burden compared with the vehicle-treated group (Figures 2B-2D). In particular, one of the **5A**-treated mice showed complete remission, reflected by the total disappearance of the tumor. All complexes, except for **5C**, exhibited similar organ biodistribution profiles, with the highest levels of Au accumulation observed in the kidney and spleen, followed by the colon, liver, lung, and tumor (Figure 2E). A similar distribution pattern is commonly observed for Au nanoparticles administered via the *i.p.* route.^27^**5C** exhibited the highest accumulation inside mouse organs, in particular showing unusually high levels of Au in the kidneys and lungs. A subsequent histological examination of the livers and kidneys revealed a preserved kidney and liver cytoarchitecture, indicating no signs of Au-induced nephrotoxicity or hepatotoxicity (Figure 2F, Supplementary Figure 157). Additionally, the tissues from **5A**-treated tumors demonstrated the presence of few scattered lymphocytes (Figure 2F).

To determine the mechanisms underlying the *in vivo* efficacy of **5A**, we conducted a transcriptomic analysis of **5A**- and vehicle-treated tumors. In total, 17,675 genes were identified, and those genes that had Q values less than 0.05 and log base 2 fold change absolute values greater than 1 were considered as differentially expressed genes (DEGs). A total of 178 DEGs were upregulated and 1,174 DEGs were downregulated in the treatment group compared with the control group (Figure 2G). The full list of upregulated and downregulated genes is provided in Supplementary Tables 3 and 4. Specifically, the downregulation of *MUC16* and *WT1* is of significance for MPM, given the high levels of MUC16 and WT1 on the cell surface of MPM cells.^28–30^*MUC16* has been reported to be involved in the regulation of tumor implantation and dissemination through cell adhesion mechanisms.^28,29^ Similarly, *WT1* has been implicated in promoting oncogenesis in MPM and regulating the epithelial-to-mesenchymal transition.^30^ Functional enrichment analysis via the Kyoto Encyclopedia of Genes and Genomes (KEGG) revealed a total of 426 enriched pathways when comparing Au-treated vs vehicle-treated samples (306 for downregulated DEGs and 120 for upregulated DEGs) (Figure 2H and Supplementary Table 5). As expected, the downregulated DEGs were primarily enriched in pathways related to cell adhesion molecules, as well as cAMP signaling, cytokine-cytokine receptor signaling, and JAK-STAT signaling.

### Correlations between Phagocytosis, Lipophilicity, and Cell Death

**Figure 3:**
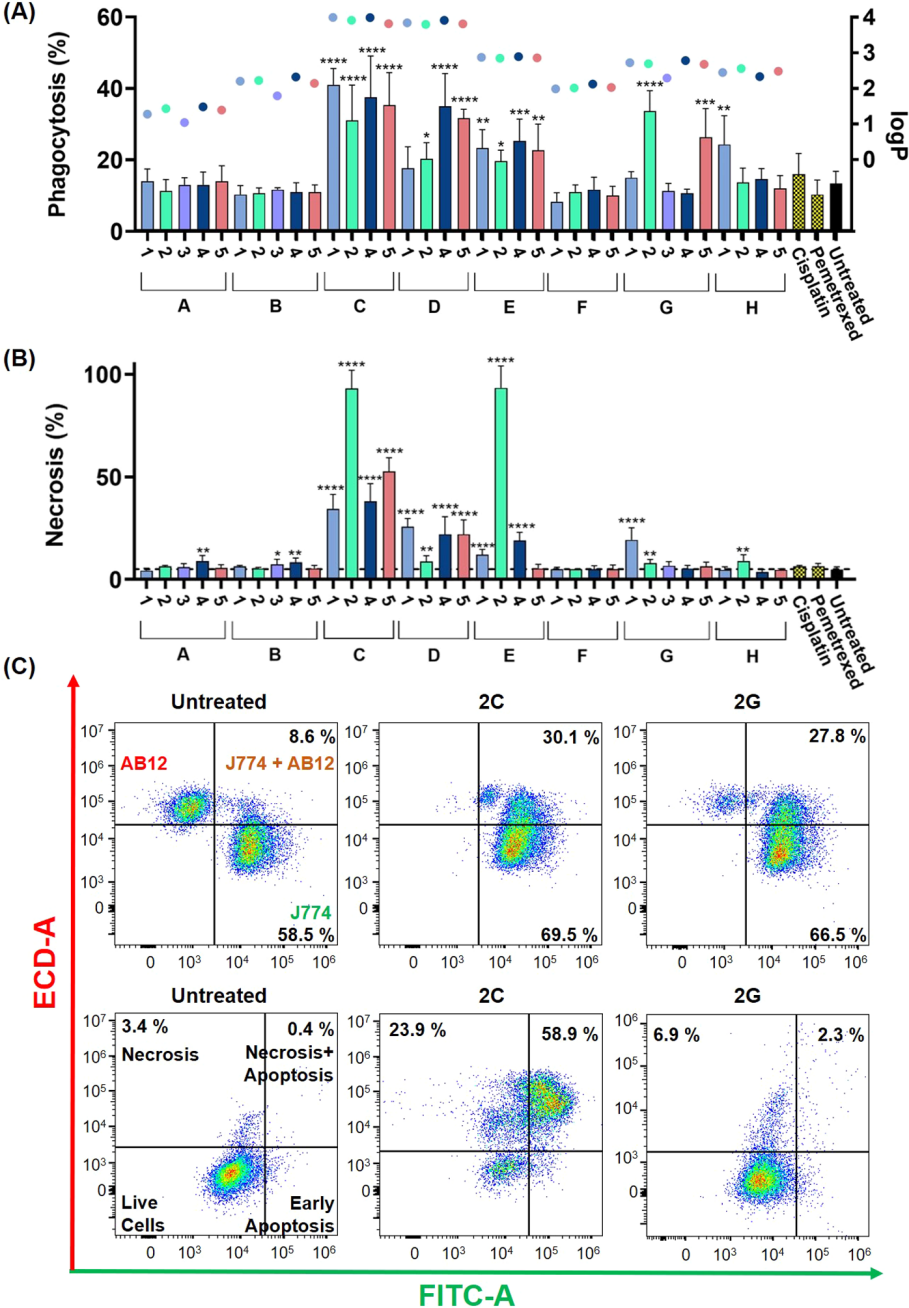
Correlations between phagocytosis, lipophilicity, and necrosis in treated AB12 cells. (**A**) Flow cytometry analysis of the phagocytosis of AB12 cells by J774 macrophages. AB12 cells were treated for 100 min at equipotent concentrations (5 × IC_50 (72 h)_) and exposed to J774 macrophages for 16 h. The phagocytosis rates were calculated by dividing the number of double-positive macrophages (Q2) by the total number of macrophages (Q2 + Q4). Inset: circular symbols correspond to the right axis and represent logP values determined using HPLC. (**B**) Flow cytometry analysis of necrosis using the Annexin V/PI assay. AB12 cells were treated for 100 min at equipotent concentrations (5 × IC_50 (72 h)_). The necrosis rate was calculated as Q1 + Q2. (**C**) Representative flow cytometry dot blots for panel A (top row) and panel B (bottom row). (**A** and **B**) Mean ± standard deviations were calculated based on at least three independent experiments. Statistical analysis was performed using the unpaired one-tailed *t*-test.

To investigate whether Au(III)–DTC complexes might induce antitumor immunity in immunologically “cold” MPM tumors, we evaluated the ability of 35 complexes to stimulate the phagocytosis of MPM cells by macrophages. The enhanced levels of phagocytosis caused by the ICD-inducing agents often lead to improved effectiveness of cancer vaccinations in murine models *in vivo.*(31) This observation can be attributed to the initiation of antitumor immune responses when immunogenic cancer cells are engulfed by immature dendritic cells or macrophages.^32^ Hence, red-stained AB12 cells were exposed to Au(III) complexes at equipotent concentrations of 5 × IC_50 (72 h)_ for only 100 min before being harvested, washed, and mixed with green-stained J774 macrophages for a 16-h coincubation. The cell mixtures were then harvested for a flow cytometry analysis. The phagocytic induction of J774 macrophages on AB12 cells was evidenced by the colocalization of both dyes within the same cell. Intriguingly, some of the tested Au(III)–DTC complexes induced high phagocytosis levels, and the most significant phagocytosis activation was observed for **1C** (41% ± 5%), **4C** (37% ± 12%), **4D** and **5C** (35% ± 9%), and **2G** (34% ± 6%) (Figure 3A, left axis). In contrast, cisplatin and pemetrexed failed to initiate any ICD response, even though pemetrexed was previously shown to trigger ICD in colon tumors.^13^ Notably, a distinct correlation emerged between phagocytosis levels and the structure of the complexes, while no correlations between phagocytosis and IC_50_ values were observed. Specifically, complexes featuring the DTC ligands **A** (-NMe_2_), **B** (-NEt_2_), and **F** (pyrrolidine-1-yl) did not trigger any phagocytic activity, whereas all complexes with ligands **C** (-NBu_2_), **D** (-NBn_2_), and **E** (-NEtPh) induced substantial phagocytosis.

Given the higher lipophilicity of ligands **C**, **D**, and **E**, as evidenced by their logP values (Supplementary Table 6), we explored potential associations between phagocytosis and the lipophilicity of the Au(III) complexes. The lipophilicity of all 35 complexes was assessed using the HPLC method in agreement with the OECD guidelines for the testing of chemicals (Figure 3A, right axis and Supplementary Figure 158).^33^ Based on the comparison of retention times in HPLC chromatograms, the lipophilicity of the complexes increased in the order **A** < **B** ≈ **F** < **G** ≈ **H** < **E** < **C** ≈ **D**, aligning perfectly with their respective phagocytosis levels.

Given that significant levels of phagocytosis were induced by Au(III) complexes only after a 100 min incubation period with cancer cells, our aim was to explore the nature of cell death triggered by Au(III) complexes using the Annexin V/PI assay under conditions identical to those of phagocytosis experiments. Notably, all complexes that stimulated phagocytosis also triggered varying levels of necrosis. Specifically, certain complexes, such as those containing the DTC ligand **C**, exhibited a correlation between elevated phagocytosis and high levels of necrosis (>35%). In contrast, complexes such as **2G** or **5G** triggered substantial phagocytosis but relatively low levels of necrosis (6%–8%) (Figure 3B).

### Release of Immunogenic Danger Signals

**Figure 4:**
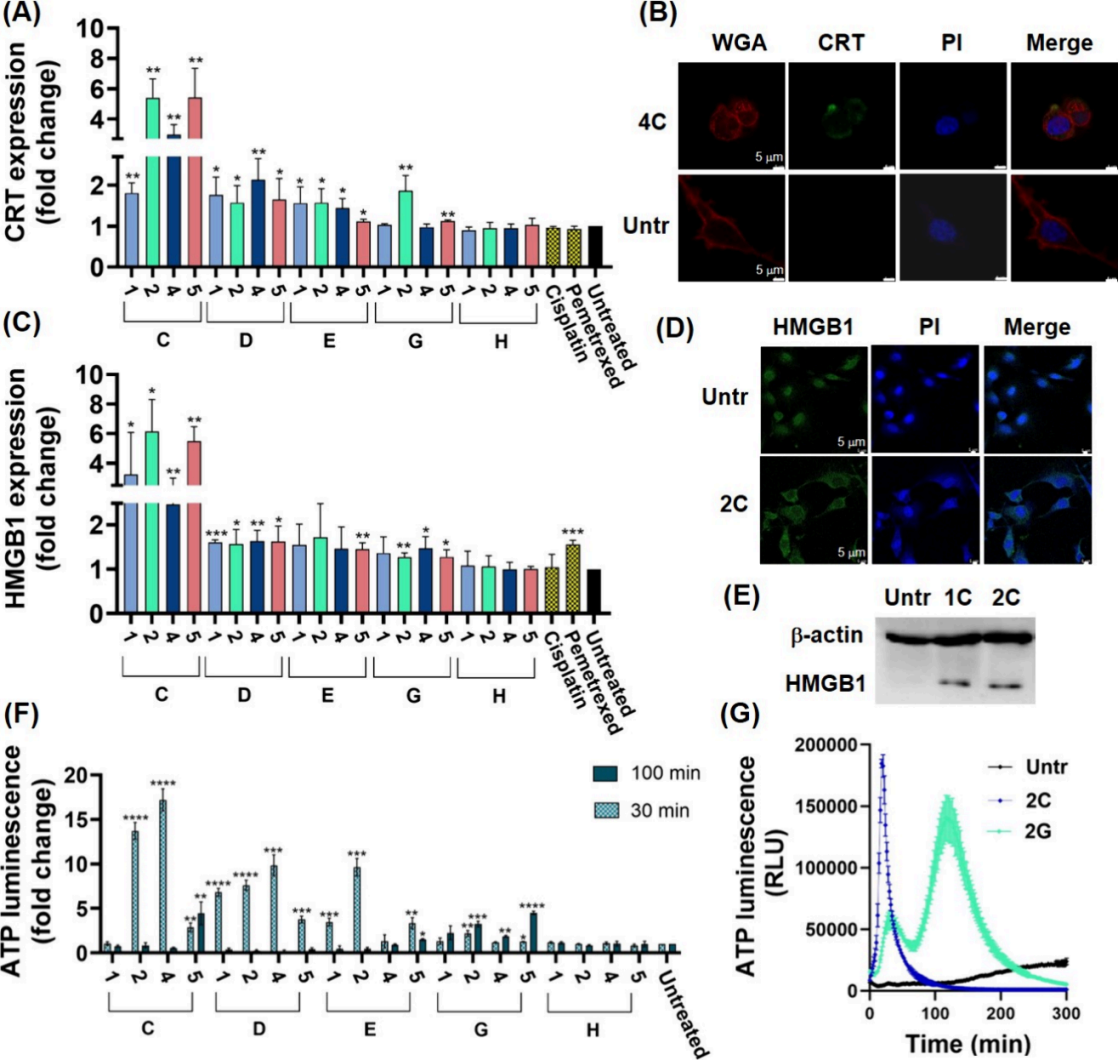
Detection of DAMPs in treated AB12 cells. (**A**) Flow cytometry analysis of CRT expression. (**B**) Confocal imaging of CRT expression (scale bar 5 μm, wheat germ agglutinin (WGA) stains the membrane; PI stains the nucleus). (**C**) Flow cytometry analysis of HMGB1 expression. (**D**) Confocal imaging of HMGB1 expression (scale bar 5 μm; PI stains the nucleus). (**E**) Western blotting of supernatants from treated AB12 cells. (**F**) Extracellular ATP-dependent luminescence. (**G**) Real-time assessment of ATP-dependent luminescence profile. AB12 cells were treated with respective compounds at equipotent concentrations (5 × IC_50 (72 h)_) for (**A**, **B**, **D**) 120 min, (**C**, **E**) 240 min, (**F**) 30 and 100 min, and (**G**) 0–300 min. (**A**, **C**, and **F**) Mean ± standard deviations were calculated based on at least three independent experiments. The results are presented as a fold change normalized to untreated control. Statistical analysis was performed using the unpaired one-tailed *t*-test.

The three most recognized hallmarks of ICD include the presentation of CRT on the cell membrane, as well as the extracellular release of HMGB1 and ATP. In its native form, CRT is localized in the ER but translocates to the cell membrane as ecto-CRT upon ICD induction.^34^ To determine the level of CRT presentation, AB12 cells were exposed to 20 selected Au(III) complexes at equipotent concentrations of 5 × IC_50 (72 h)_, as well as cisplatin and pemetrexed, for 120 min. Next, the cells were stained with an FITC-conjugated CRT antibody and a nonpermeable DNA-staining PI to exclude dead cells with a compromised membrane. Consistent with the results of the phagocytosis assay, Au(III) complexes with the DTC ligand **C** demonstrated the highest levels of CRT, showing increases of 1.8–5.4-fold compared with untreated cells (Figure 4A), followed by Au(III) complexes with the ligand **2G** (1.9-fold increase) and complexes with ligands **D** and **E** (1.1–1.8-fold increase). In contrast, no changes in CRT expression were observed when cells were treated with complexes with the ligand **H**, cisplatin, or pemetrexed. Additionally, the translocation of CRT to the plasma membrane in **4C**-treated AB12 cells was confirmed by confocal microscopy (Figure 4B).

To determine the changes in HMGB1 expression, AB12 cells were exposed to 20 Au(III) complexes at equipotent concentrations of 5 × IC_50 (72 h)_, as well as cisplatin and pemetrexed, for 240 min. The cells were then stained with a primary HMGB1 antibody and a secondary FITC-conjugated antibody. The changes in HMGB1 expression observed for all tested compounds correlated with the changes in CRT expression (Figure 4C), with the exception of pemetrexed, which demonstrated a significant increase in HMGB1 expression, in agreement with the literature.^35^ The changes in HMGB1 expression in **2C**-treated cells were confirmed by confocal microscopy (Figure 4D). Additionally, a Western Blot analysis of HMGB1 in the supernatant fractions from **1C**- and **2C**-treated cells indicated the presence of extracellular HMGB1 (Figure 4E). Subsequently, we investigated the time-dependent ATP release from AB12 cells treated with 20 Au(III) complexes at equipotent concentrations of 5 × IC_50 (72 h)_ for 300 min. As expected, complexes that triggered substantial necrosis, such as those with ligands **C** (except for **1C**), **D**, and **E**, caused a rapid ATP release within 30 min, indicating passive leakage through the damaged membrane (Figures 4F and 4G).^36^ In contrast, compounds characterized by low levels of necrosis, such as **2G**, exhibited a secondary surge in ATP release around the 100 min mark, implying an alternative mechanism for ATP release that is crucial for the induction of ICD.^37^

Given that the initiation of ER stress is a fundamental requirement for the initiation of ICD, we examined the ability of one of the Au(III)–DTC complexes, specifically **2G**, to stimulate reactive oxygen species (ROS) production within the ER and the activation of key indicators of ER stress, binding immunoglobulin protein (BiP) and C/EBP homologous protein (CHOP) (Supplementary Figures 159–160). After 240 min, AB12 cells treated with **2G** at 5 × IC_50 (72 h)_ exhibited a marked increase in H_2_DCFDA fluorescence, indicating ROS induction. As expected, the examination of the colocalization of H_2_DCFDA fluorescence with nuclear, mitochondrial, and ER dyes revealed a significant overlap of ROS with the ER tracker, indicating that the generation of ROS predominantly occurred within the ER organelle (Supplementary Figure 159). The induction of ER stress was further validated by the dose-dependent increase in BiP and CHOP, which was identified via Western Blot analysis (Supplementary Figure 160).

### *In Vivo* Immune Responses

**Figure 5:**
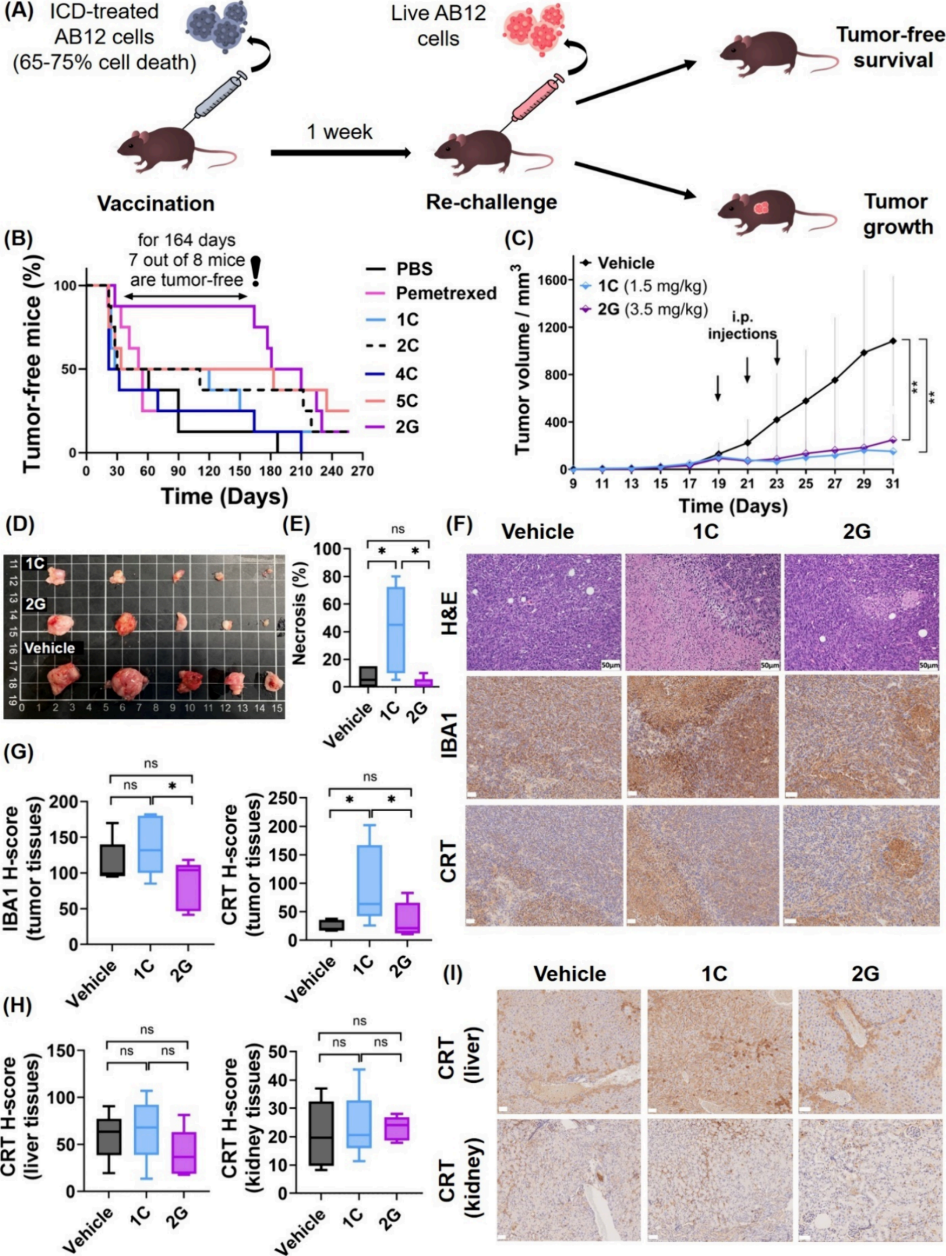
Activation of immune responses in MPM tumors by selected Au(III)–DTC complexes. (**A**) Schematic diagram illustrating the *in vivo* vaccination experiment. (**B**) The percentage of tumor-free mice that were vaccinated with 3 × 10^5^ of live/dying/dead cells pretreated with the compounds of interest (65%–75% cell death) by injection in the right flank and then rechallenged by the injection of 3 × 10^5^ of live AB12 cells in the left flank 1 week later (*n* = 8). (**C**) Time-dependent growth of AB12 tumors reflected by changes in the tumor volume. BALB/c mice were subcutaneously inoculated with AB12 cells and treated with complexes **1C** and **2G** at the indicated doses or the corresponding vehicle on Days 19, 21, and 23 via the intraperitoneal route. Tumor growth was monitored using calipers. (**D**) Corresponding tumor images at the experimental end point (Day 31). (**E**) Quantification of necrosis in H&E-stained tumor tissues. (**F**) Representative H&E- and IHC-stained tumor tissues. (**G**) Quantification of IBA1 and CRT expression in IHC-stained tumor tissues, and (**H**) quantification of CRT expression in IHC-stained liver and kidney tissues using the H-score. (**I**) IHC-stained liver and kidney tissues. (**F** and **I**) Scale bar represents 50 μm. Statistical analysis was performed using the unpaired one-tailed *t*-test.

Vaccination experiments using dying tumor cells in immunocompetent mice represent the gold standard for assessing the effects of ICD induced by different treatment modalities.^38^ Therefore, we conducted this *in vivo* experiment using the Au(III) complexes **1C**, **2C**, **4C**, **5C**, and **2G**, which were identified as the lead compounds in the *in vitro* studies, as well as pemetrexed. Initially, AB12 cells underwent pretreatment with varying concentrations of Au(III) complexes, and the induced cell death and release of desired ICD biomarkers were monitored over time. Upon reaching a cell death rate of 65%–75%, BALB/c mice (n = 8) were subcutaneously injected in the left flank with a vaccine containing 3 × 10^5^ of live/dying/dead cells, while the vehicle group received injections of PBS. Seven days postvaccine administration, the immunized mice were rechallenged by inoculating the same number of live AB12 cells (without any prior treatment) into the right flanks (Figure 5A). The progression of tumor growth on the right flanks was monitored using a caliper. Mice that did not develop tumors on the right side were classified as “tumor-free.” Notably, none of the mice developed tumor growth on the left flanks (the site of vaccine administration), thereby averting the premature termination of experiments, as documented in the literature.^15^

Within the first 30 days after vaccination, ≈50% of mice treated with PBS or complexes **1C**, **2C**, **4C**, and **5C** developed tumors on the right flank, suggesting that they are not effective inducers of ICD (Figure 5B). Similarly, four of the eight mice vaccinated with pemetrexed developed tumors by Day 51. In contrast, seven of the eight mice that were vaccinated with **2G** remained tumor-free for 164 days (5.5 months) and four of the eight mice remained tumor-free for 210 days (7 months), indicating that **2G** induced a robust and durable antitumor adaptive immune response in the immunocompetent mice.

Next, to investigate whether Au(III) complexes can induce ICD directly upon administration, rather than via vaccination, we conducted AB12 mesothelioma allograft experiments with **1C** and **2G** at their maximum soluble doses (**1C** at 1.5 mg/kg and **2G** at 3.5 mg/kg in 4% DMSO/Cremophor EL in PBS) that did not cause any weight loss or other signs of toxicity (Supplementary Figures 161 and 162). It should be noted that based on dynamic light scattering (DLS) results, complexes **1C** and **2G** formed aggregates at these concentrations, with median diameters of 715 and 289 nm, respectively (Supplementary Figure 163), reflecting differences in their lipophilicity and aqueous solubility, which is typical for Au complexes.^39^ As anticipated, both complexes significantly inhibited tumor growth in comparison with the vehicle-treated mice and exhibited comparable organ biodistribution profiles (Figures 5C and 5D and Supplementary Figure 164) to those of previously discussed complexes with scaffold **5**. Similarly, a histological examination of the livers and kidneys indicated no signs of Au-induced nephrotoxicity or hepatotoxicity (Supplementary Figures 165 and 166). While **1C** and **2G** exhibited similar tumor burden-reducing effects, significant differences were observed upon histological examination of tumor tissues (Figures 5E-5G, Supplementary Figure 167). **2G** did not induce any necrosis in tumors; in contrast, **1C** led to significant necrosis, in line with our *in vitro* findings (Figure 5E).

Considering the potential macrophage toxicity of tested Au(III) complexes, we analyzed macrophage population in freshly isolated tumors using Fluorescence-Activated Cell Sorting (FACS) (Supplementary Figure 168). Macrophages were identified as the CD64^+^(FcγRI)/CD11b^+^ double-positive cell subset. The analysis indicated no significant differences in macrophage populations within the tumors. Subsequently, we performed immunohistochemistry (IHC) analysis of the expression levels of CRT and the ionized calcium-binding adapter molecule 1 (IBA1) in tumor tissues treated with **1C**, **2G**, and the corresponding vehicle (Figures 5F and 5G). IBA1 is a frequently used marker of the phagocytic activity of brain macrophages; however, it can also be utilized for the quantification of macrophages in MPM tumors, given that AB12 tumor tissues were observed to be substantially infiltrated by IBA1^+^ macrophages.^40^ Biomarker expression was quantified using an automated QuPath algorithm to generate an H-score,^41^ which integrates both the intensity and distribution of the IHC staining. Initially, the tissue regions of interest were delineated and segmented to distinguish staining areas from the background. Following this, individual tumor cells within the segmented areas were identified to evaluate the staining intensity and distribution. To ensure that the quantification of the biomarker expression was specifically conducted in non-necrotic areas, the areas of necrosis were morphologically analyzed and segregated by a board-certified pathologist. The assessment of the H-score for IBA1 staining indicated no statistically significant differences between vehicle- and Au-treated tumors, implying that the overall number of macrophages remained consistent, in agreement with flow cytometry analysis (Figure 5G). In contrast, the H-score for CRT staining exhibited notably elevated expression in the non-necrotic regions of tumor tissues derived from **1C**-treated mice (Figures 5F and 5G), suggesting a pro-phagocytic response of the tumors to the treatment. Finally, to investigate whether **1C** affected CRT expression in nontumor tissues, we performed IHC staining of kidney and liver tissues (Figures 5H and 5I). The analysis of the CRT H-score revealed no changes in expression across three treatment groups, suggesting that **1C** selectively activated *in vivo* immunogenic responses only in tumor tissues.

## Discussion

Since our discovery of the first metal-based Type II ICD inducer,^42^ a large number of compounds featuring diverse metal centers, in particular Pt and Au centers, have exhibited excellent ICD-inducing properties both *in vitro* and *in vivo.*(43−47) However, the majority of these compounds have been discovered serendipitously. Previously, we performed various structural modifications of a metal-based scaffold of an ICD inducer to explore the distinct features associated with their ICD-inducing properties.^48^ We demonstrated that the extent of ICD induction was dependent on the effectiveness of ROS generation localized in the ER and attributed the superior immunogenic activity to a stable carbene scaffold.^48^ However, the compound library was limited to seven compounds, and their ICD-inducing properties have not been tested *in vivo*. Subsequently, Senthilkumar, Kumar, and Patil et al. tested a library of 40 benzo[*a*]quinolizinium Au(I)–NHC complexes and identified the lead Au compound with excellent *in vitro* ICD-inducing properties. Nevertheless, the specific structural components responsible for eliciting these ICD-inducing properties have not been identified.^22^

We hypothesized that cyclometalated Au(III) complexes featuring bidentate polyaromatic C^N and DTC-chelating ligands might serve as a good model for elucidating potential ICD-inducing properties and the structure–activity relationships in the context of ICD. The diversity in cyclometalated scaffolds and DTC ligands enables the fine-tuning of the physicochemical properties of the resulting Au(III) complexes,^49,50^ such as the lipophilicity, ligand bite angle, and electronic density of the central metal atom. Moreover, cyclometalated Au(III) complexes have previously demonstrated significant cytotoxicity in cancer cells by interacting with key proteins involved in the maintenance of cellular redox balance and ER homeostasis,^51−59^ thereby triggering ER stress.^54^ We have successfully synthesized and characterized 35 Au(III)–DTC complexes and evaluated their ability to induce phagocytosis *in vitro* against immunologically “cold” MPM cells (Figure 3). Importantly, the phagocytosis levels were strongly dependent on the cyclometalated scaffold and the overall lipophilicity of the complexes. One plausible explanation for this strong correlation could be that, for the induction of targeted immunogenic effects leading to phagocytosis activation, complexes first induce ER stress.^34,60^ The ER is recognized for its lipid-rich membrane; therefore, the increase in the lipophilic nature of molecules would lead to enhanced ER targeting and accumulation.^61^

Our next goal was to identify the relationship between the levels of phagocytosis and the extent of cell death induced by the tested compounds. It is widely recognized that cancer cells must undergo a form of programmed cell death to initiate ICD.^61^ However, even in instances of necrotic cell death, certain immunogenic responses can still manifest after vaccination.^62^ These responses are usually less potent and efficient in eliciting a robust, adaptive, and long-term immune reaction to vaccination^62,63,64^ than responses from *bona fide* ICD inducers, which trigger ICD without directly causing the death of cancer cells. Following the quantification of necrotic cell death caused by Au(III)–DTC complexes after a short exposure of 120 min, we classified all complexes into three distinct groups based on their effects: Group 1 consisted of compounds that induced neither phagocytosis nor necrosis (scaffolds **A**, **B**, **F**, and **H**); Group 2 consisted of compounds that elicited high levels of both phagocytosis and necrosis (scaffolds **C**, **D**, and **E**); and Group 3 included compounds, such as **2D**, **2G**, and **1H**, that induced high levels of phagocytosis but did not result in significant necrotic cell death (Figure 3). The complexes from Group 2 and Group 3 were additionally screened for DAMPs release (Figure 4). Those complexes that elevated CRT expression, along with causing HMGB1 and ATP release, were then selected for further *in vivo* vaccination experiments.

Satisfyingly, seven of the eight immunocompetent BALB/c mice immunized with **2G** remained tumor-free for 164 days (5.5 months) and four of the eight mice stayed tumor-free for 210 days (7 months), demonstrating a potent and long-lasting antitumor adaptive immune reaction (Figure 5). These results indicate that **2G** can be characterized as a *bona fide* ICD inducer. In contrast, ≈50% of mice treated with complexes **1C**, **2C**, **4C**, and **5C** from Group 2 developed MPM tumors. Notably, a tumor-free survival period of 5–7 months holds clinical significance for patients with MPM. For example, in the CheckMate 743 trial, a phase III randomized study, patients with unresectable MPM who received immunotherapy (nivolumab/ipilimumab) demonstrated a 4-month extension in overall survival in comparison with those who received the standard-of-care cisplatin-pemetrexed chemotherapy.^7,65^

In previous studies by Liu et al., selected Au(I) complexes were shown to simultaneously induce ICD and activation of innate or adaptive immune responses, thereby providing the foundation for their potential use as chemoimmunotherapies.^19,20^ Hence, we investigated whether selected Au(III) complexes from Groups 2 and 3 could induce ICD directly upon administration, rather than via vaccination (Figure 5). Complexes **1C** and **2G** demonstrated prominent efficacy against AB12 MPM tumors. While complex **1C** caused marked levels of necrosis, affecting up to 80% of the tissue, **2G** did not cause significant necrotization of tumor tissues, with levels ranging from 0%–10%, consistent with the results of the *in vitro* studies. Interestingly, the remaining non-necrotic tumor tissues from **1C**-treated mice were characterized by a significant increase in CRT expression, indicating ICD activation. It should be noted that elevated CRT expression in various tissues has been linked to the development of fibrosis,^68,69^ contributing to chronic conditions in patients. Hence, we also examined CRT expression in Au-treated liver and kidney tissues and found no substantial changes in CRT expression in either organ, indicating that **1C** specifically triggered CRT activation solely in tumor tissues.

From these findings, we can infer that highly lipophilic Au complexes like **1C**, which triggered significant production of authentic necrotic cells, i.e. those cells that die with membrane rupture, were weakly pro-inflammatory and somewhat immunogenic. Their immunogenic properties were primarily evident upon direct administration rather than through signals from dying cells. Conversely, complexes like **2G**, which caused minimal cell rupture but displayed specific surface markers like phosphatidyl serine (PS), as detected by Annexin V, were genuinely immunogenic. However, when directly administered, these complexes did not exhibit strong DAMP signals. These results suggest that necrosis per se cannot be categorized as a strictly negative or positive feature of ICD; however, for robust immunogenicity, programmed necrosis appears to be distinctly advantageous.^70,71^

One possible concern regarding the use of Au complexes as immune-activating agents for the treatment of MPM could be their potential toxicity to immune cells. This concern stems from the common clinical use of Au-based drugs in treating rheumatoid arthritis due to their strong immunosuppressive effects.^72^ It is important to emphasize that *bona fide* ICD inducers like **2G** are expected to activate immune system indirectly, i.e. not through the direct administration of drugs, but rather via vaccination with dying cancer cells. This vaccination method ensures that any potential immunosuppressive properties are not a relevant concern. In contrast, when Au complexes are administered directly via the i.p. route, the consideration of their potential immunosuppressive properties becomes highly relevant. Therefore, we conducted a comparative analysis of the cytotoxicity of several Au complexes toward THP-1-derived M0 macrophages and examined the macrophage population in freshly isolated tumors from mice treated with **1C** and **2G** using flow cytometry and IHC. Although the differentiated M0 macrophages were highly sensitive to the tested Au complexes, no significant variations in macrophage numbers within the tumors were observed, indicating an unharmed macrophage population. In future, to avoid the potential harm that novel Au-based ICD inducers may pose to immune cells, bio-orthogonal or photoactivated Au-based prodrug strategies could be employed.^73,74^ These strategies enable targeted activation within tumors, while preserving the systemic immune system.

## Conclusions

In this study, we, for the first time, shed light on the underlying reasons why some metal complexes induce ICD, while others do not. We provided the foundation for understanding how *in vitro* screening can predict potential *in vivo* inducers of ICD in MPM tumors and elucidated the role of the cell death mechanisms in the observed *in vivo* ICD effects. We identified an Au(III)–DTC complex that induced a robust immune response in immunocompetent MPM tumor-bearing mice for more than half a year. Moreover, our findings highlighted the potential of Au complexes as promising therapeutic candidates for the treatment of MPM, as they exhibited superior effects to the conventional cisplatin-pemetrexed regimen both *in vitro* and *in vivo*. Importantly, they demonstrated high efficacy across cell lines derived from patients with epithelioid, sarcomatoid, and mixed biphasic types of MPM. For future research, it is essential to elucidate the biomolecular targets of metal-based ICD inducers and establish correlations between their physicochemical properties and these targets. This approach may provide additional valuable insights for predicting the ICD-inducing activity of compounds *in vivo*. Without this crucial link, the discovery of ICD inducers will remain serendipitous.

## Data Availability

All relevant data supporting the findings of this study are available within the article and its Supporting Information.

## Supporting Information Available

Table S3: Full list of upregulated genes (XLSX)
Table S4: Full list of downregulated genes (XLSX)
Table S5: KEGG pathway enrichment analysis (XLSX)
Table S7: IHC data (XLSX)
Materials and methods; instrumentation; general procedure for synthesis of cyclometalated Au(III) dithiocarbamate; X-ray crystallography; RP-HPLC analysis; dynamic light scattering (DLS) experiments; cell lines and culture conditions; inhibition of cell viability assay; flow cytometry experiments; confocal microscopy experiments; Western Blotting experiments; RealTime-Glo extracellular ATP assay; animal experiments; tumor analysis; statistical analysis; crystallographic data for an Au(III) complex **1H**; cytotoxicity of Au(III) complexes in comparison with cisplatin against MPM cell lines and nonmalignant lung fibroblasts; logP values determined from retention time using RP-HPLC; ^1^H and ^13^C NMR spectra; high-resolution ESI-MS spectra; stability spectra in water/DMSO 1:1; concentration-effect curves of 35 Au(III)-DTC complexes; body weight changes of Balb/C mice; representative H&E-stained images of liver, kidney and tumor sections; quantification of total percentage of macrophages in tumors freshly isolated from all mice (PDF)
