# Supplementary material for: Leveraging Immunogenic Cell Death to Enhance the Immune Response against Malignant Pleural Mesothelioma Tumors
Source: J Am Chem Soc. 2025 Feb 24;147(9):7908–20. doi: 10.1021/jacs.4c17966 (PMC11887451; doi:10.1021/jacs.4c17966)
Supplement: Supplementary file 5 — ja4c17966_si_005.pdf [file ja4c17966_si_005.pdf]

Supplementary information

for

# Leveraging immunogenic cell death to enhance the immune response against malignant pleural mesothelioma tumors

Meng Rui Chang,[a,≠] Egor M. Matnurov,[b,≠] Chengnan Wu,[b,≠] Jemma Arakelyan,[b] Ho-Jung Choe,[b] Vladimir Kushnarev,[b] Jian Yu Yap,[a,c] Xiu Xuan Soo,[a] Mun Juinn Chow,[a,d] Walter Berger,[e,*] Wee Han Ang,[a,c,*] Maria V. Babak[b,*]

[a] Department of Chemistry, National University of Singapore, 4 Science Drive 2, Singapore, 117543, Singapore

[b] Drug Discovery Lab, Department of Chemistry, City University of Hong Kong, 83 Tat Chee Avenue, Hong Kong SAR, 999077, People's Republic of China

[c] NUS Graduate School - Integrated Science and Engineering Programme (ISEP), National University of Singapore, Singapore, 119077, Singapore

[d] A*Star Skin Research Labs (A*SRL), Singapore 308232, Singapore

[e] Center for Cancer Research and Comprehensive Cancer Center, Medical University of Vienna, Borschkegasse 8A, Vienna 1090, Austria

≠ equally contributing authors

* Corresponding authors. E-mail: walter.berger@meduniwien.ac.at (Walter Berger), ang.weehan@nus.edu.sg (Wee Han Ang), mbabak@cityu.edu.hk (Maria V. Babak)

# Table of Contents

**Materials and methods** .......... **S9**

**Instrumentation** .......... **S9**

**General procedure for synthesis of cyclometalated Au(III) dithiocarbamate complexes** .......... **S10**

**X-ray crystallography** .......... **S18**

**RP-HPLC analysis** .......... **S19**

**Dynamic light scattering (DLS) experiments** .......... **S19**

**Cell lines and culture conditions** .......... **S20**

**Inhibition of cell viability assay** .......... **S20**

**Flow cytometry experiments** .......... **S21**

- **Phagocytosis assay** .......... **S21**
- **Annexin V/PI assay** .......... **S22**
- **CRT detection** .......... **S23**
- **HMGB1 detection** .......... **S23**
- **Fluorescence-activated cell sorting of macrophages** .......... **S24**

**Confocal microscopy experiments** .......... **S25**

- **CRT detection** .......... **S25**
- **HMGB1 detection** .......... **S26**
- **ROS detection** .......... **S26**

**Western Blotting experiments** .......... **S27**

**RealTime-Glo extracellular ATP assay** .......... **S29**

**Animal experiments** .......... **S29**

- ***In vivo* AB12 mouse allograft model (*i.p.* route)** .......... **S30**
- ***In vivo* vaccination model** .......... **S31**

**Tumor analysis** .......... **S32**

- **RNA sequencing** .......... **S32**
- **Histopathological analysis** .......... **S33**
- **Organ distribution studies** .......... **S33**

**Statistical analysis** .......... **S34**

**Supplementary Table 1.** Crystallographic data for an Au(III) complex **1H**. .......... **S35**

**Supplementary Table 2.** Cytotoxicity of Au(III) complexes in comparison with cisplatin against MPM cell lines and non-malignant lung fibroblasts. .......... **S36**

**Supplementary Table 3.** Up-regulated differentially expressed genes in tumors isolated from Balb/C mice treated with **5A** (saved as a separate excel file) ..........

**Supplementary Table 4.** Down-regulated differentially expressed genes in tumors isolated from Balb/C mice treated with **5A** (saved as a separate excel file) ..........................................

**Supplementary Table 5.** KEGG pathway enrichment analysis for differentially expressed genes in tumors isolated from Balb/C mice treated with **5A** (saved as a separate excel file) ....

**Supplementary Table 6.** LogP values determined from retention time using RP-HPLC. .**S38**

**Supplementary Table 7.** Analysis of IHC-stained expression of CRT and IBA1 in tumors, kidneys and livers isolated from Balb/C mice treated with **1C** and **2G** (saved as a separate file).........................................................................................................................

**Supplementary Figure 1**. RP-HPLC analysis of **1A**. .......................................................**S39**

**Supplementary Figure 2**. RP-HPLC analysis of **1B**. ........................................................**S39**

**Supplementary Figure 3**. RP-HPLC analysis of **1C**. .......................................................**S40**

**Supplementary Figure 4**. RP-HPLC analysis of **1D**. .......................................................**S40**

**Supplementary Figure 5**. RP-HPLC analysis of **1E**. ........................................................**S41**

**Supplementary Figure 6**. RP-HPLC analysis of **1F**. ........................................................**S41**

**Supplementary Figure 7**. RP-HPLC analysis of **1G**. .......................................................**S42**

**Supplementary Figure 8**. RP-HPLC analysis of **1H**. .......................................................**S42**

**Supplementary Figure 9**. RP-HPLC analysis of **2A**. ....................................................... **S43**

**Supplementary Figure 10**. RP-HPLC analysis of **2B**. ...................................................... **S43**

**Supplementary Figure 11**. RP-HPLC analysis of **2C**. ..................................................... **S44**

**Supplementary Figure 12**. RP-HPLC analysis of **2D**. ..................................................... **S44**

**Supplementary Figure 13**. RP-HPLC analysis of **2E**. ...................................................... **S45**

**Supplementary Figure 14**. RP-HPLC analysis of **2F**. ...................................................... **S45**

**Supplementary Figure 15**. RP-HPLC analysis of **2G**. ..................................................... **S46**

**Supplementary Figure 16**. RP-HPLC analysis of **2H**. ..................................................... **S46**

**Supplementary Figure 17**. RP-HPLC analysis of **3A**. ..................................................... **S47**

**Supplementary Figure 18**. RP-HPLC analysis of **3B**. ...................................................... **S47**

**Supplementary Figure 19**. RP-HPLC analysis of **3G**. ..................................................... **S47**

**Supplementary Figure 20**. RP-HPLC analysis of **4A.** .................................................... **S48**

**Supplementary Figure 21**. RP-HPLC analysis of **4B.** ..................................................... **S48**

**Supplementary Figure 22**. RP-HPLC analysis of **4C.** ..................................................... **S49**

**Supplementary Figure 23**. RP-HPLC analysis of **4D**. ..................................................... **S49**

**Supplementary Figure 24**. RP-HPLC analysis of **4E**. ...................................................... **S50**

**Supplementary Figure 25**. RP-HPLC analysis of **4F.** ...................................................... **S50**

**Supplementary Figure 26**. RP-HPLC analysis of **4G**. ..................................................... **S51**

**Supplementary Figure 27**. RP-HPLC analysis of **4H.** ..................................................... **S51**

**Supplementary Figure 28**. RP-HPLC analysis of **5A**. ..................................................... **S52**

**Supplementary Figure 29**. RP-HPLC analysis of **5B**. ........ **S52**
**Supplementary Figure 30**. RP-HPLC analysis of **5C**. ........ **S53**
**Supplementary Figure 31**. RP-HPLC analysis of **5D**. ........ **S53**
**Supplementary Figure 32**. RP-HPLC analysis of **5E**. ........ **S54**
**Supplementary Figure 33**. RP-HPLC analysis of **5F**. ........ **S54**
**Supplementary Figure 34**. RP-HPLC analysis of **5G**. ........ **S55**
**Supplementary Figure 35**. RP-HPLC analysis of **5H**. ........ **S55**
**Supplementary Figure 36**. $^{1}$H NMR of **1D** in DMSO-d$^{6}$. ........ **S56**
**Supplementary Figure 37**. $^{1}$H NMR of **1E** in DMSO-d$^{6}$. ........ **S57**
**Supplementary Figure 38**. $^{1}$H NMR of **1F** in DMSO-d$^{6}$. ........ **S58**
**Supplementary Figure 39**. $^{1}$H NMR of **1G** in DMSO-d$^{6}$. ........ **S59**
**Supplementary Figure 40**. $^{1}$H NMR of **1H** in DMSO-d$^{6}$. ........ **S60**
**Supplementary Figure 41**. $^{1}$H NMR of **2C** in DMSO-d$^{6}$. ........ **S61**
**Supplementary Figure 42**. $^{1}$H NMR of **2D** in DMSO-d$^{6}$. ........ **S62**
**Supplementary Figure 43**. $^{1}$H NMR of **2E** in DMSO-d$^{6}$. ........ **S63**
**Supplementary Figure 44**. $^{1}$H NMR of **2F** in DMSO-d$^{6}$. ........ **S64**
**Supplementary Figure 45**. $^{1}$H NMR of **2G** in DMSO-d$^{6}$. ........ **S65**
**Supplementary Figure 46**. $^{1}$H NMR of **3G** in DMSO-d$^{6}$. ........ **S66**
**Supplementary Figure 47**. $^{1}$H NMR of **4A** in DMSO-d$^{6}$. ........ **S67**
**Supplementary Figure 48**. $^{1}$H NMR of **4B** in DMSO-d$^{6}$. ........ **S68**
**Supplementary Figure 49**. $^{1}$H NMR of **4C** in ACN-d$^{3}$. ........ **S69**
**Supplementary Figure 50**. $^{1}$H NMR of **4D** in ACN-d$^{3}$. ........ **S70**
**Supplementary Figure 51**. $^{1}$H NMR of **4E** in DMSO-d$^{6}$. ........ **S71**
**Supplementary Figure 52**. $^{1}$H NMR of **4F** in ACN-d$^{3}$. ........ **S72**
**Supplementary Figure 53**. $^{1}$H NMR of **4G** in DMSO-d$^{6}$. ........ **S73**
**Supplementary Figure 54**. $^{1}$H NMR of **4H** in DMSO-d$^{6}$. ........ **S74**
**Supplementary Figure 55**. $^{1}$H NMR of **5A** in DMSO-d$^{6}$. ........ **S75**
**Supplementary Figure 56**. $^{1}$H NMR of **5B** in DMSO-d$^{6}$. ........ **S76**
**Supplementary Figure 57**. $^{1}$H NMR of **5C** in ACN-d$^{3}$. ........ **S77**
**Supplementary Figure 58**. $^{1}$H NMR of **5D** in DMSO-d$^{6}$. ........ **S78**
**Supplementary Figure 59**. $^{1}$H NMR of **5E** in DMSO-d$^{6}$. ........ **S79**
**Supplementary Figure 60**. $^{1}$H NMR of **5G** in DMSO-d$^{6}$. ........ **S80**
**Supplementary Figure 61.** $^{13}$C NMR of **1D** in DMSO-d$^{6}$. ........ **S81**
**Supplementary Figure 62.** $^{13}$C NMR of **1E** in DMSO-d$^{6}$. ........ **S82**
**Supplementary Figure 63.** $^{13}$C NMR of **1F** in DMSO-d$^{6}$. ........ **S83**

**Supplementary Figure 64.** $^{13}C$ NMR of **1G** in DMSO-d$^6$. ................................................ **S84**
**Supplementary Figure 65.** $^{13}C$ NMR of **1H** in DMSO-d$^6$. ................................................ **S85**
**Supplementary Figure 66.** $^{13}C$ NMR of **2C** in DMSO-d$^6$. ................................................ **S86**
**Supplementary Figure 67.** $^{13}C$ NMR of **2D** in DMSO-d$^6$. ................................................ **S87**
**Supplementary Figure 68.** $^{13}C$ NMR of **2E** in $CDCl_3$. ...................................................... **S88**
**Supplementary Figure 69.** $^{13}C$ NMR of **2F** in DMSO-d$^6$. ................................................ **S89**
**Supplementary Figure 70.** $^{13}C$ NMR of **2G** in DMSO-d$^6$. ................................................ **S90**
**Supplementary Figure 71.** $^{13}C$ NMR of **3G** in DMSO-d$^6$. ................................................ **S91**
**Supplementary Figure 72.** $^{13}C$ NMR of **4A** in DMSO-d$^6$. ................................................ **S92**
**Supplementary Figure 73.** $^{13}C$ NMR of **4B** in DMSO-d$^6$. ................................................ **S93**
**Supplementary Figure 74.** $^{13}C$ NMR of **4C** in DMSO-d$^6$. ................................................ **S94**
**Supplementary Figure 75.** $^{13}C$ NMR of **4D** in DMSO-d$^6$. ................................................ **S95**
**Supplementary Figure 76.** $^{13}C$ NMR of **4E** in DMSO-d$^6$. ................................................ **S96**
**Supplementary Figure 77.** $^{13}C$ NMR of **4F** in DMSO-d$^6$. ................................................ **S97**
**Supplementary Figure 78.** $^{13}C$ NMR of **4G** in DMSO-d$^6$. ................................................ **S98**
**Supplementary Figure 79.** $^{13}C$ NMR of **4H** in DMSO-d$^6$. ................................................ **S99**
**Supplementary Figure 80.** $^{13}C$ NMR of **5A** in DMSO-d$^6$. ...............................................**S100**
**Supplementary Figure 81.** $^{13}C$ NMR of **5B** in DMSO-d$^6$. .............................................. **S101**
**Supplementary Figure 82.** $^{13}C$ NMR of **5C** in DMSO-d$^6$. .............................................. **S102**
**Supplementary Figure 83.** $^{13}C$ NMR of **5D** in DMSO-d$^6$. .............................................. **S103**
**Supplementary Figure 84.** $^{13}C$ NMR of **5E** in DMSO-d$^6$. .............................................. **S104**
**Supplementary Figure 85.** $^{13}C$ NMR of **5G** in DMSO-d$^6$. .............................................. **S105**
**Supplementary Figure 86.** High resolution ESI-MS spectrum of **1D**. ............................ **S106**
**Supplementary Figure 87.** High resolution ESI-MS spectrum of **1E**. ............................ **S106**
**Supplementary Figure 88.** High resolution ESI-MS spectrum of **1F**. ............................ **S106**
**Supplementary Figure 89.** High resolution ESI-MS spectrum of **1G**. ............................ **S107**
**Supplementary Figure 90.** High resolution ESI-MS spectrum of **1H**. ............................ **S107**
**Supplementary Figure 91.** High resolution ESI-MS spectrum of **2C**. ............................ **S107**
**Supplementary Figure 92.** High resolution ESI-MS spectrum of **2D**. .............................**S108**
**Supplementary Figure 93.** High resolution ESI-MS spectrum of **2E**. ............................ **S108**
**Supplementary Figure 94.** High resolution ESI-MS spectrum of **2F**. ............................ **S108**
**Supplementary Figure 95.** High resolution ESI-MS spectrum of **2G**. ............................ **S109**
**Supplementary Figure 96.** High resolution ESI-MS spectrum of **3G**. ............................ **S109**
**Supplementary Figure 97.** High resolution ESI-MS spectrum of **4A**. ............................ **S109**
**Supplementary Figure 98.** High resolution ESI-MS spectrum of **4B**. ............................ **S110**

**Supplementary Figure 99.** High resolution ESI-MS spectrum of **4C**. ........................... **S110**
**Supplementary Figure 100.** High resolution ESI-MS spectrum of **4D**. .......................... **S110**
**Supplementary Figure 101.** High resolution ESI-MS spectrum of **4E**. .......................... **S111**
**Supplementary Figure 102.** High resolution ESI-MS spectrum of **4F**. .......................... **S111**
**Supplementary Figure 103.** High resolution ESI-MS spectrum of **4G**. ..........................**S111**
**Supplementary Figure 104.** High resolution ESI-MS spectrum of **4H**. ..........................**S112**
**Supplementary Figure 105.** High resolution ESI-MS spectrum of **5A**. ...........................**S112**
**Supplementary Figure 106.** High resolution ESI-MS spectrum of **5B**. ............................**S112**
**Supplementary Figure 107.** High resolution ESI-MS spectrum of **5C**. ...........................**S113**
**Supplementary Figure 108.** High resolution ESI-MS spectrum of **5D**. ...........................**S113**
**Supplementary Figure 109.** High resolution ESI-MS spectrum of **5E**. ............................**S113**
**Supplementary Figure 110.** High resolution ESI-MS spectrum of **5G**. ...........................**S114**
**Supplementary Figure 111.** Molecular structure of **1H** ...................................................**S114**
**Supplementary Figure 112**. Stability of **1A** in water/DMSO 1:1 using RP-HPLC. ........**S115**
**Supplementary Figure 113**. Stability of **1B** in water/DMSO 1:1 using RP-HPLC. .........**S115**
**Supplementary Figure 114**. Stability of **1C** in water/DMSO 1:1 using RP-HPLC. ........**S116**
**Supplementary Figure 115**. Stability of **1D** in water/DMSO 1:1 using RP-HPLC. ........**S116**
**Supplementary Figure 116**. Stability of **1E** in water/DMSO 1:1 using RP-HPLC. .........**S117**
**Supplementary Figure 117.** Stability of **1F** in water/DMSO 1:1 using RP-HPLC. .........**S117**
**Supplementary Figure 118**. Stability of **1G** in water/DMSO 1:1 using RP-HPLC. ...... **S118**
**Supplementary Figure 119**. Stability of **1H** in water/DMSO 1:1 using RP-HPLC. ....... **S118**
**Supplementary Figure 120**. Stability of **2A** in water/DMSO 1:1 using RP-HPLC. ....... **S119**
**Supplementary Figure 121**. Stability of **2B** in water/DMSO 1:1 using RP-HPLC. ........ **S119**
**Supplementary Figure 122**. Stability of **2C** in water/DMSO 1:1 using RP-HPLC. ....... **S120**
**Supplementary Figure 123.** Stability of **2D** in water/DMSO 1:1 using RP-HPLC. ...... **S120**
**Supplementary Figure 124**. Stability of **2E** in water/DMSO 1:1 using RP-HPLC. ....... **S121**
**Supplementary Figure 125**. Stability of **2F** in water/DMSO 1:1 using RP-HPLC. ........ **S121**
**Supplementary Figure 126**. Stability of **2G** in water/DMSO 1:1 using RP-HPLC. ....... **S122**
**Supplementary Figure 127**. Stability of **2H** in water/DMSO 1:1 using RP-HPLC. ....... **S122**
**Supplementary Figure 128**. Stability of **3A** in water/DMSO 1:1 using RP-HPLC. ....... **S123**
**Supplementary Figure 129**. Stability of **3B** in water/DMSO 1:1 using RP-HPLC. ........ **S123**
**Supplementary Figure 130.** Stability of **3G** in water/DMSO 1:1 using RP-HPLC. ....... **S124**
**Supplementary Figure 131**. Stability of **4A** in water/DMSO 1:1 using RP-HPLC. ...... **S124**
**Supplementary Figure 132**. Stability of **4B** in water/DMSO 1:1 using RP-HPLC. ....... **S125**
**Supplementary Figure 133**. Stability of **4C** in water/DMSO 1:1 using RP-HPLC. ...... **S125**

**Supplementary Figure 134**. Stability of **4D** in water/DMSO 1:1 using RP-HPLC. ...... **S126**
**Supplementary Figure 135**. Stability of **4E** in water/DMSO 1:1 using RP-HPLC. ....... **S126**
**Supplementary Figure 136**. Stability of **4F** in water/DMSO 1:1 using RP-HPLC. ....... **S127**
**Supplementary Figure 137**. Stability of **4G** in water/DMSO 1:1 using RP-HPLC. ....... **S127**
**Supplementary Figure 138**. Stability of **4H** in water/DMSO 1:1 using RP-HPLC. ...... **S128**
**Supplementary Figure 139**. Stability of **5A** in water/DMSO 1:1 using RP-HPLC. ....... **S128**
**Supplementary Figure 140**. Stability of **5B** in water/DMSO 1:1 using RP-HPLC. ........ **S129**
**Supplementary Figure 141.** Stability of **5C** in water/DMSO 1:1 using RP-HPLC. ....... **S129**
**Supplementary Figure 142**. Stability of **5D** in water/DMSO 1:1 using RP-HPLC. ...... **S130**
**Supplementary Figure 143**. Stability of **5E** in water/DMSO 1:1 using RP-HPLC. ........ **S130**
**Supplementary Figure 144**. Stability of **5F** in water/DMSO 1:1 using RP-HPLC. ........ **S131**
**Supplementary Figure 145**. Stability of **5G** in water/DMSO 1:1 using RP-HPLC. ...... **S131**
**Supplementary Figure 146**. Stability of **5H** in water/DMSO 1:1 using RP-HPLC. ....... **S132**
**Supplementary Figure 147.** Concentration-effect curves of 35 Au(III)-DTC complexes in a murine AB12 MPM cell line in comparison with cisplatin and pemetrexed. .................... **S133**
**Supplementary Figure 148.** Concentration-effect curves of 35 Au(III)-DTC complexes in a human JU77 MPM cell line in comparison with cisplatin and pemetrexed. ..................... **S134**
**Supplementary Figure 149.** Concentration-effect curves of 35 Au(III)-DTC complexes in a human LO68 MPM cell line in comparison with cisplatin and pemetrexed. .................... **S135**
**Supplementary Figure 150.** Concentration-effect curves of 35 Au(III)-DTC complexes in a patient-derived VMC23 cell line in comparison with cisplatin and pemetrexed. .............. **S136**
**Supplementary Figure 151.** Concentration-effect curves of 35 Au(III)-DTC complexes in a patient-derived Meso84 cell line in comparison with cisplatin and pemetrexed. .............. **S137**
**Supplementary Figure 152.** Concentration-effect curves of 35 Au(III)-DTC complexes in a patient-derived Meso92 cell line in comparison with cisplatin and pemetrexed. .............. **S138**
**Supplementary Figure 153.** Concentration-effect curves of 35 Au(III)-DTC complexes in a human Met5A non-malignant mesothelioma cell line in comparison with cisplatin and pemetrexed. ........................................................................................................... **S139**
**Supplementary Figure 154.** Concentration-effect curves of complexes **1G**, **5A**, **5B** and **5G** toward M0-differentiated THP-1 cells and murine AB12 MPM cells .............................. **S140**
**Supplementary Figure 155.** Body weight changes of Balb/C mice during a toxicity study. Mice were treated with **5A** (4 mg/kg), **5B** (4 mg/kg), **5C** (4 mg/kg), **5F** (2.5 mg/kg), **5G** (8 mg/kg), **5H** (4 mg/kg) in 4% DMSO/Cremophore EL in saline and respective vehicle. ........... ........................................................................................................................... **S140**
**Supplementary Figure 156.** Body weight changes of Balb/C mice (n=6) with subcutaneous AB12 MPM tumor allografts treated with **5A** (4 mg/kg), **5B** (4 mg/kg), **5C** (8 mg/kg), **5F** (2.5 mg/kg), **5G** (8 mg/kg), **5H** (4 mg/kg), cisplatin (1 mg/kg) + pemetrexed (5 mg/kg), and respective vehicle (4% DMSO/Cremophore EL in saline) on Days 19, 21, and 23 via i.p. route. ........................................................................................................................ **S141**

**Supplementary Figure 157.** Representative H&E-stained images of liver and kidney sections from mice treated with vehicle, pemetrexed + cisplatin and complexes **5A**, **5H** and **5F**. ........................................................................................................................ **S141**

**Supplementary Figure 158**. LogP standard curve used to determine the log p of the complexes. ........................................................................................................... **S142**

**Supplementary Figure 159.** ROS detection using live cell confocal microscopy imaging. ..... **S143**

**Supplementary Figure 160.** Western blot analysis of Binding Immunoglobulin Protein (BiP) and C/EBP Homologous Protein (CHOP) involved in ER stress in AB12 cells treated with **2G** for 4 h at indicated concentrations. ............................................................................... **S144**

**Supplementary Figure 161.** Body weight changes of Balb/C mice in a toxicity study, with injections starting on Day 1. The maximum soluble doses were administered in 4% DMSO/4% Cremophore EL in PBS: **1C** (1.5 mg/kg), **2G** (3.5 mg/kg), and vehicle (4% DMSO/4% Cremophore EL in saline). ............................................................................... **S144**

**Supplementary Figure 162.** Body weight changes of Balb/C mice (n=6) with subcutaneous AB12 MPM tumor allografts treated with **1C** (1.5 mg/kg), **2G** (3.5 mg/kg) and respective vehicle (4% DMSO/Cremophore EL in PBS) on Days 19, 21, and 23 via i.p. route. ....... **S145**

**Supplementary Figure 163**. Au accumulation in mouse organs and tumors obtained from Balb/C mice treated with **1C** (1.5 mg/kg) and **2G** (3.5 mg/kg) via i.p. route at the endpoint (Day 23) and quantified by ICP-MS. ..................................................................................... **S145**

**Supplementary Figure 164**. Dynamic light scattering (DLS) spectra of the vehicle, **1C** (0.16 mg/ml) and **2G** (0.39 mg/ml) in PBS with 4-5% DMSO/Cremophor EL...........................**S146**

**Supplementary Figure 165.** Representative H&E stained images of liver sections from all mice (n=6) treated with **1C**, **2G** and respective vehicle. ....................................................**S147**

**Supplementary Figure 166.** Representative H&E stained images of kidney sections from all mice (n=6) treated with **1C**, **2G** and respective vehicle. ................................................... **S148**

**Supplementary Figure 167.** Representative H&E stained images of tumor sections from all mice (n=6) treated with **1C**, **2G** and respective vehicle. .................................................. **S149**

**Supplementary Figure 168. (A)** Gating strategy for Fluorescence-Activated Cell Sorting (FACS). **(B)** Quantification of total percentage of macrophages in tumors freshly isolated from all mice (n=6) treated with **1C**, **2G** and respective vehicle. ........................................................... **S150**

**References** ..........................................................................................................**S151**

## Materials and methods

All chemicals used for synthesis were purchased from Tokyo Chemical Industry Co., Ltd (Tokyo, Japan) and BLD pharm (China). All solvents and chemicals were of analytical grade and used without further purification. The cyclometalated Au(III) precursors (scaffolds **1** - **5**) [1-4], as well as complexes **1A-1C**, **2A**, **2B**, **2H**, **3A**, **3B**, **5F** and **5H** were synthesized according to the previously described methods.[5-8] Ultrapure nitric acid (60%) for ICP-OES and ICP-MS was obtained from Kanto Kagaku Singapore Pte Ltd. (Singapore). Pt internal standard for ICP-OES and Au and Re standards for ICP-MS were obtained from CPI International (USA). Thiazolyl blue tetrazolium bromide (MTT) was purchased from Alfa Aesar, Dulbecco's Modified Eagle's Medium/High glucose, Roswell Park Memorial Institute (RMPI) 1640 medium, Fetal Bovine Serum (FBS), Phosphate Buffered Saline (PBS), Tris–acetate EDTA (TAE), Apoptosis Kit with Annexin V for Flow Cytometry (Catalog number: V13242), Penicillin-Streptomycin (5,000 U/mL) were purchased from Thermo Fisher Scientific. RealTime-Glo extracellular ATP assay kit (Catalog number: GA5010) was purchased from Promega (Madison, USA). Milli-Q-grade purified water was obtained from a Milli-Q UV purification system (Sartorius Stedim Biotech S.A., Aubagne Cedex, France). Cremophor® EL were purchased from Sigma-Aldrich (St. Louis, MO, USA). Clinical grade cisplatin (1 mg/mL) was purchased from Accord Healthcare, Inc. (Durham, NC), clinical grade 5-fluorouracil (5-FU, 50 mg/mL) was purchased from Teva Pharmaceutical (Petach Tikva, Israel), clinical grade paclitaxel (6 mg/mL) was purchased from AqVida (Germany) and oxaliplatin (5 mg/mL) was purchased from Medac GmbH (Hamburg, Germany).

## Instrumentation

$^{1}$H NMR spectra were recorded using Bruker AV500 NMR spectrometer locked to the deuterium resonance of the solvent. Chemical shifts (δ) and coupling constants (*J*) are given in parts per million (ppm) and hertz (Hz), respectively. The chemical shifts were calculated

relative to the solvent signals used as the internal standard: δH 2.50 ppm for DMSO-$d^6$ and δH 1.94 ppm for ACN-$d^3$. ESI mass spectra measurements of complexes were carried out on a Thermo Finnigan LCQ ESI-MS ion trap mass spectrometer. Analytical HPLC was carried out on Shimadzu Prominence UFLC System, equipped with a DGU-20A3 Degasser and two LC-AD Liquid Chromatography Pump, SPD-20A UV/Vis detector and a C18 reverse phase column as its stationary phase (4.6 mm x 150 mm). ICP-OES determination of Au content was determined by the Chemical, Molecular and Materials Analysis Center, NUS, using ICP-OES Optima 8000 spectrometer (Perkin Elmer, Watham, MA, USA). ICP-MS determination of Au content was conducted using Agilent 7700e ICP-MS system. The absorbance of thiazolyl blue tetrazolium bromide (MTT) was measured using the BioTek Synergy H1 microplate reader (Bio-Tek, Winoosky, VT, USA). Flow cytometry was performed using a Cytoflex flow cytometer (Beckman Coulter, Brea, CA, USA). A Thermo Scientific high-speed centrifuge (Thermo Fisher Scientific, Sorvall ST8R) was utilized for the separation and isolation of samples based on their density gradients. Histopathological analysis was performed using Leica DM2700 microscope, Panoramic 250 Flash III (3D Histech, Hungary), Olympus BX 36-Bright Field Automated microscope (Olympus, Japan) and NanoZoomer S60 slide scanner (Hamamatsu Photonics, Japan). Western Blotting analysis was performed using ChemiDoc Touch Imaging System (Bio-Rad, USA).

**General procedure for synthesis of cyclometalated Au(III) dithiocarbamate complexes**

1 eq. of Au(III) benzyl pyridine chloride was dissolved in MeOH and 1.2 eq. of sodium dithiocarbamate was added dropwise. The reaction mixture was left to stir in the dark at r. t. for 1 day or until majority of the solids dissolved. The solids were filtered off. 10 eq. of $NH_4PF_6$ was added into the filtrate and the solution was stirred for 1 h. Product that have precipitated was obtained via filtration and washed with water. If no solids were observed, product was

obtained by precipitation with MeOH and water. The compound is stored at -20 °C until further use.

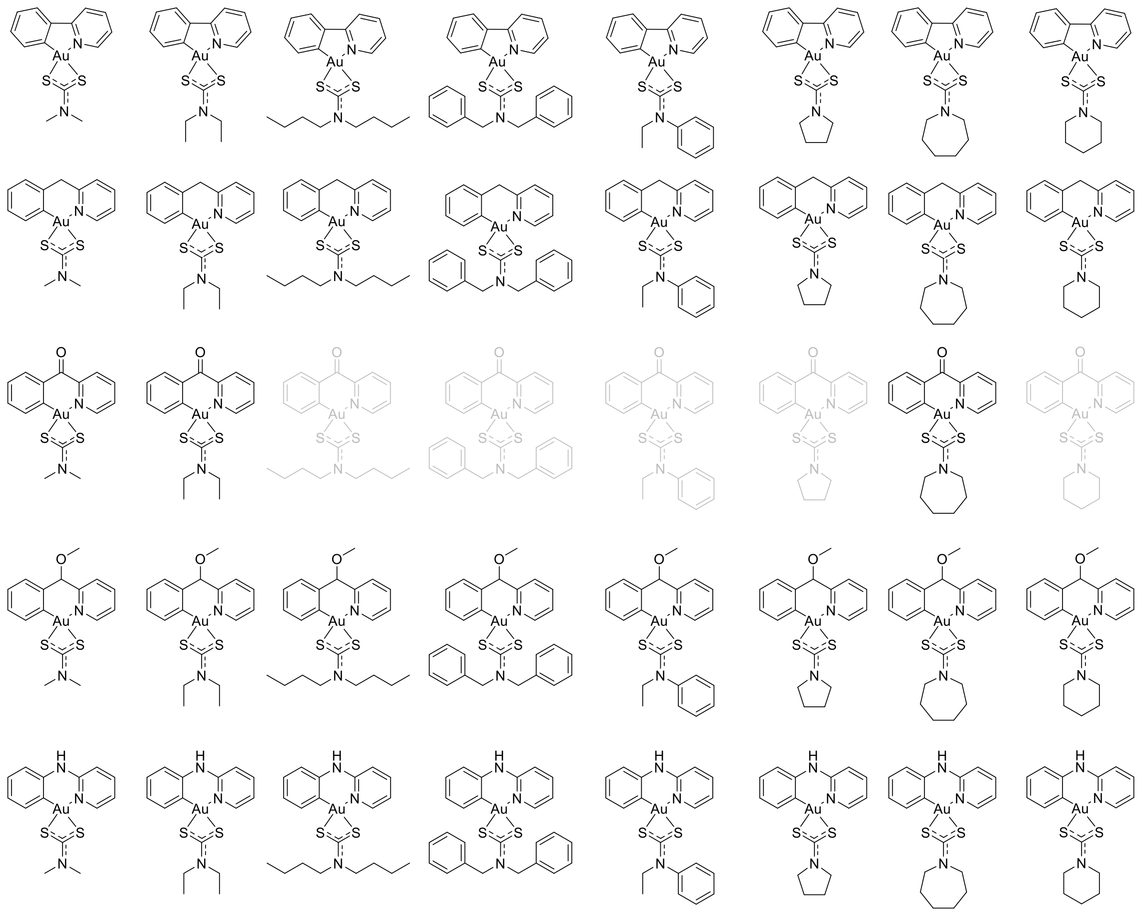


**1D:** $^{1}$H NMR (400 MHz, DMSO) δ 8.85 (ddd, *J* = 5.8, 1.4, 0.7 Hz, 1H), 8.48 (dt, *J* = 8.0, 1.2 Hz, 1H), 8.42 (dd, *J* = 7.4, 1.4 Hz, 1H), 8.11 (dd, *J* = 7.9, 1.4 Hz, 1H), 7.66 (ddd, *J* = 7.4, 5.8, 1.5 Hz, 1H), 7.53 (td, *J* = 7.5, 1.1 Hz, 1H), 7.56 – 7.31 (m, 11H), 7.17 (dd, *J* = 7.8, 1.0 Hz, 1H), 5.16 (d, *J* = 5.3 Hz, 4H). $^{13}$C NMR (126 MHz, DMSO) δ 197.86, 162.98, 150.99, 149.90, 143.78, 143.72, 132.85, 132.74, 132.20, 129.16, 129.08, 129.00, 128.76, 128.60, 128.19, 128.17, 128.13, 127.19, 126.06, 122.26, 56.65, 55.17. ESI-MS: m/z = 623.0886 $[M]^{+}$. RP-HPLC (% Purity): 99.9% at 254 nm; tr = 19.72 min.

**1E:** $^{1}$H NMR (400 MHz, DMSO) δ 9.06 – 8.68 (m, 1H), 8.61 – 8.29 (m, 2H), 8.09 (ddd, *J* = 9.5, 7.8, 1.5 Hz, 1H), 7.89 – 7.36 (m, 7H), 7.26 (qd, *J* = 7.6, 1.2 Hz, 1H), 7.00 (dd, *J* = 7.7, 1.0

Hz, 1H), 4.26 (q, $J$ = 7.3 Hz, 2H), 1.32 (q, $J$ = 7.5 Hz, 3H). $^{13}$C NMR (126 MHz, DMSO) δ 197.98, 197.80, 163.21, 163.04, 151.53, 150.86, 150.12, 144.00, 143.82, 143.76, 138.45, 137.52, 132.28, 132.20, 130.95, 130.63, 130.56, 130.39, 129.19, 129.16, 128.51, 128.24, 127.21, 127.15, 127.09, 126.82, 126.17, 126.01, 122.31, 52.13, 50.66, 12.16, 11.83. ESI-MS: m/z = 547.0573 $[M]^+$. RP-HPLC (% Purity): 99.9% at 254 nm; tr = 15.31 min.

**1F:** $^1$H NMR (400 MHz, DMSO) δ 8.81 (ddd, $J$ = 5.7, 1.4, 0.6 Hz, 1H), 8.53 – 8.47 (m, 1H), 8.46 – 8.39 (m, 1H), 8.12 (dd, $J$ = 7.8, 1.4 Hz, 1H), 7.68 (ddd, $J$ = 7.4, 5.8, 1.5 Hz, 1H), 7.54 (td, $J$ = 7.5, 1.1 Hz, 1H), 7.40 (td, $J$ = 7.6, 1.4 Hz, 1H), 7.16 (dd, $J$ = 7.7, 1.0 Hz, 1H), 4.15 – 3.70 (m, 4H), 2.26 – 1.98 (m, 4H). $^{13}$C NMR (126 MHz, DMSO) δ 187.91, 163.22, 151.15, 149.84, 143.98, 143.81, 132.32, 129.23, 128.23, 127.24, 126.21, 122.40, 52.49, 51.82, 24.14, 23.84. ESI-MS: m/z = 497.0416 $[M]^+$. RP-HPLC (% Purity): 99.9% at 254 nm; tr = 11.30 min.

**1G:** $^1$H NMR (400 MHz, DMSO) δ 8.84 (ddd, $J$ = 5.7, 1.4, 0.6 Hz, 1H), 8.61 – 8.47 (m, 1H), 8.43 (ddd, $J$ = 8.1, 7.4, 1.4 Hz, 1H), 8.13 (dd, $J$ = 7.8, 1.4 Hz, 1H), 7.69 (ddd, $J$ = 7.4, 5.8, 1.5 Hz, 1H), 7.54 (td, $J$ = 7.5, 1.1 Hz, 1H), 7.41 (td, $J$ = 7.5, 1.4 Hz, 1H), 7.19 (dd, $J$ = 7.7, 1.1 Hz, 1H), 4.01 (dd, $J$ = 5.8, 2.2 Hz, 4H), 1.94 (s, 4H), 1.59 (t, $J$ = 3.4 Hz, 4H). $^{13}$C NMR (126 MHz, DMSO) δ 193.11, 163.19, 151.26, 149.80, 143.97, 143.74, 132.20, 129.14, 128.14, 127.19, 126.10, 122.32, 53.62, 52.02, 26.27, 26.12, 26.08, 25.76. ESI-MS: m/z = 525.073 $[M]^+$. RP-HPLC (% Purity): 96.7% at 254 nm; tr = 14.64 min.

**1H:** $^1$H NMR (400 MHz, DMSO) δ 8.92 – 8.73 (m, 1H), 8.65 – 8.47 (m, 1H), 8.43 (td, $J$ = 7.8, 1.4 Hz, 1H), 8.13 (dd, $J$ = 7.8, 1.4 Hz, 1H), 7.70 (ddd, $J$ = 7.4, 5.8, 1.5 Hz, 1H), 7.54 (td, $J$ = 7.6, 1.1 Hz, 1H), 7.42 (td, $J$ = 7.6, 1.4 Hz, 1H), 7.18 (dd, $J$ = 7.7, 1.1 Hz, 1H), 3.97 (m, 4H), 1.78 (m, 6H). $^{13}$C NMR (126 MHz, DMSO) δ 190.65, 163.17, 151.26, 149.78, 143.94, 143.75, 132.22, 129.15, 128.13, 127.21, 126.14, 122.35, 52.32, 50.31, 25.53, 25.27, 23.28. ESI-MS: m/z = 511.0571 $[M]^+$. RP-HPLC (% Purity): 99.7% at 254 nm; tr = 13.38 min.

**2C:** $^{1}$H NMR (400 MHz, DMSO) δ 8.99 (dd, *J* = 5.9, 1.5 Hz, 1H), 8.32 (td, *J* = 7.7, 1.5 Hz, 1H), 8.09 (dd, *J* = 7.9, 1.5 Hz, 1H), 7.71 (ddd, *J* = 7.5, 5.8, 1.5 Hz, 1H), 7.38 (td, *J* = 7.7, 1.4 Hz, 2H), 7.29 (td, *J* = 7.3, 1.2 Hz, 1H), 7.16 (td, *J* = 7.5, 1.7 Hz, 1H), 4.55 (s, 2H), 3.81 (q, *J* = 7.8 Hz, 4H), 1.75 (ddd, *J* = 9.8, 5.1, 2.7 Hz, 4H), 1.55 – 1.24 (m, 4H), 0.94 (td, *J* = 7.4, 3.0 Hz, 6H).$^{13}$C NMR (126 MHz, DMSO) δ 194.32, 156.03, 151.92, 145.27, 143.57, 133.19, 130.58, 128.85, 128.58, 128.27, 126.95, 125.57, 52.74, 51.14, 45.94, 28.80, 28.50, 19.41, 19.37, 13.53. ESI-MS: m/z = 569.1355 $[M]^+$. RP-HPLC (% Purity): 96.5% at 254 nm; tr = 20.04 min.

**2D:** $^{1}$H NMR (400 MHz, DMSO) δ 8.99 (dd, *J* = 5.9, 1.4 Hz, 1H), 8.32 (d, *J* = 1.6 Hz, 1H), 8.09 (d, *J* = 1.5 Hz, 1H), 7.70 (s, 1H), 7.51 – 7.31 (m, 12H), 7.27 (dd, *J* = 7.4, 1.2 Hz, 1H), 7.15 (td, *J* = 7.5, 1.7 Hz, 1H), 5.13 (d, *J* = 10.3 Hz, 4H), 4.56 (s, 2H). $^{13}$C NMR (126 MHz, DMSO) δ 198.65, 156.20, 152.14, 145.11, 143.74, 133.35, 133.18, 133.10, 130.72, 129.22, 129.16, 129.05, 128.86, 128.79, 128.73, 128.46, 128.36, 128.32, 127.10, 125.69, 56.30, 54.83, 46.04.ESI-MS: m/z = 637.1042 $[M]^+$. RP-HPLC (% Purity): 96.9% at 254 nm; tr = 19.52 min.

**2E:** $^{1}$H NMR (400 MHz, DMSO) δ 9.11 – 8.75 (m, 1H), 8.46 – 8.19 (m, 1H), 8.08 (dd, *J* = 12.8, 7.8 Hz, 1H), 7.78 – 7.51 (m, 6H), 7.42 (ddd, *J* = 12.8, 7.6, 1.3 Hz, 1H), 7.38 – 7.27 (m, 1H), 7.24 (td, *J* = 7.3, 1.2 Hz, 1H), 7.21 – 7.15 (m, 1H), 7.07 (dd, *J* = 7.6, 1.6 Hz, 1H), 4.52 (d, *J* = 3.7 Hz, 2H), 4.24 (dd, *J* = 10.4, 7.2 Hz, 2H), 1.56 – 1.13 (m, 3H). $^{13}$C NMR (151 MHz, $CDCl_3$) δ 200.54, 200.10, 156.23, 151.50, 151.00, 146.34, 146.11, 143.63, 143.57, 138.54, 137.58, 132.09, 131.22, 131.06, 130.94, 130.78, 130.74, 129.37, 129.26, 129.02, 128.88, 127.77, 127.59, 126.97, 126.72, 126.30, 125.97, 52.27, 50.91, 47.64, 47.62, 12.69, 12.37, 0.13. ESI-MS: m/z = 561.0726 $[M]^+$. RP-HPLC (% Purity): 96.0% at 254 nm; tr = 15.19 min.

**2F**: $^{1}$H NMR (400 MHz, DMSO) δ 8.97 (ddd, *J* = 5.8, 1.5, 0.6 Hz, 1H), 8.32 (dd, *J* = 7.7, 1.5 Hz, 1H), 8.09 (ddd, *J* = 7.8, 1.5, 0.7 Hz, 1H), 7.73 (s, 1H), 7.39 (dd, *J* = 7.4, 1.6 Hz, 1H), 7.36 (dd, *J* = 7.7, 1.2 Hz, 1H), 7.32 – 7.27 (m, 1H), 7.18 (dd, *J* = 7.5, 1.6 Hz, 1H), 4.54 (s, 2H), 4.18

– 3.67 (m, 4H), 2.18 – 1.82 (m, 4H). $^{13}$C NMR (126 MHz, DMSO) δ 188.74, 155.97, 151.87, 145.25, 143.63, 133.12, 130.53, 128.94, 128.66, 128.35, 127.00, 125.65, 52.14, 51.51, 46.02, 24.13, 23.82. ESI-MS: m/z = 511.057 $[M]^+$. RP-HPLC (% Purity): 98.7% at 254 nm; tr = 11.42 min.

**2G:** $^1$H NMR (400 MHz, DMSO) δ 8.99 (ddd, *J* = 5.9, 1.5, 0.7 Hz, 1H), 8.32 (dd, *J* = 7.7, 1.5 Hz, 1H), 8.26 – 8.03 (m, 1H), 7.72 (ddd, *J* = 7.5, 5.8, 1.6 Hz, 1H), 7.39 (td, *J* = 7.5, 1.4 Hz, 2H), 7.29 (td, *J* = 7.3, 1.2 Hz, 1H), 7.18 (dd, *J* = 7.5, 1.7 Hz, 1H), 4.55 (s, 2H), 3.98 (dt, *J* = 7.5, 5.8 Hz, 4H), 1.91 (s, 4H), 1.58 (t, *J* = 3.4 Hz, 4H). $^{13}$C NMR (126 MHz, DMSO) δ 193.75, 156.06, 151.84, 145.37, 143.58, 133.21, 130.53, 128.86, 128.58, 128.26, 126.96, 125.57, 53.16, 51.59, 45.97, 26.21, 26.12, 26.09, 25.81. ESI-MS: m/z = 539.0884 $[M]^+$. RP-HPLC (% Purity): 99.9% at 254 nm; tr = 14.51 min.

**3G:** $^1$H NMR (400 MHz, DMSO) δ 9.17 (ddd, *J* = 5.7, 1.4, 0.6 Hz, 1H), 8.74 – 8.57 (m, 1H), 8.54 (ddd, *J* = 7.9, 1.7, 0.6 Hz, 1H), 8.09 (ddd, *J* = 7.5, 5.7, 1.7 Hz, 1H), 8.00 – 7.91 (m, 1H), 7.67 – 7.49 (m, 3H), 3.99 (t, *J* = 6.0 Hz, 4H), 1.91 (m, 4H), 1.57 (m, 4H). ). $^{13}$C NMR (126 MHz, DMSO) δ 192.40, 189.14, 152.39, 146.31, 144.31, 141.38, 135.09, 131.11, 130.88, 130.52, 129.31, 129.14, 127.91, 53.25, 51.85, 26.22, 26.12, 26.08, 25.81. ESI-MS: m/z = 553.0675 $[M]^+$. RP-HPLC (% Purity): 98.8% at 254 nm; tr = 12.67 min.

**4A:** $^1$H NMR (400 MHz, DMSO) δ 9.08 (d, *J* = 5.7 Hz, 1H), 8.43 (td, *J* = 7.7, 1.5 Hz, 1H), 8.20 (d, *J* = 7.7 Hz, 1H), 7.83 (t, *J* = 6.7 Hz, 1H), 7.54 (d, *J* = 7.3 Hz, 1H), 7.45 (dd, *J* = 7.5, 1.2 Hz, 1H), 7.36 (td, *J* = 7.3, 1.2 Hz, 1H), 7.28 (t, *J* = 7.4 Hz, 1H), 5.85 (s, 1H), 3.47 (d, *J* = 10.6 Hz, 6H). $^{13}$C NMR (126 MHz, DMSO) δ 193.44, 153.15, 152.77, 145.28, 144.11, 134.15, 131.18, 130.58, 129.58, 128.41, 127.65, 86.60, 56.28, 41.66, 40.39. ESI-MS: m/z = 515.0523 $[M]^+$. RP-HPLC (% Purity): 99.5% at 254 nm; tr = 8.59 min.

**4B:** $^1$H NMR (400 MHz, DMSO) δ 9.08 (d, *J* = 5.7 Hz, 1H), 8.43 (td, *J* = 7.7, 1.5 Hz, 1H), 8.20 (d, *J* = 7.7 Hz, 1H), 7.83 (t, *J* = 6.5 Hz, 1H), 7.53 (d, *J* = 7.3 Hz, 1H), 7.45 (dd, *J* = 7.6, 1.2 Hz, 1H), 7.36 (td, *J* = 7.3, 1.2 Hz, 1H), 7.28 (t, *J* = 7.5 Hz, 1H), 5.86 (s, 1H), 4.07 – 3.74 (m, 4H), 1.35 (dt, *J* = 10.3, 7.2 Hz, 6H). $^{13}$C NMR (126 MHz, DMSO) δ 193.47, 153.24, 152.70, 145.20, 144.10, 134.11, 131.13, 130.53, 129.59, 128.42, 127.63, 86.61, 56.36, 47.95, 46.45, 12.36, 12.04. ESI-MS: m/z = 543.0839 $[M]^+$. RP-HPLC (% Purity): 99.5% at 254 nm; tr = 12.00 min.

**4C:** $^1$H NMR (400 MHz, $CD_3CN$) δ 8.98 – 8.78 (m, 1H), 8.28 (td, *J* = 7.7, 1.5 Hz, 1H), 8.03 (ddd, *J* = 7.8, 1.6, 0.7 Hz, 1H), 7.68 (ddd, *J* = 7.5, 5.8, 1.5 Hz, 1H), 7.48 (ddd, *J* = 17.5, 7.5, 1.4 Hz, 2H), 7.35 (td, *J* = 7.4, 1.2 Hz, 1H), 7.27 (td, *J* = 7.5, 1.6 Hz, 1H), 5.63 (s, 1H), 4.12 – 3.59 (m, 4H), 3.45 – 3.23 (m, 3H), 1.85 – 1.68 (m, 4H), 1.42 (qd, *J* = 7.5, 5.9 Hz, 4H), 0.98 (td, *J* = 7.4, 4.0 Hz, 6H). $^{13}$C NMR (126 MHz, DMSO) δ 194.27, 152.70, 145.04, 144.01, 134.20, 131.18, 130.44, 128.39, 127.53, 86.48, 56.45, 52.84, 51.24, 28.78, 28.48, 19.44, 19.41, 13.52. ESI-MS: m/z = 599.1462 $[M]^+$. RP-HPLC (% Purity): 99.1% at 254 nm; tr = 19.61 min.

**4D:** $^1$H NMR (400 MHz, $CD_3CN$) δ 9.02 – 8.81 (m, 1H), 8.28 (td, *J* = 7.7, 1.5 Hz, 1H), 8.04 (ddd, *J* = 7.8, 1.6, 0.7 Hz, 1H), 7.68 (ddd, *J* = 7.5, 5.7, 1.5 Hz, 1H), 7.58 – 7.30 (m, 13H), 7.26 (td, *J* = 7.5, 1.6 Hz, 1H), 5.65 (s, 1H), 5.04 (s, 2H), 5.02 – 4.80 (s, 2H), 3.38 (s, 3H). $^{13}$C NMR (126 MHz, DMSO) δ 198.43, 152.79, 144.06, 134.06, 132.91, 132.80, 131.10, 130.54, 129.06, 129.00, 128.73, 128.71, 128.60, 128.51, 128.44, 128.30, 128.27, 128.22, 127.88, 127.70, 86.51, 56.25, 54.77. ESI-MS: m/z = 667.1148 $[M]^+$. RP-HPLC (% Purity): 97.1% at 254 nm; tr = 19.57 min.

**4E:** $^1$H NMR (400 MHz, DMSO) δ 9.05 (dd, *J* = 62.4, 5.7 Hz, 1H), 8.61 – 8.32 (m, 1H), 8.32 – 8.10 (m, 1H), 8.00 – 7.42 (m, 7H), 7.43 – 7.08 (m, 3H), 5.84 (s, 1H), 4.23 (ddt, *J* = 16.4, 9.5, 7.0 Hz, 2H), 3.23 (d, *J* = 47.1 Hz, 3H), 1.37 – 1.18 (m, 3H). $^{13}$C NMR (126 MHz, DMSO) δ

198.57, 198.31, 153.42, 152.86, 145.03, 144.12, 144.05, 138.18, 137.29, 134.01, 131.32, 131.00, 130.92, 130.60, 130.52, 130.33, 129.62, 128.47, 128.40, 127.66, 127.41, 127.15, 126.86, 86.68, 56.34, 51.80, 50.25, 12.16, 11.84. ESI-MS: m/z = 591.0832 $[M]^+$. RP-HPLC (% Purity): 98.5% at 254 nm; tr = 15.25 min.

**4F:** $^1$H NMR (400 MHz, $CD_3CN$) δ 8.99 – 8.87 (m, 1H), 8.28 (td, *J* = 7.7, 1.5 Hz, 1H), 8.03 (ddd, *J* = 7.8, 1.6, 0.7 Hz, 1H), 7.68 (ddd, *J* = 7.5, 5.7, 1.6 Hz, 1H), 7.48 (ddd, *J* = 17.5, 7.5, 1.4 Hz, 2H), 7.34 (td, *J* = 7.4, 1.2 Hz, 1H), 7.27 (dd, *J* = 7.5, 1.6 Hz, 1H), 5.63 (s, 1H), 3.94 – 3.78 (m, 4H), 3.34 (s, 3H), 2.13 – 2.03 (m, 4H). $^{13}$C NMR (126 MHz, DMSO) δ 188.60, 153.30, 152.75, 145.25, 144.11, 134.04, 131.16, 130.64, 129.63, 128.43, 127.70, 86.63, 56.28, 52.13, 51.49. ESI-MS: m/z = 541.068 $[M]^+$. RP-HPLC (% Purity): 98.8% at 254 nm; tr = 11.49 min.

**4G:** $^1$H NMR (400 MHz, DMSO) δ 9.09 (d, *J* = 5.7 Hz, 1H), 8.43 (td, *J* = 7.7, 1.5 Hz, 1H), 8.20 (d, *J* = 7.7 Hz, 1H), 7.83 (t, *J* = 6.5 Hz, 1H), 7.70 – 7.08 (m, 4H), 5.86 (s, 1H), 4.35 – 3.78 (m, 4H), 3.29 (s, 2H), 1.91 (t, *J* = 5.3 Hz, 4H), 1.59 (q, *J* = 3.7 Hz, 4H). $^{13}$C NMR (126 MHz, DMSO) δ 193.68, 153.41, 152.71, 145.43, 144.09, 134.15, 131.15, 130.57, 129.60, 128.41, 127.66, 86.67, 56.32, 53.16, 51.57, 26.32, 26.21, 26.13, 25.82. ESI-MS: m/z = 569.0988 $[M]^+$. RP-HPLC (% Purity): 99.9% at 254 nm; tr = 14.43 min.

**4H:** $^1$H NMR (400 MHz, DMSO) δ 9.08 (d, *J* = 5.7 Hz, 1H), 8.43 (td, *J* = 7.7, 1.5 Hz, 1H), 8.20 (d, *J* = 7.7 Hz, 1H), 7.83 (t, *J* = 6.6 Hz, 1H), 7.54 (d, *J* = 7.3 Hz, 1H), 7.45 (d, *J* = 7.5 Hz, 1H), 7.36 (td, *J* = 7.3, 1.2 Hz, 1H), 7.29 (d, *J* = 7.5 Hz, 1H), 5.86 (s, 1H), 4.20 – 3.72 (m, 4H), 1.77 (s, 6H). $^{13}$C NMR (126 MHz, DMSO) δ 191.20, 153.45, 152.72, 145.38, 144.10, 134.18, 131.22, 130.55, 129.62, 128.40, 127.67, 86.65, 56.29, 51.85, 49.81, 25.54, 25.28, 23.29. ESI-MS: m/z = 555.0836 $[M]^+$. RP-HPLC (% Purity): 99.3% at 254 nm; tr = 12.86 min.

**5A:** $^1$H NMR (400 MHz, DMSO) δ 10.97 (s, 1H), 8.37 (d, *J* = 5.9 Hz, 1H), 8.04 (s, 1H), 7.52 (d, *J* = 8.7 Hz, 1H), 7.48 – 7.41 (m, 1H), 7.35 (dd, *J* = 7.9, 1.5 Hz, 1H), 7.29 (dd, *J* = 7.9, 1.3

Hz, 1H), 7.17 – 6.99 (m, 2H), 3.51 (d, *J* = 15.9 Hz, 6H). $^{13}$C NMR (126 MHz, DMSO) δ 191.35, 147.33, 146.49, 141.02, 131.77, 130.22, 129.71, 123.67, 122.21, 118.07, 116.57, 116.32, 41.73, 40.73. ESI-MS: m/z = 486.0366 $[M]^+$. RP-HPLC (% Purity): 99.5% at 254 nm; tr = 9.00 min.

**5B:** $^{1}$H NMR (400 MHz, DMSO) δ 10.96 (s, 1H), 8.37 (dd, *J* = 6.4, 1.5 Hz, 1H), 8.04 (ddd, *J* = 8.6, 6.9, 1.6 Hz, 1H), 7.57 – 7.48 (m, 1H), 7.44 (ddd, *J* = 8.2, 7.0, 1.3 Hz, 1H), 7.35 (dd, *J* = 8.0, 1.5 Hz, 1H), 7.29 (dd, *J* = 7.9, 1.3 Hz, 1H), 7.15 – 6.96 (m, 2H), 3.90 (t, *J* = 7.2 Hz, 4H), 1.35 (dt, *J* = 12.2, 7.2 Hz, 6H). $^{13}$C NMR (126 MHz, DMSO) δ 191.27, 147.31, 146.47, 141.03, 131.74, 130.27, 129.70, 123.70, 122.35, 118.03, 116.58, 116.28, 48.11, 46.92, 12.37, 12.05. ESI-MS: m/z = 514.0679 $[M]^+$. RP-HPLC (% Purity): 99.3% at 254 nm; tr = 12.81 min.

**5C:** $^{1}$H NMR (400 MHz, CD3CN) δ 9.04 (s, 1H), 8.27 – 8.11 (m, 1H), 7.90 (ddd, J = 8.6, 6.9, 1.6 Hz, 1H), 7.48 – 7.29 (m, 2H), 7.24 (dd, J = 7.9, 1.4 Hz, 2H), 7.07 – 6.96 (m, 1H), 6.96 – 6.85 (m, 1H), 3.86 – 3.58 (m, 4H), 1.86 – 1.69 (m, 4H), 1.47 – 1.29 (m, 4H), 0.99 (td, J = 7.4, 5.4 Hz, 6H). $^{13}$C NMR (126 MHz, DMSO) δ 192.22, 147.45, 146.64, 140.94, 131.90, 130.39, 129.64, 123.72, 122.56, 118.06, 116.52, 116.29, 52.92, 51.62, 28.76, 28.47, 19.42, 19.39, 13.54. ESI-MS: m/z = 570.1306 $[M]^+$. RP-HPLC (% Purity): 99.6% at 254 nm; tr = 20.37 min.

**5D:** $^{1}$H NMR (400 MHz, DMSO) δ 11.00 (s, 1H), 8.38 (ddd, *J* = 6.4, 1.5, 0.6 Hz, 1H), 8.03 (ddd, *J* = 8.6, 6.9, 1.6 Hz, 1H), 7.56 – 7.50 (m, 1H), 7.50 – 7.35 (m, 12H), 7.26 (dd, *J* = 7.9, 1.2 Hz, 1H), 7.11 – 7.01 (m, 2H), 5.16 (s, 4H). $^{13}$C NMR (101 MHz, DMSO) δ 196.45, 147.66, 146.69, 141.07, 132.87, 132.79, 131.89, 130.51, 129.70, 129.05, 128.99, 128.68, 128.56, 128.15, 128.11, 123.84, 122.33, 117.98, 116.62, 116.16, 56.22, 55.04. ESI-MS: m/z = 638.0995 $[M]^+$. RP-HPLC (% Purity): 98.1% at 254 nm; tr = 20.03 min.

**5E:** $^{1}$H NMR (400 MHz, DMSO) δ 10.95 (d, *J* = 2.9 Hz, 1H), 8.33 (ddd, *J* = 84.0, 6.5, 1.5 Hz, 1H), 8.02 (dddd, *J* = 24.6, 8.6, 6.9, 1.6 Hz, 1H), 7.75 – 7.23 (m, 8H), 7.16 – 6.85 (m, 3H), 4.27 (d, *J* = 7.2 Hz, 2H), 1.65 – 1.03 (m, 3H). $^{13}$C NMR (126 MHz, DMSO) δ 196.50, 196.25,

147.81, 147.74, 146.74, 146.56, 141.14, 141.07, 138.36, 137.66, 131.96, 131.81, 130.92, 130.71, 130.63, 130.53, 130.39, 130.27, 129.75, 129.68, 127.05, 126.82, 123.84, 123.79, 122.67, 122.32, 118.03, 117.95, 116.68, 116.61, 116.26, 116.15, 51.93, 50.73, 12.14, 11.84. ESI-MS: m/z = 562.0679 $[M]^+$. RP-HPLC (% Purity): 98.9% at 254 nm; tr = 15.41 min.

**5G:** $^1$H NMR (400 MHz, DMSO) δ 10.97 (s, 1H), 8.37 (dt, *J* = 6.5, 0.9 Hz, 1H), 8.04 (ddd, *J* = 8.6, 6.9, 1.6 Hz, 1H), 7.52 (ddd, *J* = 8.7, 1.4, 0.7 Hz, 1H), 7.44 (ddd, *J* = 8.2, 7.0, 1.3 Hz, 1H), 7.32 (ddd, *J* = 22.0, 7.9, 1.4 Hz, 2H), 7.12 – 6.99 (m, 2H), 4.26 – 3.76 (m, 4H), 1.92 (s, 4H), 1.58 (p, *J* = 2.6 Hz, 4H). $^{13}$C NMR (126 MHz, DMSO) δ 191.63, 147.45, 146.65, 141.07, 131.91, 130.39, 129.68, 123.74, 122.65, 117.98, 116.59, 116.23, 53.35, 52.05, 26.25, 26.11, 26.05, 25.78. ESI-MS: m/z = 540.0836 $[M]^+$. RP-HPLC (% Purity): 99.4% at 254 nm; tr = 14.91 min.

**X-ray crystallography**

Single crystal of the complex **1H** was prepared by layering $CDCl_3$ solutions with diethyl ether in an NMR tube. Crystals were measured at low temperature (T = 100 K) on a four circles goniometer using monochromatized Mo X-ray radiation (λ = 0.71073 Å). Frames were integrated with the Bruker SAINT software package using a narrow-frame algorithm. Data were corrected for absorption effects using the multi-scan method implanted in the software (SADABS). Structure was solved using direct methods and subsequent differences Fourier maps, and then refined by least squares procedures on weighted $F^2$ values using the SHELXL-version 2014/6 included in WinGX system programs for Windows**.** All non-hydrogen atoms were assigned anisotropic displacement parameters. Hydrogen atoms were located on difference Fourier maps then introduced as fixed contributors in idealized geometry with an isotropic thermal parameters fixed at 20 % higher than those carbons atoms they were attached.

**RP-HPLC analysis**

Determination of the purity of cyclometalated Au(III) complexes was performed using analytical HPLC on a Shimadzu Prominence System equipped with a DGU-20A3 Degasser, two LC-20AD Liquid Chromatography Pump, a SPD-20A UV/Vis Detector and a Shim Pack GVP-ODS 2.0 mm 18 column (5 μM, 120Å, 250 mm × 4.60 mm i.d.). The analysis was conducted at r.t. with a flow rate of 1.0 mL/min and detection at 254 and 280 nm. Mobile phase A was $H_2O$ + 0.1% Trifluoroacetic acid (TFA) while mobile phase B is ACN + 0.1% TFA. The gradient program parameters were as follows: mobile phase B was increased from 30 to 95% over 30 min. 95% mobile phase B was maintained for 10 min, 95 to 30% over 5 min, and finally returned to 30% for 10 min for the next analysis. LogP values of the complexes were calculated based on the HPLC retention times derived from the calibration standard curve. The calibration standard curve was established using 2-butanone, phenylacetonitrile, anisole, ethyl benzoate, benzophenone, diphenyl ether and triphenylamine. To conduct an aqueous stability study, the HPLC profiles of the sample stock solutions were diluted to a 1:1 ratio of DMSO to water. These profiles were subsequently monitored over a 72 h period, with readings taken at 24 h intervals.

**Dynamic light scattering (DLS) experiments**

The particle size distribution was obtained from DLS experiments conducted on a Malvern Zetasizer Ultra (United Kingdom) using polystyrene cuvettes (Sarstedt, Germany) with a 1 cm optical path length at 25 °C and a 12.78° Scattering Collection Angle. Spectra were obtained in PBS for the vehicle (5.0% DMSO/Cremophor EL), **1C** (0.16 mg/ml, 4.0% DMSO/Cremophor EL) and **2G** (0.39 mg/ml, 4.9% DMSO/Cremophor EL) under the same experimental conditions as for the corresponding in vivo experiment. Data was analysed using ZS Xplorer software (version 3.00) and illustrated with GraphPad Prism software (version 9.2).

## Cell lines and culture conditions

Human malignant mesothelioma cancer cell lines JU77 and LO68 were obtained from Cell Bank Australia (Children's Medical Research Institute, Westmead, Australia). Mouse malignant mesothelioma cancer cell line AB12, human nonmalignant mesothelioma cancer cell line Met5A (mesothelial cells), mouse macrophage cell line J774 and human monocytic cell line THP-1 were obtained from American Type Culture Collection (ATCC) (Rockville, MD, USA). Human malignant mesothelioma cell lines VMC23 (epithelial), Meso82 (sarcomatous), Meso92 (biphasic), were kindly provided by Prof. Walter Berger (Medical University of Vienna). All cells except for J774 were cultured in RPMI and J774 cells were cultured in DMEM. Both types of media were supplemented with 10% FBS and 1% penicillin-streptomycin (10,000 U/mL). Cells were grown in tissue culture flasks (75 $cm^2$ and 25 $cm^2$, SPL Life Sciences Co., Ltd., Korea). All cell lines were grown at 37 °C in a humidified atmosphere of 95% air and 5% $CO_2$ and passaged when they reached 80–90% confluency. All Au stock solutions were prepared in 1% DMSO in the respected medium, ensuring that cell viability was not inhibited. The Au concentration in the stock solutions was confirmed through inductively coupled plasma optical emission spectrometry. The stock solution of pemetrexed (10 mM) was prepared in Milli-Q water.

## Inhibition of cell viability assay

The cytotoxicity of compounds was determined using an MTT colorimetric test. The cells were harvested from culture flasks by trypsinization and seeded into Cellstar 96-well microculture plates (Greiner Bio-One) at the seeding density of 6000 cells per well ($6 \times 10^4$ cells/mL). After the cells were allowed to resume exponential growth for 16 h, they were exposed to drugs at different concentrations in media for 72 h. The drugs were diluted in a complete medium at the desired concentration added to each well (100 μL) and serially diluted in other wells. After exposure for 72 h, the media was replaced with MTT in media (5 mg/mL, 100 μL/well) and

incubated for an additional 50 min. Subsequently, the medium was aspirated, and the purple formazan crystals formed in viable cells were dissolved in DMSO (100 μL/well). Optical densities were measured at 570 nm using a BioTek Synergy H1 microplate and Multiskan™ SkyHigh plate readers. The quantity of viable cells was expressed in terms of treated/control (T/C) values in comparison to untreated control cells, and 50% inhibitory concentrations ($IC_{50}$) were calculated from concentration-effect curves by interpolation. An evaluation was based on means from at least three independent experiments, each comprising three replicates per concentration level.

For macrophage analyses, the acute monocytic leukemia-derived THP-1 cell line (ATCC) was used as a model. $1 \times 10^4$ THP-1 cells were seeded per well in 96-well plates in RPMI-1640 with 10% FCS and treated for 24 h with 75 nM phorbol 12-myristate 13-acetate (PMA, Sigma-Aldrich) to induce M0 macrophage differentiation. After the differentiation period, PMA was replaced by fresh culture medium to recover for 2h. Afterwards increasing concentrations of **1G**, **5A**, **5B** and **5G** were added for a 72-h continuous drug exposure assay. In parallel, the murine mesothelioma cell line AB12 was seeded into 96-well plates at $2.5 \times 10^3$ cells/well and were allowed to attach for 24 h. Gold compounds were added to the cells as described for the THP-1 model above. After the incubation period of 72 h, cell viability was determined by an MTT-based assay (EU4U, Biomedica) following the instructions of the manufacturer. DMSO had not impact on the cell viability at the concentrations used.

**Flow cytometry experiments**

***Phagocytosis assay***

AB12 cells (70-80% confluence in a T75 flask) were harvested by trypsinization, neutralized with RPMI, centrifuged (300 × *g*, 5 min) and labelled with CellTracker Red CMTPX Dye (0.72 μM for $5 \times 10^5$ cells) at 37°C for 30 minutes in 1 ml of serum-free RPMI. Following

centrifugation of the cells (300 × *g*, 5 min), 5 ml of fresh RPMI was introduced, and the cells were seeded at a density of 5 x $10^5$ cells per well into Cellstar 6-well plates (Greiner Bio-One) (1 ml per well). J774 macrophages (70-80% confluence in a T75 flask) were detached by scraping, centrifuged (300 × *g*, 5 min). The medium was substituted with 1 ml of DMEM containing CellTracker Green CMFDA (1 μM) and the J774 macrophages were stained at 37°C for 30 minutes. Following centrifugation of the J774 macrophages (300 × *g*, 5 min), 5 ml of fresh DMEM was introduced, and the macrophages were seeded into a 100 $cm^2$ petri dish (SPL Life Sciences, Korea). After 24 h, the media in AB12 cells was replaced with 1 ml of fresh RPMI containing compounds of interest at equipotent concentration of 5 × $IC_{50}$ and incubated for 100 minutes. After removing the drug-containing media, the cells were trypsinized (0.5 ml), neutralized with 1 ml of RPMI, centrifuged (300 × *g*, 5 min) and counted. From each sample, 2 × $10^5$ AB12 cells were seeded into Cellstar 6-well plates (Greiner Bio-One) (1 ml per well). 2 × $10^5$ J774 macrophages were detached from a petri dish by scraping and added to AB12 cells into 6-well plates. AB12 cells and J774 macrophages were co-incubated for 16 h at 37°C and 5% $CO_2$, detached by scraping, centrifuged (300 × *g*, 5 min), and the pellets were resuspended in PBS for immediate analysis using a CytoFLEX Flow Cytometer (Beckman Coulter, USA). The percentage of phagocytosis was determined by calculating the number of double-positive macrophages (Q2) over the total number of macrophages (Q2 + Q3). Dot plots were obtained from 10000 events, and FlowJo software (ver. 10.9.0) was utilized for the analysis of the flow cytometry data.

***Annexin V/PI assay***

AB12 cells were seeded into Cellstar 6-well plates (Greiner Bio-One) at a density of 5 × $10^5$ cells/well (1 ml per well). The cells were allowed to resume exponential growth for 16 h. The medium was removed and cells were washed with PBS (2 × 1 mL). Afterwards the cells were incubated with a fresh medium containing compounds of interest at their respective 5 × $IC_{50}$

concentrations for 100 min at 37 °C. After the treatment, the supernatant was collected in 2 mL microtubes, centrifuged (300 × *g*, 5 min) and the media was removed. The adherent cells were then trypsinized with 500 µL of trypsin at 37 °C for 5 min. Next, 1 mL of medium was added to neutralize trypsin and the resulting cell suspension was combined with the supernatant. The wells were washed with 200 µL of PBS, which was also combined with the supernatant. The combined solutions were centrifuged (300 × *g*, 5 min), and the resulting pellets were washed with PBS (2 × 1 mL) and resuspended in 200 µL of Annexin V binding buffer. The cells were stained with Annexin V-FITC for 15 min in ice protected from light, and then propidium iodide (PI) was added just before each measurement. Cell death was evaluated by flow cytometry using a CytoFLEX Flow Cytometer equipped with a 488 nm laser and a 638 nm laser (Beckman Coulter, Brea, CA, USA). The cells were excited by the 488 nm laser, and fluorescence emissions were detected at 525/40 nm for FITC (Annexin V) and at 610/20 nm for ECD (PI). The resulting dot blots were acquired from 10,000 events and quantified using Flowjo software (version 10.8.0, BD Biosciences, San Jose, CA, USA). The percentage of necrosis was determined by calculating the sum of cells in Q1 and Q2.

***Calreticulin (CRT) detection***

AB12 cells were seeded into Cellstar 6-well plates (Greiner Bio-One) at a density of $5 \times 10^5$ cells/well (2 ml per well). The cells were allowed to resume exponential growth for 16 h. Afterwards the cells were incubated with a fresh medium containing compounds of interest at their respective 5 × $IC_{50}$ concentrations for 2 h at 37 °C. After the treatment, the supernatant was collected in 2 mL microtubes. The wells were washed with 1 ml of PBS, trypsinized with 300 µL of trypsin at 37 °C for 5 min and combined with the corresponding supernatant. The cell suspension was then centrifuged (300 × *g*, 5 min), washed with PBS (2 × 1 ml), stained with propidium iodide (PI) for 10 min at RT, washed with 1 ml of PBS and centrifuged again (300 × *g*, 5 min). Next, cells were incubated with CRT antibody conjugated with AlexaFluor

488 (62304S, Cell Signaling Technology) at 4 °C overnight, washed twice with PBS, reconstituted in 500 μL of PBS and analyzed by flow cytometry using a CytoFLEX Flow Cytometer equipped with a 488 nm laser and a 638 nm laser (Beckman Coulter, Brea, CA, USA). The cells were excited by the 488 nm laser, and fluorescence emissions were detected at 525/40 nm for FITC (CRT) and at 610/20 nm for ECD (PI). The resulting dot blots were acquired from 10,000 events and quantified using Flowjo software (version 10.8.0, BD Biosciences, San Jose, CA, USA).

***High Mobility Group Box 1 (HMGB1) detection***

AB12 cells were seeded into Cellstar 6-well plates (Greiner Bio-One) at a density of $5 \times 10^5$ cells per well (2 ml per well). The cells were allowed to resume exponential growth for 16 h. Subsequently, were incubated with a fresh medium containing compounds of interest at their respective $5 \times IC_{50}$ concentrations for 4 h at 37 °C. After the treatment, the supernatant was collected in 2 mL microtubes. The wells were washed with 1 ml of PBS, trypsinized with 300 μL of trypsin at 37 °C for 5 min and combined with the corresponding supernatant. The cell suspension was then centrifuged (300 × *g*, 10 min), washed with PBS (2 × 1 ml). Cells were fixed with 4% paraformaldehyde for 20 min at RT, washed once with 1 mL PBS, centrifuged (500 × *g*, 10 min), permeabilized by 0.3% Triton X-100 (ST795, Beyotime) for 10 minutes at RT, washed once with 1 mL PBS and centrifuged (500 × *g*, 10 min). Next, cells were incubated with HMGB1 antibody (Catalogue number: 3935, Cell Signaling Technology) at 4 °C overnight (1:500 dilution in PBS) and washed with 1 ml of PBS (500 × *g*, 10 min). Next, cells were incubated with secondary antibody conjugated with AlexaFluor 488 (Catalogue number: ab150081, Abcam) for 1 h (1:1000 dilution in PBS), washed with 1 ml of PBS (500 × *g*, 10 min), centrifuged (500 × *g*, 10 min), reconstituted in 500 μL of PBS and analyzed by flow cytometry using a CytoFLEX Flow Cytometer equipped with a 488 nm laser (Beckman Coulter, Brea, CA, USA). The cells were excited by the 488 nm laser, and fluorescence

emission was detected at 525/40 nm for FITC (CRT). The resulting dot blots were acquired from 10,000 events and quantified using Flowjo software (version 10.8.0, BD Biosciences, San Jose, CA, USA).

***Fluorescence-Activated Cell Sorting (FACS) of macrophages***

Tumors were finely minced and placed in a digestion buffer comprising 0.1 mg/mL DNase I and 2.0 mg/mL collagenase type IV in PBS. Following a 30-minute incubation at 37°C, the samples were filtered through a 70 µm Cell Strainer, centrifuged at 300 × *g* for 5 minutes, washed with RPMI-1640 media supplemented with 10% FBS and 1% P/S, and centrifuged once more. Lysis of red cells was achieved by adding 1 mL of Lysing Buffer (Red Blood Cell Lysing Buffer Hybri-Max™, Sigma-Aldrich) to the cell pellet, gently mixing for 1 minute. The buffer was then diluted with 15 mL of PBS, followed by centrifugation at 300 × *g* for 7 minutes and decanting of the supernatant. For flow cytometry analysis, $2 \times 10^6$ cells were resuspended in 100 µL of FACS buffer (PBS, 5% FBS). Fc receptors were blocked by pre-incubating cells with 0.25 µg (0.5 µL) of TruStain FcX™ PLUS (anti-mouse CD16/32, BioLegend) antibody in a 100 µL volume for 10 min on ice. Subsequently, 0.25 µg (1 µL) of Brilliant Violet 421™ anti-mouse/human CD11b (BioLegend) and PerCP/Cyanine5.5 anti-mouse CD64 (FcγRI) antibody (BioLegend) were combined, added to each sample, and incubated on ice for 30 minutes in the dark. After centrifugation at 300 × *g* for 5 min, cells were resuspended in 200 µL of FACS buffer. Propidium iodide (0.025 µg per sample) was added just before each measurement. Flow cytometry was conducted using a CytoFLEX Flow Cytometer (Beckman Coulter, USA), acquiring dot blots from 10,000 events. FlowJo software (ver. 10.9.0) was utilized for the analysis of the flow cytometry data.

**Confocal microscopy experiments**

***CRT detection***

AB12 cells were seeded into confocal dishes (Biosharp) at a density of 3 × $10^5$ cells/dish (1 mL per dish). The cells were allowed to resume exponential growth for 16 h. Afterwards, the cells were incubated with a fresh medium containing compounds at their respective 5 × $IC_{50}$ concentrations for 2 h at 37°C. After the treatment, the dishes were washed with 1 ml of PBS, fixed in 4% paraformaldehyde in PBS at r. t. for 20 min and washed twice with 1 mL of PBS. The dishes were then incubated with CRT antibody conjugated with Alexa Fluor 488 (1:200 in PBS, 62304S, Cell Signaling Technology) at 4°C overnight and washed twice with PBS. Then the dishes were incubated with Wheat Germ Agglutinin conjugated with Alexa Fluor 633 dye (W21404, Invitrogen) at r. t. in darkness for 30 min, washed with 1 mL of PBS, stained with propidium iodide (PI) for 10 min at r. t. in darkness and washed with 1 mL of PBS. Samples were stored in 1 mL of PBS. The confocal images were acquired by Laser Confocal Scanning Microscope (Leica SPE).

***HMGB1 detection***

AB12 cells were seeded into confocal dishes (Biosharp) at a density of 3 × $10^5$ cells/dish (1 mL per dish). The cells were allowed to resume exponential growth for 16 h. Afterwards, the cells were incubated with a fresh medium containing compounds at their respective 5 × $IC_{50}$ concentrations for 2 h at 37°C. After the treatment, the dishes were washed with 1 ml of PBS, fixed in 4% paraformaldehyde in PBS at r. t. for 20 min and washed twice with 1 mL of PBS. Followed by penetration with 0.3% Triton X-100（ST795, Beyotime) for 10 min at r. t., the dishes were washed twice with 1 mL of PBS. The dishes were then incubated with HMGB1 antibody (1:200 dilution in PBS, 3935, Cell Signaling Technology) at 4° C overnight and washed twice with PBS. Then they were incubated with a secondary antibody conjugated with AlexaFluor 488 (ab150081, Abcam) (1:1000 dilution in PBS) at r. t. in darkness for 2 h, washed with 1 mL of PBS, stained with propidium iodide (PI) for 10 mins at r. t. in darkness and

washed with 1 mL of PBS. Samples were stored in 1 mL of PBS. The confocal images were acquired by Laser Confocal Scanning Microscope (Leica SPE).

**ROS detection**

AB12 cells were seeded into the confocal dish (Biosharp) at a density of $3 \times 10^5$ cells/well (1 mL per well). The cells were allowed to resume exponential growth for 16 h. The cell culture medium was aspirated and washed with PBS (2 × 1 ml). In a dimly lit setting, a solution of $H_2$DCFDA (2',7'-dichlorodihydrofluorescein diacetate) in 1× HBSS (10 µM, 1 mL) was added to each well and allowed to incubate for 15 min at 37 °C. Following this, the $H_2$DCFDA solution was aspirated and the wells were rinsed with HBSS (2 × 1 ml). Subsequently, the cells were treated with fresh colourless cell culture medium containing compounds of interest at their respective 5× $IC_{50}$ concentrations for 4 h at 37 °C. Following the incubation period, the drug-containing medium was removed and the wells were washed with PBS (2 × 1 ml).

Subsequently, the cells were treated with a culture medium containing 1 µM of Mitotracker TM Deep Red, 1 µM ER-Tracker™ Blue-White DPX and 1 µg/mL of Hoechst 33342 for 30 minutes. Following this incubation, the cells were washed with PBS (2 × 1 ml). The samples were then protected from light with aluminum foil before imaging. Images were captured using Laser Confocal Scanning Microscope (Leica SPE) and analyzed using the Microscope Software Platform LAS X Life Science.

**Western Blotting experiments**

For Western Blot detection of BiP and CHOP proteins, AB12 cells were seeded into Cellstar 6-well plates (Greiner Bio-One) at a density of $1 \times 10^6$ cells/well (2 ml per well). The cells were allowed to resume exponential growth for 16 h. Afterwards, the cells were incubated with a fresh medium containing compounds of interest at their respective 5 × $IC_{50}$ concentrations for 4 h at 37 °C. After the treatment, the wells were washed with ice-cold PBS (2 × 1 ml). The

cells were lysed with 200 μL of RIPA buffer, scraped, collected into 2 mL microtubes, sonicated for 45 sec on ice and centrifuged at 12000 × *g* for 20 min at 4 °C. Protein concentration was measured by BCA Protein Assay Kit (PierceTM). 30 μg protein were reconstituted in loading buffer (5× Laemmli Buffer with 5% DTT) and heated at 95 °C for 10 min. Subsequently, the protein mixtures were resolved on 10% SDS-PAGE gels (BeyoGel$^{TM}$) by electrophoresis (80 V for 20 min followed by 100 V for 60 min) and transferred onto a nitrocellulose membrane (250 mA for 2 h).

For Western Blot detection of extracellular HMGB1 protein, AB12 cells were seeded into Cellstar 6-well plates (Greiner Bio-One) at a density of $1 \times 10^6$ cells/well (2 ml per well). The cells were allowed to resume exponential growth for 16 h. Afterwards, the cells were incubated with a fresh medium containing compounds of interest at their respective 5 × $IC_{50}$ concentrations for 4 h at 37 °C. After the treatment, the supernatants were collected in 2 mL microtubes, centrifuged at 12000 × *g* for 20 min at 4 °C. Equal volumes of supernatants (22.5 μL) were reconstituted in loading buffer (5× Laemmli Buffer with 5% DTT) and heated at 95 °C for 10 min. Subsequently, the protein mixtures were resolved on 10% SDS-PAGE gels (BeyoGel$^{TM}$) by electrophoresis (80 V for 20 min followed by 100 V for 60 min) and transferred onto a nitrocellulose membrane (250 mA for 2 h).

For both experiments, the protein bands were visualised with Ponceau S stain solution and the nitrocellulose membranes were cut into strips based on the protein ladder. The membranes were washed with a TBS-T wash buffer (0.1% Tween-20 in 1× PBS) 3 times for 5 min. Subsequently, they were blocked in 5% BSA (w/v) in wash buffer for 1 h and incubated with the HMGB1 primary antibodies (Catalogue Number: 3935S, Cell Signaling Technology) or Bip primary antibodies (Catalogue Number: 3177T, Cell Signaling Technology) or CHOP primary antibodies (Catalogue Number: 2895T, Cell Signaling Technology) in 5% BSA (w/v) in wash

buffer at 4 °C overnight. The membranes were washed with a wash buffer 3 times for 10 min. After incubation with HRP-conjugated secondary antibodies (Catalogue Number: 7074P2, Cell Signaling Technology) in 2% BSA (w/v) in wash buffer at RT for 2 h, the membranes were washed with wash buffer 3 times for 5-10 min. Immune complexes were detected with SuperSignal™ West Atto Ultimate Sensitivity Substrate (A38554, Thermo Scientific) and analyzed using enhanced chemiluminescence imaging (Biorad). β-Actin was used as a loading control. Primary antibody was used at 1:1000 dilution and secondary antibody was 1:5000 dilution.

**RealTime-Glo extracellular ATP assay**

AB12 cells were seeded into Cellstar 96-well plates (Greiner Bio-One) at a density of $1.8 \times 10^4$ cells/well (1 ml per well). The cells were allowed to resume exponential growth for 16 h. Afterwards the medium was replaced with fresh medium containing compounds of interest at their respective $5 \times IC_{50}$ concentrations. ATP reagent was added according to the manufacturer's protocol (RealTime-Glo Extracellular ATP Assay Kit (Catalogue number: GA5010)). Luminescence was measured using Molecular Devices SpectraMax iD5 plate reader. Measurements were taken at 2-minute intervals to capture real-time changes in extracellular ATP release. The luminescence data was recorded for a total duration of 6 h following the treatment. Kinetic graphs were generated to visualize the time-dependent changes in ATP release. An evaluation was based on means from at least three independent experiments.

**Animal experiments**

All animal procedures were performed under the Guidelines for Care and Use of Laboratory Animals of the City University of Hong Kong and approved by the Animal Ethics Committee of the City University of Hong Kong. All mice were maintained in the laboratory animal

research unit (LARU) of the City University of Hong Kong in specific pathogen-free conditions. All mice experiments were performed in compliance with the guidelines and protocols approved by the institutional and local ethics committee of HKSAR, Department of Health. Six- to eight-week old BALB/c mice were purchased from the Laboratory Animal Research Unit (LARU) of the City University of Hong Kong and the Laboratory Animal Research Unit (LARU) of Chinese University of Hong Kong. Animals were randomly assigned to different groups. Prior to assignment to groups, the weight variation of the animals did not exceed 20% of the mean weight. Animals were grouped and housed in solid bottom polycarbonate cages (5 mice per cage) and provided with pelleted food and water ad libitum. Environmental controls for the animal room were set to maintain 22−27 °C, a relative humidity of 55−75%, a minimum of 10 air changes/h, and a 12 h light/12 h dark cycle. No known contaminants were present in the diet or water at levels that might interfere with this study. The toxicity study was performed at maximum soluble dose in 4% DMSO/4% Cremophore EL in saline (or PBS for **1C** and **2G**), namely **5G** (8 mg/kg), **5A**, **5B**, **5C** and **5H** (4 mg/kg), **2G** (3.5 mg/kg), **5F** (2.5 mg/kg), **1C** (1.5 mg/kg). **5A**, **5H** and **5F**, were administered daily (12 injections in total), while the remaining compounds were administered every second day (6 injections in total). The control group received only the respective vehicle (DMSO/Cremophor EL/saline). The i.p. injection was administered in the lower left abdominal quadrant. Following this, the weight fluctuations and any signs of distress in the mice were observed every other day for an additional two weeks.

***In vivo AB12 mouse allograft model (i.p. route)***

BALB/c mice were subcutaneously injected with 0.1 mL of AB12 cells ($1 \times 10^7$ cells/mL) on the right flank. Animals were randomly divided into 10 groups (n = 6): Group 1 (**5A**, 4 mg/kg), Group 2 (**5B**, 4 mg/kg), Group 3 (**5C**, 4 mg/kg), Group 4 (**5F**, 2.5 mg/kg), Group 5 (**5G**, 8

mg/kg), Group 6 (**5H**, 4 mg/kg), Group 7 (**2G**, 3.5 mg/kg), Group 8 (**1C**, 1.5 mg.kg), Group 9 (cisplatin (1 mg/kg) + pemetrexed (5 mg/kg)), Group 10 (vehicle). When all tumors had reached approximately 100-200 $mm^3$ in size, systemic treatment was initiated through intraperitoneal injections on Days 19, 21, and 23 (3 injections in total). Injections were administered into the lower left abdominal quadrant. Tumor dimensions were measured using a caliper. The volume ($mm^3$) of the tumor was calculated according to the formula tumor volume = (longest diameter) × (shortest diameter)/2 ×0.5. Animals were controlled for distress development. Their weight changes were monitored every second day during the whole experiment. All mice were bright, alert, and responsive during the whole study. After the end of treatment, the animals were sacrificed on Day 29 or Day 27 for **5F** and **5H**. After sacrificing the mice, the tumors and other organs were harvested for further examinations.

***In vivo vaccination model***

AB12 cells were seeded into Cellstar 24-well plates (Greiner Bio-One) at a density of $1.8 \times 10^4$ cells/ml (1 ml per well) and allowed to grow exponentially for 16 hours. Subsequently, the medium was replaced with 1 ml of fresh medium containing compounds of interest at predetermined concentrations (with a minimum of 4 wells per treatment condition). These concentrations were selected to induce 65-75% cell death. (**1C**, $5 \times IC_{50}$, 4 h; **2C**, **5C** and **6C**, $5 \times IC_{50}$, 100 min; **2G**, $5 \times IC_{50}$, 6 h; pemetrexed $10 \times IC_{50}$, 16 h). Subsequently, the culture medium was aspirated, and the cells were washed twice with 1 mL of PBS. The cell concentration was adjusted to $3 \times 10^5$ cells (including dead/dying/live cells) per 100 μL of PBS, and the ratio of dead/live cells was assessed using the Invitrogen Countess 3 cell counter. In the subsequent experiment, BALB/c mice (8 mice per group) were subcutaneously injected with 0.1 mL of AB12 cells ($3 \times 10^5$ cells/mL, including dead/dying/live cells) or the corresponding vehicle (PBS) on the left flank. Tumor growth was monitored daily. No mice developed tumors on the left flank. After 7 days, the mice were subcutaneously injected with

0.1 mL of AB12 cells ($3 \times 10^5$ cells/mL, live cells) on the right flank. Regular measurements of body weights and tumor sizes were taken. Mice that developed palpable tumors were euthanized for further analysis.

**Tumor analysis**

***RNA sequencing***

Tissue sample fragmentation was carried out using a grinding machine. Snap-frozen AB12 tumor tissues were transferred to corresponding grinding tubes, and 1.5 mL of TRIzol lysis buffer was added. The mixture was transferred to the grinding machine, and the samples were ground for 30 s and then left for 5 min to ensure complete lysis of tissues. After grinding, the tissue samples were centrifuged at 12000 × *g* for 5 min at 4°C, and then the supernatant was transferred to a centrifuge tube with 300 µL of chloroform/isoamyl alcohol (24:1) and mixed thoroughly by upside-down violent shaking. The mixture was then centrifuged at 12000 × *g* for 8 min at 4°C. The supernatant was transferred to a 1.5 mL centrifuge tube, and 2/3 volume of isopropyl alcohol was added. The mixture was gently inverted and mixed, placed in a -20°C refrigerator for more than 2 h, and then centrifuged at 17500 × *g* for 25 min at 4°C. The supernatant was discarded, and the precipitate was resuspended with 0.9 mL of 75% ethanol by inverting, and then centrifuged at 17500 × *g* for 3 min at 4°C. The supernatant was discarded, and the precipitate was air-dried for 3-5 min. Finally, the precipitate was dissolved in 20-200 µL of DEPC (Diethylpyrocarbonate)-$H_2O$. The concentration and quality of extracted bacterial genomic DNA were verified by NanoDrop spectrophotometer. Strand-Specific mRNA Library Preparation, DNBseq sequencing and bioinformatic analysis were performed by BGI Tech Solutions (Hong Kong) Co., Ltd.

### *Histopathological analysis*

Tumor, liver and kidney tissues were fixed in 10% buffered formalin at RT overnight, washed twice with PBS and embedded in paraffin. The slides for the histopathological analysis were prepared by the CityU Veterinary Diagnostic Laboratory. Sections with a thickness of 4 µm were prepared, mounted on slides and deparaffinised in xylene (twice). Then sections were rehydrated in a graded series of alcohol (2× 100% alcohol and 2× 75% alcohol) and distilled water. Later, sections were stained with a haematoxylin solution, rinsed in water, passed through a 70% ethanol solution containing 1% HCl, and rinsed again with tap water. Sections were stained with eosin for 5 min and rinsed with absolute alcohol and xylene for 5 min. For immunohistochemical (IHC) analysis, selected paraffin sections along with positive and negative control tissue sections were cut at 4 µm and attached to charged slides (Leica BOND Plus Slides, Leica Microsystems Limited, Hong Kong). The antibodies used were anti-IBA1 (1:200; mouse polyclonal antibody, Dako, Tokyo, Japan) and anti-CRT (1:100; mouse monoclonal antibody (1G6A7), Thermo Fisher Scientific, # MA5-15382). Tissue sections with the primary antibody replaced by antibody diluent (Leica Biosystems Newcastle) served as negative controls. The image analysis was performed by the board-certified pathologist using QuPath algorithm, focusing on quantifying H-score and the percentage of positive tumor cells with the entire tumor area.[9] Additionally, kidney and liver tissues were analyzed across full tissue sections to calculate the percentage of positive cells. The H-score was determined by combining staining intensity and the proportion of positive cells, enabling precise evaluation of tumor positivity.

### *Organ distribution studies*

Organs and tumor tissues were collected from AB12 tumor-bearing mice at the experimental endpoint and flash-frozen in liquid nitrogen. The organs and tumors of the vehicle-treated

mouse were used for the measurements of the background. The organ parts were lyophilized for 4 d, weighed, mechanically minced and digested in 100 µl of ultrapure 65% $HNO_3$ at 105°C for 3 d. The digested lysate was further reconstituted back to 5% v/v $HNO_3$ solution with 60% ultrapure $HNO_3$ and ultrapure water and filtered through 0.22 µm hydrophilic PTFE filter (CTECH). The Au content was quantified using ICP-MS in He mode, with Re being used as internal standard. Au was measured at m/z 197 whereas Re was measured at m/z 186. 8 Au standards for standard calibration were prepared before each analysis (0, 0.5, 1, 2, 5, 10, 20, 40, 80 ppb). All readings were taken with at least triplicate.

**Statistical analysis**

All data were analyzed using GraphPad Prism software (version 9.2). For *in vitro* studies, data are presented as mean ± standard deviation (SD) of at least three independent experiments. For *in vivo* studies, data are presented as mean ± standard deviation (SD) from individual tumors. Statistical significance was determined using unpaired one-tailed T test. P values less than 0.05 were considered statistically significant (* $p<0.05$, ** $p<0.01$, *** $p<0.001$, **** $p<0.0001$, ns = $p>0.05$). Individual p values and description of the statistical analysis for each experiment are provided in the Figure legends.

**Supplementary Table 1.** Crystallographic data for an Au(III) complex **1H**.

| **Complex** | **1C** |
|---|---|
| CCDC number | 1586393 |
| Empirical formula | $[C_{17}H_{18}AuN_2S_2]PF_6$ |
| Formula weight | 656.39 |
| Temperature [K] | 100(2) |
| Wavelength [Å] | 0.71073 |
| Crystal system | monoclinic |
| Space group | $P2_1/c$ |
| *a* [Å] | 13.5669(14) |
| *b* [Å] | 25.873(3) |
| c [Å] | 12.4108(12) |
| α [°] | 90 |
| β [°] | 111.521(4) |
| γ [°] | 90 |
| Volume [$Å^3$] | 4052.7(7) |
| Z | 8 |
| Density [$Mg/m^3$] | 2.152 |
| Absorption coefficient [$mm^{-1}$] | 7.606 |
| F(000) | 2512 |
| Crystal size [$mm^3$] | 0.241 × 0.121 × 0.118 |
| $\theta$ range [deg] | 2.254 to 28.396 |
| Reflections collected | 86888 |
| Independent reflections | 10127 [R(int) = 0.1144] |
| Completeness to theta = 25.242° | 99.9 % |
| Data / restraints / parameters | 10127 / 0 / 523 |
| Goodness-of-fit on $F^2$ | 1.014 |
| Final R indices [I>2sigma(I)] | R1 = 0.0451; wR2 = 0.1017 |
| R indices (all data) | R1 = 0.0687; wR2 = 0.1113 |
| Largest diff. peak and hole [e.$Å^{-3}$] | 2.604 and -3.102 |

**Supplementary Table 2.** Cytotoxicity of Au(III) complexes in comparison with cisplatin against MPM cell lines and non-malignant lung fibroblasts.

| Compound | $IC_{50}$ (µM)[a] | | | | | | |
|---|---|---|---|---|---|---|---|
| | **AB12** | **JU77** | **LO68** | **VMC23** | **Meso84** | **Meso92** | **Met5A** |
| **1A** | 0.088 ± 0.002 | 1.1 ± 0.3 | 1.0 ± 0.3 | 0.63 ± 0.15 | 1.3 ± 0.3 | 0.19 ± 0.04 | 0.31 ± 0.07 |
| **1B** | 0.19 ± 0.02 | 0.89 ± 0.13 | 0.55 ± 0.16 | 1.6 ± 0.3 | 0.085 ± 0.007 | 0.69 ± 0.21 | 0.53 ± 0.07 |
| **1C** | 0.47 ± 0.04 | 0.13 ± 0.01 | 0.30 ± 0.06 | 0.31 ± 0.08 | 0.26 ± 0.06 | 0.33 ± 0.07 | 0.32 ± 0.08 |
| **1D** | 1.9 ± 0.2 | 0.76 ± 0.10 | 1.5 ± 0.4 | 1.4 ± 0.2 | 1.4 ± 0.1 | 1.4 ± 0.3 | 1.3 ± 0.3 |
| **1E** | 0.84 ± 0.20 | 0.85 ± 0.10 | 2.3 ± 0.3 | 1.3 ± 0.2 | 1.2 ± 0.1 | 2.0 ± 0.3 | 0.69 ± 0.13 |
| **1F** | 0.20 ± 0.06 | 1.5 ± 0.1 | 1.7 ± 0.3 | 0.93 ± 0.28 | 0.19 ± 0.04 | 0.22 ± 0.06 | 0.18 ± 0.05 |
| **1G** | 1.1 ± 0.1 | 0.22 ± 0.03 | 0.50 ± 0.12 | 0.72 ± 0.14 | 0.50 ± 0.04 | 1.4 ± 0.2 | 0.43 ± 0.09 |
| **1H** | 0.091 ± 0.018 | 0.62 ± 0.12 | 0.37 ± 0.10 | 1.4 ± 0.1 | 0.70 ± 0.09 | 1.1 ± 0.1 | 0.13 ± 0.03 |
| **2A** | 0.17 ± 0.02 | 2.8 ± 0.2 | 1.2 ± 0 | 8.4 ± 1.0 | 0.22 ± 0.06 | 0.26 ± 0.04 | 0.43 ± 0.08 |
| **2B** | 0.30 ± 0.06 | 3.0 ± 0.4 | 2.2 ± 0.4 | 6.6 ± 1.7 | 0.42 ± 0.12 | 6.2 ± 1.7 | 0.53 ± 0.08 |
| **2C** | 2.7 ± 0.3 | 2.7 ± 0.4 | 3.0 ± 0.3 | 2.4 ± 0.5 | 3.9 ± 0.7 | 3.1 ± 0.7 | 1.4 ± 0 |
| **2D** | 3.9 ± 0.5 | 2.3 ± 0.7 | 2.2 ± 0.4 | 2.4 ± 0.5 | 3.0 ± 0.3 | 5.4 ± 1.3 | 2.5 ± 0.4 |
| **2E** | 2.7 ± 0.6 | 1.6 ± 0.2 | 2.3 ± 0.1 | 2.0 ± 0.6 | 1.6 ± 0.4 | 1.6 ± 0.2 | 3.0 ± 0.6 |
| **2F** | 0.29 ± 0.04 | 1.1 ± 0.1 | 0.72 ± 0.12 | 0.97 ± 0.26 | 0.56 ± 0.10 | 1.0 ± 0.3 | 0.73 ± 0.12 |
| **2G** | 1.2 ± 0.2 | 1.6 ± 0.2 | 2.6 ± 0.6 | 3.0 ± 0.3 | 2.3 ± 0.2 | 2.2 ± 0.3 | 1.3 ± 0 |
| **2H** | 0.31 ± 0.08 | 2.0 ± 0.5 | 1.9 ± 0.3 | 8.4 ± 1.3 | 0.30 ± 0.07 | 5.2 ± 1.2 | 2.2 ± 0.5 |
| **3A** | 0.14 ± 0.04 | 1.9 ± 0.5 | 0.99 ± 0.19 | 0.64 ± 0.06 | 1.2 ± 0.2 | 0.53 ± 0.11 | 0.44 ± 0.13 |
| **3B** | 0.22 ± 0.01 | 1.2 ± 0 | 0.93 ± 0.18 | 7.5 ± 1.4 | 1.4 ± 0.3 | 1.1 ± 0.3 | 1.4 ± 0.1 |

| | | | | | | | |
|---|---|---|---|---|---|---|---|
| **3G** | 0.35 ± 0.07 | 1.1 ± 0.2 | 1.2 ± 0.1 | 1.4 ± 0.2 | 1.8 ± 0.3 | 1.8 ± 0.2 | 1.5 ± 0.2 |
| **4A** | 0.29 ± 0.04 | 1.4 ± 0.2 | 1.3 ± 0.3 | 4.7 ± 0.7 | 0.19 ± 0.03 | 0.16 ± 0.03 | 0.72 ± 0.21 |
| **4B** | 0.33 ± 0.10 | 4.8 ± 0.7 | 2.9 ± 0.8 | 7.3 ± 1.8 | 1.7 ± 0.5 | 6.3 ± 1.0 | 0.43 ± 0.13 |
| **4C** | 3.1 ± 0.4 | 1.6 ± 0.1 | 3.1 ± 0.3 | 2.5 ± 0.2 | 2.7 ± 0.8 | 4.5 ± 0.8 | 3.0 ± 0.7 |
| **4D** | 2.3 ± 0.1 | 1.7 ± 0.2 | 2.3 ± 0.6 | 4.2 ± 0.7 | 2.9 ± 0.7 | 4.1 ± 0.9 | 3.2 ± 0.3 |
| **4E** | 1.9 ± 0.4 | 3.2 ± 0.3 | 4.5 ± 0.5 | 4.3 ± 0.8 | 2.3 ± 0.6 | 5.3 ± 0.2 | 1.4 ± 0.3 |
| **4F** | 0.32 ± 0.05 | 3.3 ± 0.8 | 1.4 ± 0.2 | 2.9 ± 0.3 | 0.34 ± 0.10 | 1.7 ± 0.5 | 0.74 ± 0.09 |
| **4G** | 0.23 ± 0.06 | 2.4 ± 0.7 | 1.6 ± 0.2 | 3.5 ± 0.7 | 1.8 ± 0.4 | 4.8 ± 0.5 | 2.0 ± 0.3 |
| **4H** | 0.22 ± 0.03 | 2.4 ± 0.2 | 2.5 ± 0.1 | 2.8 ± 0.3 | 0.33 ± 0.08 | 1.7 ± 0.3 | 0.68 ± 0.14 |
| **5A** | 0.15 ± 0.01 | 0.37 ± 0.03 | 0.27 ± 0.07 | 0.74 ± 0.06 | 0.10 ± 0.02 | 0.15 ± 0.01 | 0.19 ± 0.05 |
| **5B** | 0.053 ± 0.002 | 0.19 ± 0.05 | 0.24 ± 0.02 | 0.30 ± 0.09 | 0.063 ± 0.019 | 0.21 ± 0.06 | 0.18 ± 0.05 |
| **5C** | 0.13 ± 0.03 | 0.086 ± 0.022 | 0.36 ± 0.07 | 0.64 ± 0.14 | 0.18 ± 0.03 | 0.41 ± 0.06 | 0.065 ± 0.015 |
| **5D** | 1.1 ± 0 | 0.52 ± 0.08 | 0.95 ± 0.13 | 1.3 ± 0.3 | 1.1 ± 0.3 | 1.1 ± 0.2 | 1.0 ± 0.2 |
| **5E** | 0.18 ± 0.01 | 0.44 ± 0.13 | 0.76 ± 0.18 | 0.34 ± 0.04 | 0.51 ± 0.10 | 0.40 ± 0.05 | 0.28 ± 0.08 |
| **5F** | 0.17 ± 0.02 | 0.47 ± 0.11 | 0.67 ± 0.19 | 1.1 ± 0.3 | 0.18 ± 0.05 | 0.36 ± 0.09 | 0.37 ± 0.03 |
| **5G** | 0.33 ± 0.09 | 0.22 ± 0.04 | 0.43 ± 0.05 | 0.73 ± 0.16 | 0.30 ± 0.07 | 0.35 ± 0.04 | 0.25 ± 0.05 |
| **5H** | 0.10 ± 0.01 | 0.40 ± 0.09 | 0.44 ± 0.09 | 0.94 ± 0.27 | 0.10 ± 0.02 | 0.82 ± 0.18 | 0.13 ± 0.01 |
| **Cisplatin** | 4.5 ± 0.8 | 4.2 ± 0.5 | 20 ± 6 | 3.6 ± 1.0 | 17 ± 2 | 4.0 ± 0.9 | 2.0 ± 0.5 |
| **Pemetrexed** | 0.023 ± 0.007 | 0.073 ± 0.014 | 0.094 ± 0.028 | 0.17 ± 0.04 | 0.027 ± 0.002 | 0.14 ± 0.04 | 0.30 ± 0.10 |

[a]Effective concentrations of 50% by means of the MTT assay with the exposure time of 72 h in mouse malignant mesothelioma cancer cell line AB12 (mesothelial cells); human malignant mesothelioma cancer cell lines JU77 (mesothelial cells), LO68 (mesothelial cells), VMC23 (epithelial cells), Meso82 (sarcomatous), and Meso92 (biphasic); human nonmalignant mesothelioma cancer cell line Met5A (mesothelial cells). Values are means ± standard deviations obtained from at least three independent experiments.

**Supplementary Table 6.** LogP values determined from retention time using RP-HPLC.

| Compound | LogP | Compound | LogP |
|---|---|---|---|
| **1A** | 1.28 | **4A** | 1.39 |
| **1B** | 2.20 | **4B** | 2.14 |
| **1C** | 3.99 | **4C** | 3.82 |
| **1D** | 3.84 | **4D** | 3.81 |
| **1E** | 2.87 | **4E** | 2.86 |
| **1F** | 1.99 | **4F** | 2.03 |
| **1G** | 2.72 | **4G** | 2.68 |
| **1H** | 2.45 | **4H** | 2.33 |
| **2A** | 1.43 | **5A** | 1.48 |
| **2B** | 2.22 | **5B** | 2.32 |
| **2C** | 3.91 | **5C** | 3.99 |
| **2D** | 3.80 | **5D** | 3.91 |
| **2E** | 2.85 | **5E** | 2.89 |
| **2F** | 2.01 | **5F** | 2.12 |
| **2G** | 2.69 | **5G** | 2.78 |
| **2H** | 2.56 | **5H** | 2.48 |
| **3A** | 1.04 | **doxorubicin** | 0.68 |
| **3B** | 1.79 | | |
| **3G** | 2.29 | | |

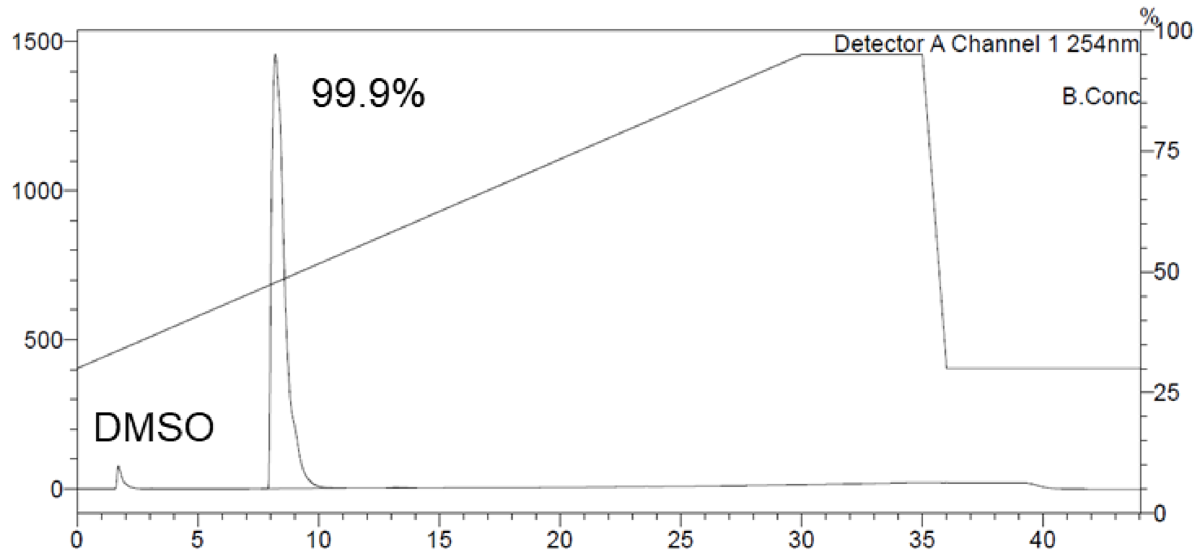


**Supplementary Figure 1**. RP-HPLC analysis of **1A**.

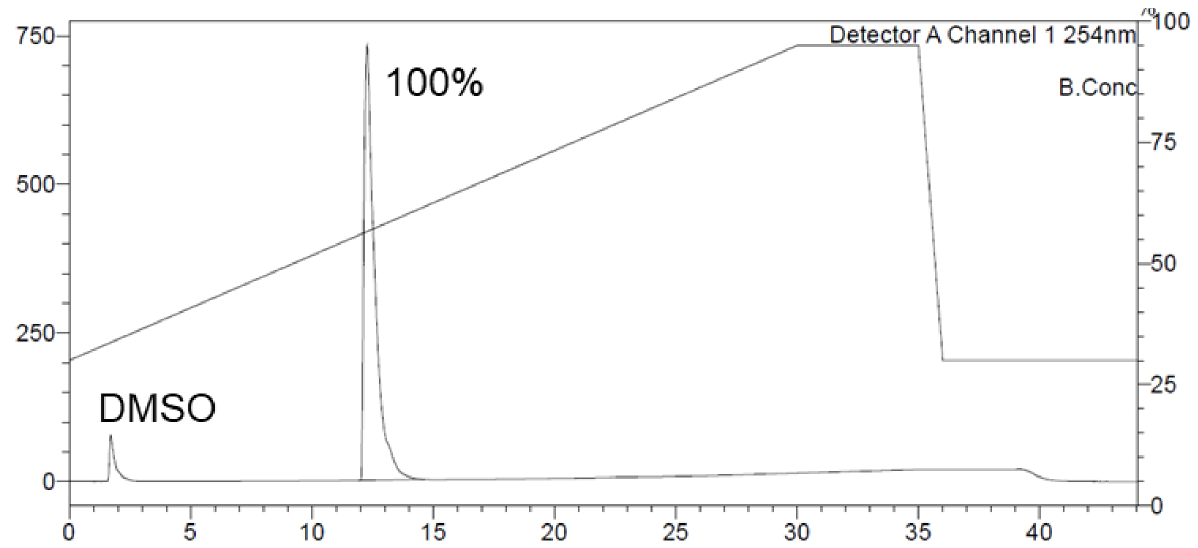


**Supplementary Figure 2**. RP-HPLC analysis of **1B**.

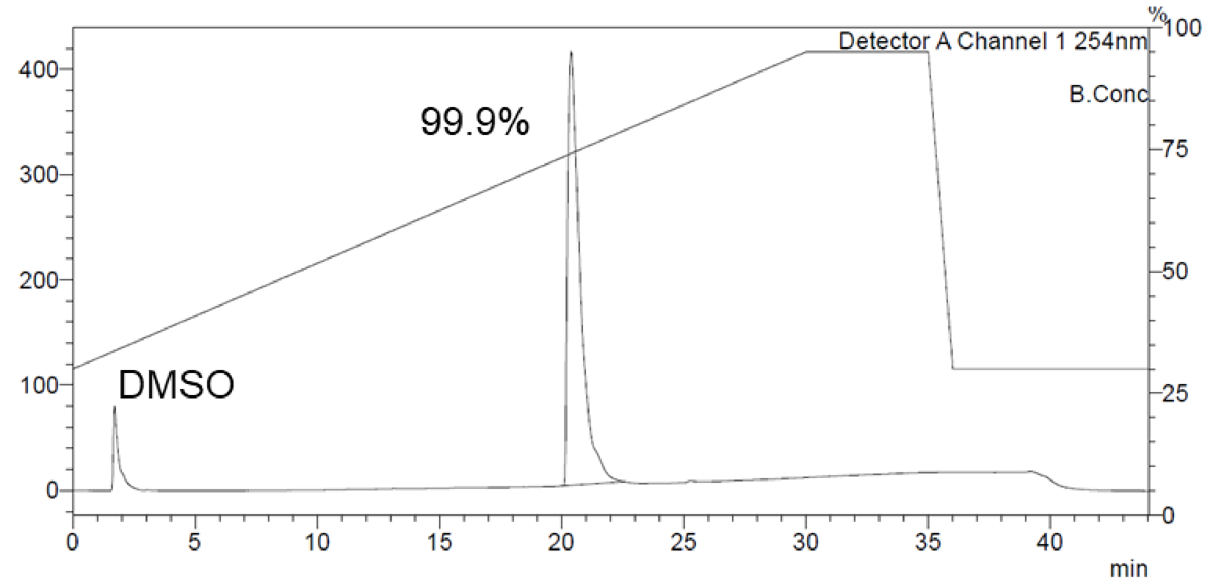


**Supplementary Figure 3**. RP-HPLC analysis of **1C**.

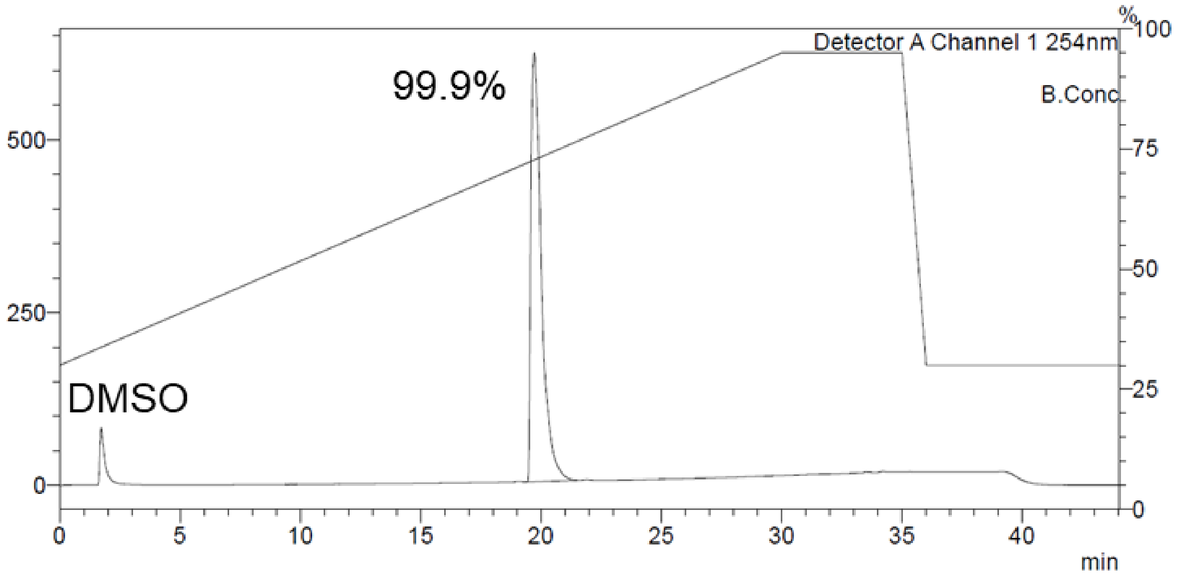


**Supplementary Figure 4**. RP-HPLC analysis of **1D**.

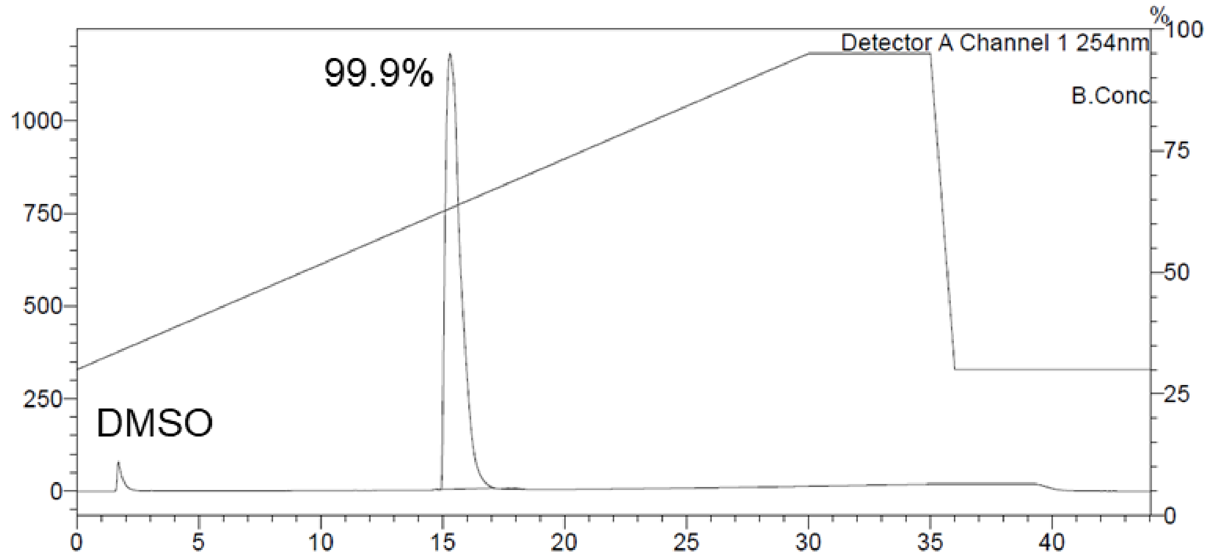


**Supplementary Figure 5**. RP-HPLC analysis of **1E.**

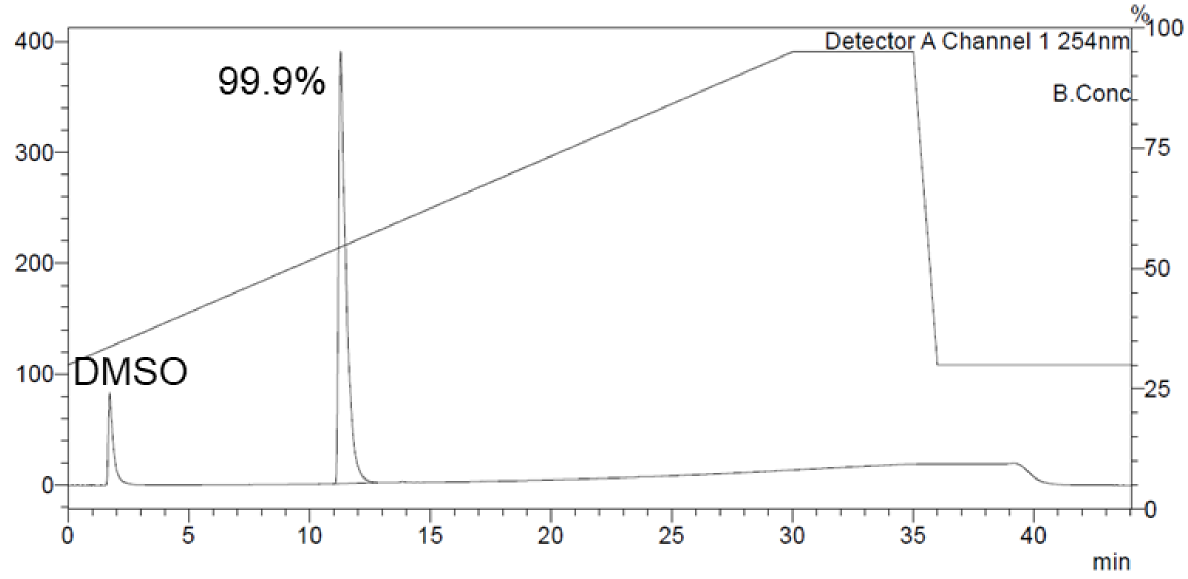


**Supplementary Figure 6**. RP-HPLC analysis of **1F.**

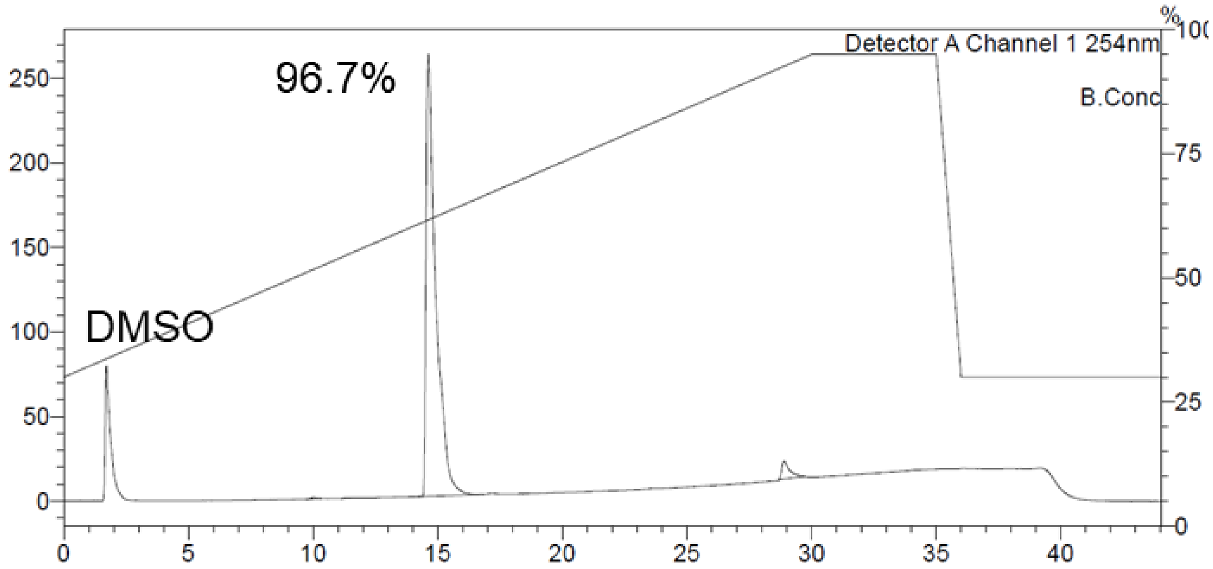


**Supplementary Figure 7**. RP-HPLC analysis of **1G.**

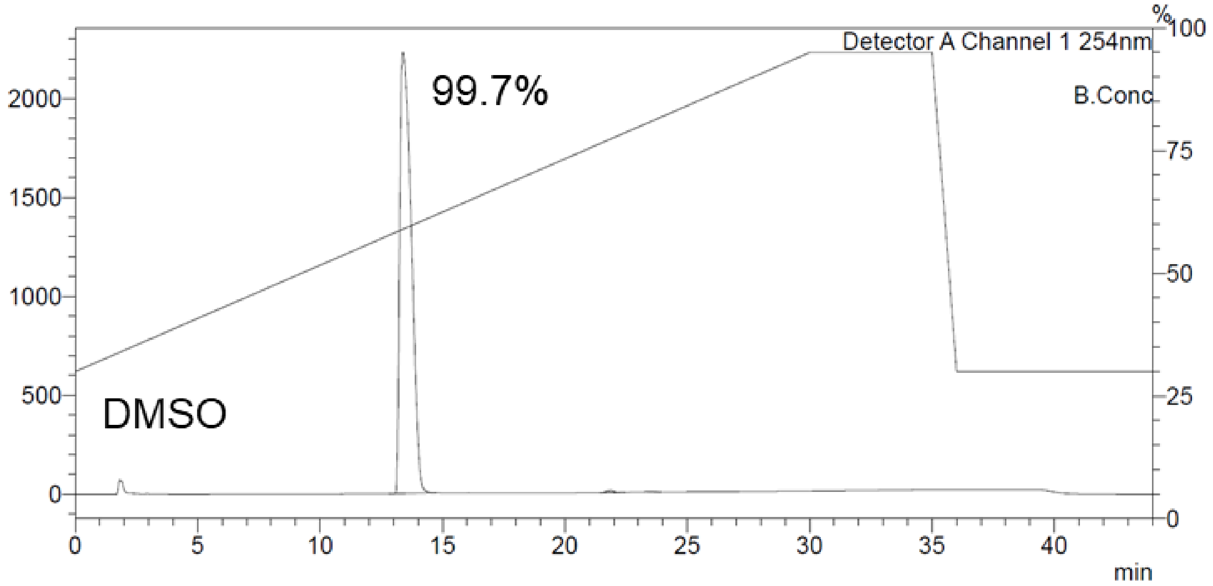


**Supplementary Figure 8**. RP-HPLC analysis of **1H.**

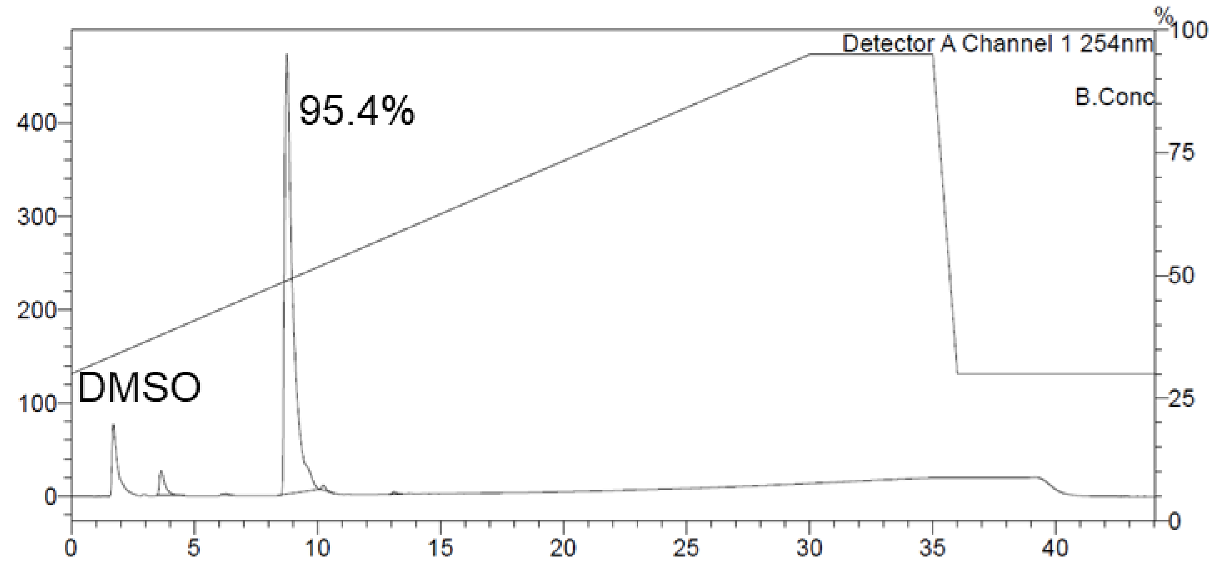


**Supplementary Figure 9**. RP-HPLC analysis of **2A**.

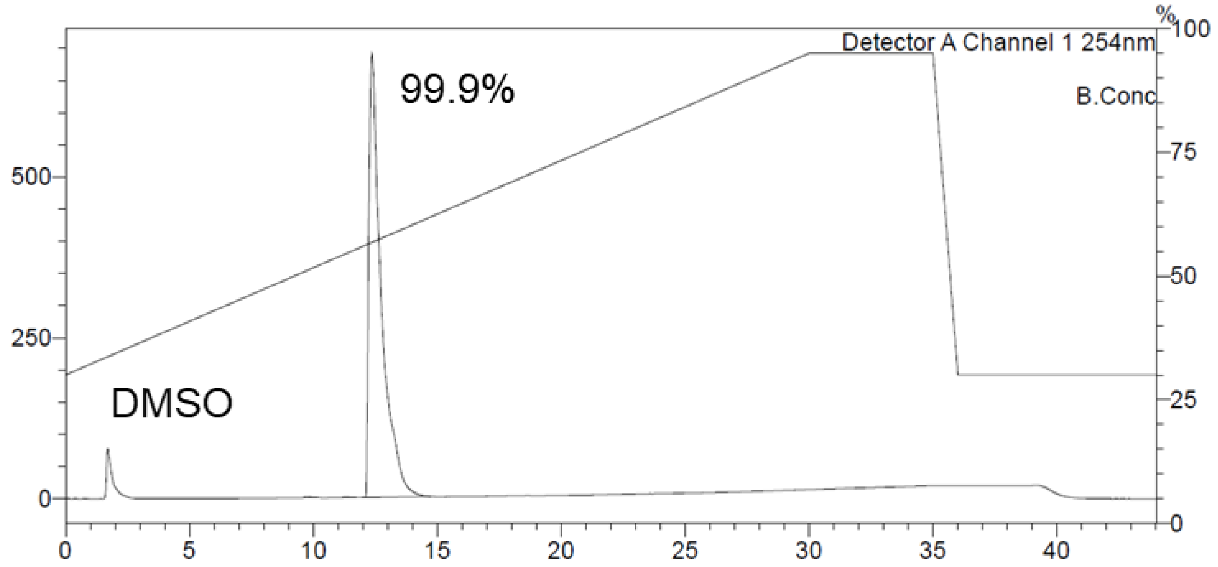


**Supplementary Figure 10**. RP-HPLC analysis of **2B.**

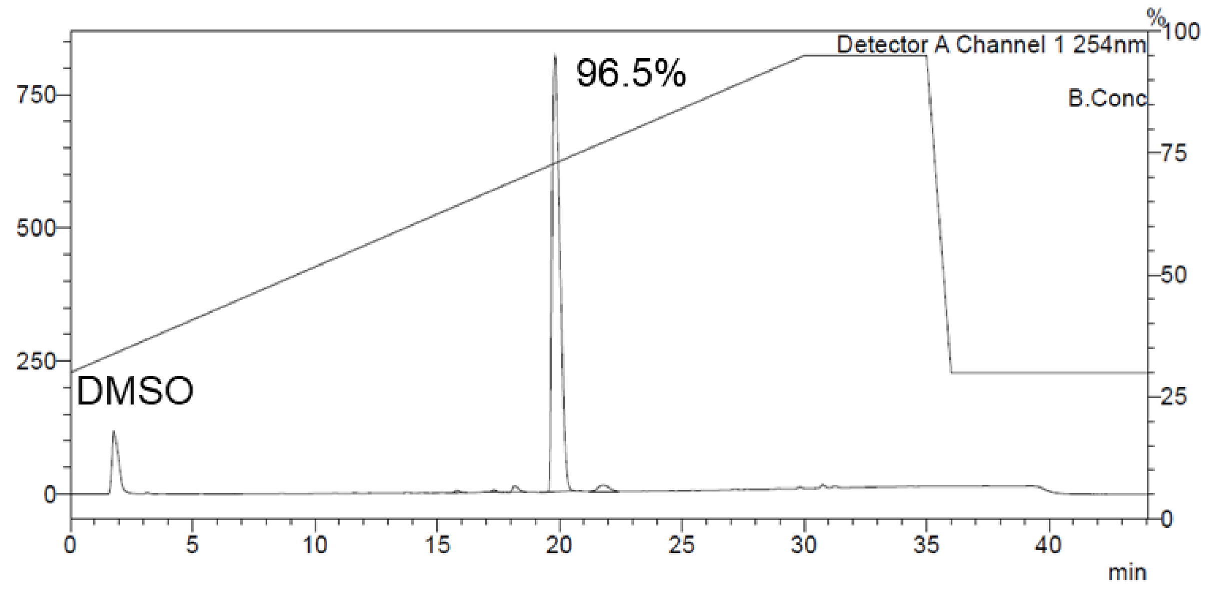


**Supplementary Figure 11**. RP-HPLC analysis of **2C.**

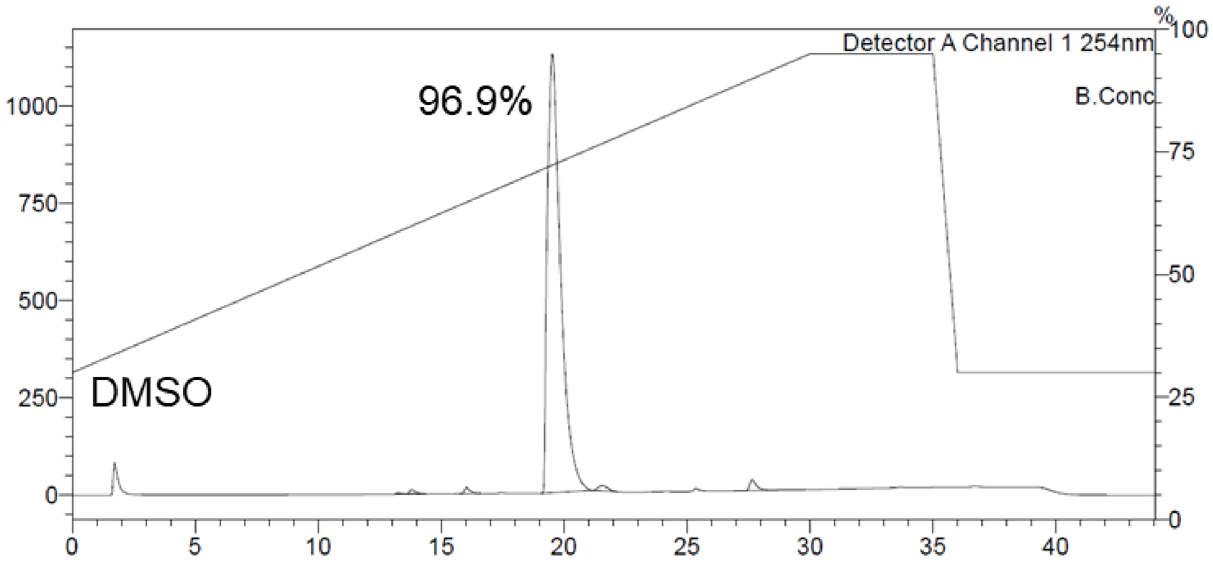


**Supplementary Figure 12**. RP-HPLC analysis of **2D**.

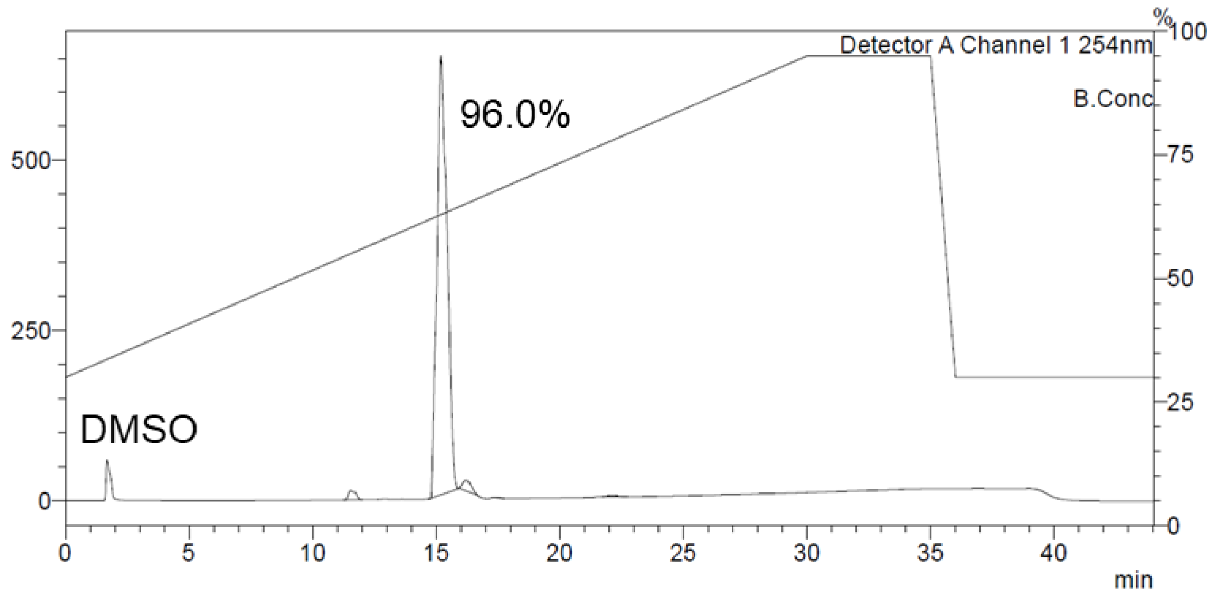


**Supplementary Figure 13**. RP-HPLC analysis of **2E**.

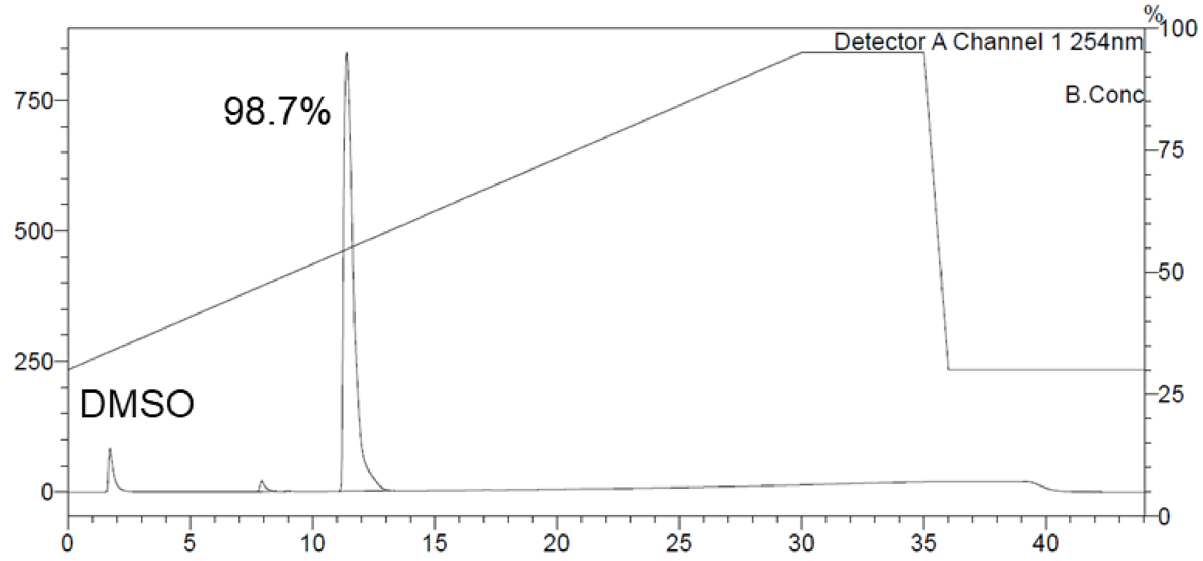


**Supplementary Figure 14**. RP-HPLC analysis of **2F**.

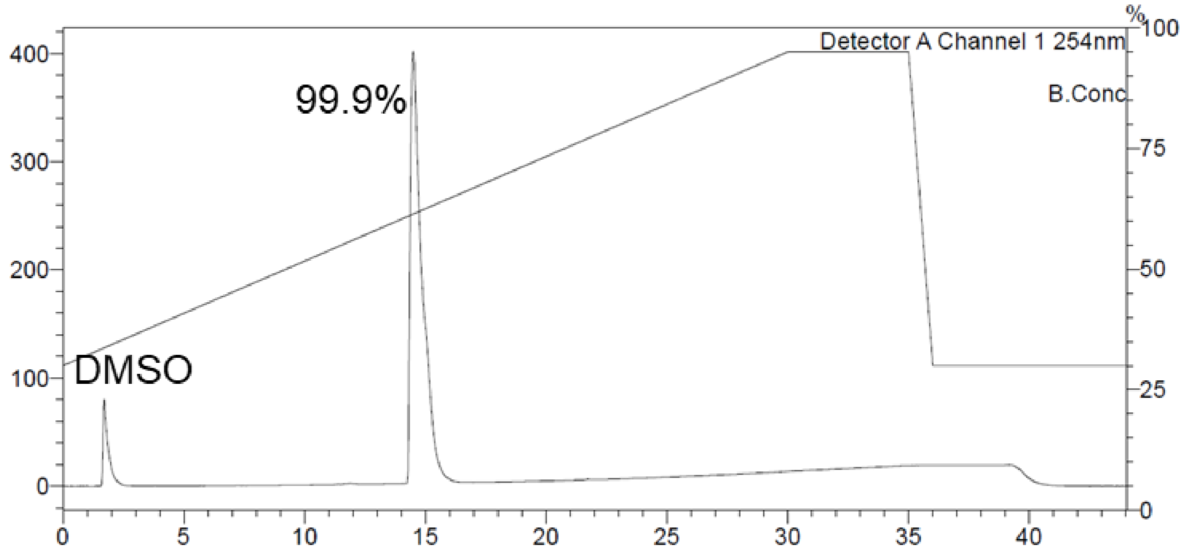


**Supplementary Figure 15**. RP-HPLC analysis of **2G**.

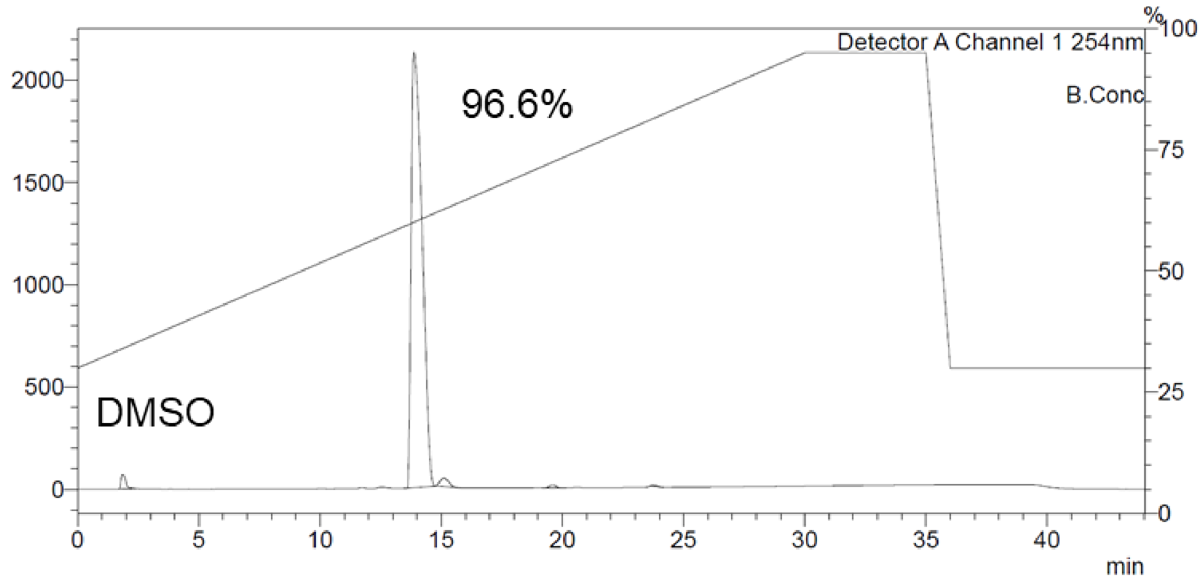


**Supplementary Figure 16**. RP-HPLC analysis of **2H**.

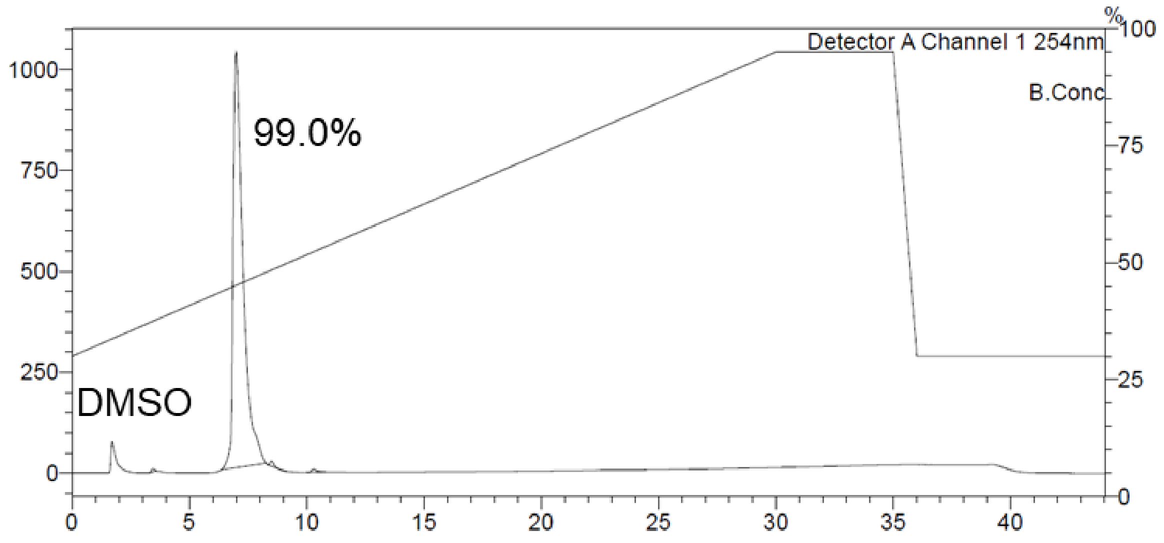


**Supplementary Figure 17**. RP-HPLC analysis of **3A**.

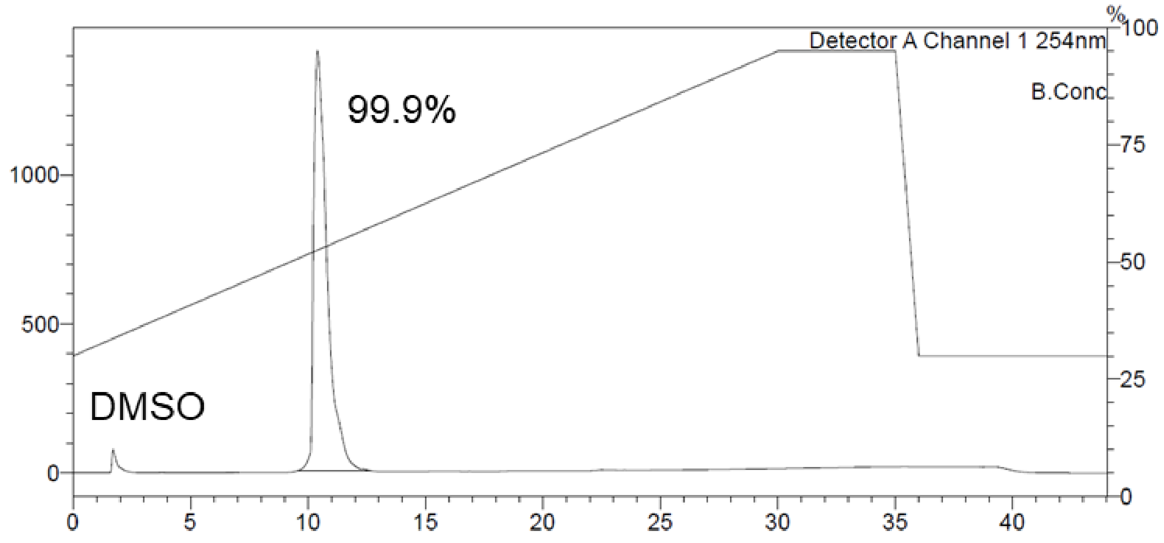


**Supplementary Figure 18**. RP-HPLC analysis of **3B**.

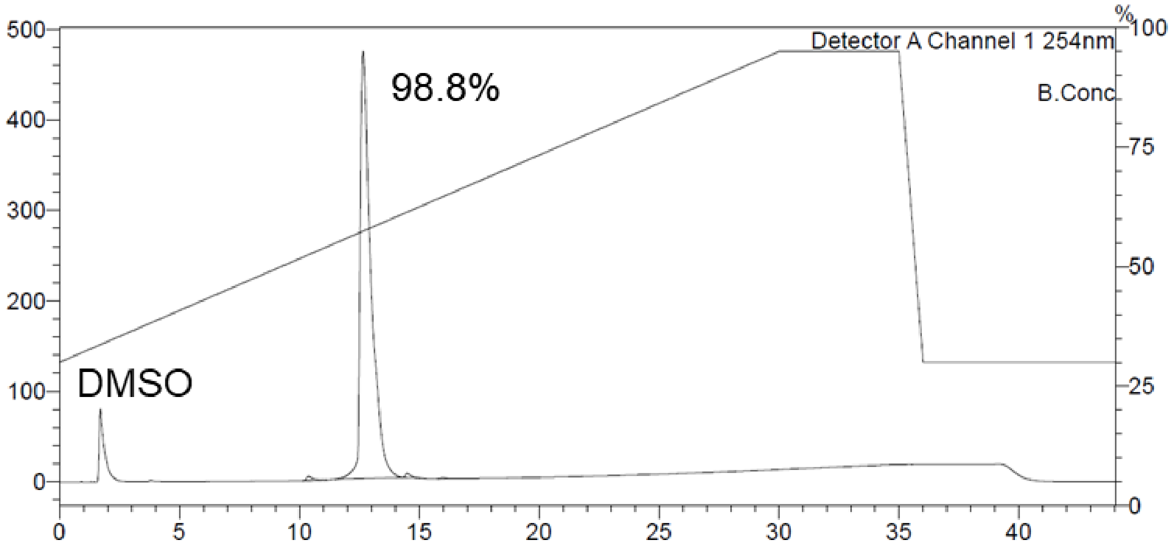


**Supplementary Figure 19**. RP-HPLC analysis of **3G**.

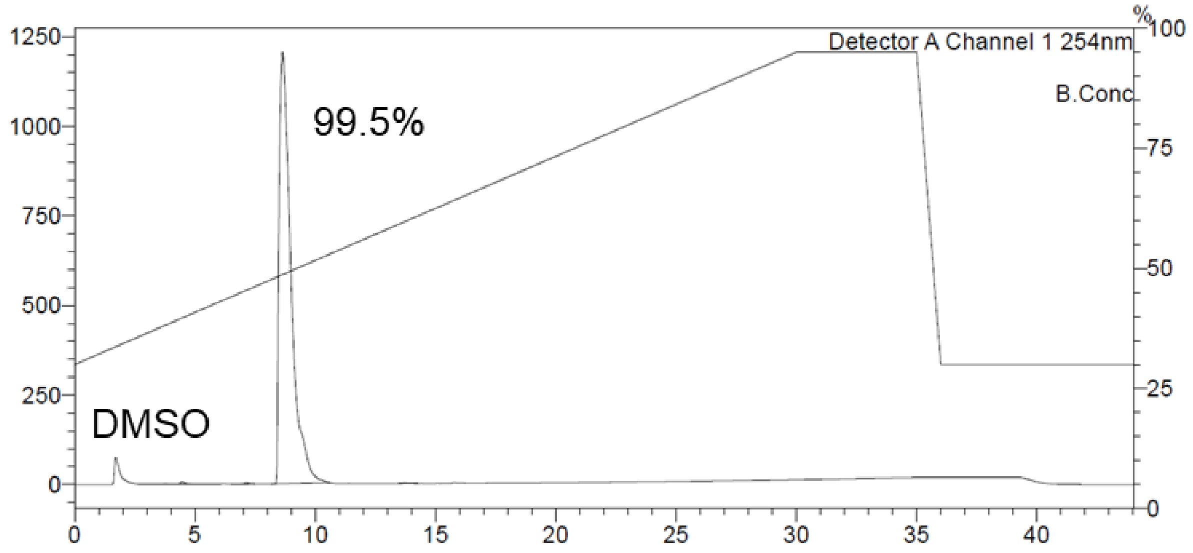


**Supplementary Figure 20**. RP-HPLC analysis of **4A.**

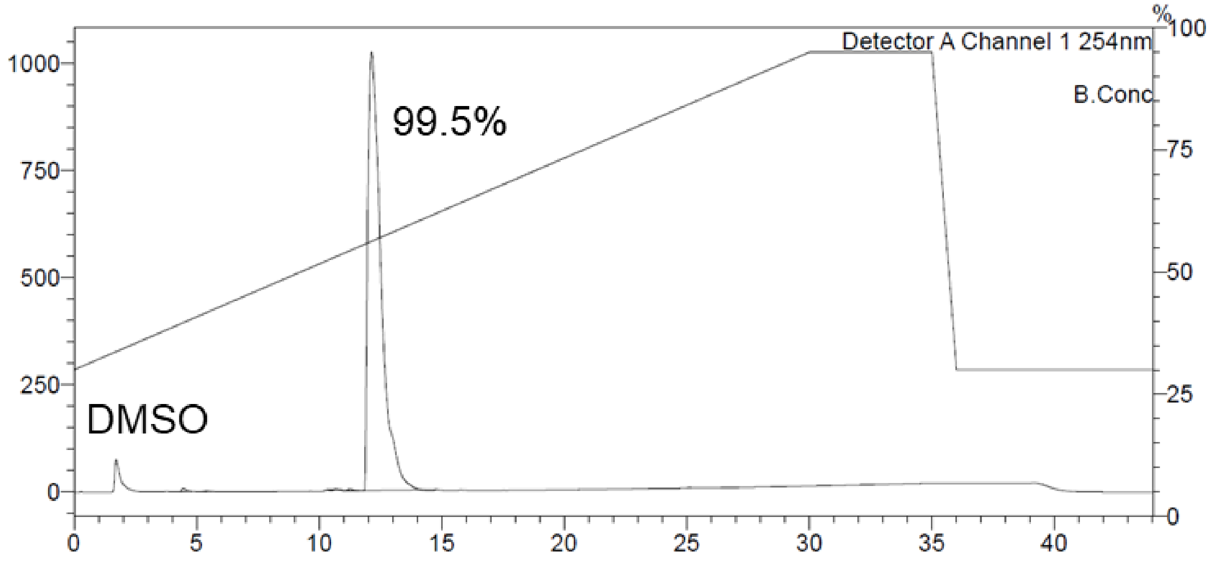


**Supplementary Figure 21**. RP-HPLC analysis of **4B.**

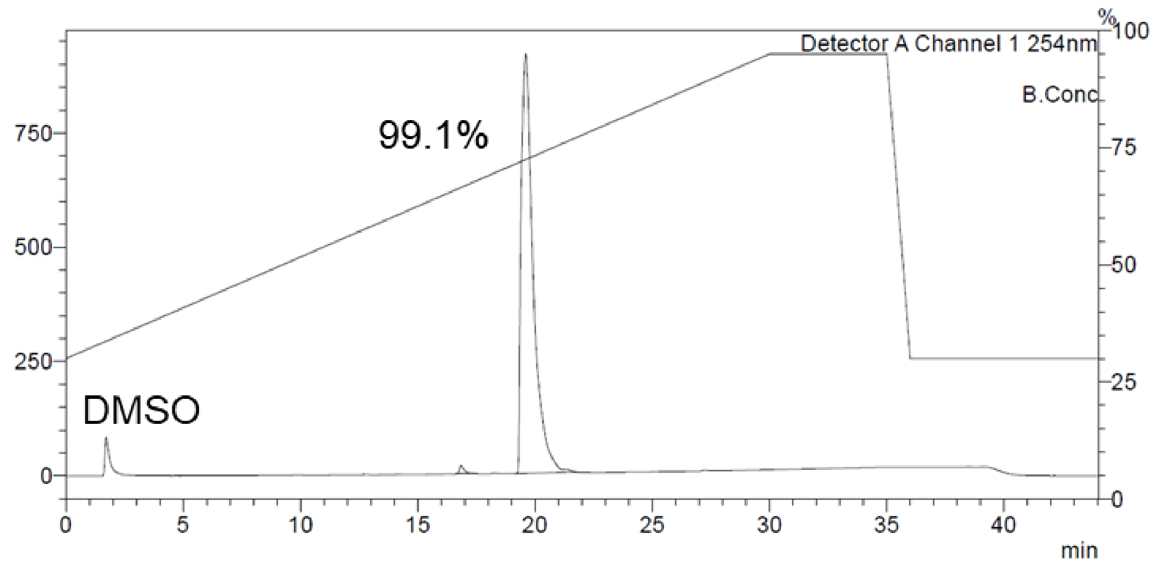


**Supplementary Figure 22**. RP-HPLC analysis of **4C.**

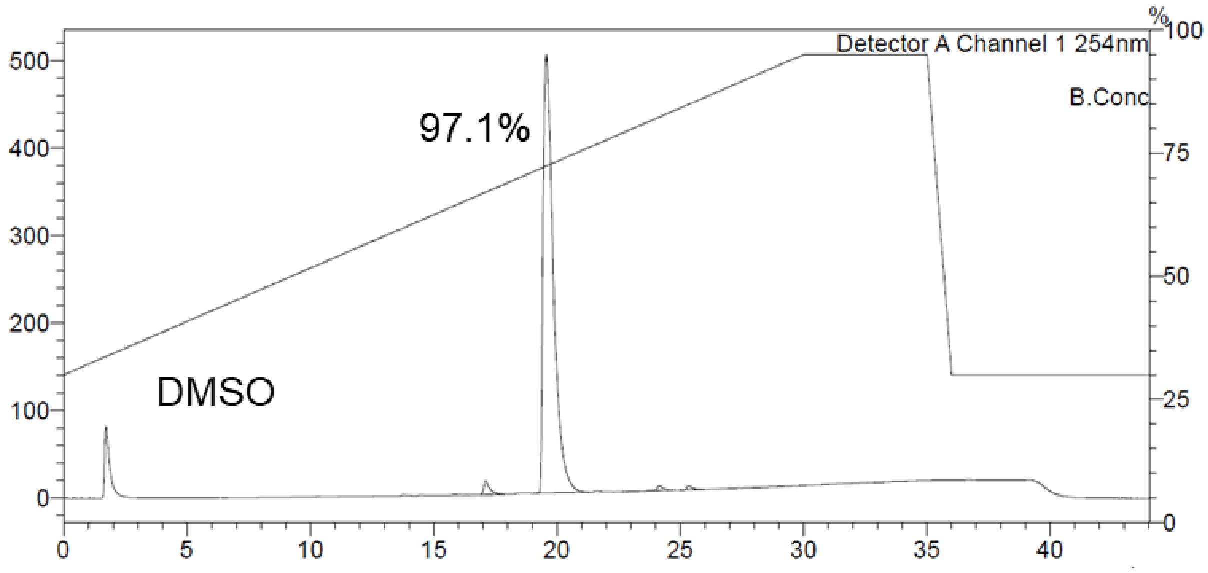


**Supplementary Figure 23**. RP-HPLC analysis of **4D**.

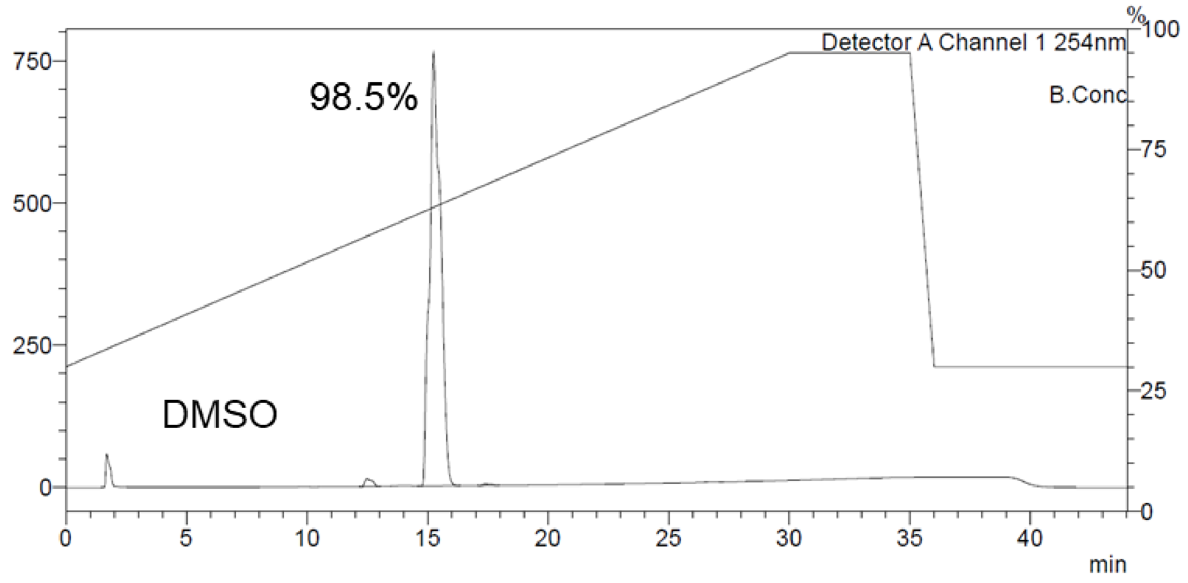


**Supplementary Figure 24**. RP-HPLC analysis of **4E**.

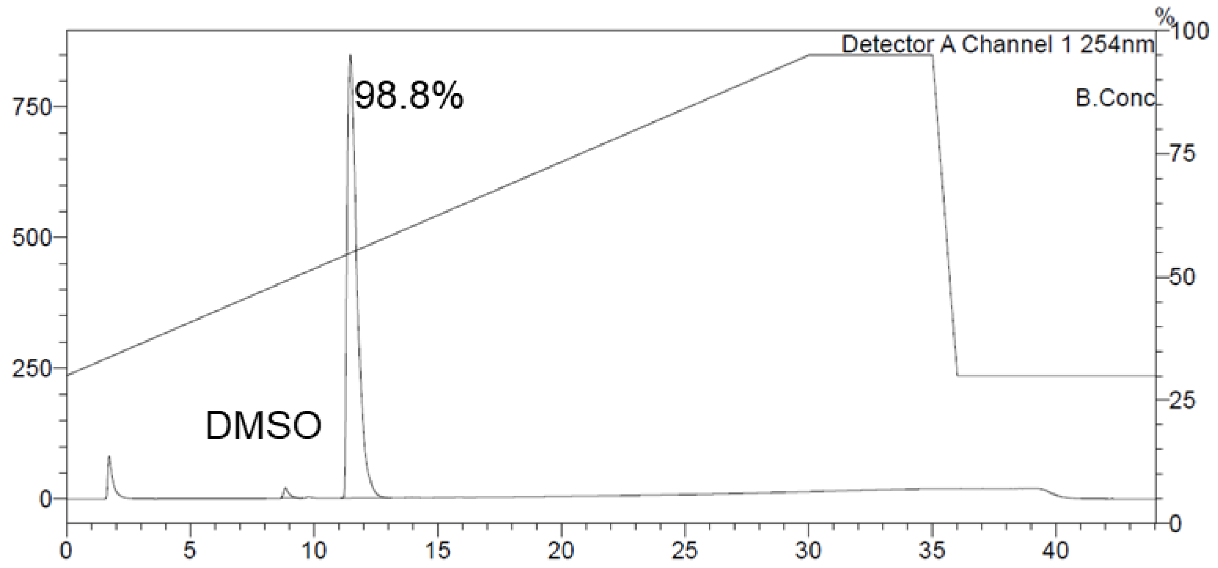


**Supplementary Figure 25**. RP-HPLC analysis of **4F.**

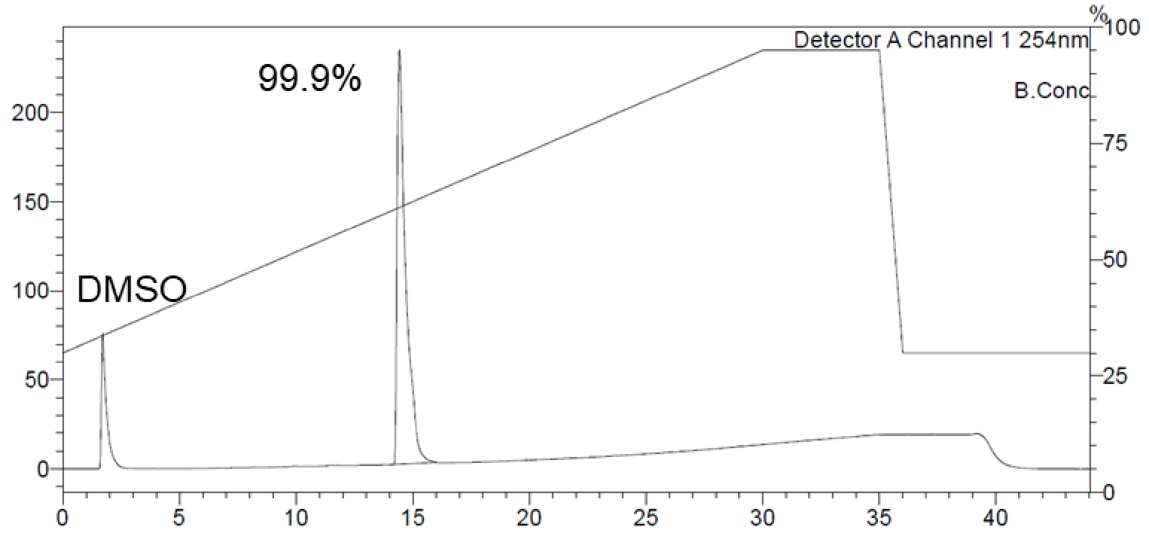


**Supplementary Figure 26**. RP-HPLC analysis of **4G**.

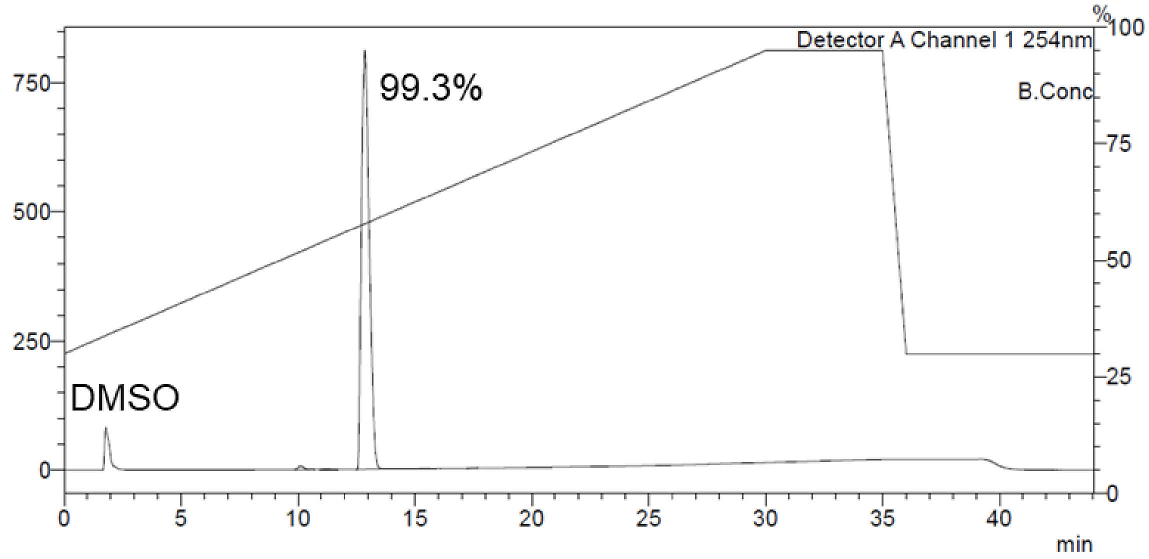


**Supplementary Figure 27**. RP-HPLC analysis of **4H.**

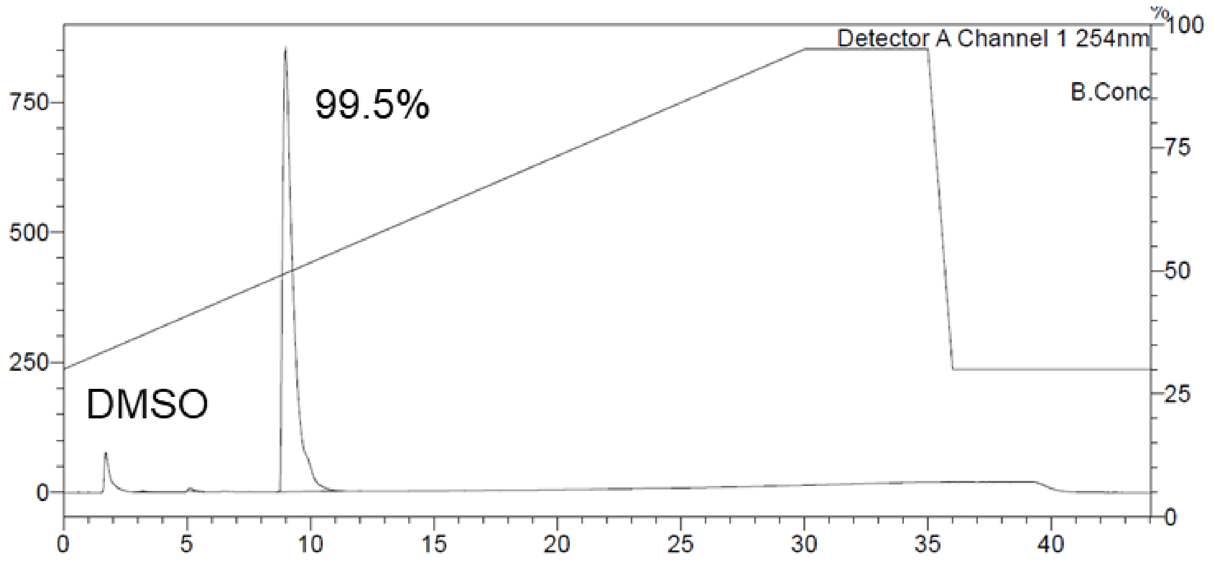


**Supplementary Figure 28**. RP-HPLC analysis of **5A**.

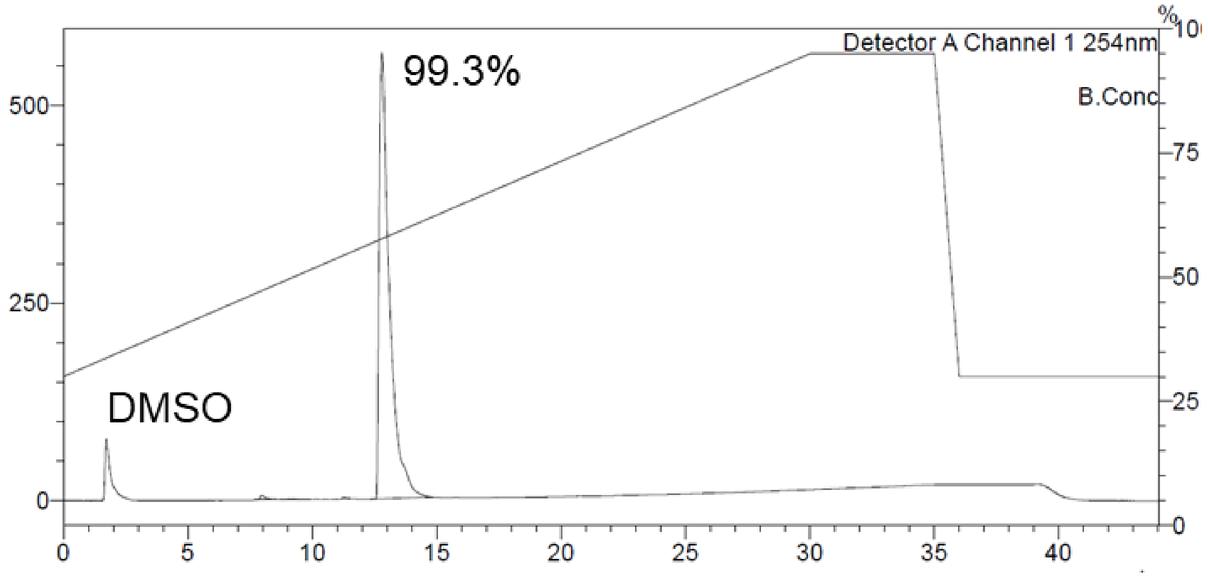


**Supplementary Figure 29**. RP-HPLC analysis of **5B**.

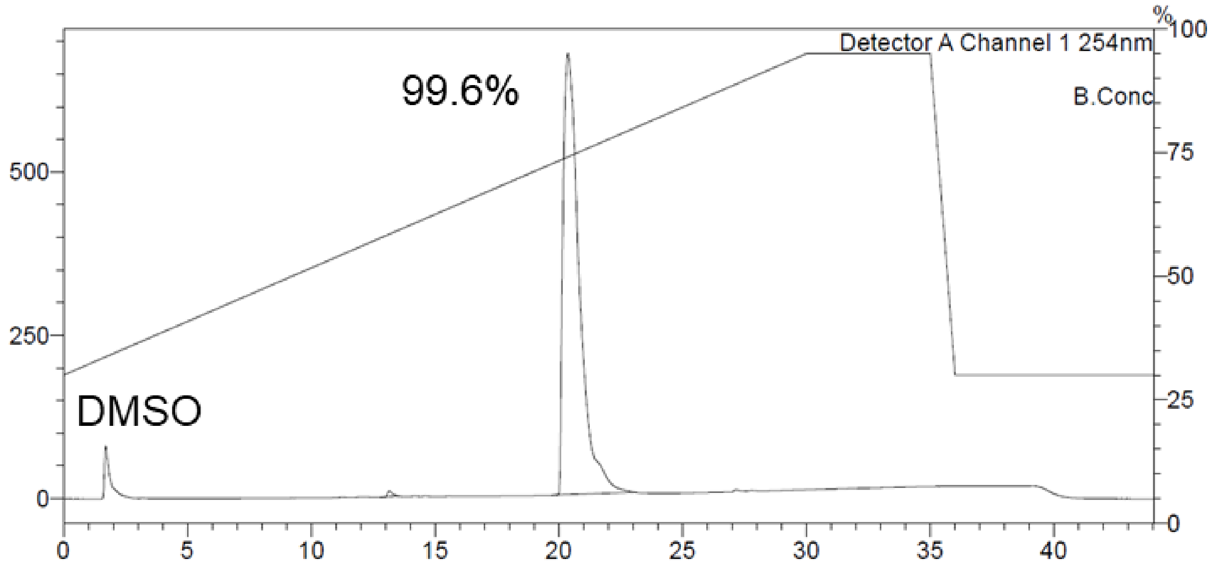


**Supplementary Figure 30**. RP-HPLC analysis of **5C**.

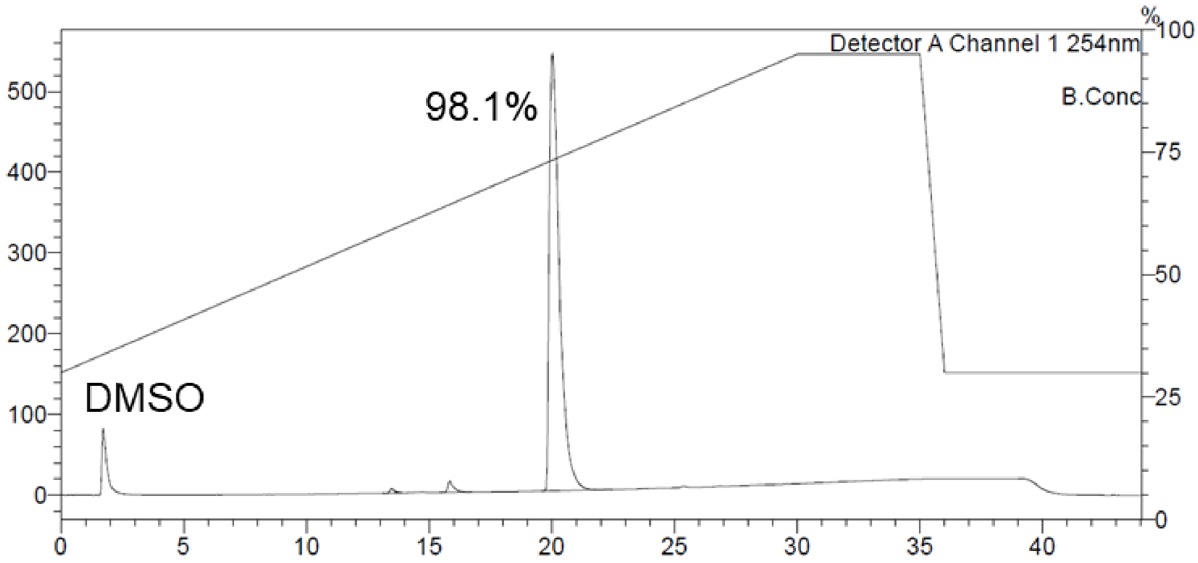


**Supplementary Figure 31**. RP-HPLC analysis of **5D**.

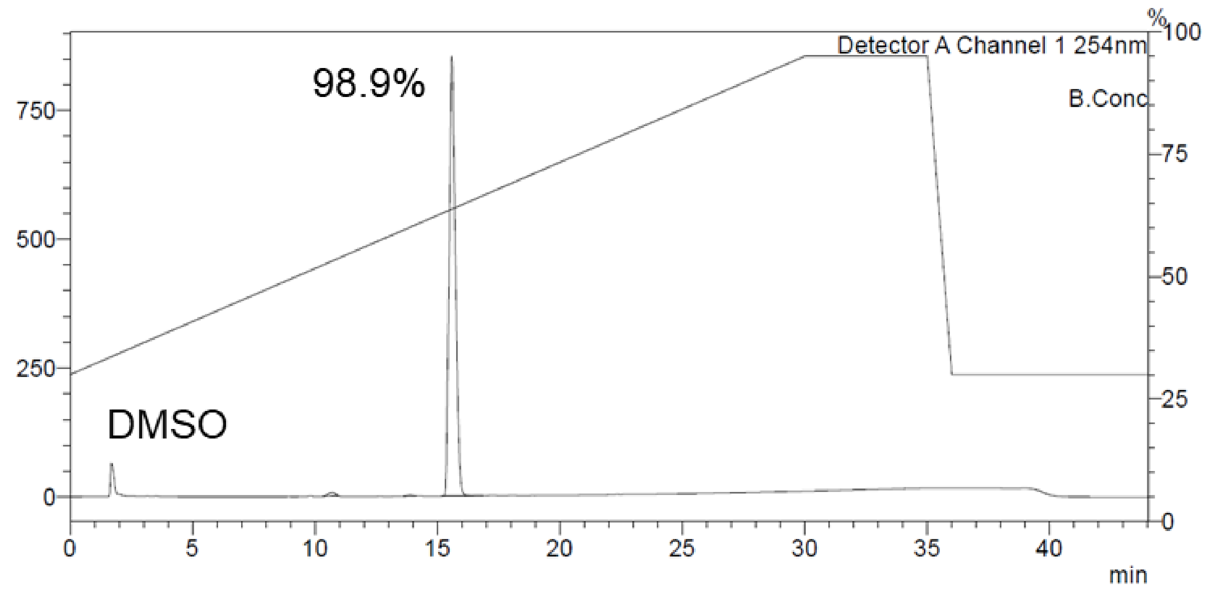


**Supplementary Figure 32**. RP-HPLC analysis of **5E**.

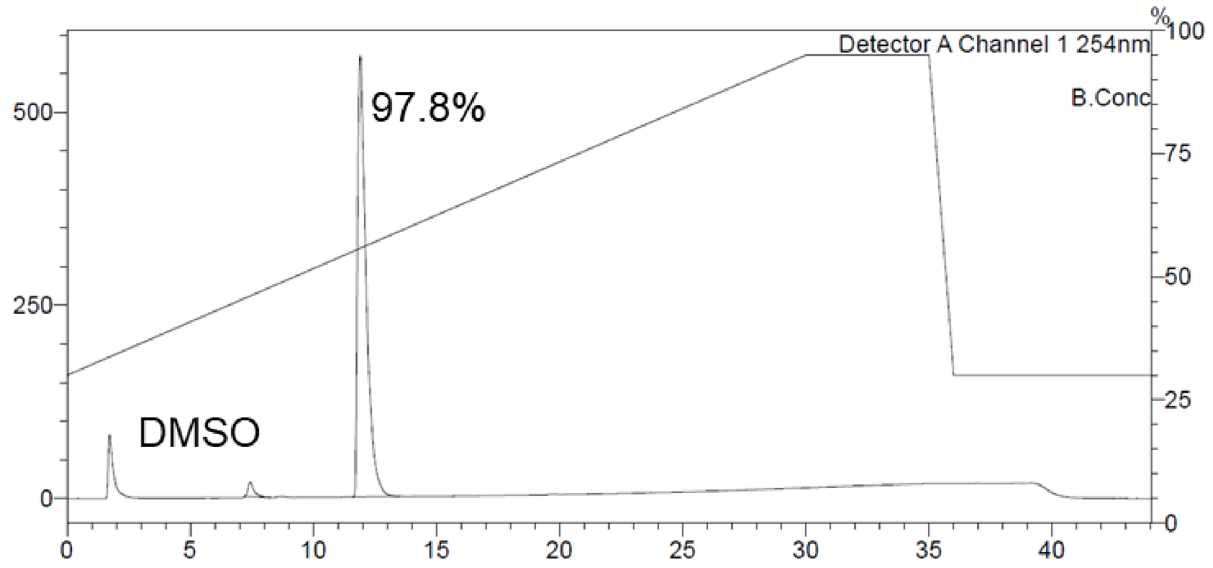


**Supplementary Figure 33**. RP-HPLC analysis of **5F**.

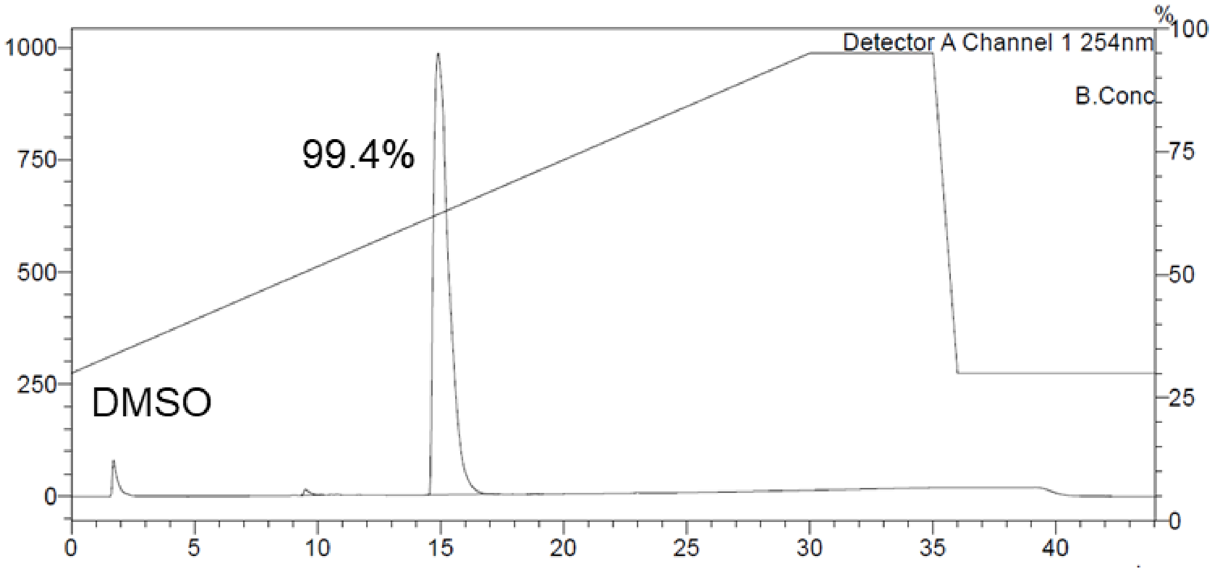


**Supplementary Figure 34**. RP-HPLC analysis of **5G**.

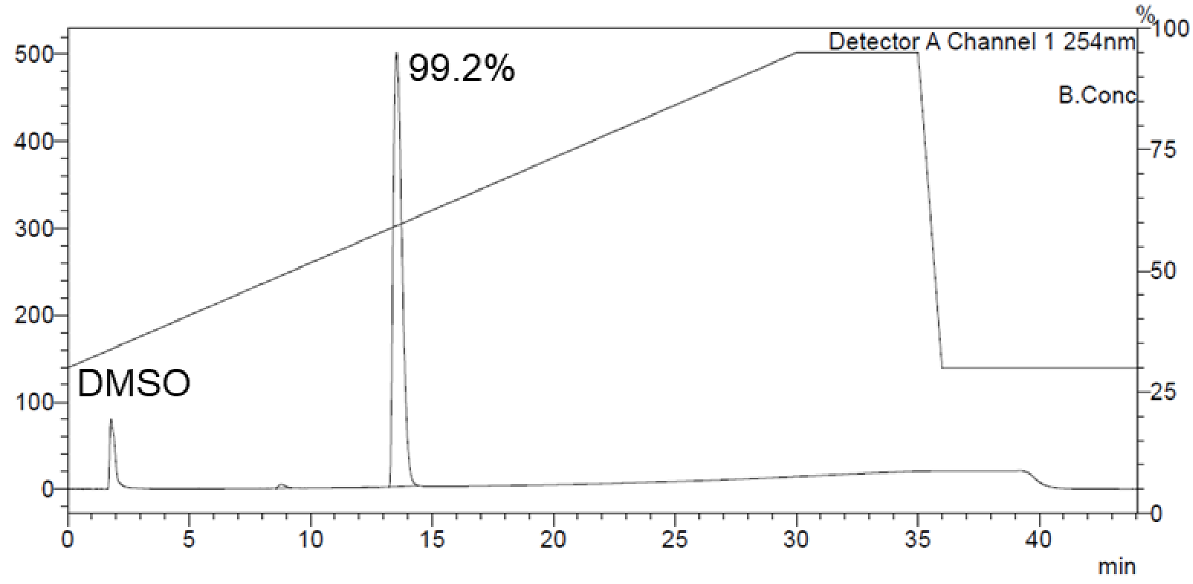


**Supplementary Figure 35**. RP-HPLC analysis of **5H**.

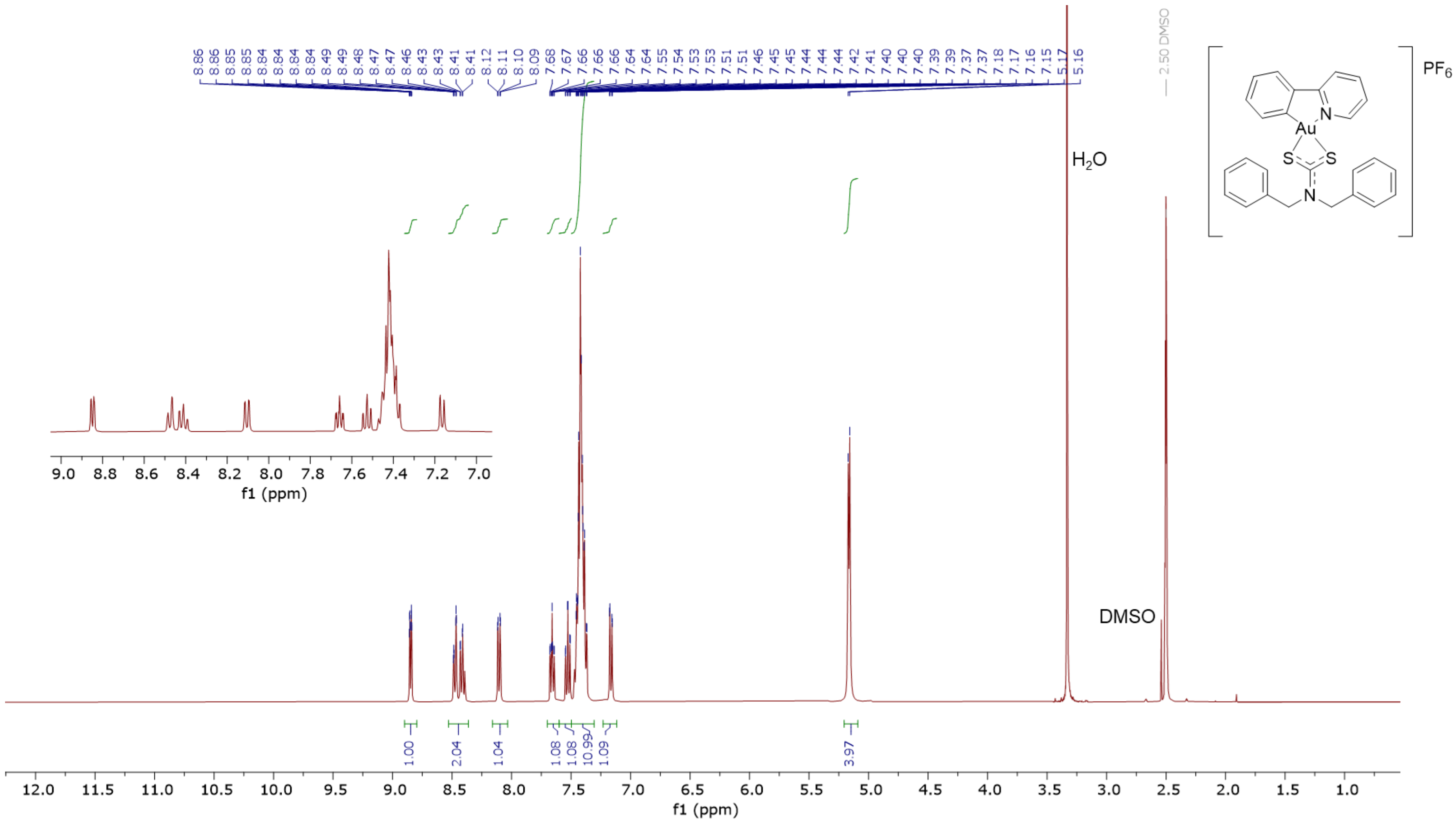


**Supplementary Figure 36**. $^{1}H$ NMR of **1D** in DMSO-d$^{6}$.

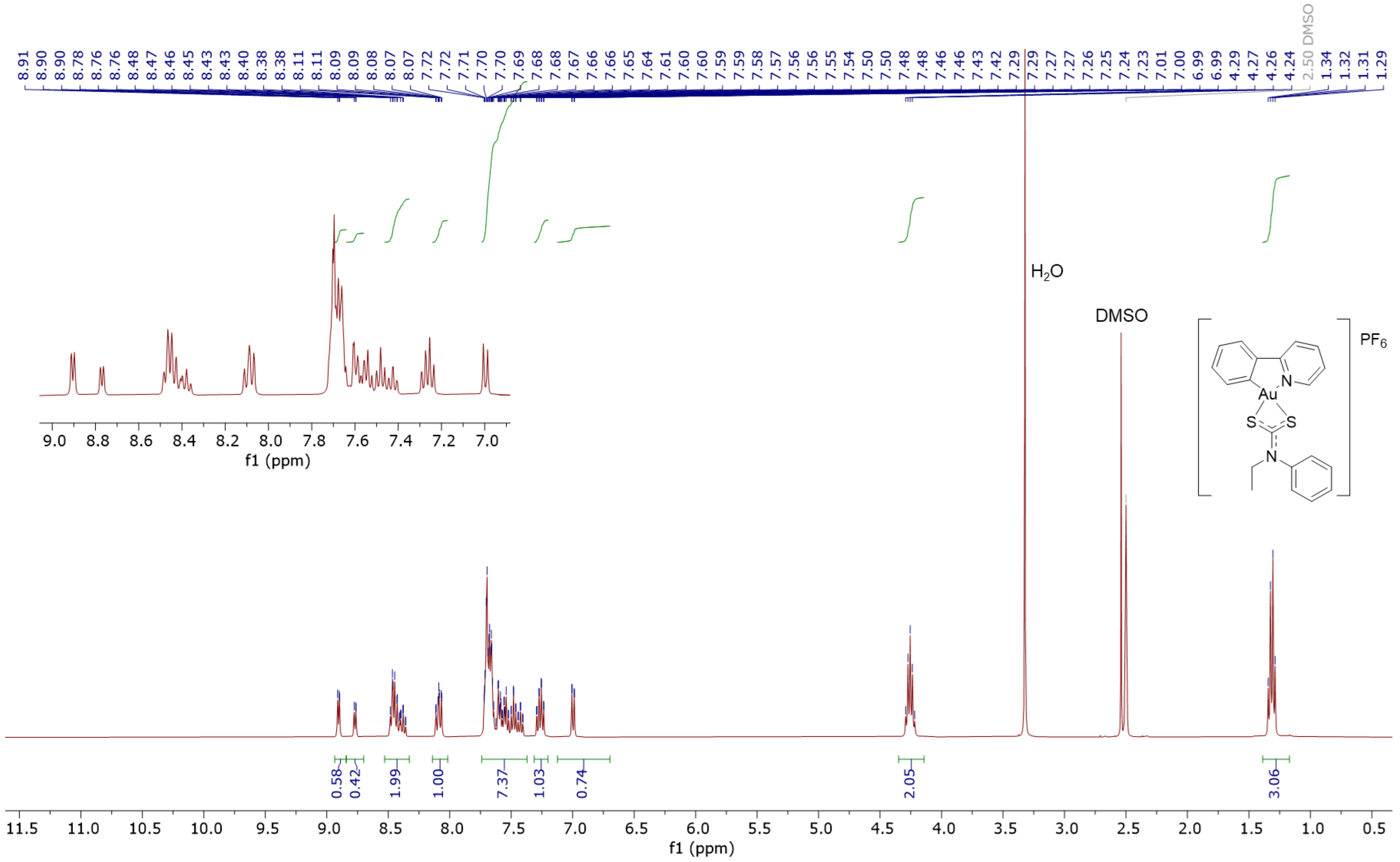

**Supplementary Figure 37**. $^{1}$H NMR of **1E** in DMSO-d$^{6}$.

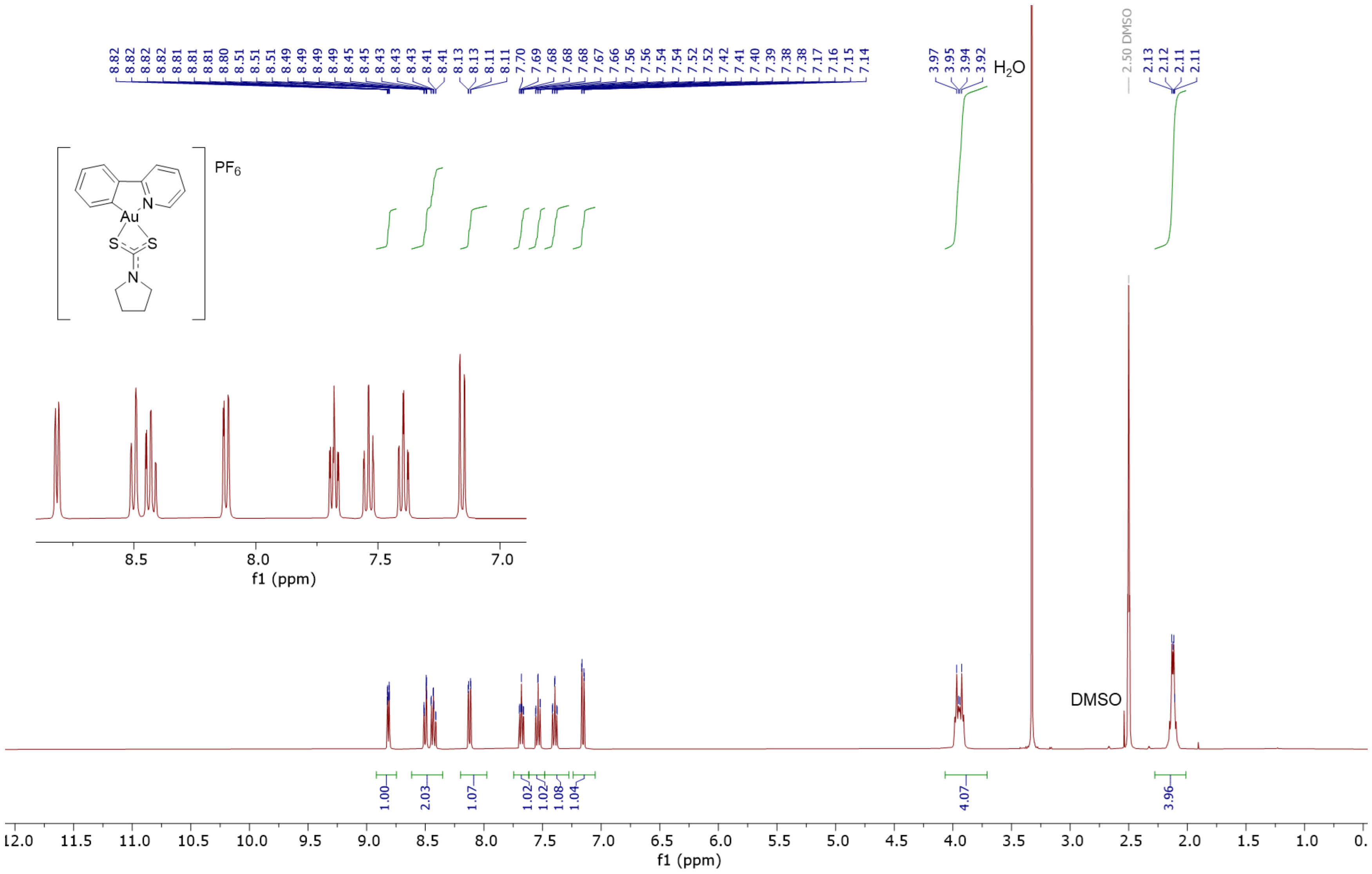

**Supplementary Figure 38**. $^{1}H$ NMR of **1F** in DMSO-d[6].

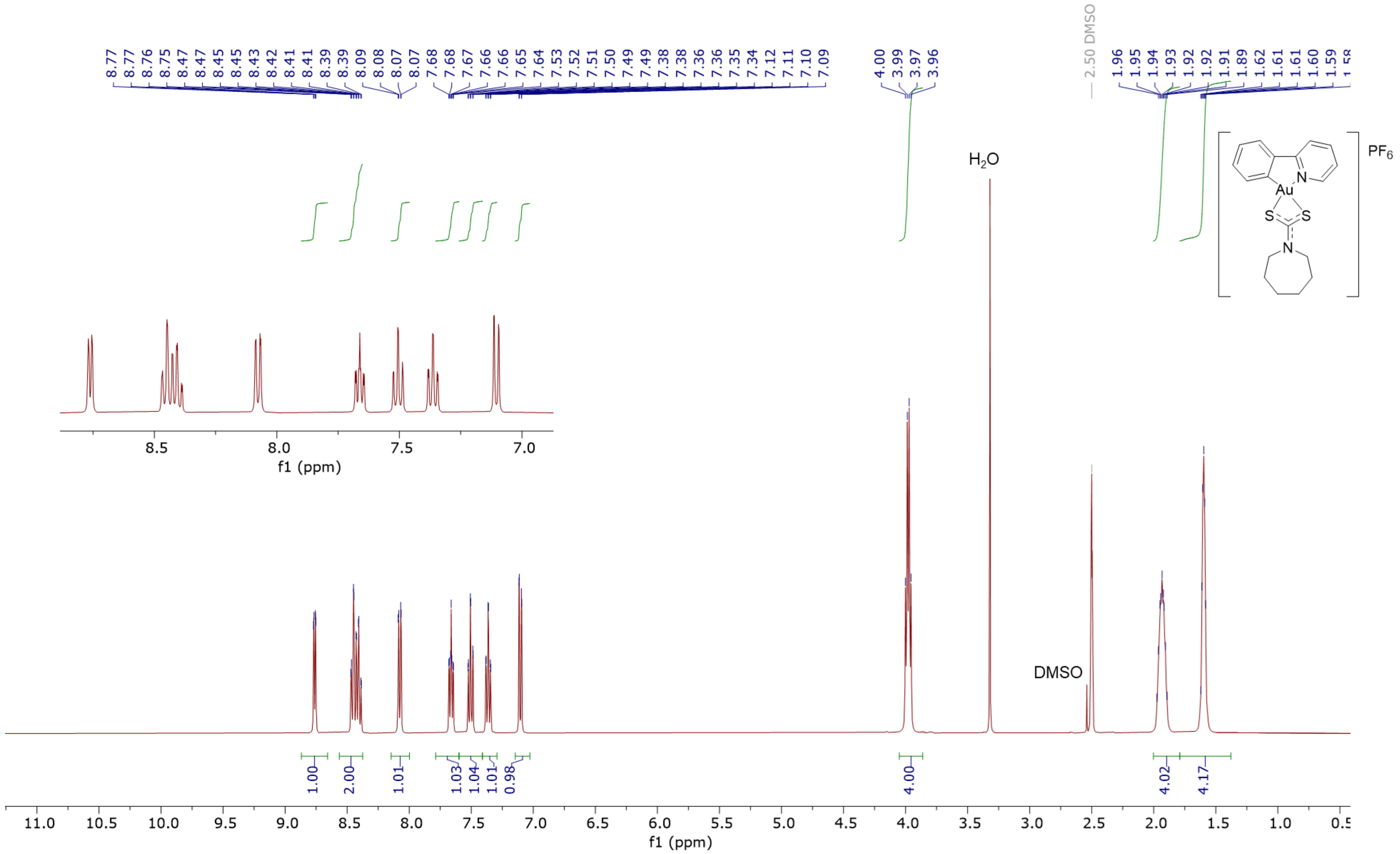

**Supplementary Figure 39**. $^{1}H$ NMR of **1G** in DMSO-d$^{6}$.

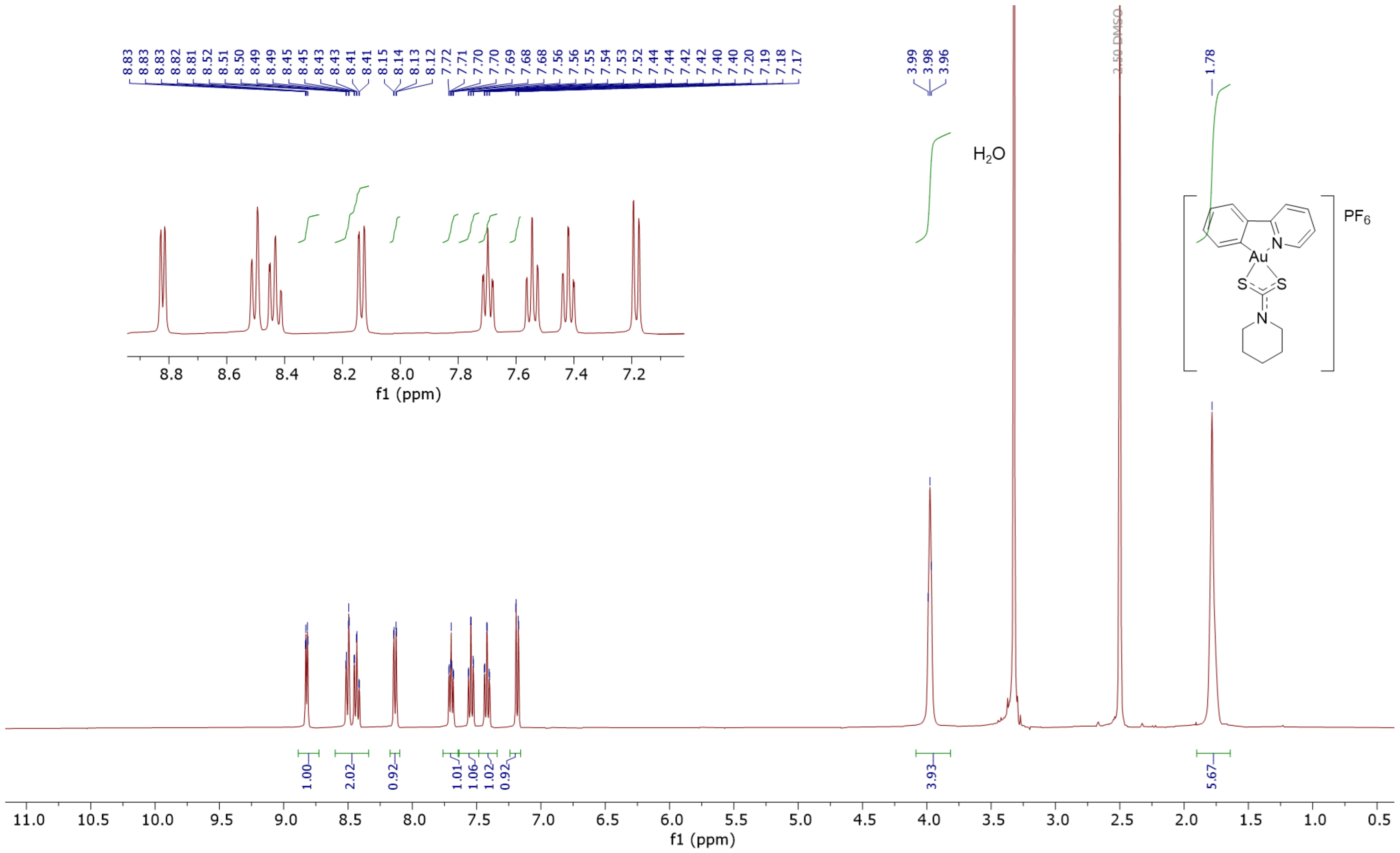


**Supplementary Figure 40**. $^{1}$H NMR of **1H** in DMSO-d$^{6}$.

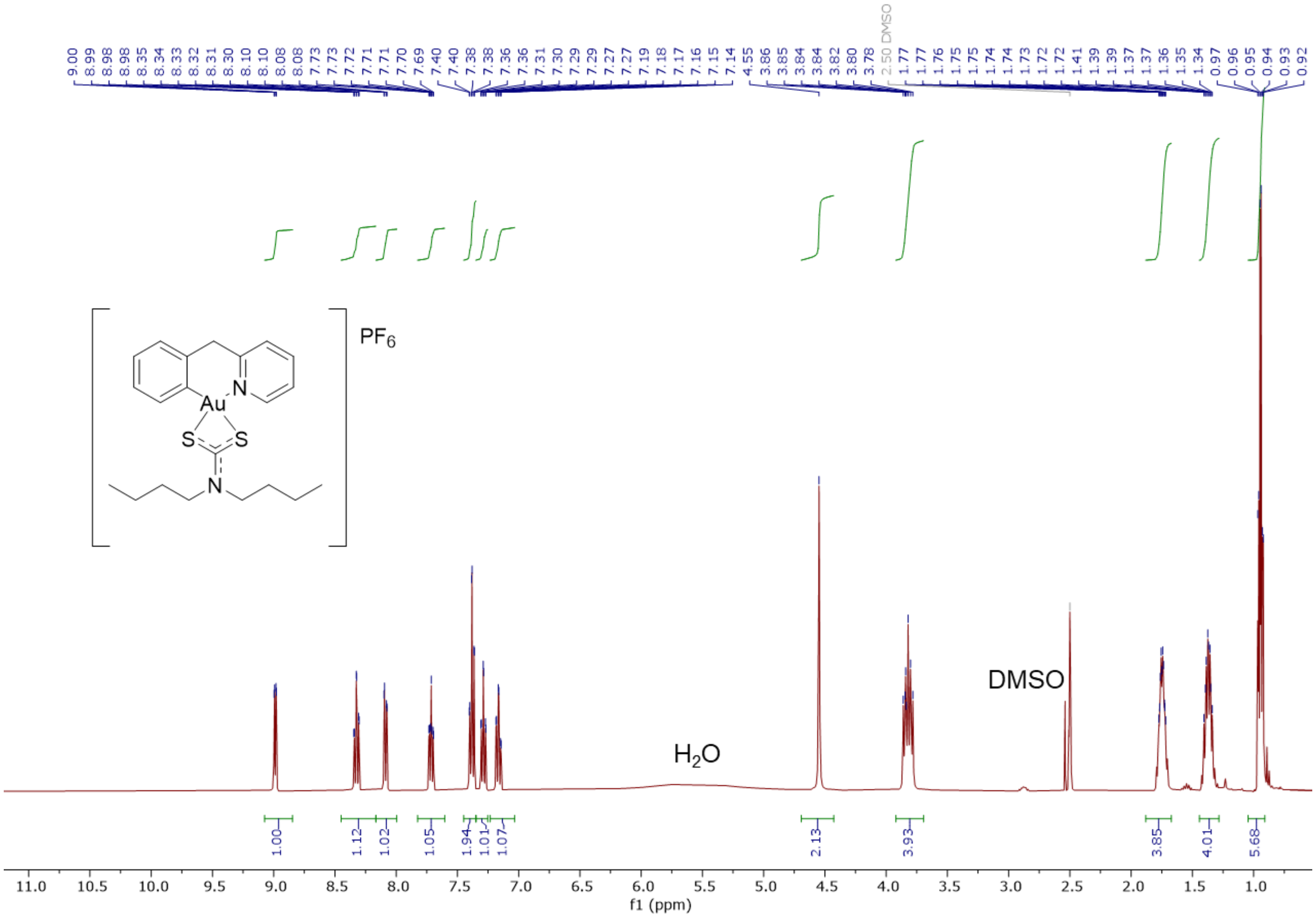


**Supplementary Figure 41**. $^{1}H$ NMR of **2C** in DMSO-d[6].

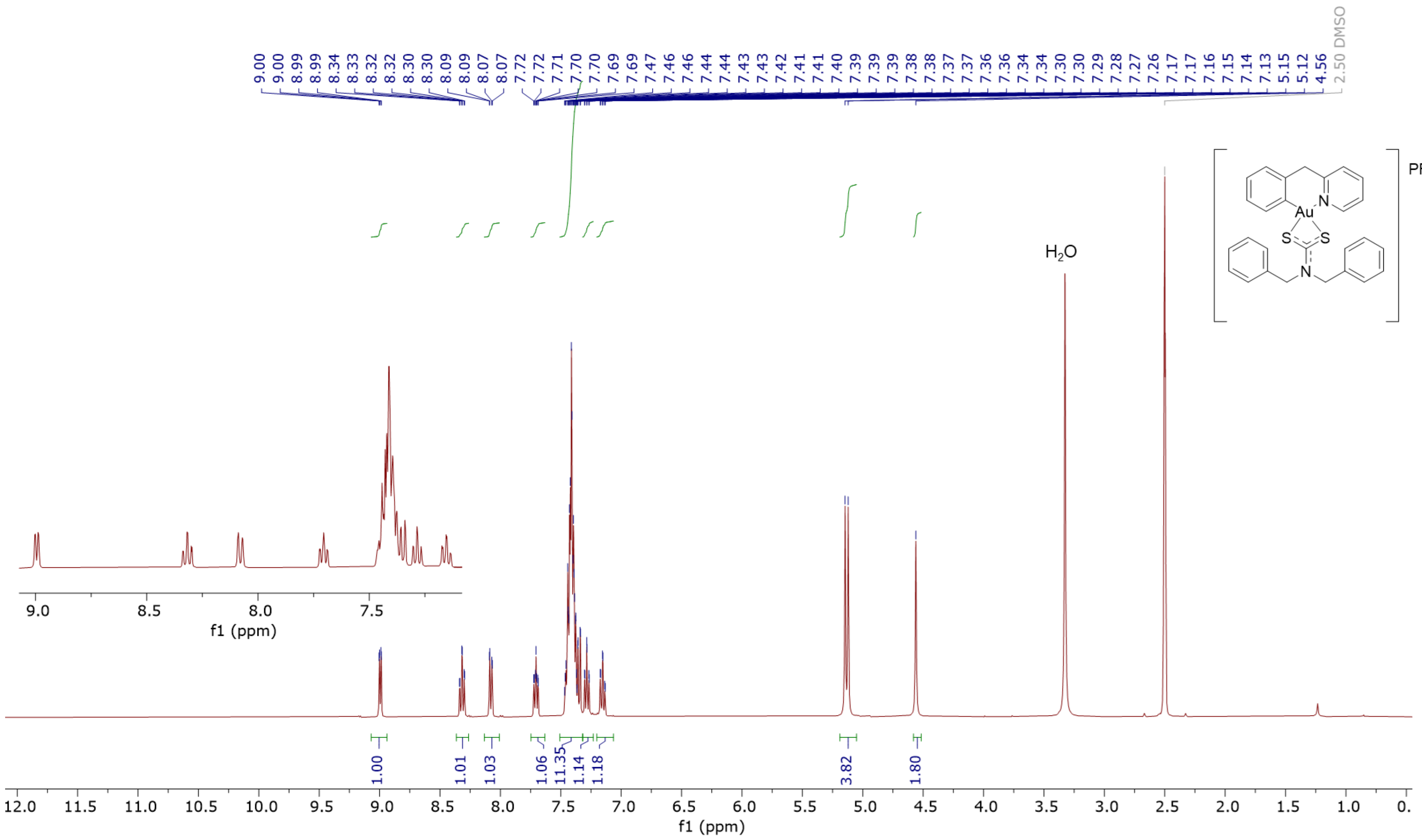

**Supplementary Figure 42**. $^{1}$H NMR of **2D** in DMSO-d[6].

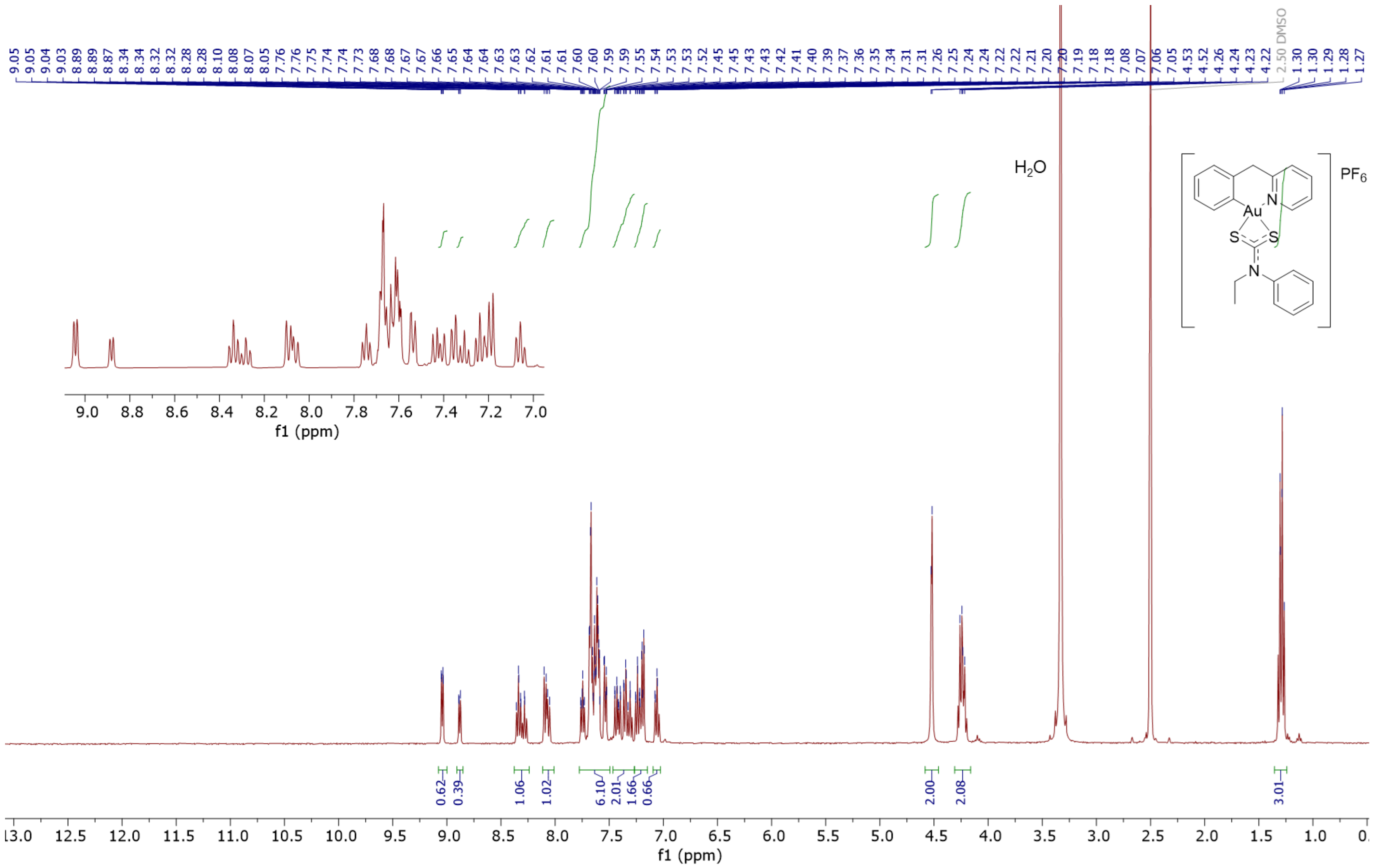


**Supplementary Figure 43**. $^1$H NMR of **2E** in DMSO-d$^6$.

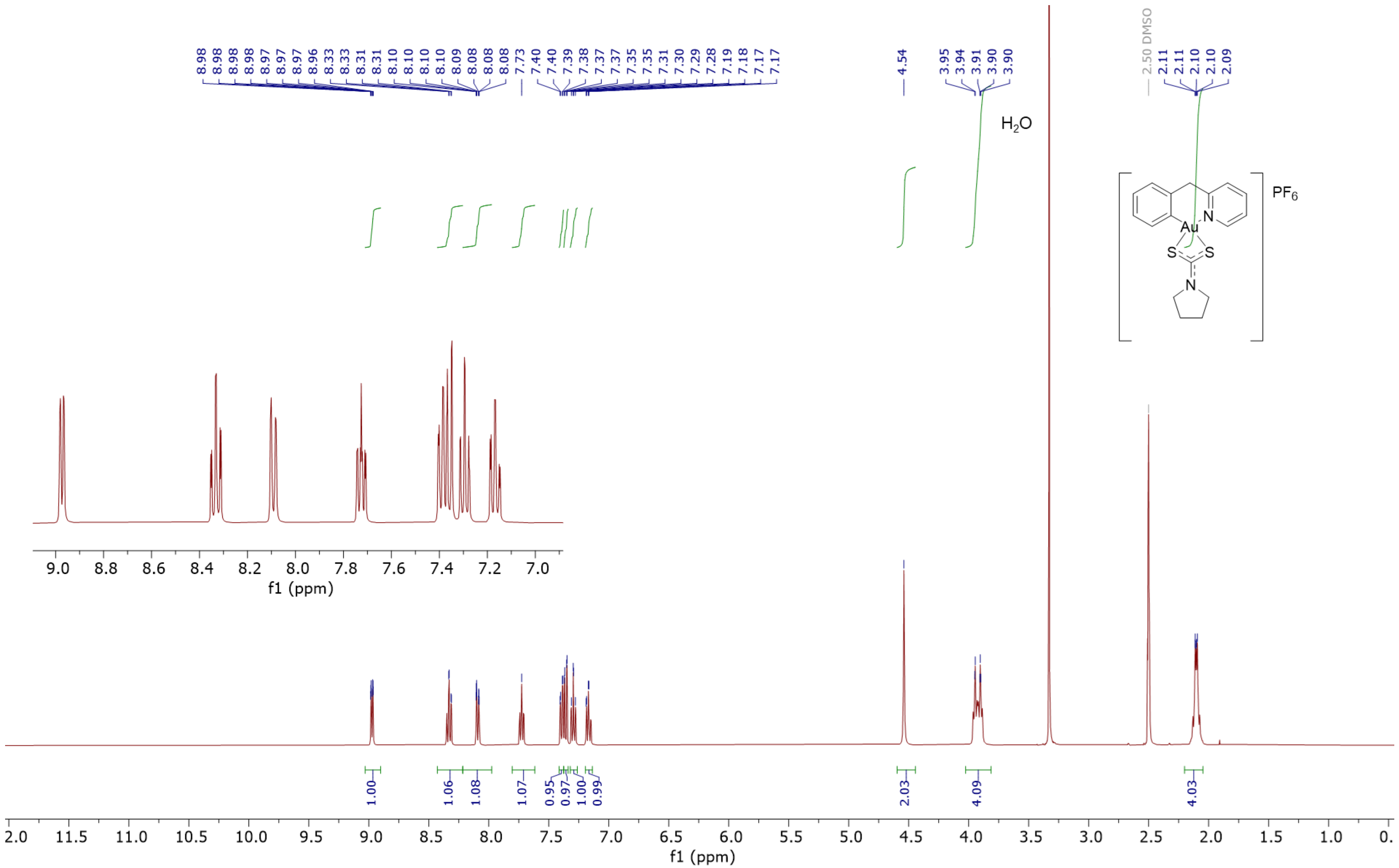


**Supplementary Figure 44**. $^{1}$H NMR of **2F** in DMSO-d$^{6}$.

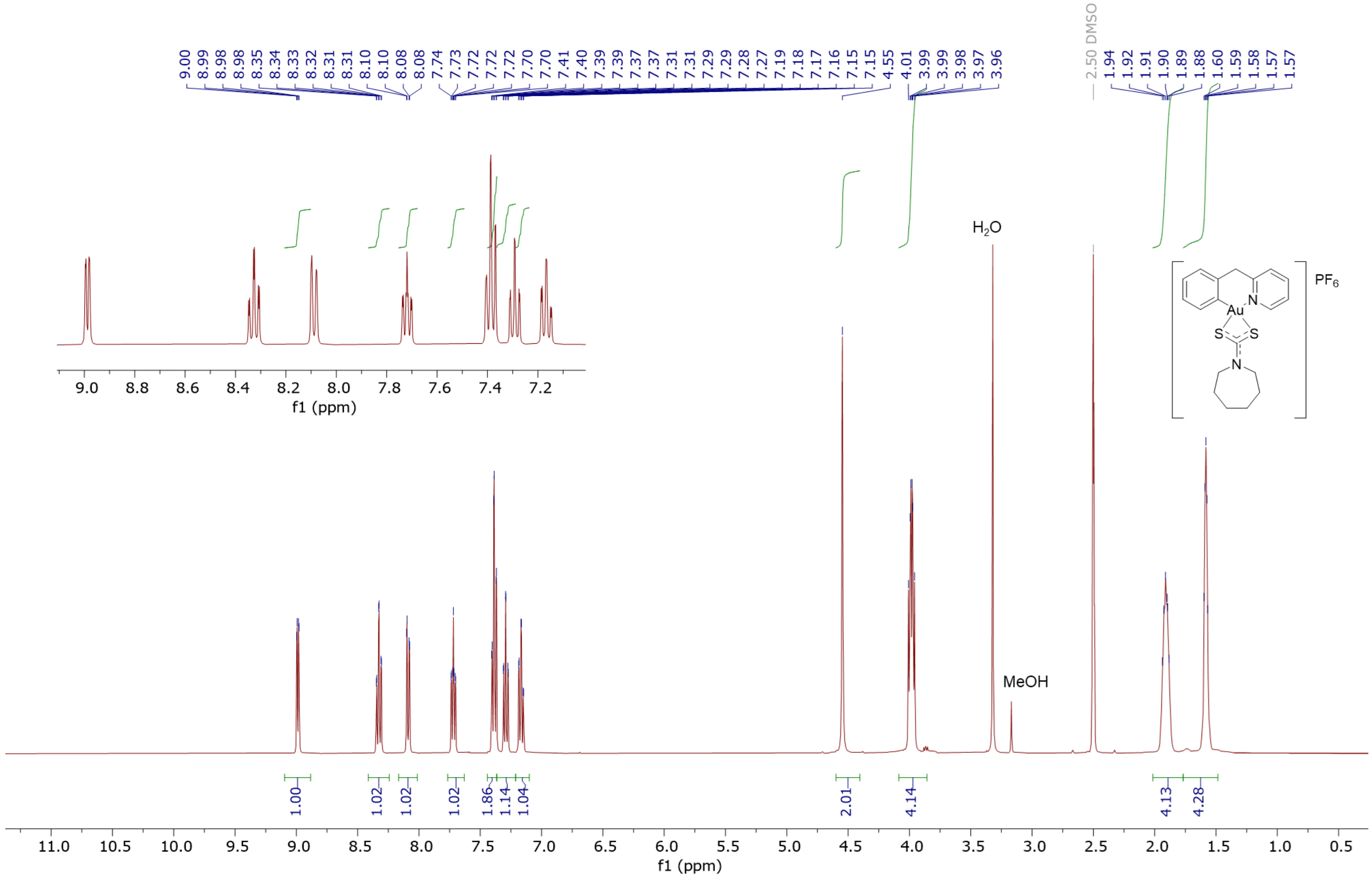


**Supplementary Figure 45**. $^{1}$H NMR of **2G** in DMSO-d$^{6}$.

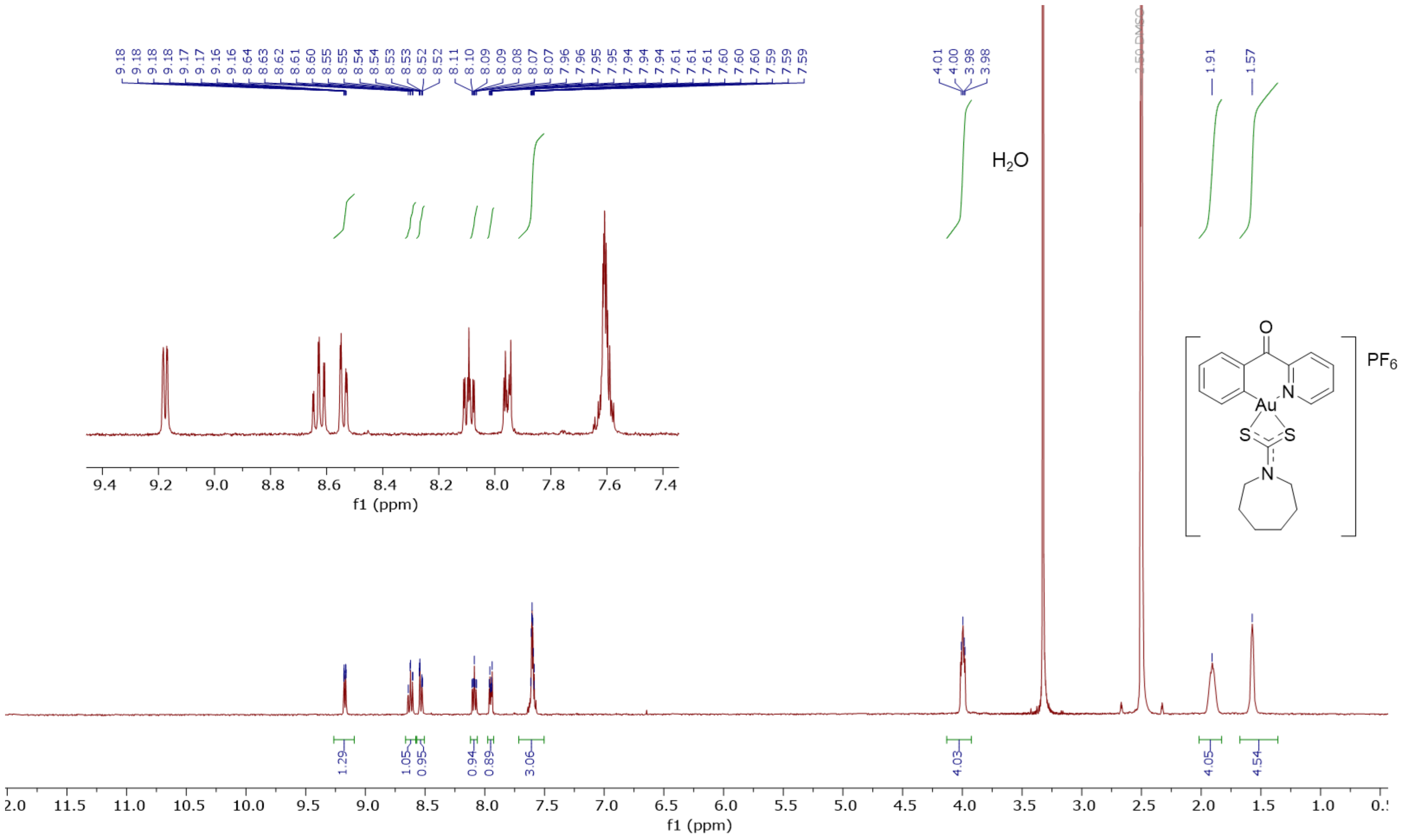

**Supplementary Figure 46**. $^{1}H$ NMR of **3G** in DMSO-d[6].

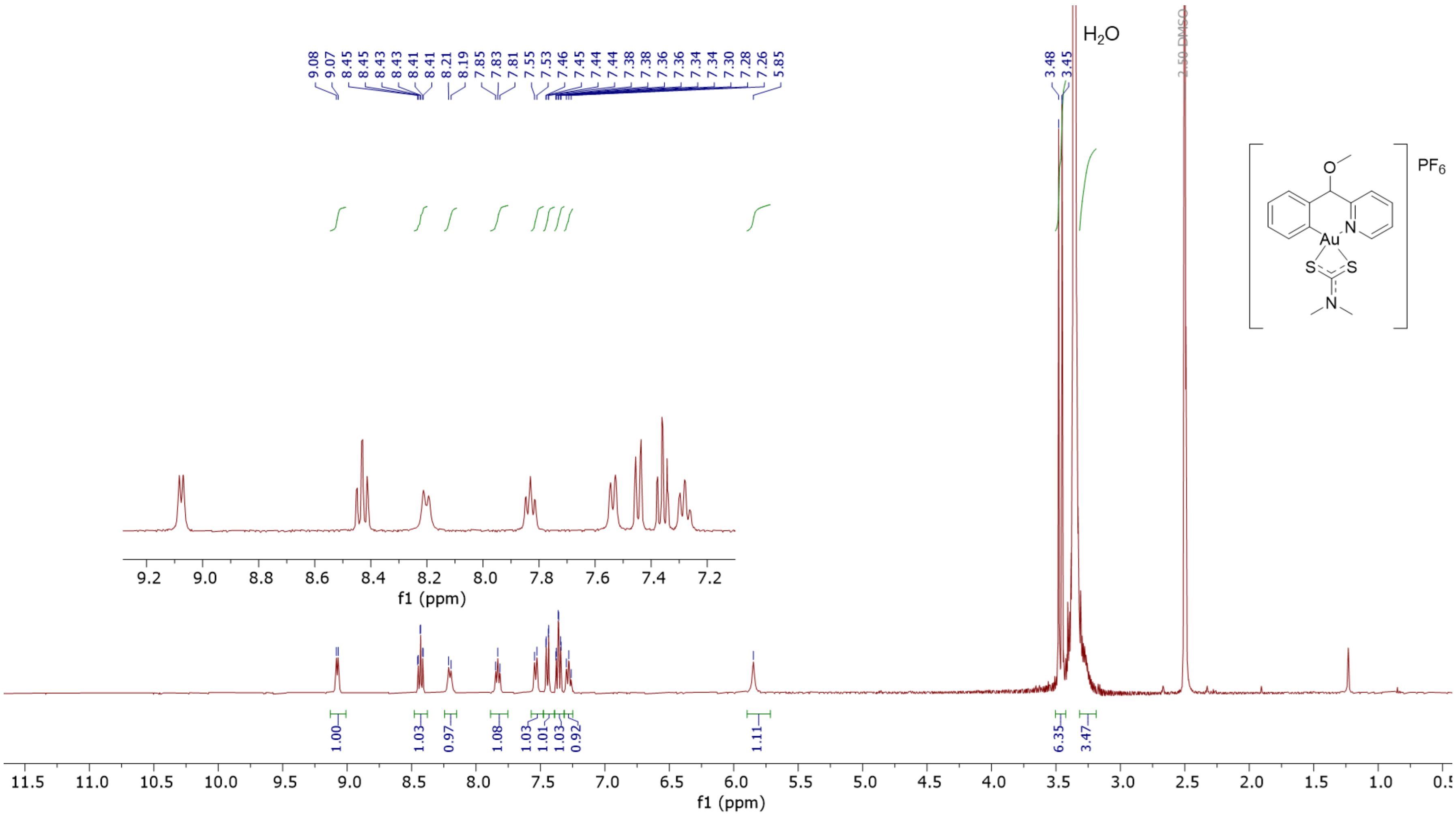

**Supplementary Figure 47**. $^{1}H$ NMR of **4A** in DMSO-d$^{6}$.

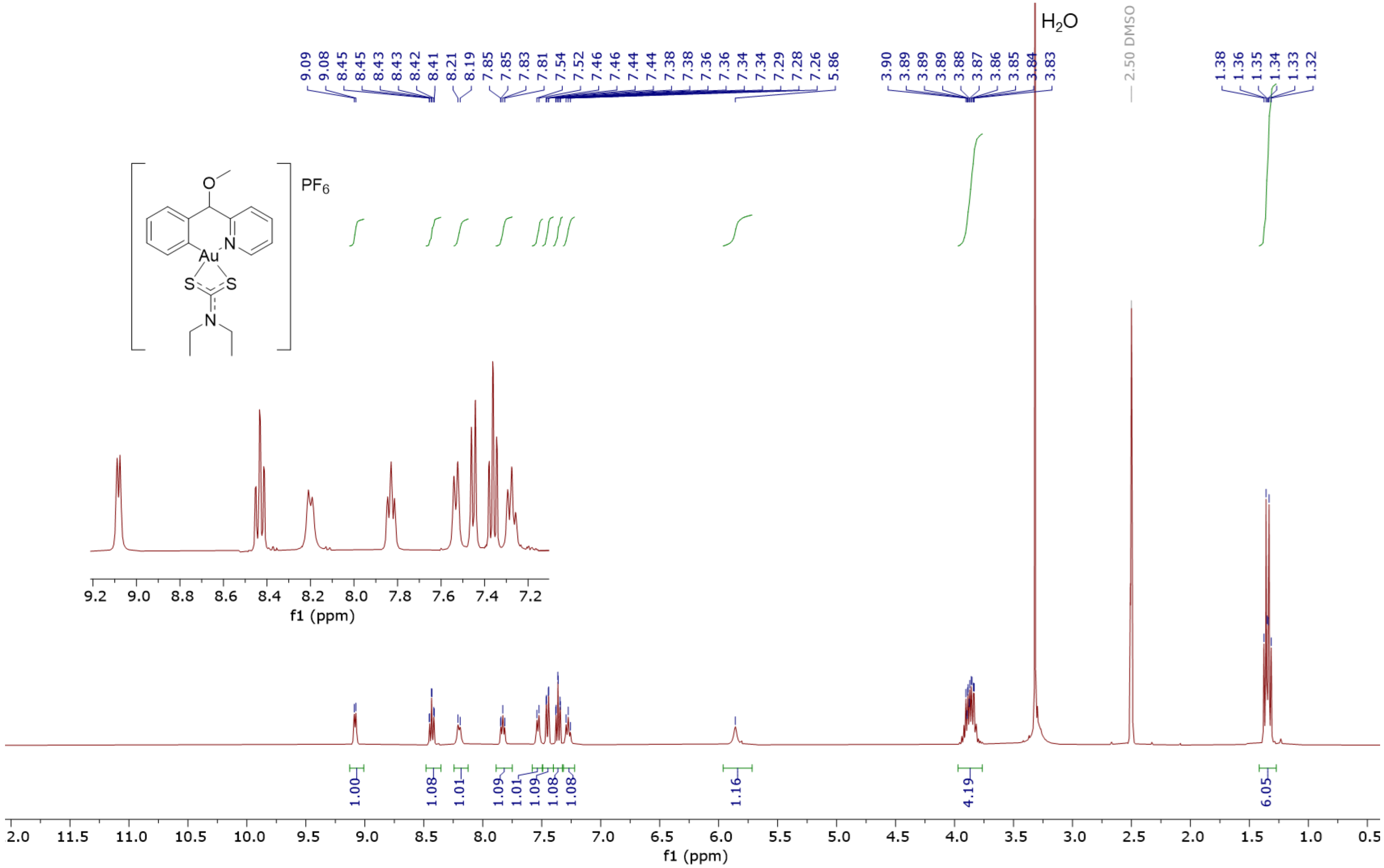

**Supplementary Figure 48**. $^1$H NMR of **4B** in DMSO-d$^6$.

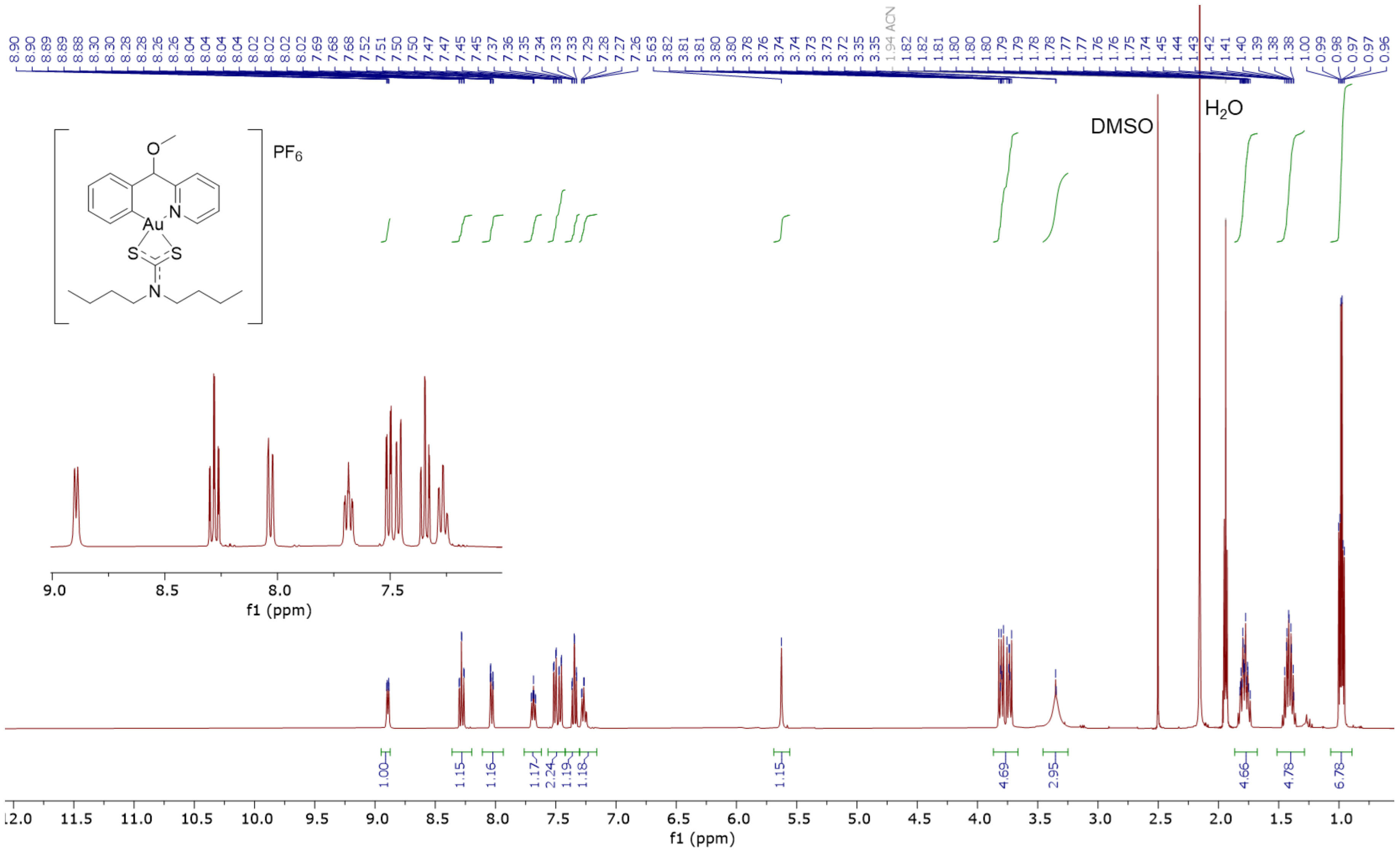


**Supplementary Figure 49**. $^1$H NMR of **4C** in ACN-d$^3$.

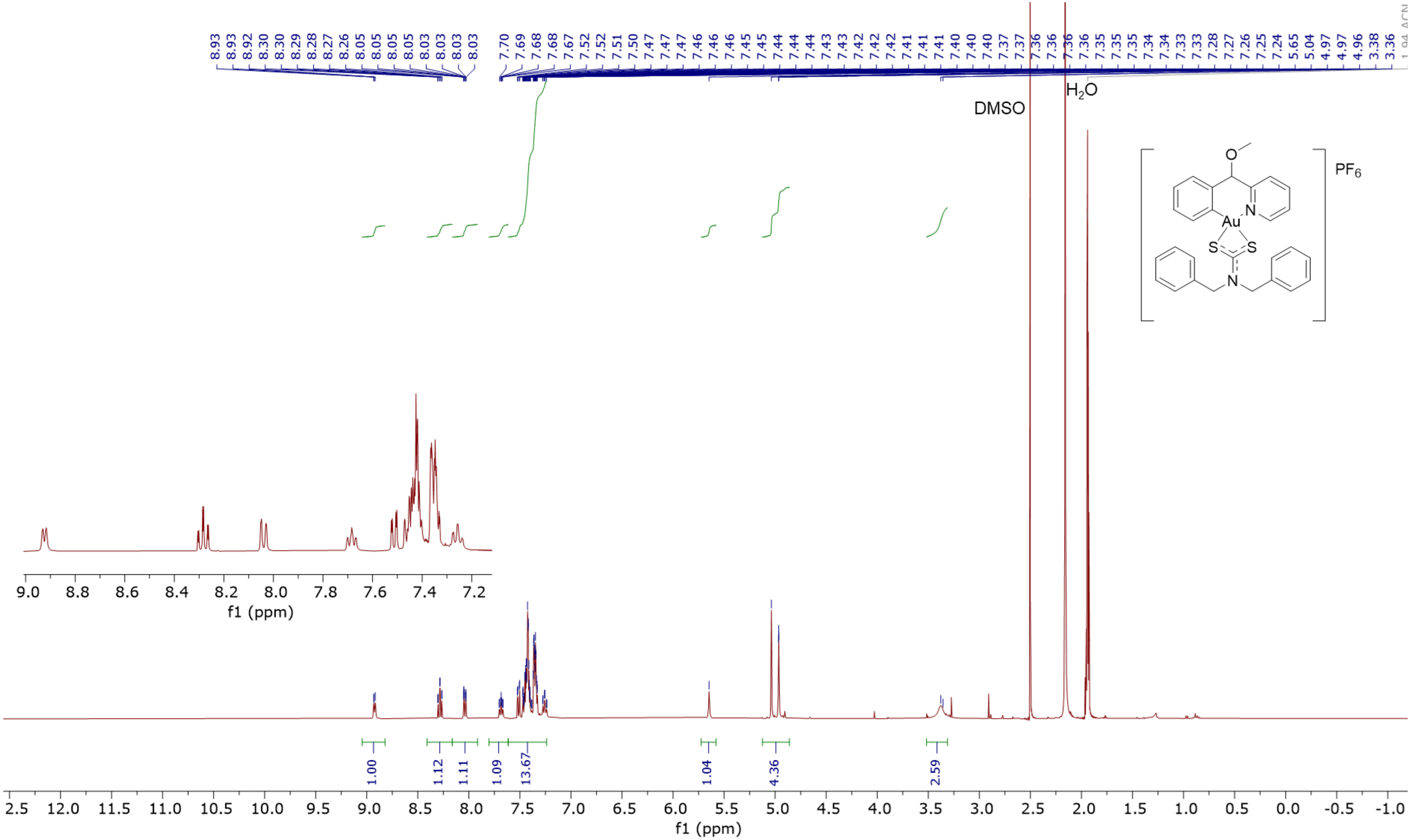


**Supplementary Figure 50**. $^1$H NMR of **4D** in ACN-d$^3$.

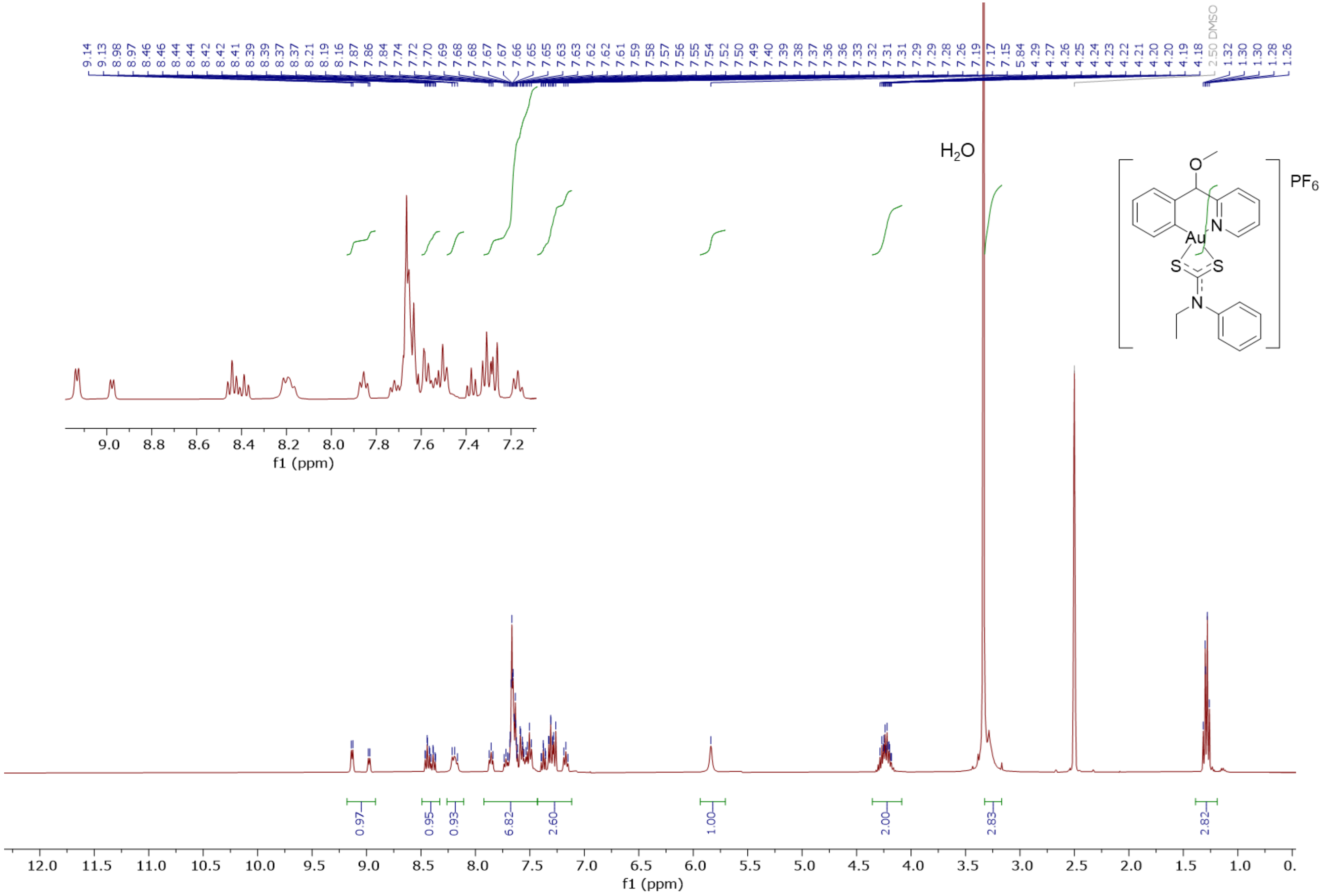

**Supplementary Figure 51**. $^1H$ NMR of **4E** in DMSO-d$^6$.

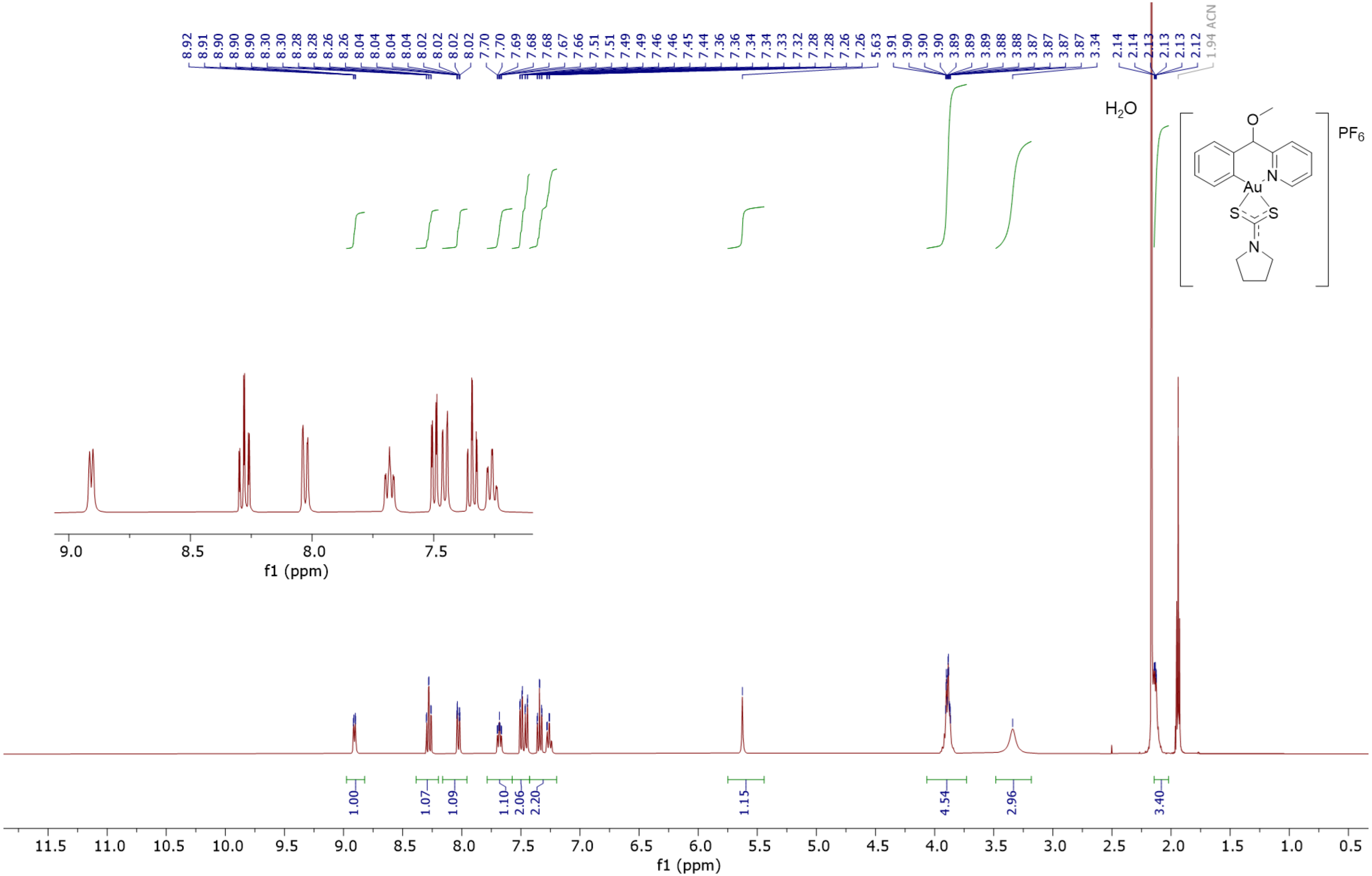

**Supplementary Figure 52**. $^1H$ NMR of **4F** in ACN-$d^3$.

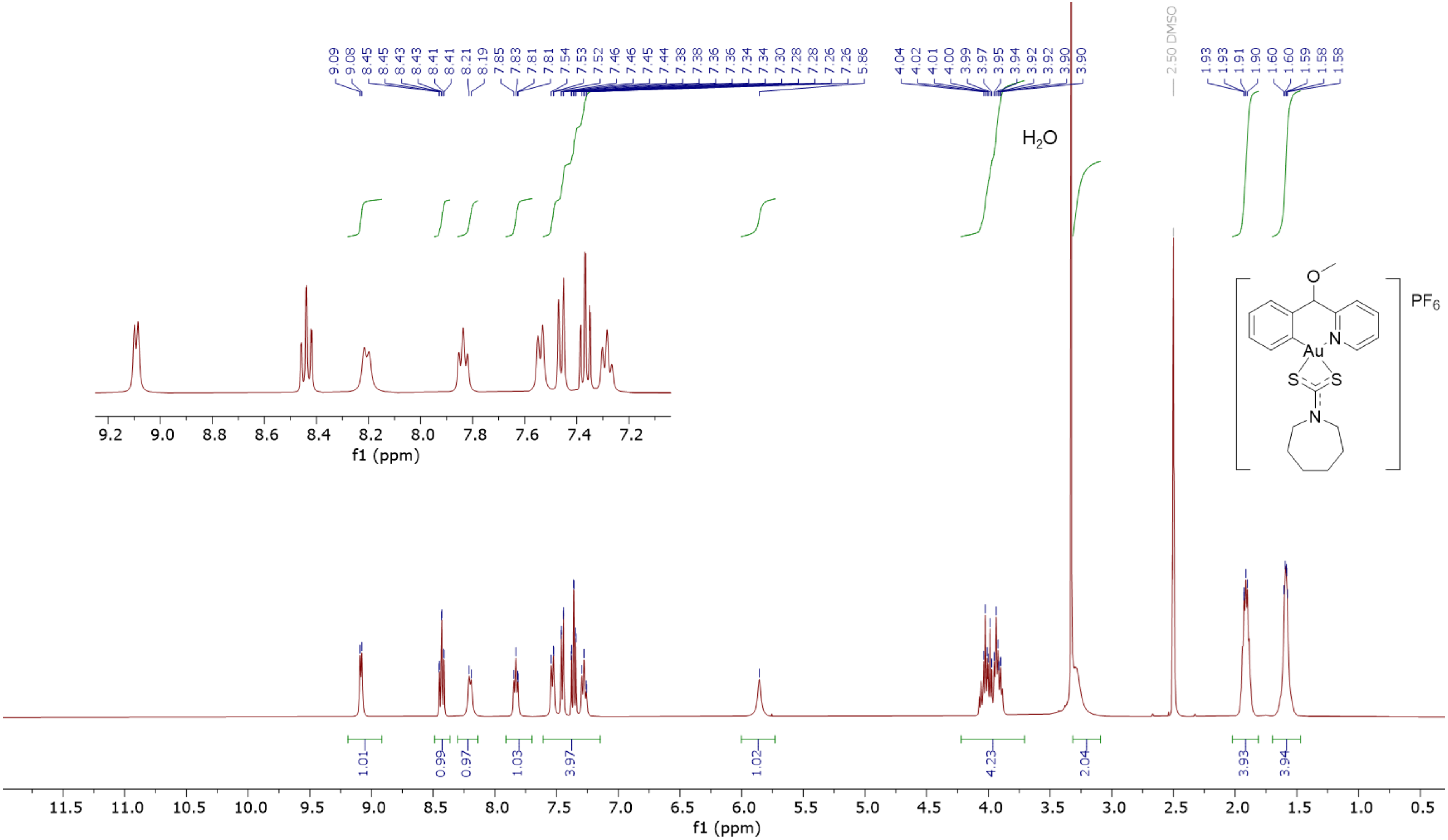


**Supplementary Figure 53**. $^{1}$H NMR of **4G** in DMSO-d$^{6}$.

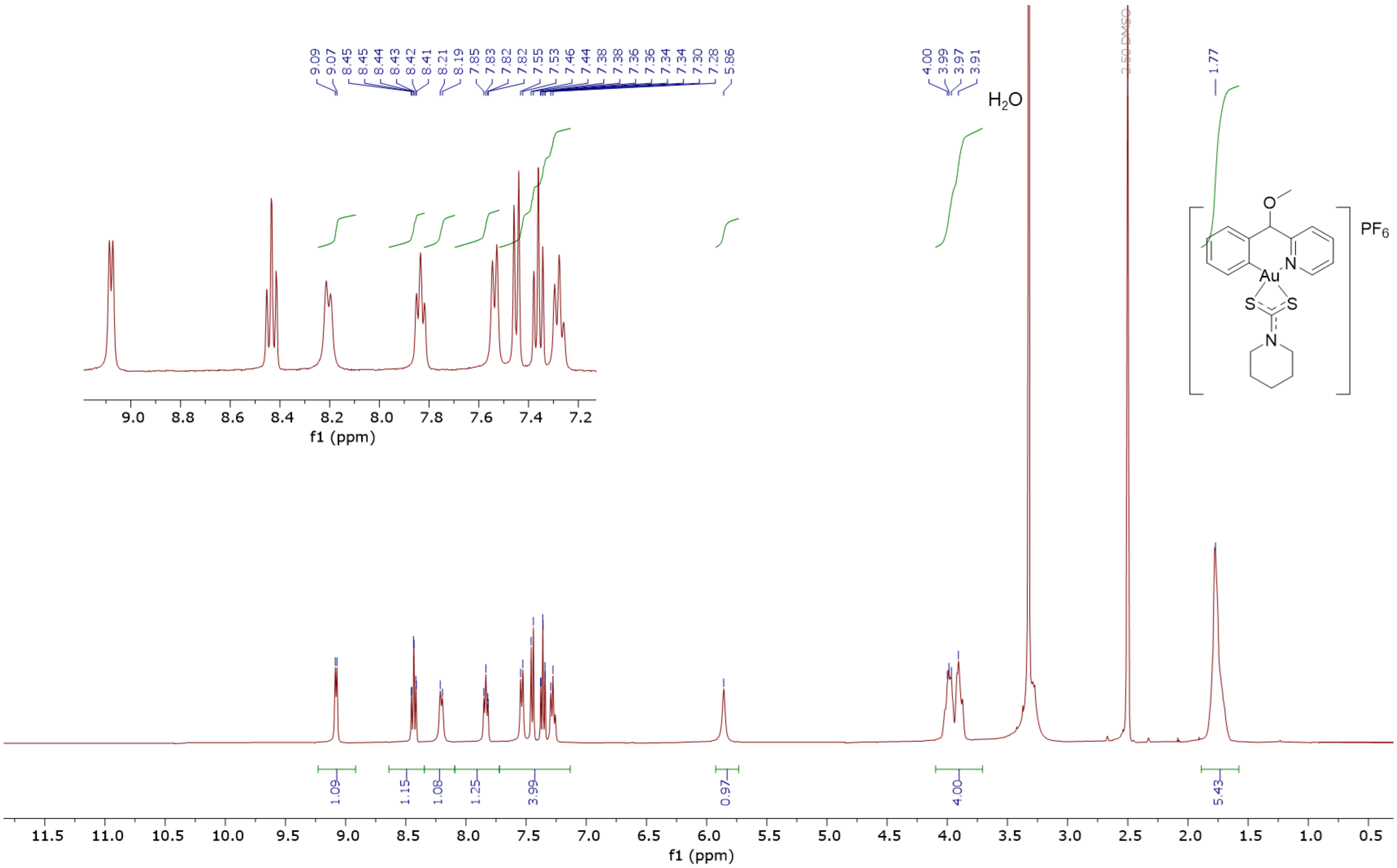


**Supplementary Figure 54**. $^1$H NMR of **4H** in DMSO-d$^6$.

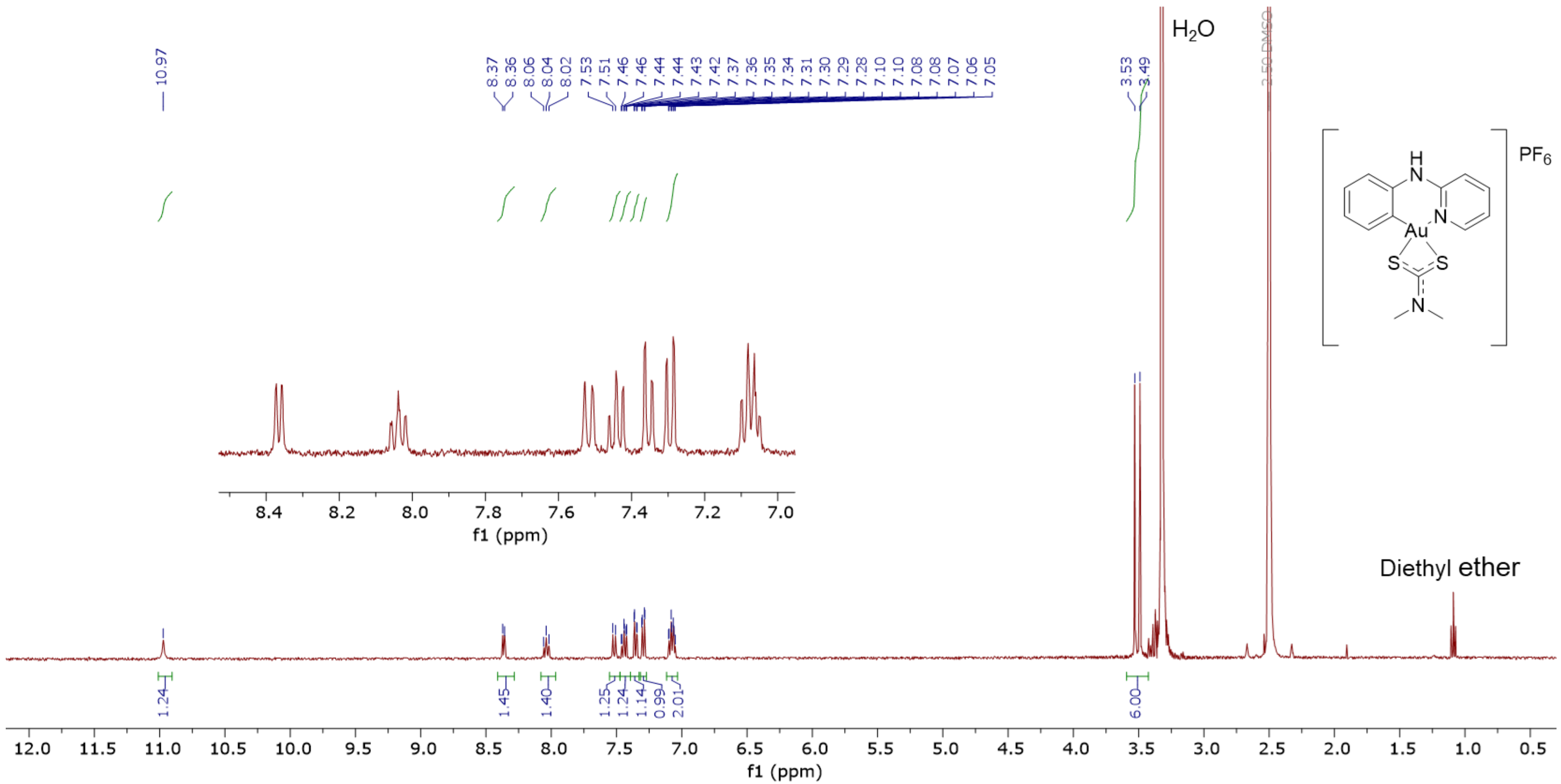


**Supplementary Figure 55**. $^{1}$H NMR of **5A** in DMSO-d$^{6}$.

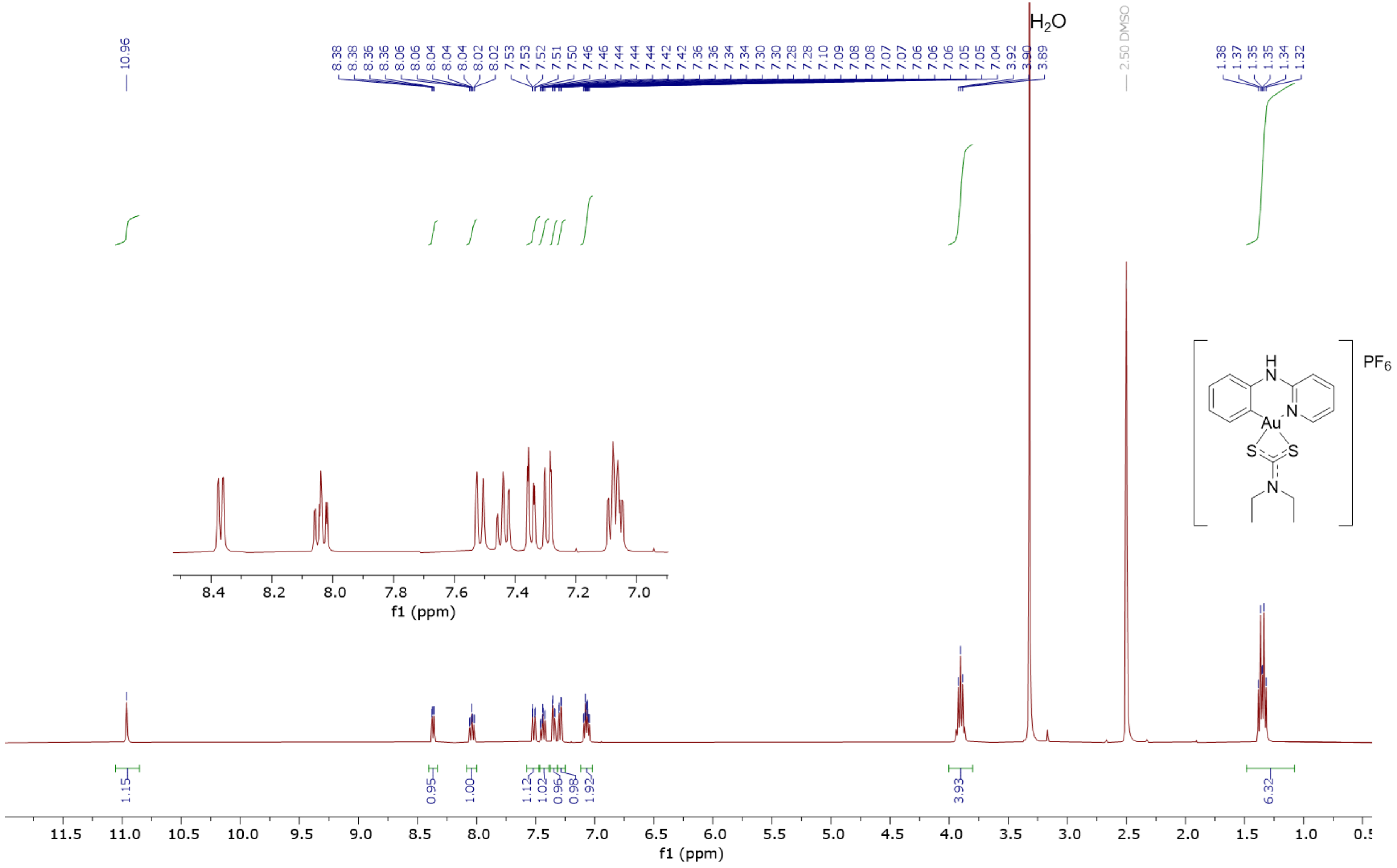


**Supplementary Figure 56**. $^{1}$H NMR of **5B** in DMSO-d$^{6}$.

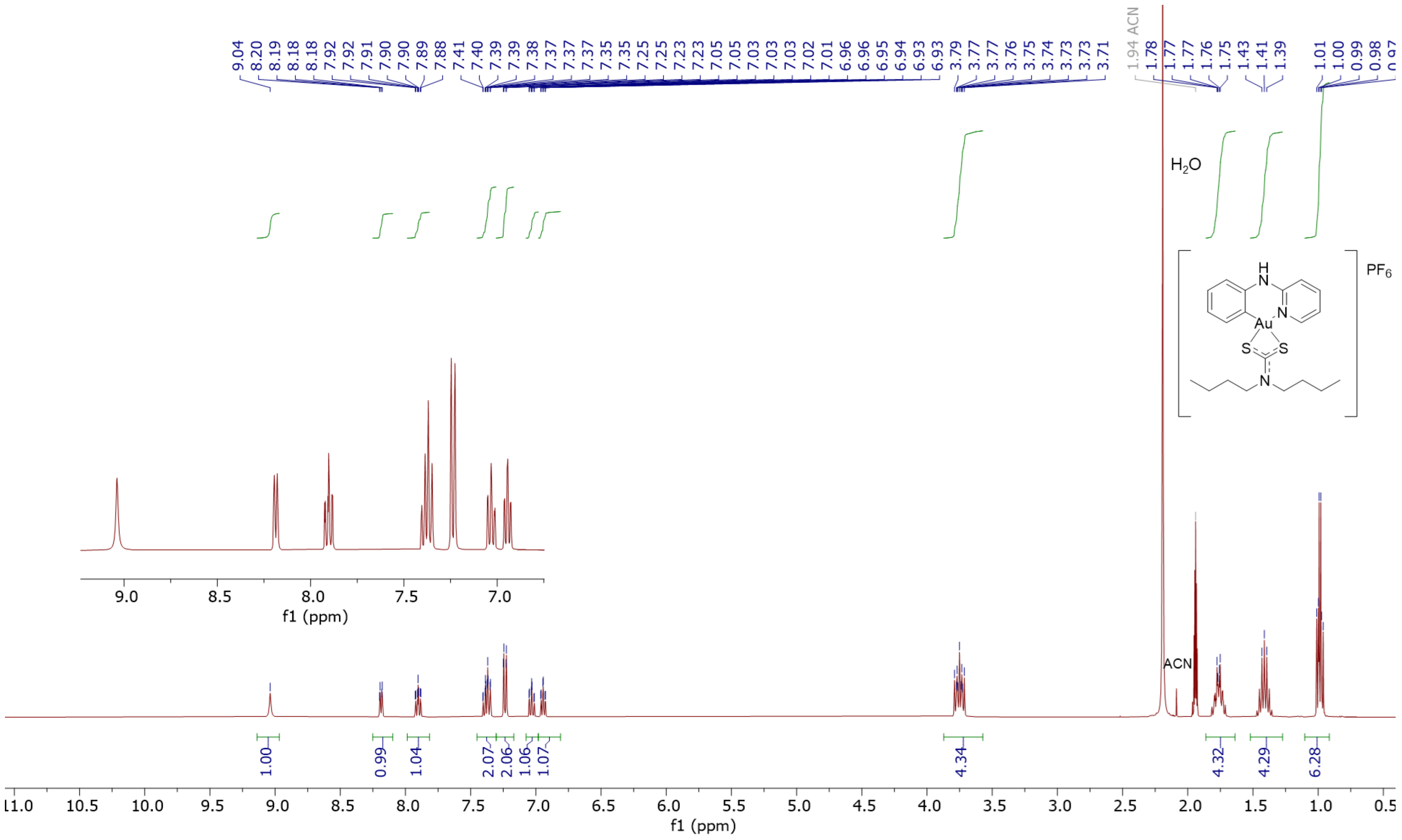


**Supplementary Figure 57**. $^{1}$H NMR of **5C** in ACN-d$^3$.

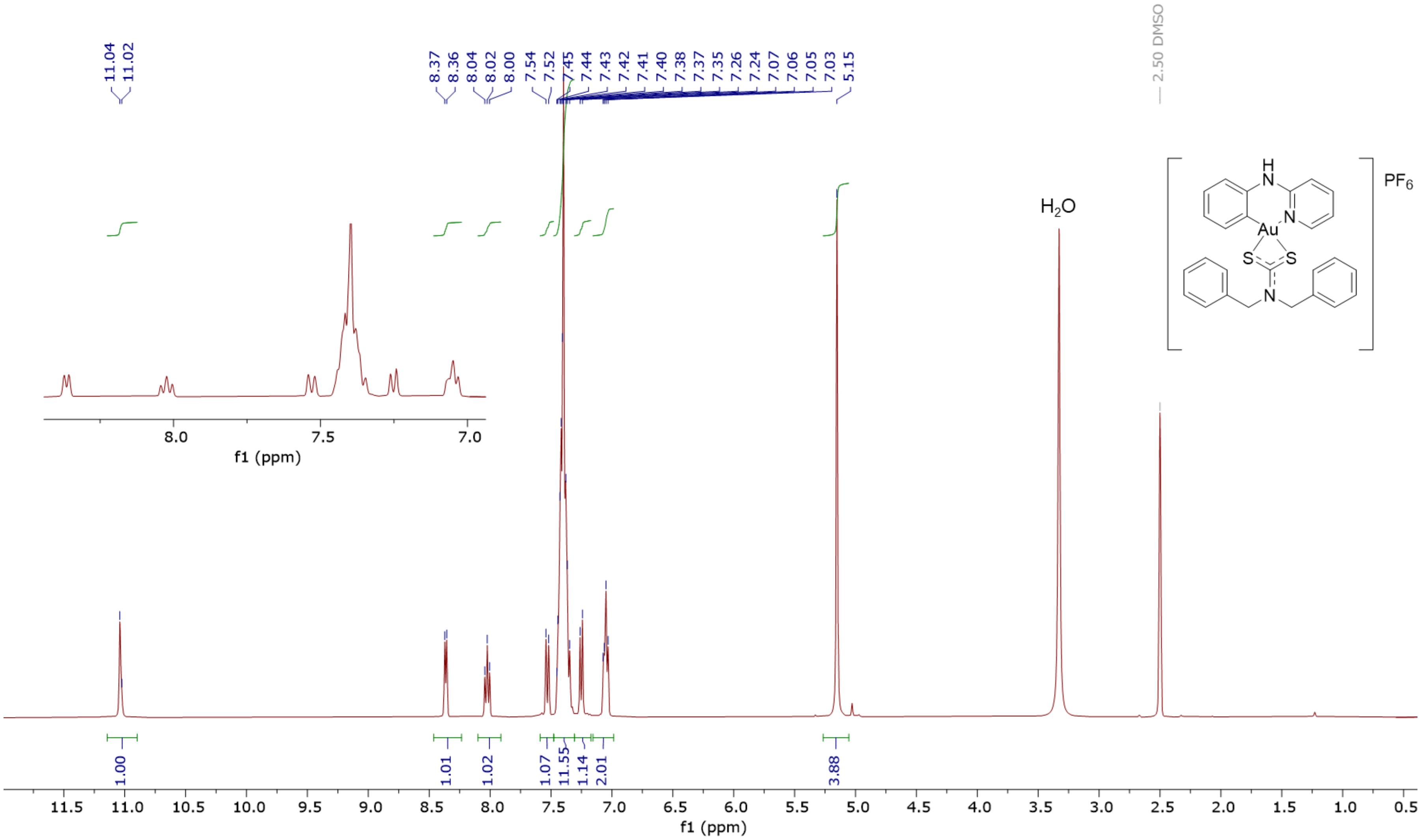


**Supplementary Figure 58**. $^{1}$H NMR of **5D** in DMSO-d[6].

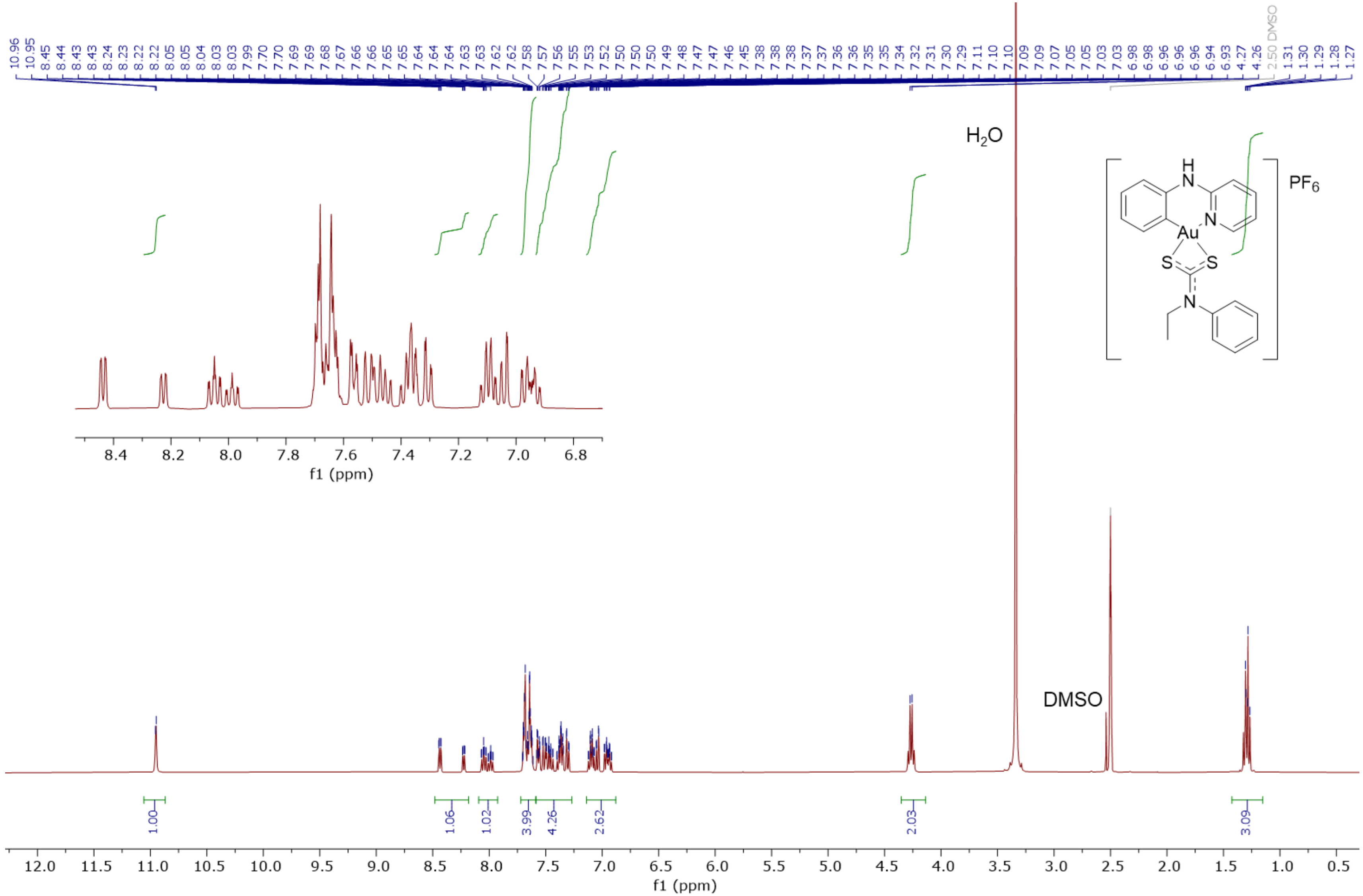


**Supplementary Figure 59**. $^{1}H$ NMR of **5E** in DMSO-$d^{6}$.

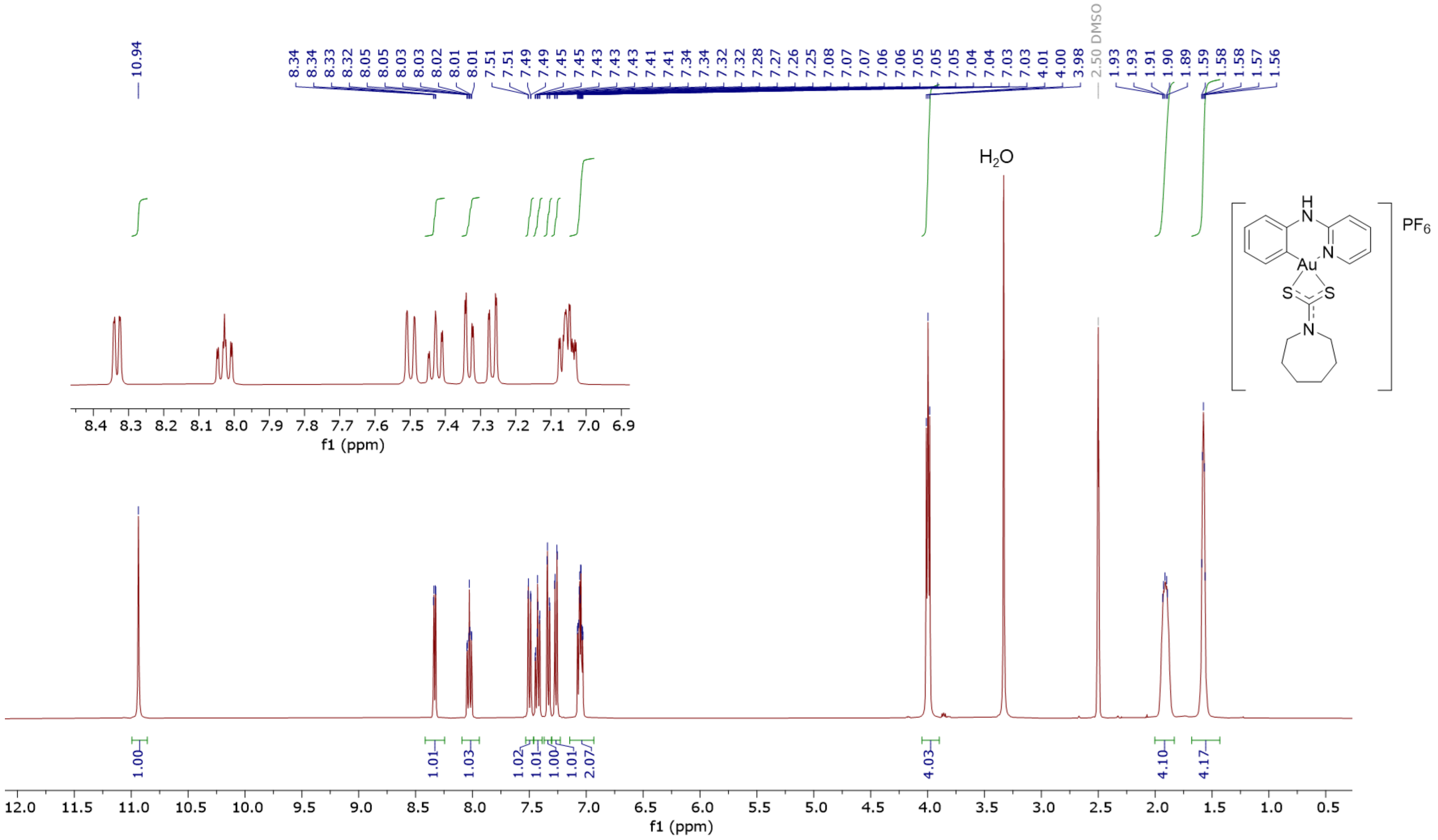


**Supplementary Figure 60**. $^1$H NMR of **5G** in DMSO-d$^6$.

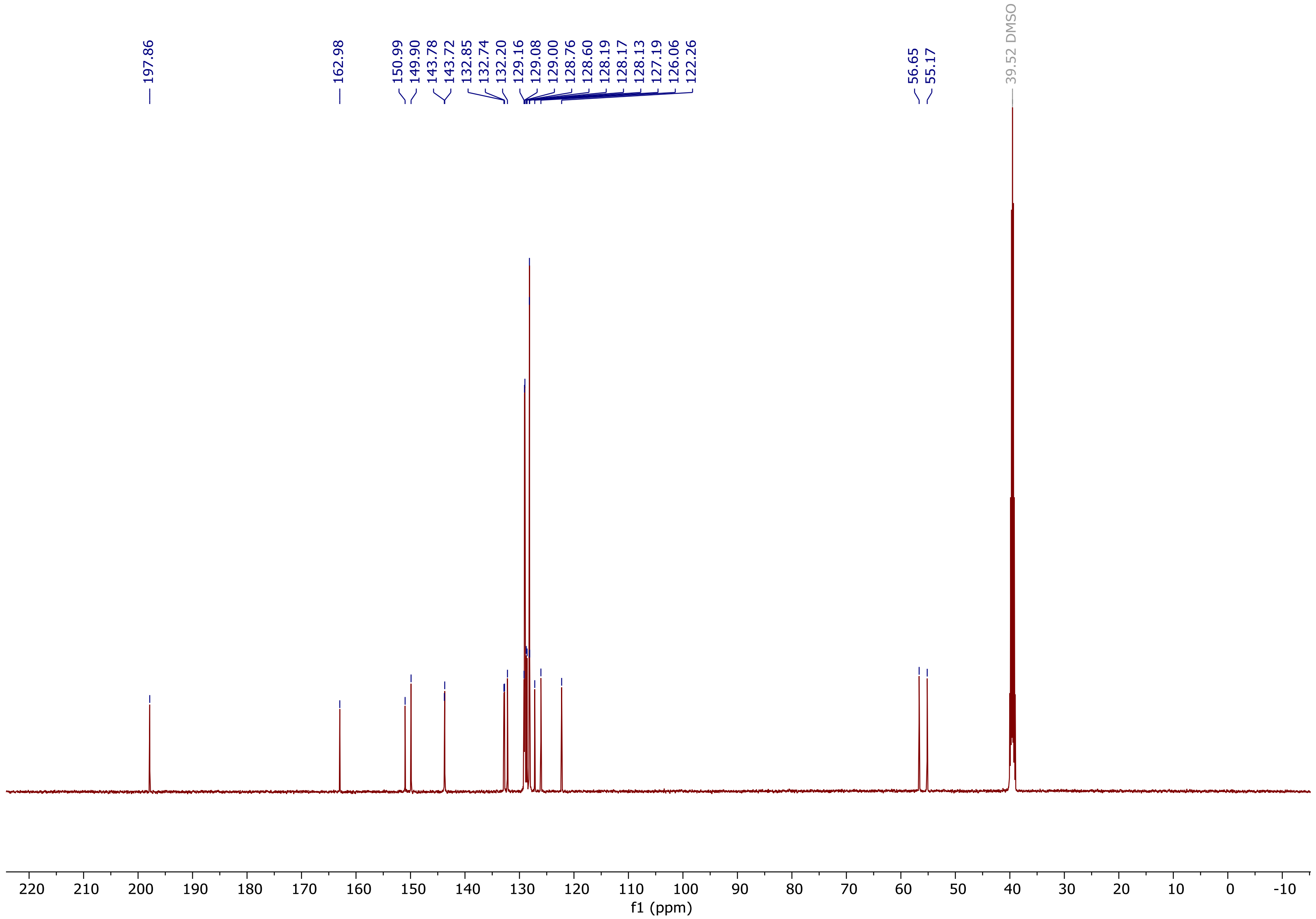


**Supplementary Figure 61.** $^{13}$C NMR of **1D** in DMSO-d$^{6}$.

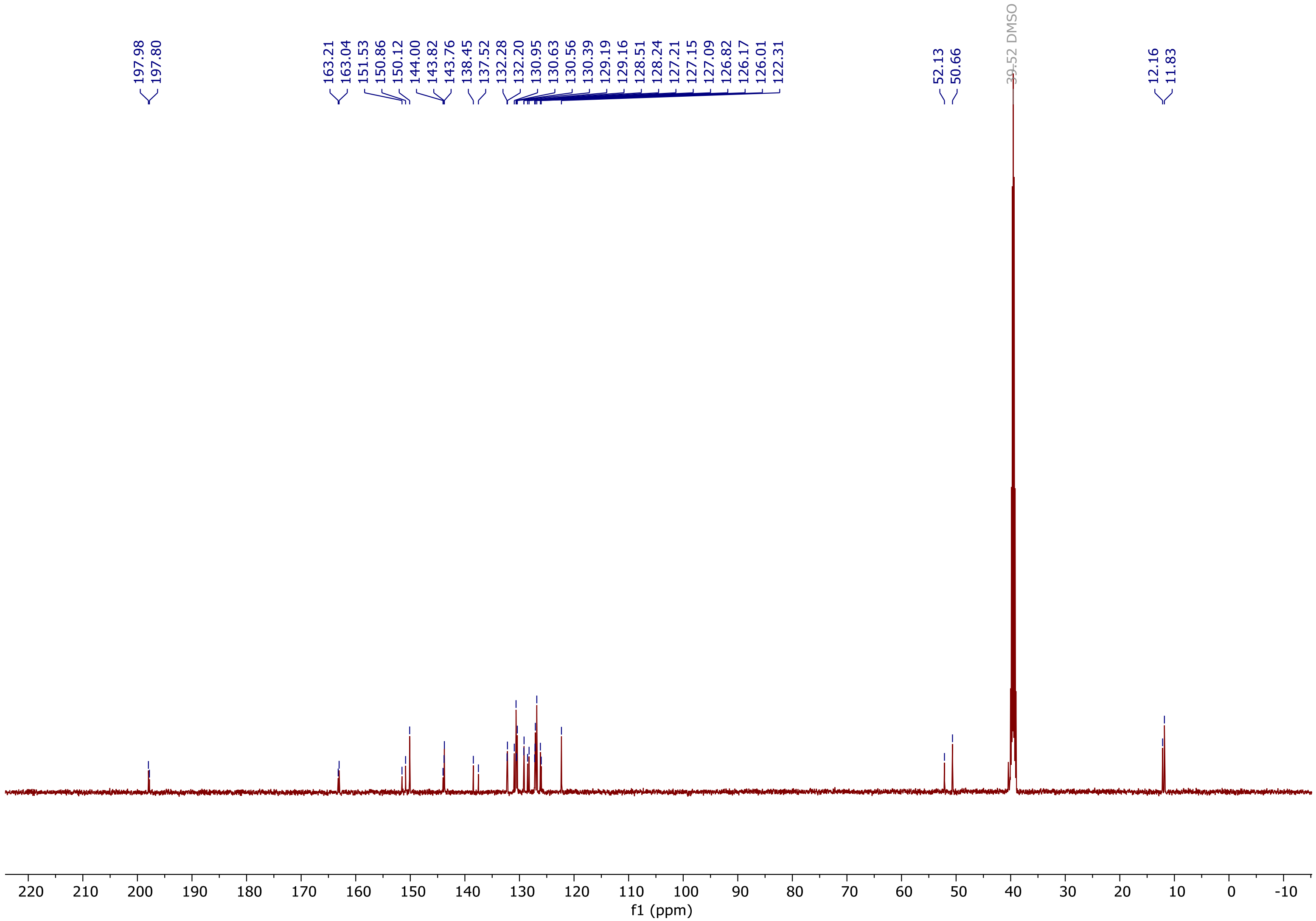


**Supplementary Figure 62.** $^{13}C$ NMR of **1E** in DMSO-$d^6$.

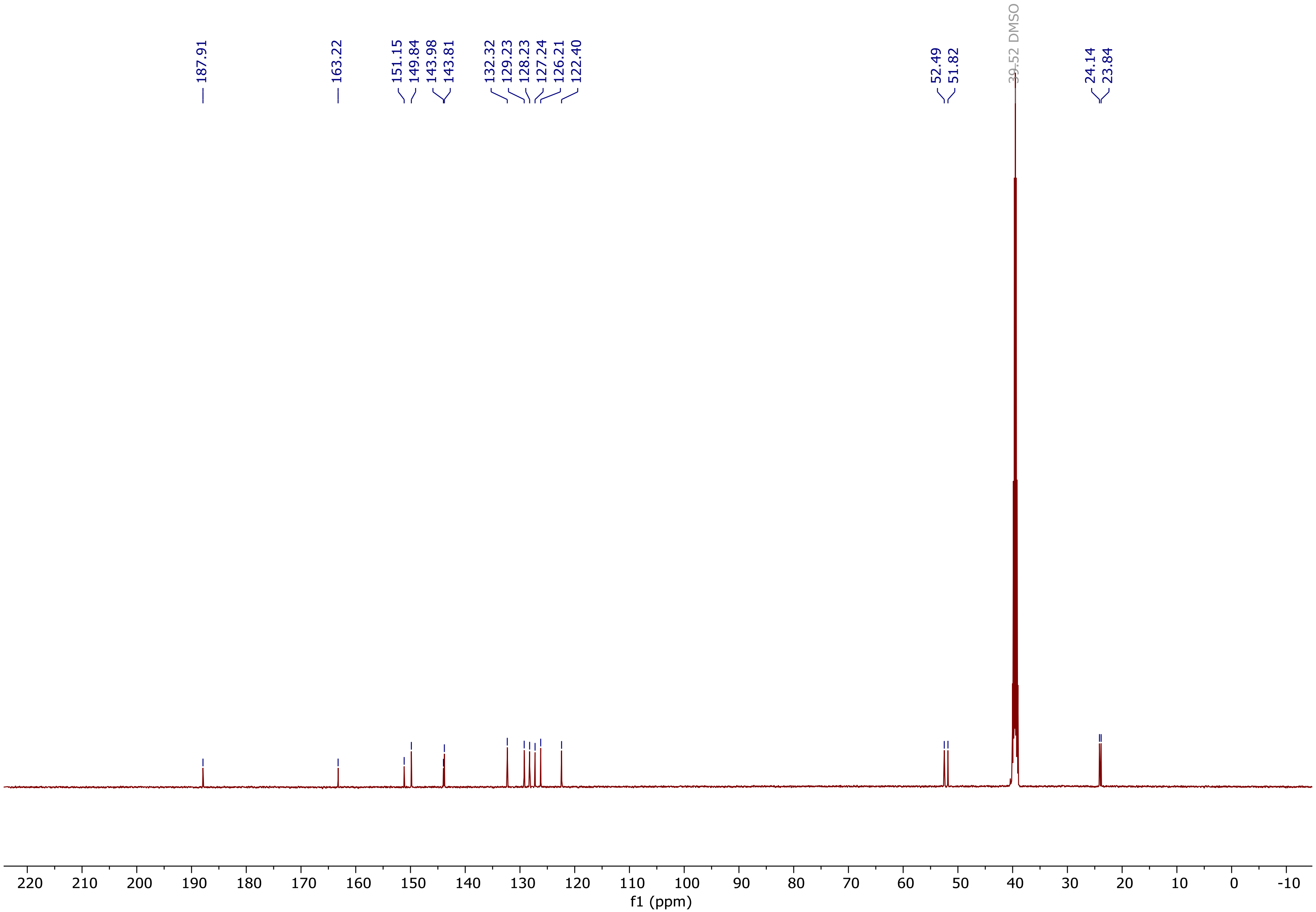


**Supplementary Figure 63.** $^{13}C$ NMR of **1F** in DMSO-$d^6$.

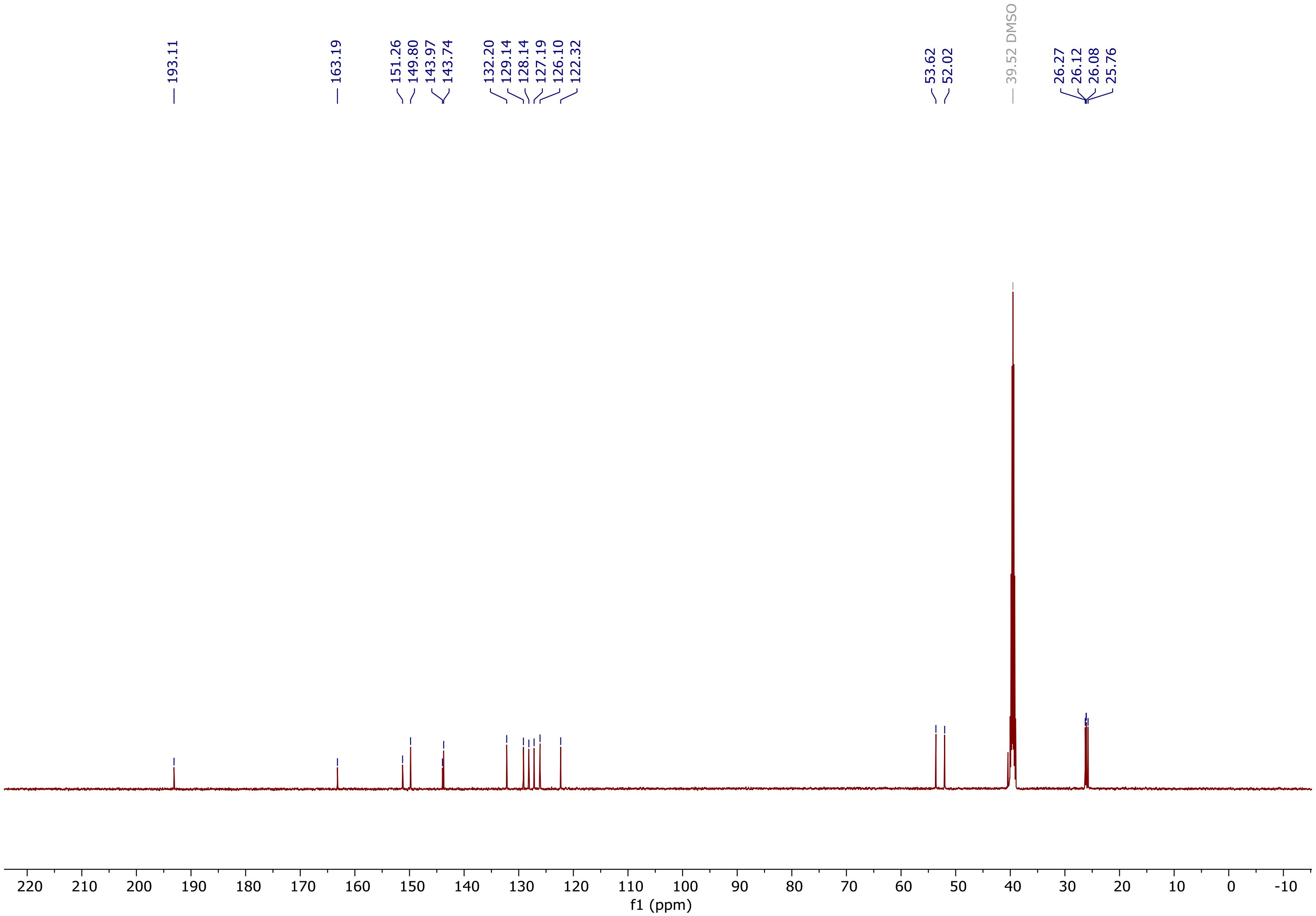


**Supplementary Figure 64.** $^{13}C$ NMR of **1G** in DMSO-$d^6$.

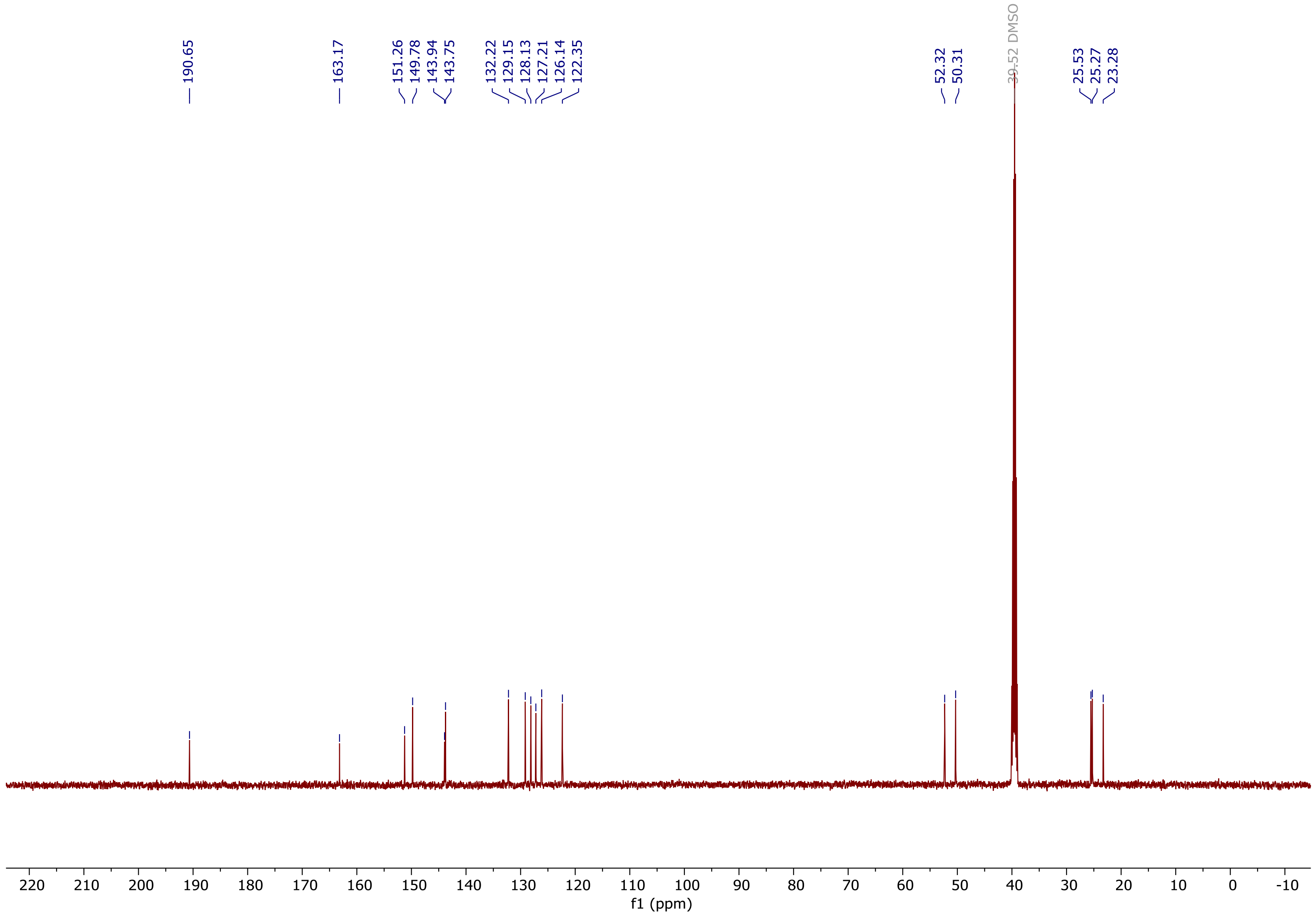


**Supplementary Figure 65.** $^{13}C$ NMR of **1H** in DMSO-d$^{6}$.

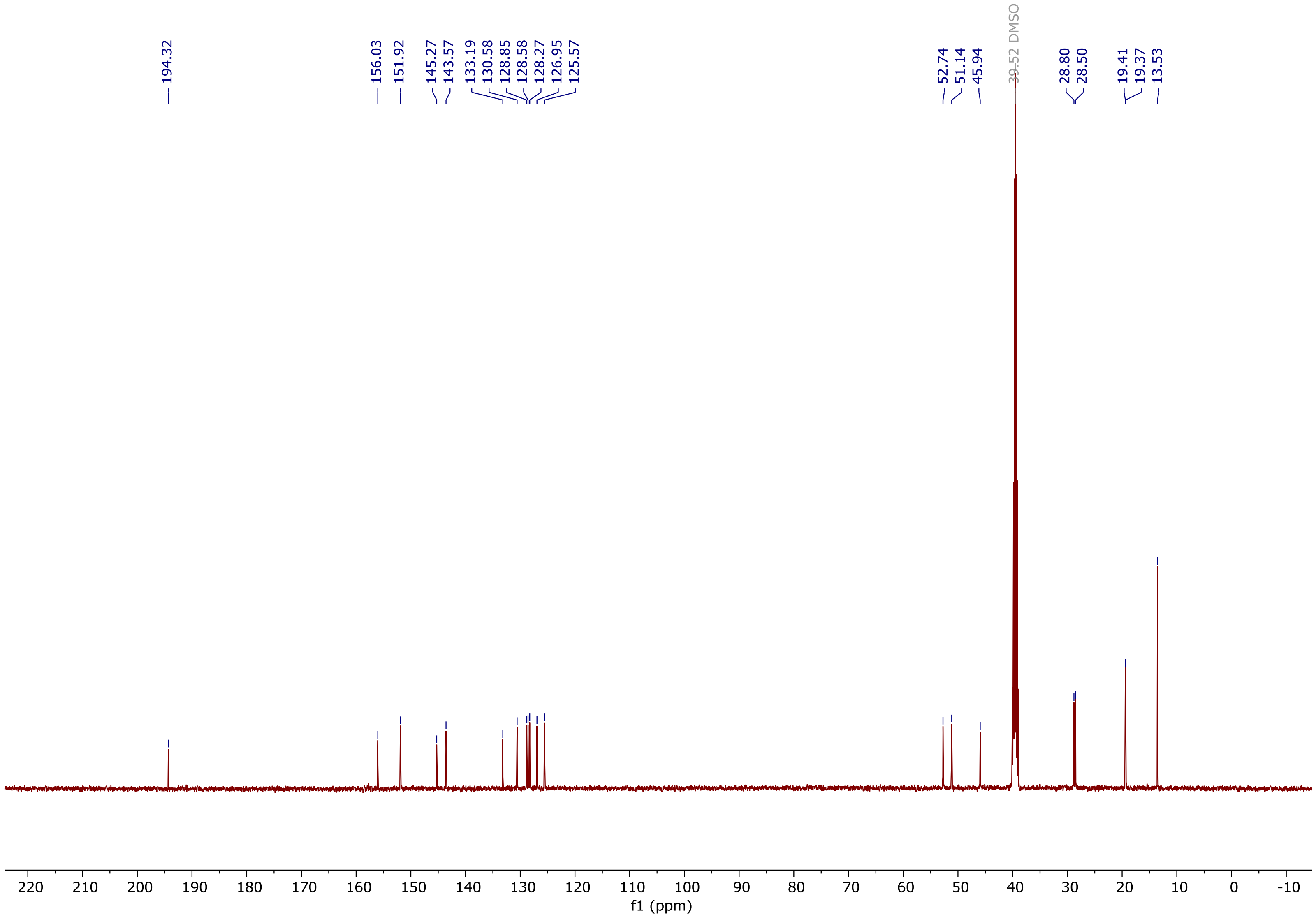


**Supplementary Figure 66.** $^{13}$C NMR of **2C** in DMSO-d$^{6}$.

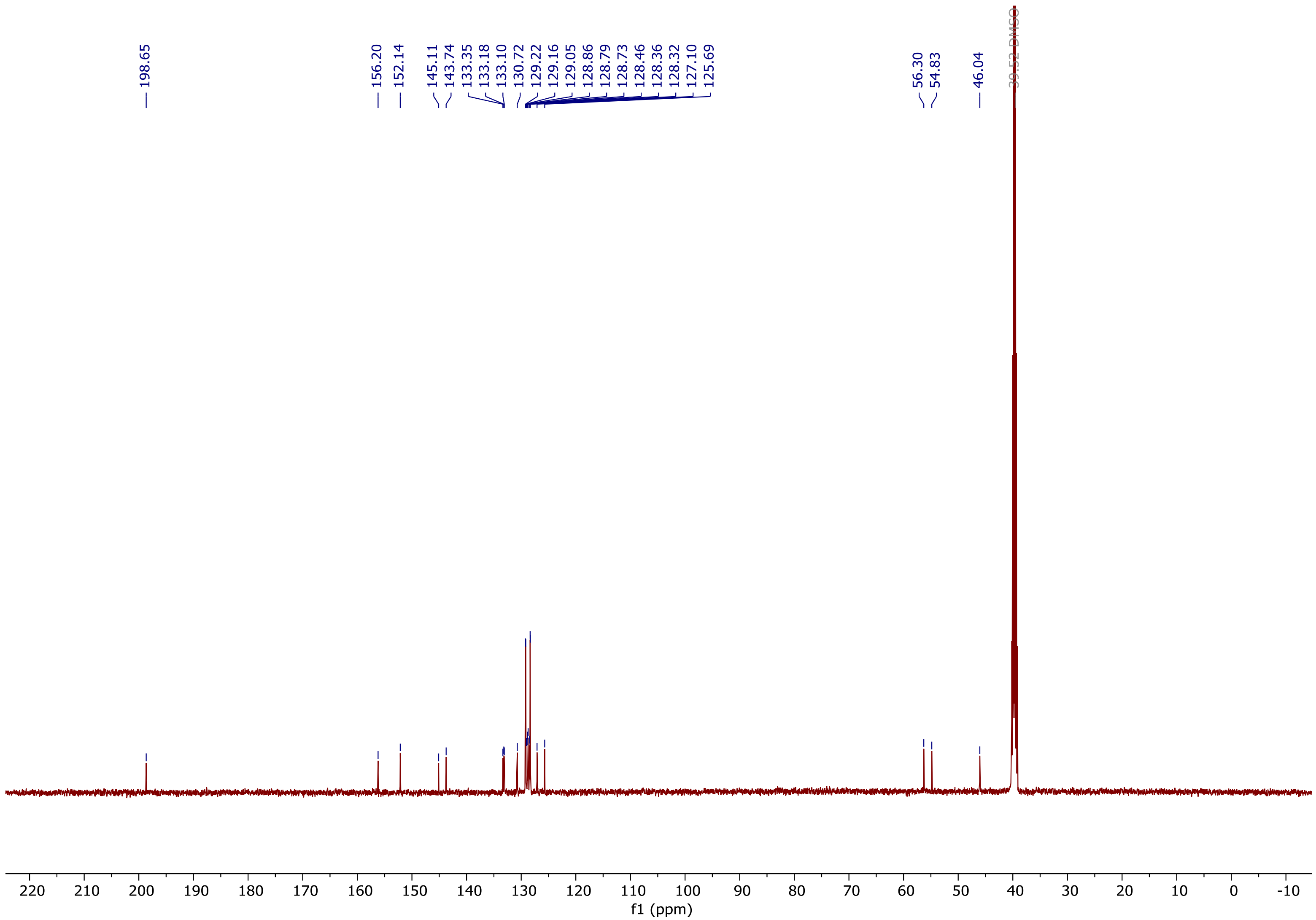


**Supplementary Figure 67.** $^{13}C$ NMR of **2D** in DMSO-d[6].

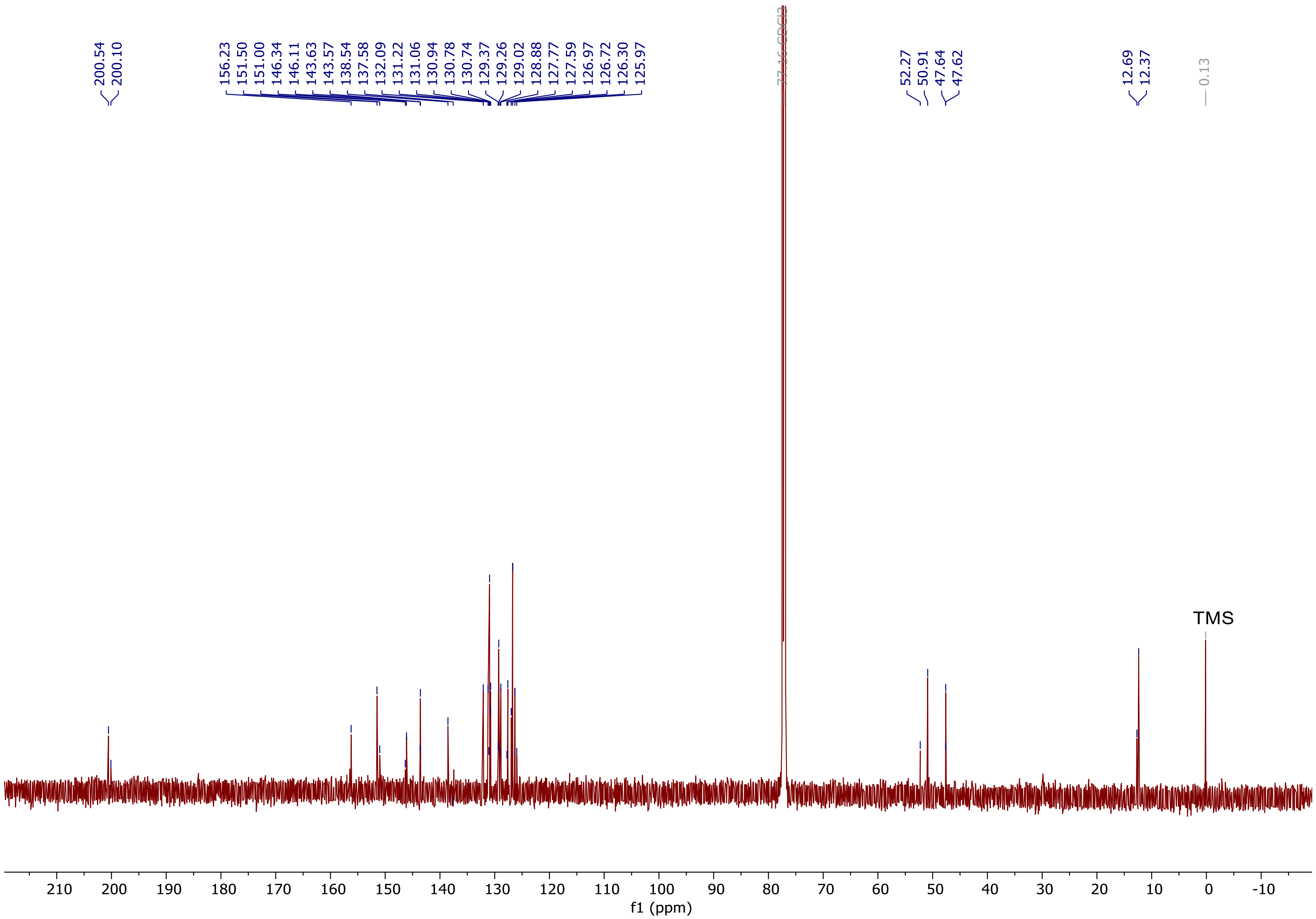


**Supplementary Figure 68.** $^{13}$C NMR of **2E** in $CDCl_3$.

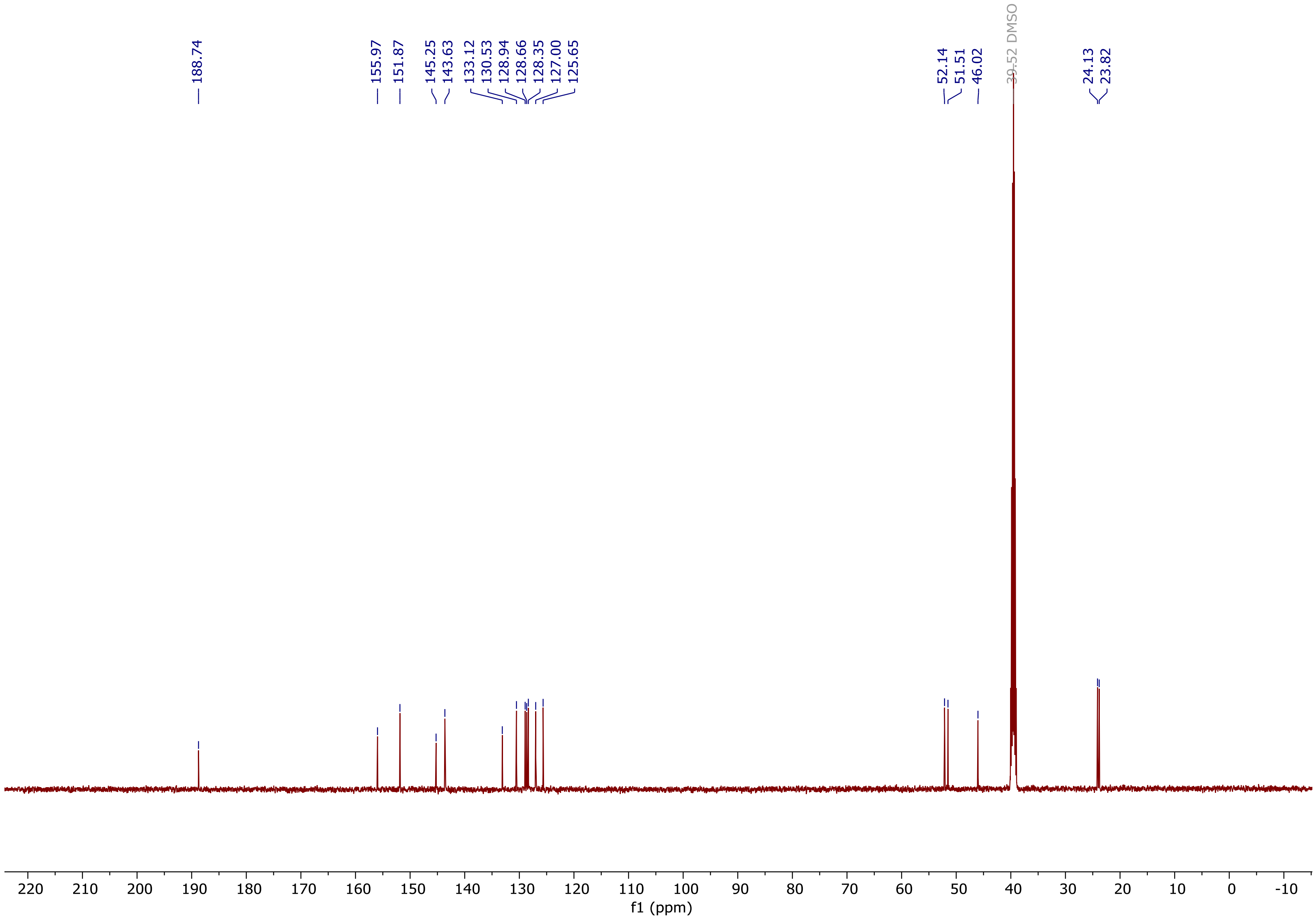


**Supplementary Figure 69.** $^{13}$C NMR of **2F** in DMSO-d$^6$.

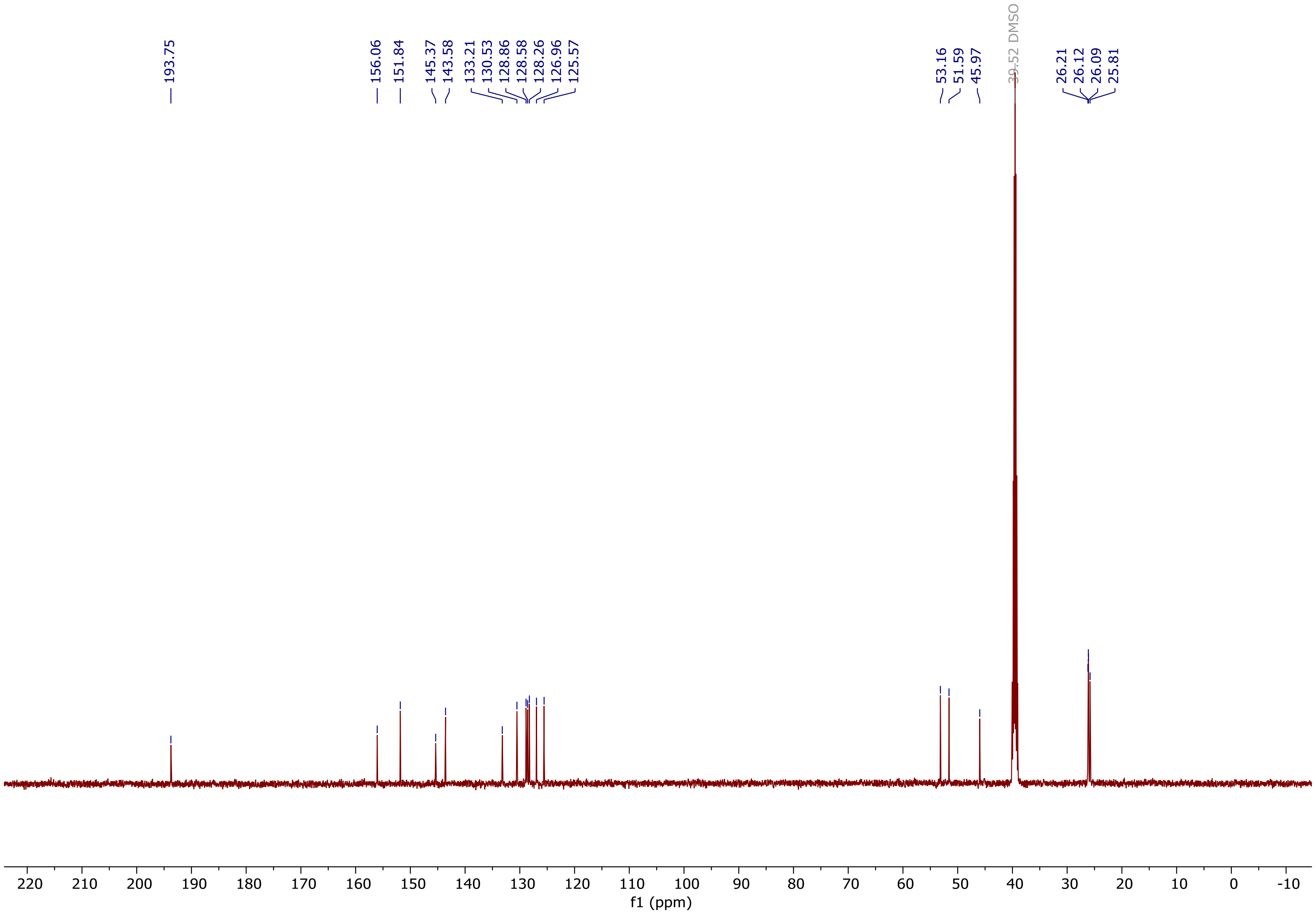


**Supplementary Figure 70.** $^{13}C$ NMR of **2G** in DMSO-d$^{6}$.

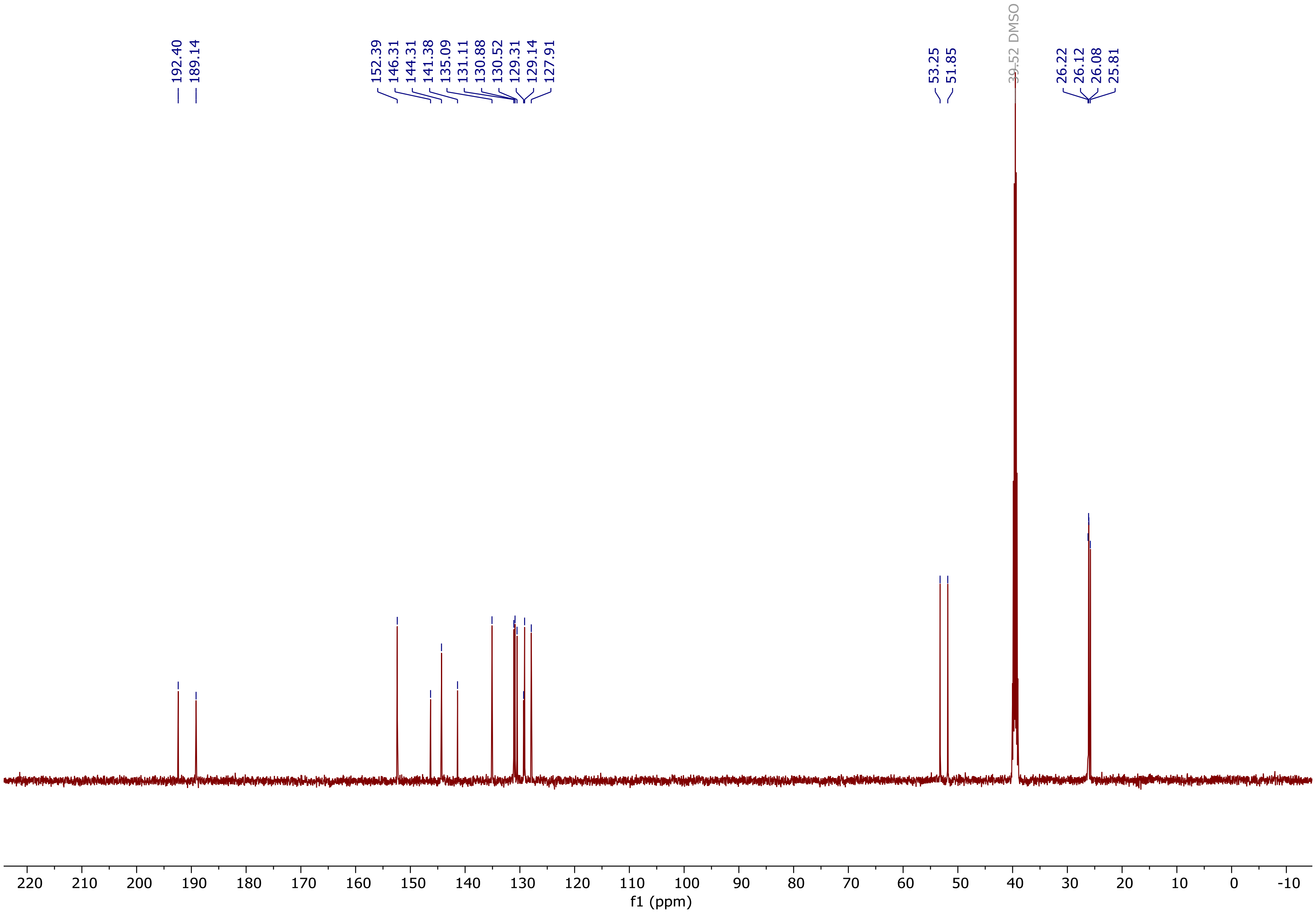


**Supplementary Figure 71.** $^{13}C$ NMR of **3G** in DMSO-$d^6$.

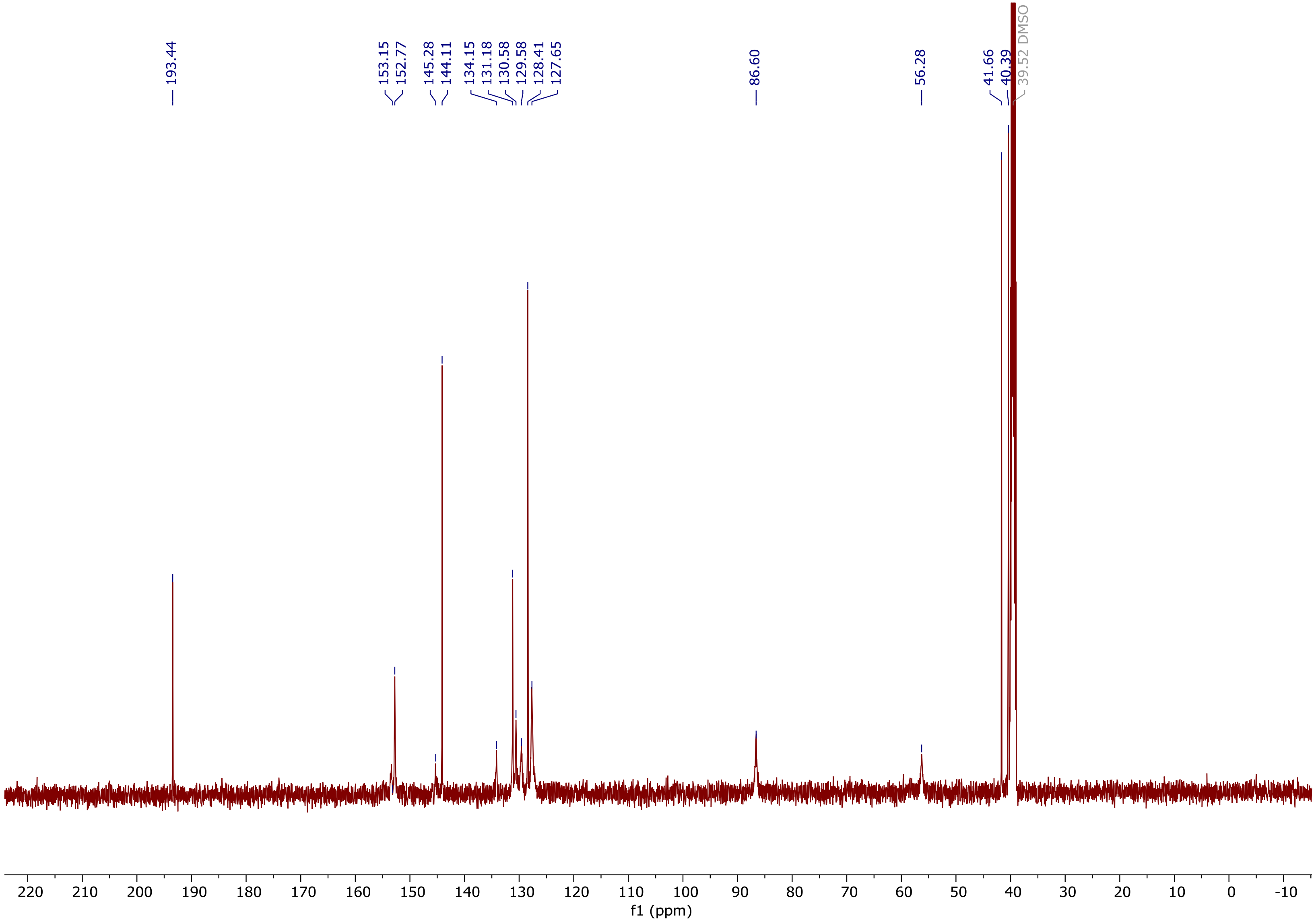


**Supplementary Figure 72.** $^{13}C$ NMR of **4A** in DMSO-$d^6$.

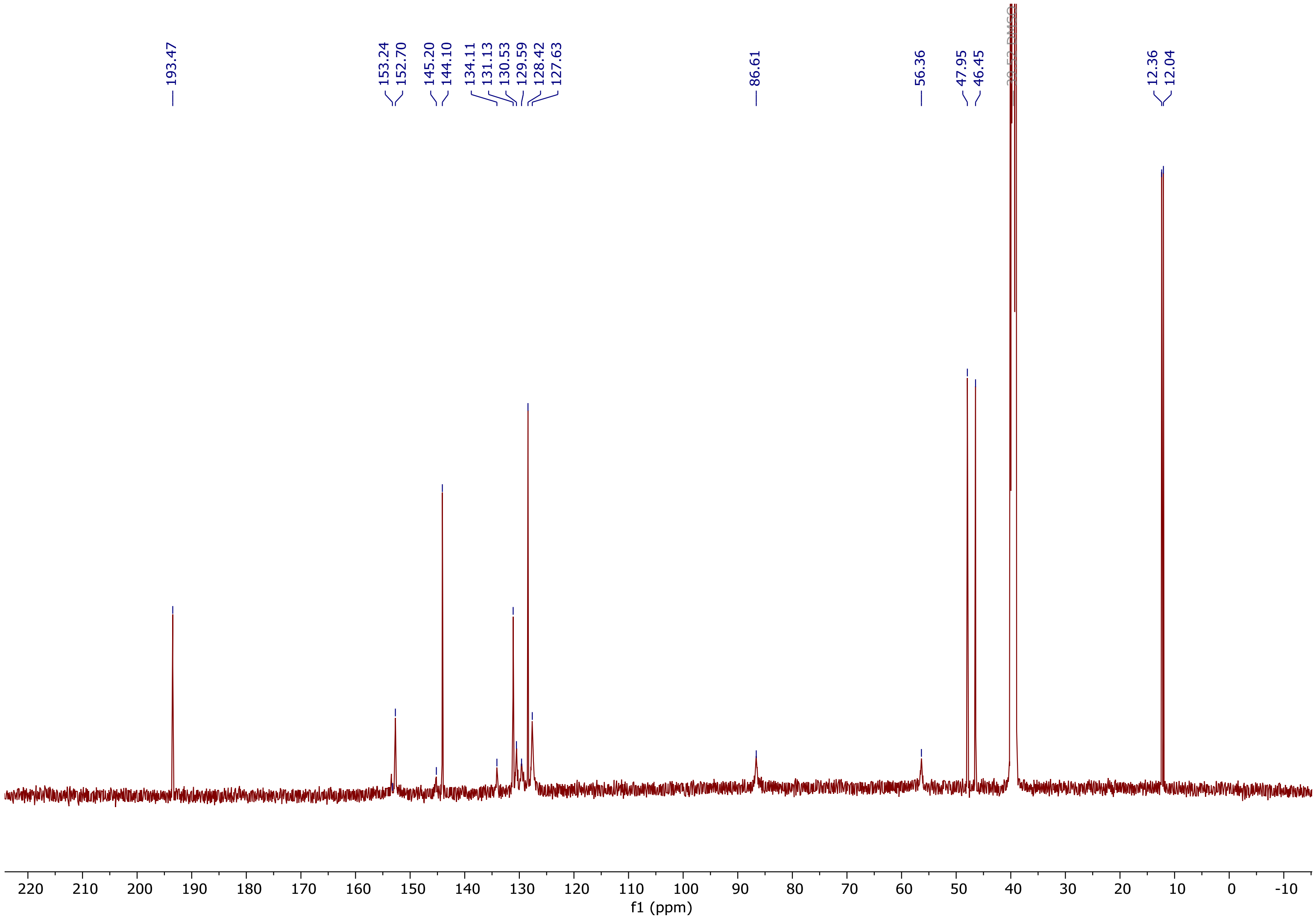


**Supplementary Figure 73.** $^{13}$C NMR of **4B** in DMSO-d$^{6}$.

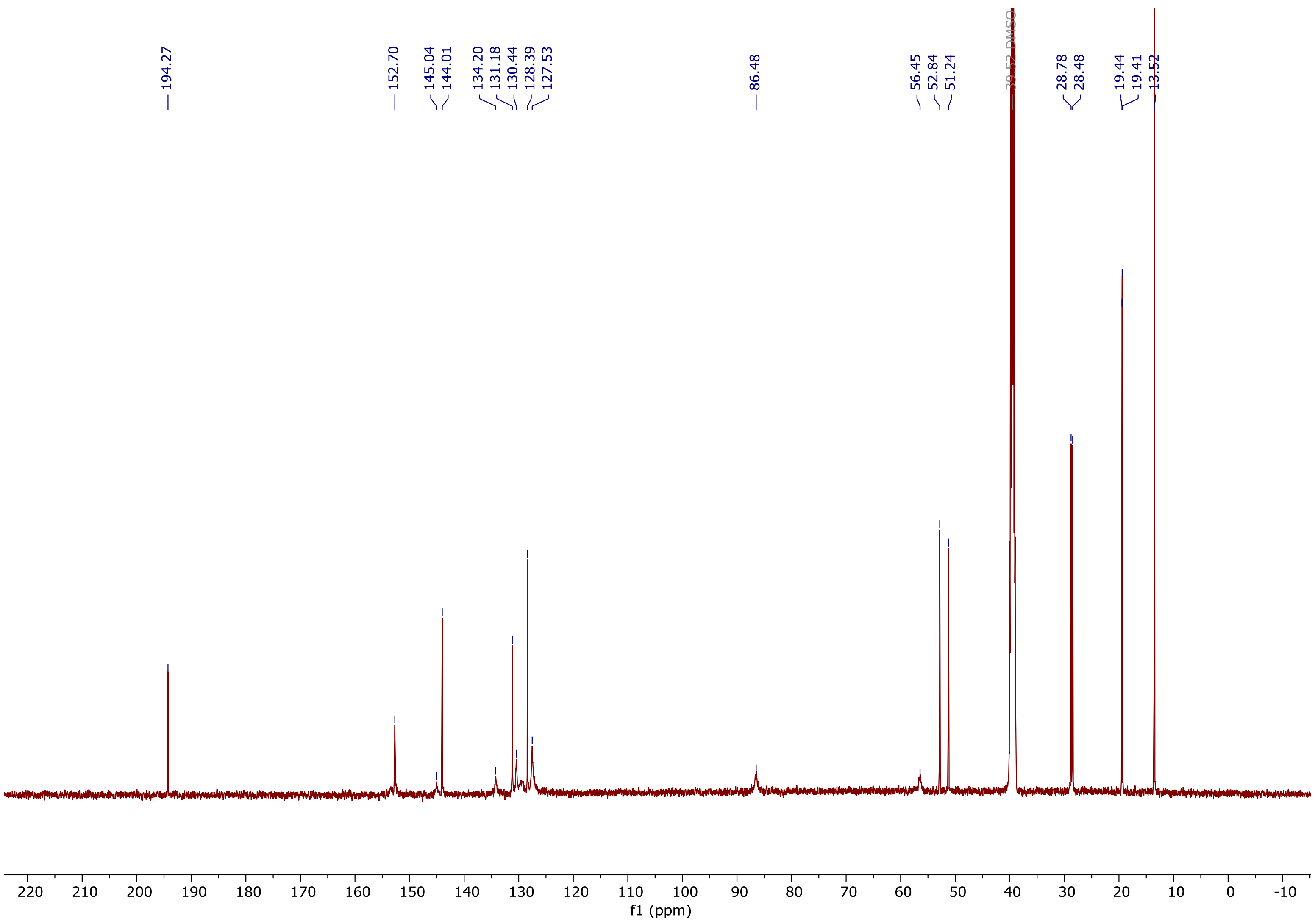


**Supplementary Figure 74.** $^{13}C$ NMR of **4C** in DMSO-$d^6$.

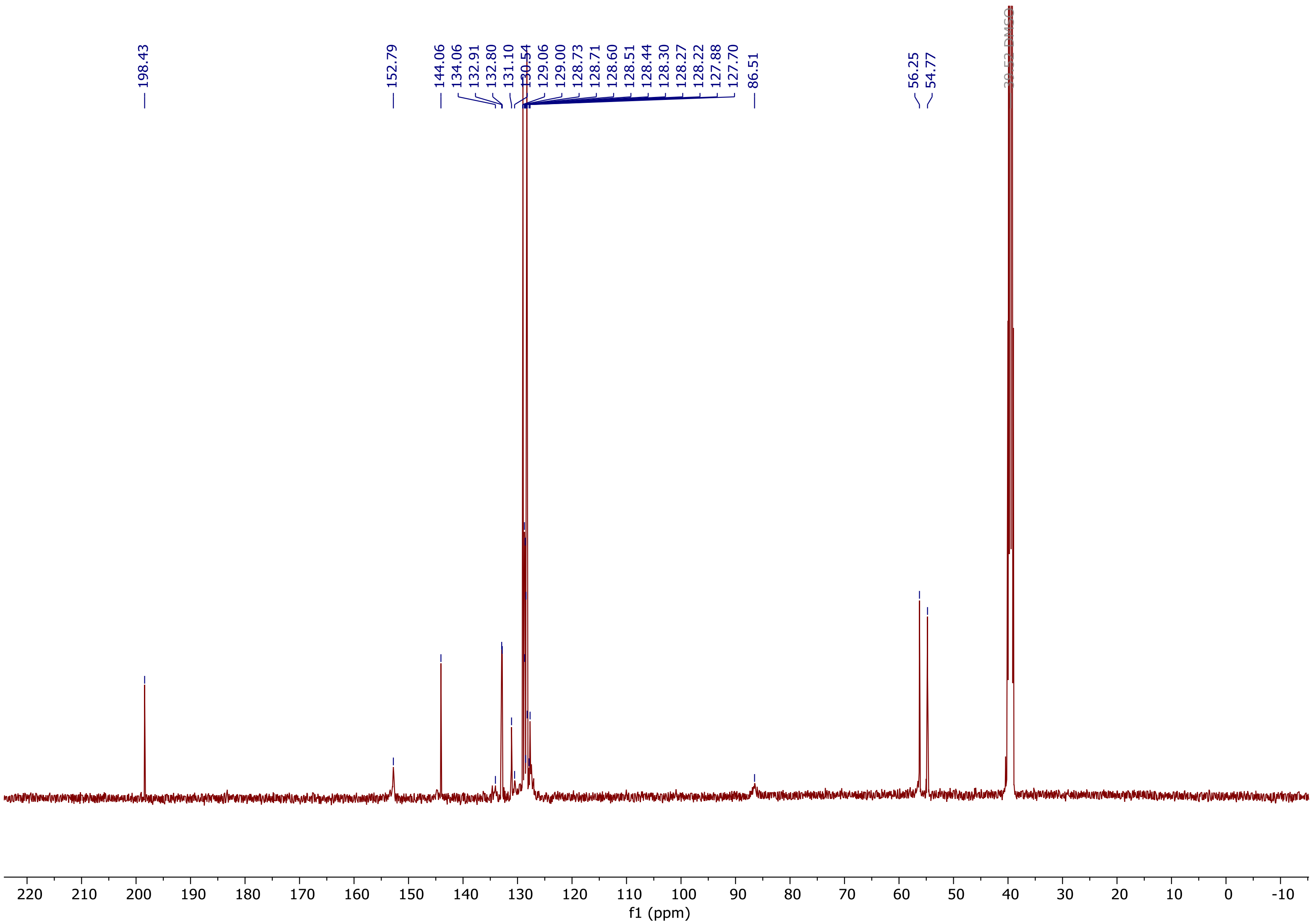


**Supplementary Figure 75.** $^{13}$C NMR of **4D** in DMSO-d$^6$.

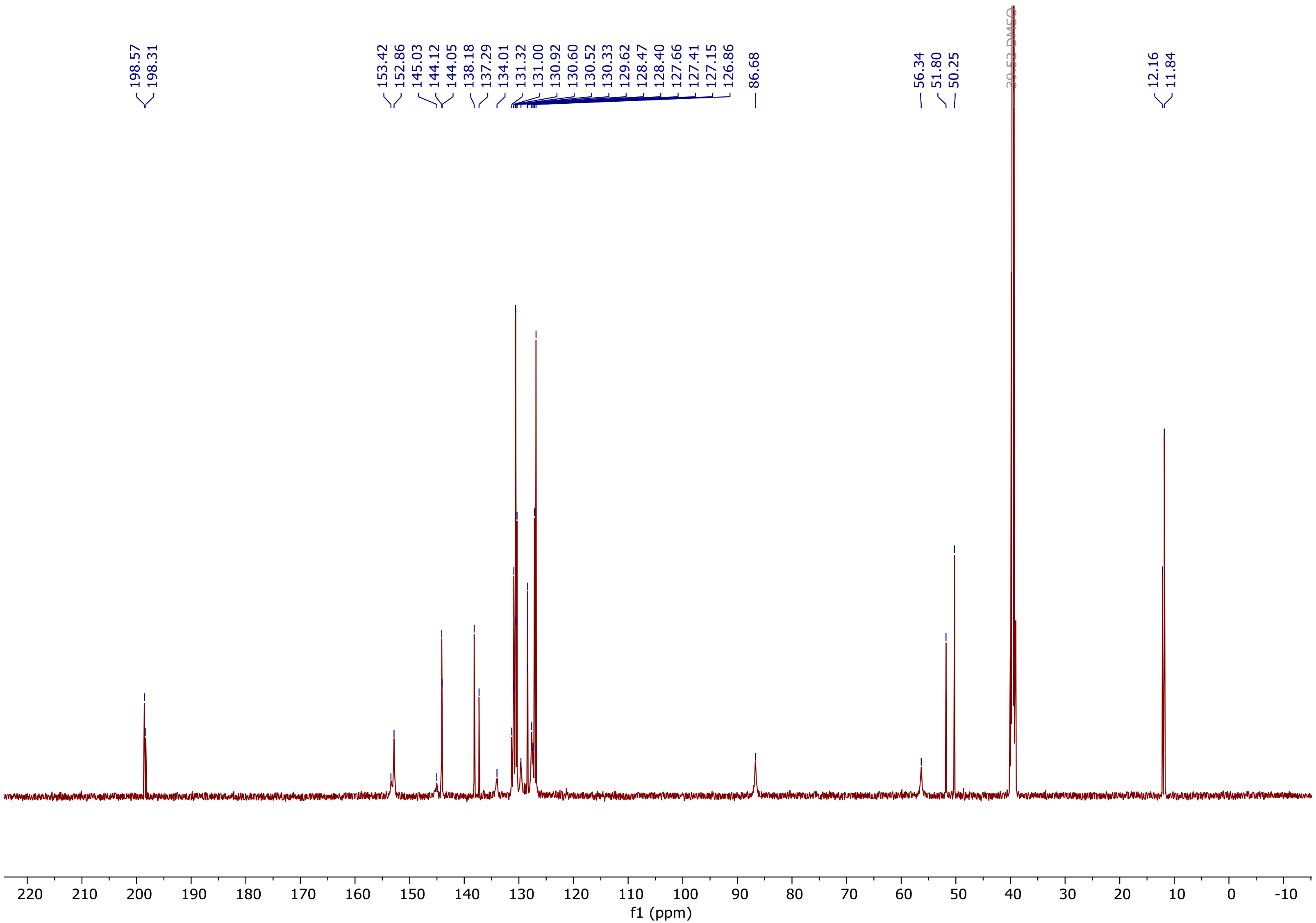


**Supplementary Figure 76.** $^{13}C$ NMR of **4E** in DMSO-d[6].

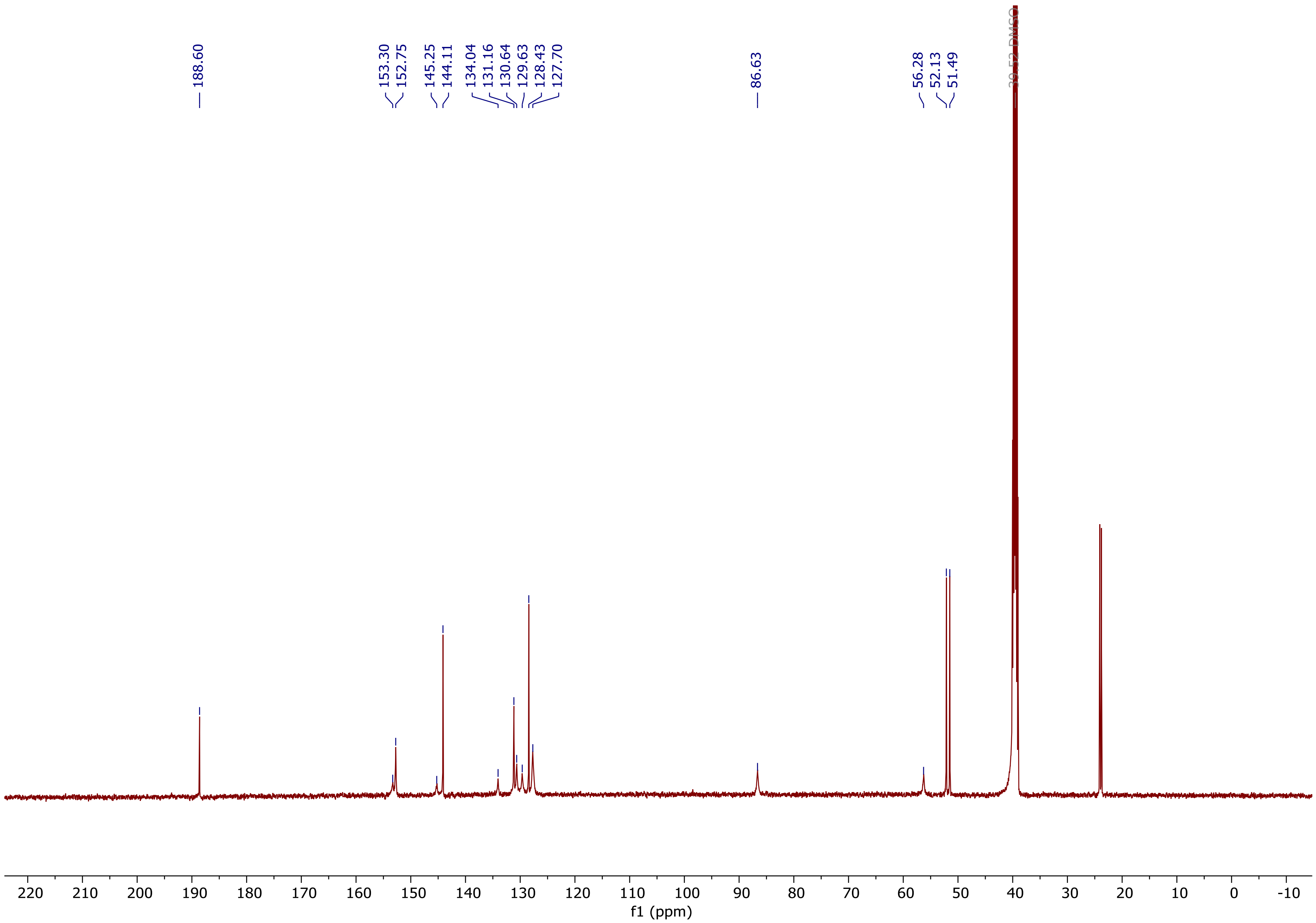


**Supplementary Figure 77.** $^{13}$C NMR of **4F** in DMSO-d$^6$.

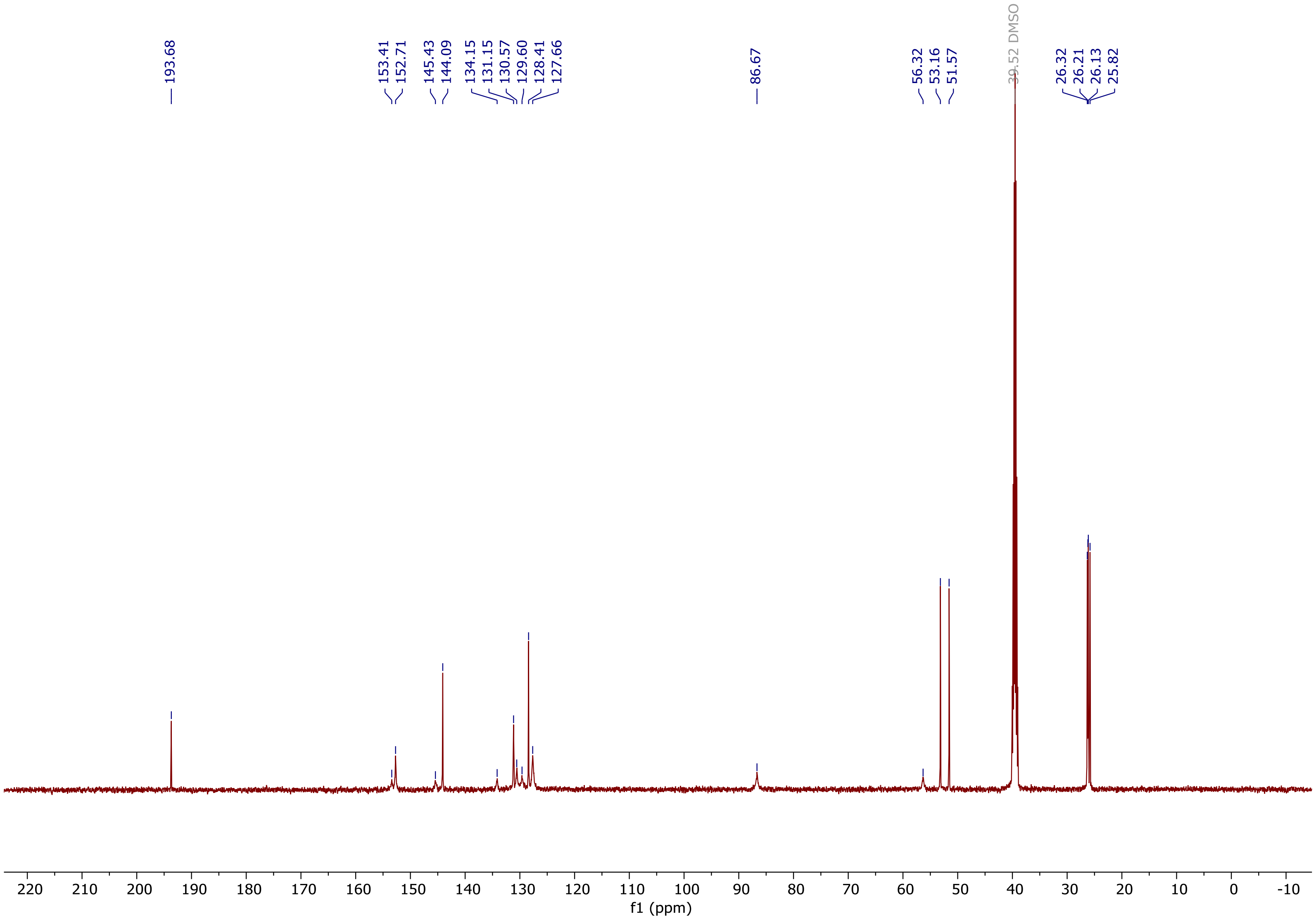


**Supplementary Figure 78.** $^{13}C$ NMR of **4G** in DMSO-d$^{6}$.

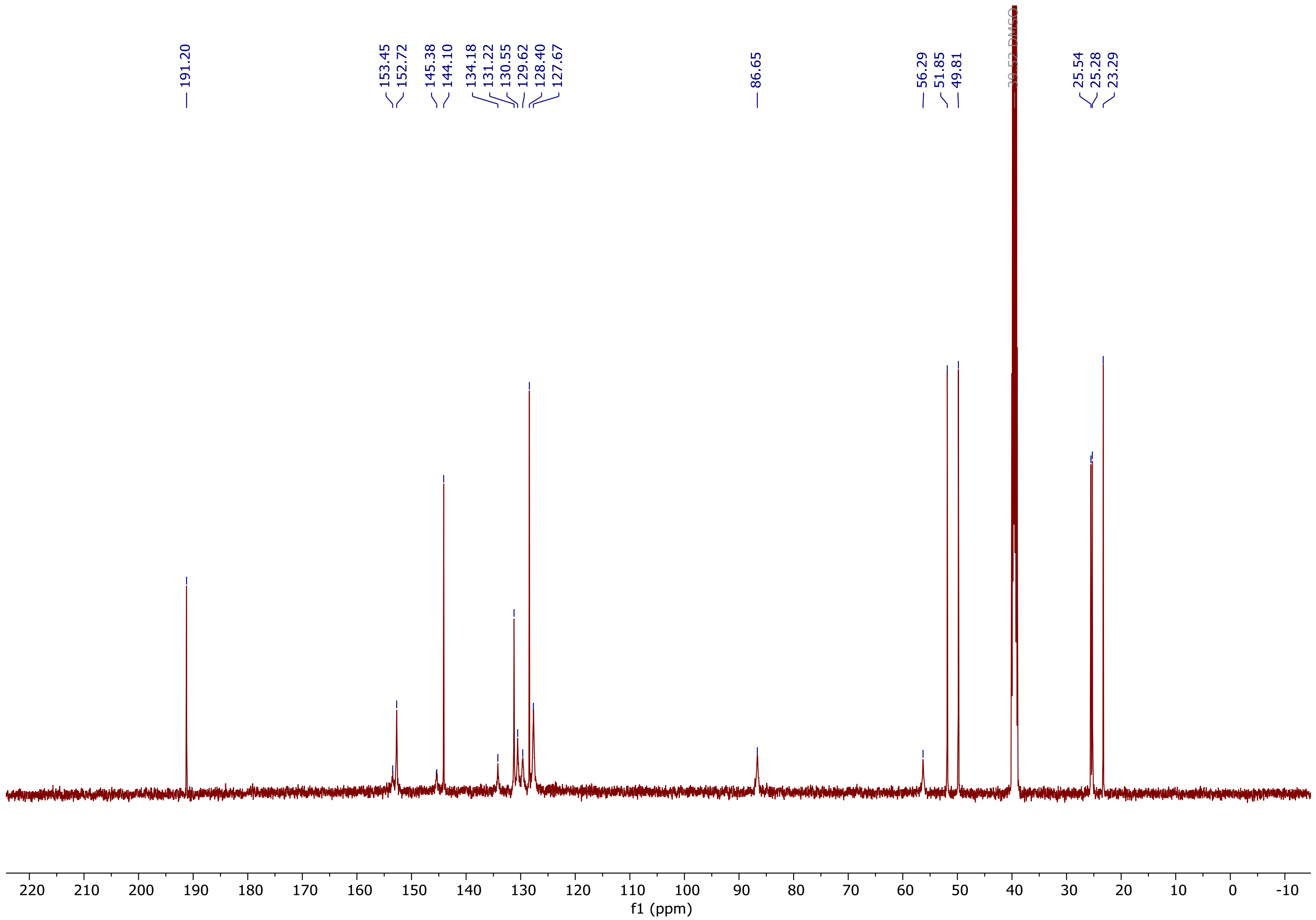


**Supplementary Figure 79.** $^{13}$C NMR of **4H** in DMSO-d$^{6}$.

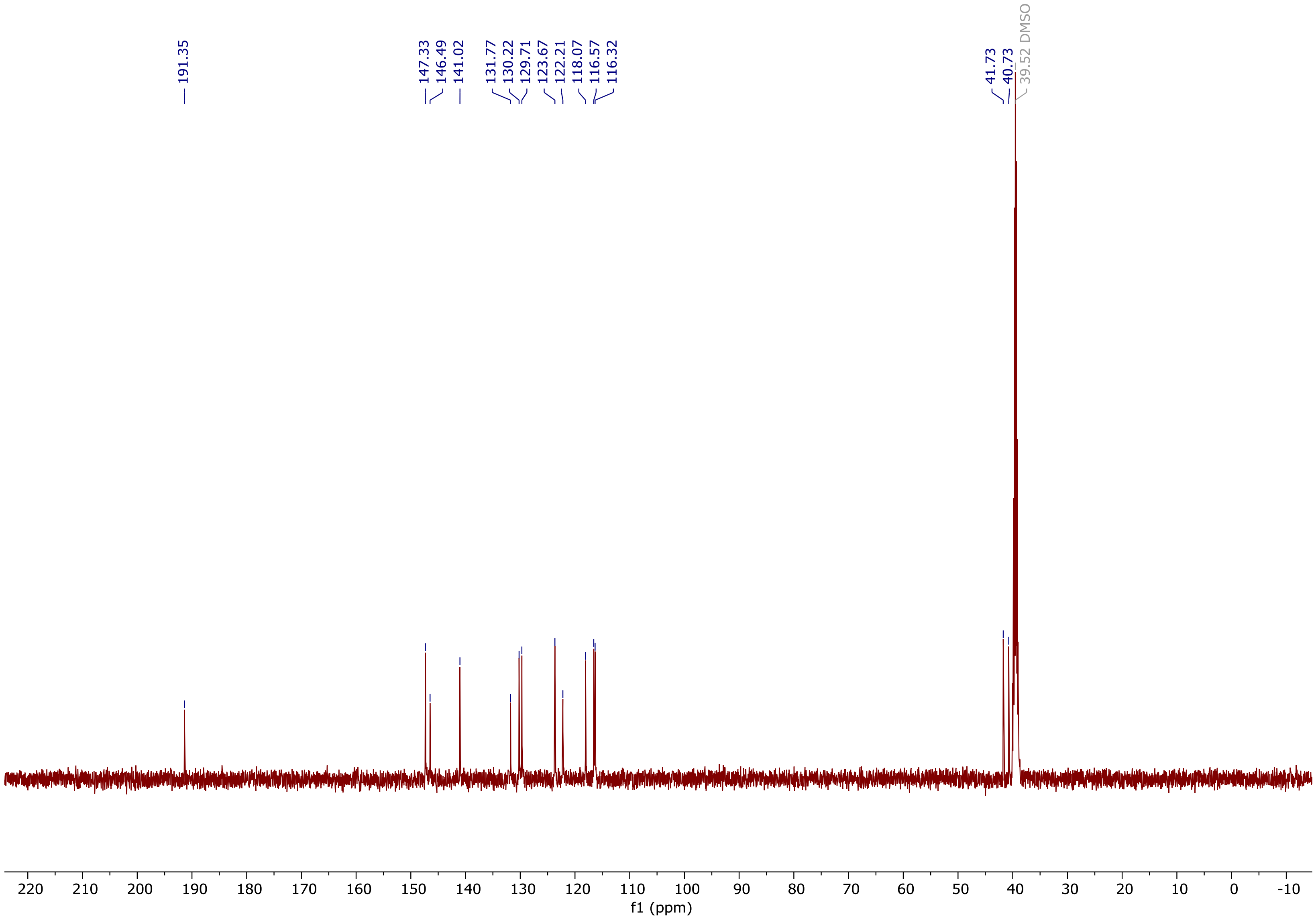


**Supplementary Figure 80.** $^{13}$C NMR of **5A** in DMSO-d$^6$.

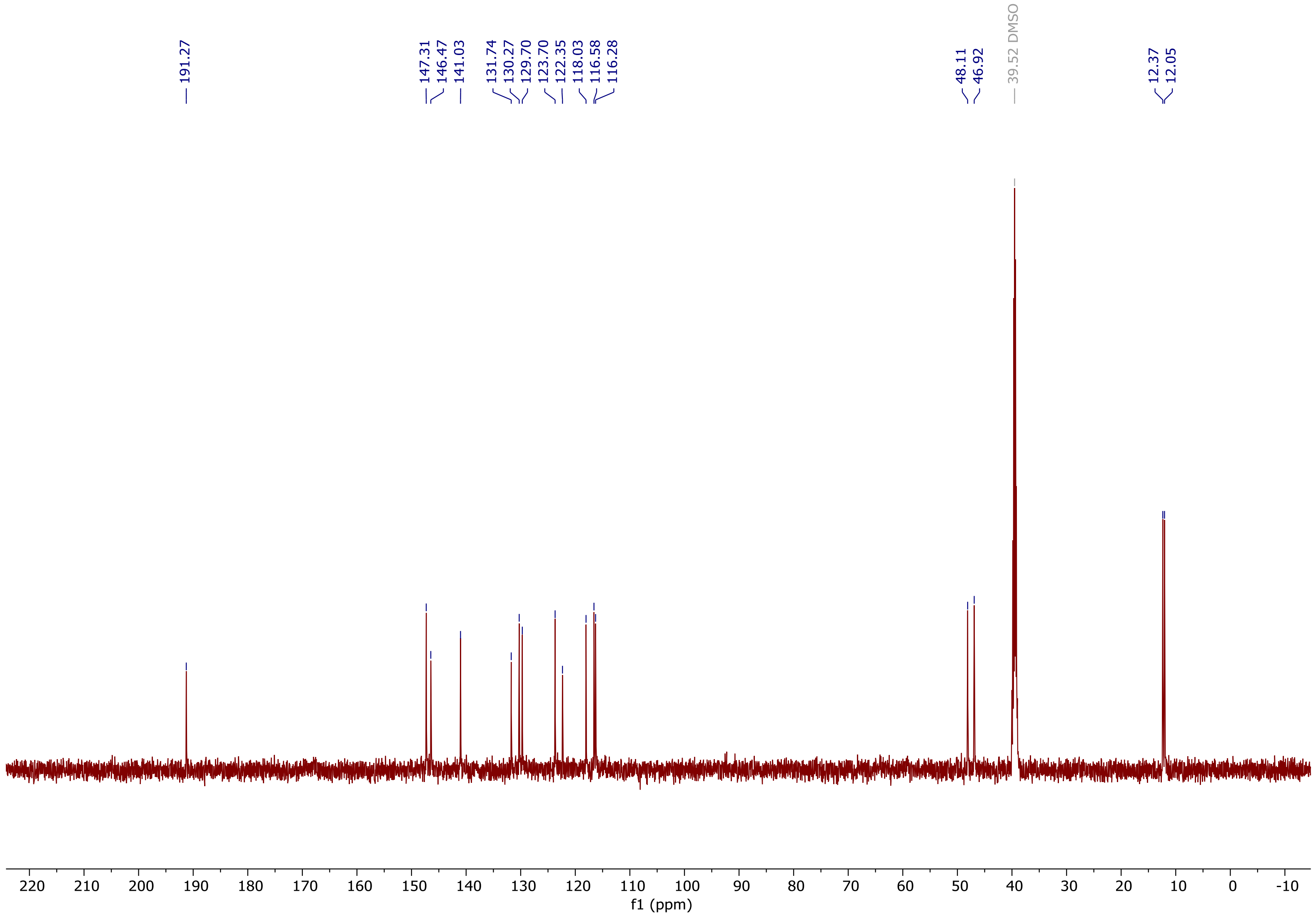


**Supplementary Figure 81.** $^{13}$C NMR of **5B** in DMSO-d$^6$.

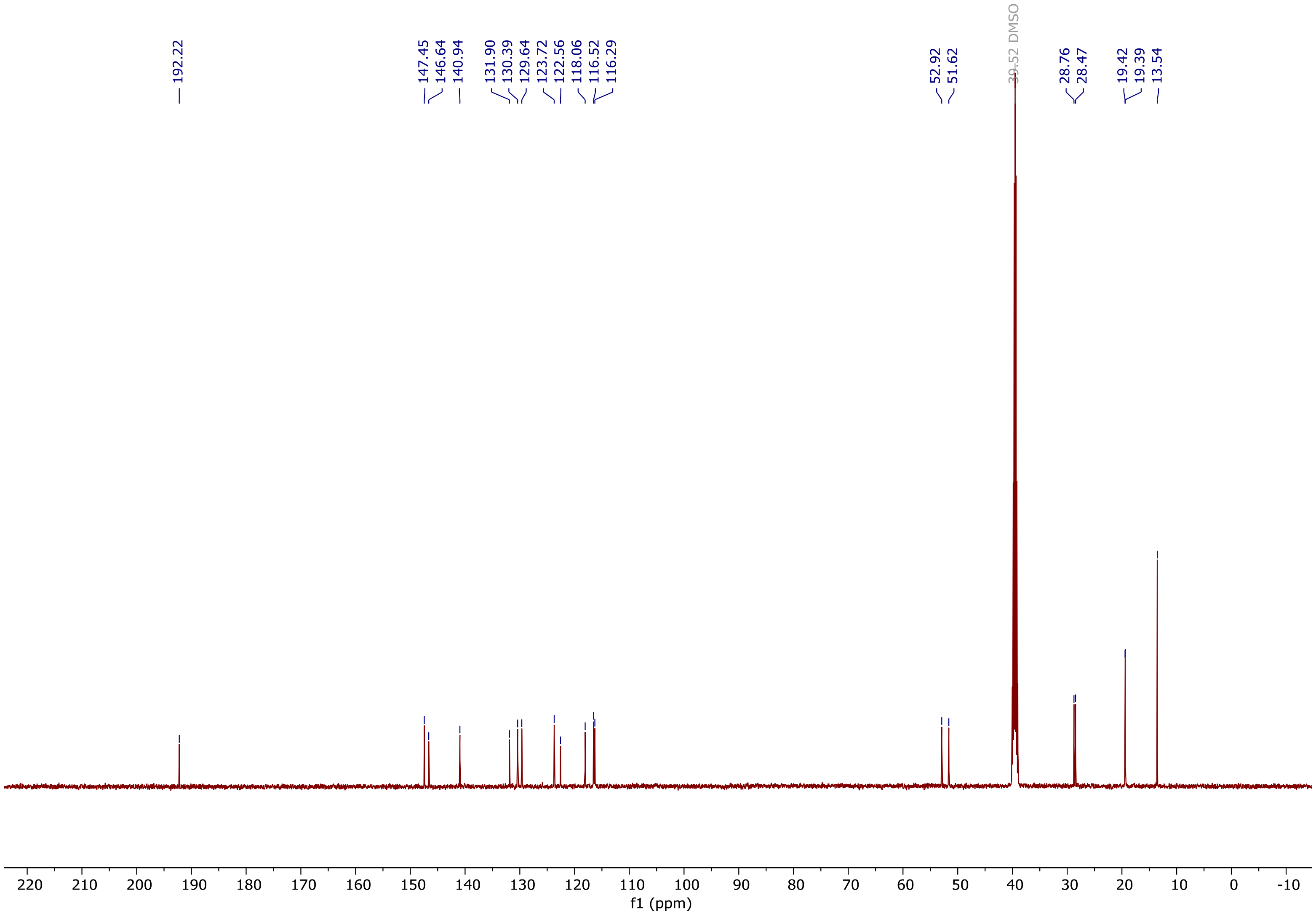


**Supplementary Figure 82.** $^{13}$C NMR of **5C** in DMSO-d$^{6}$.

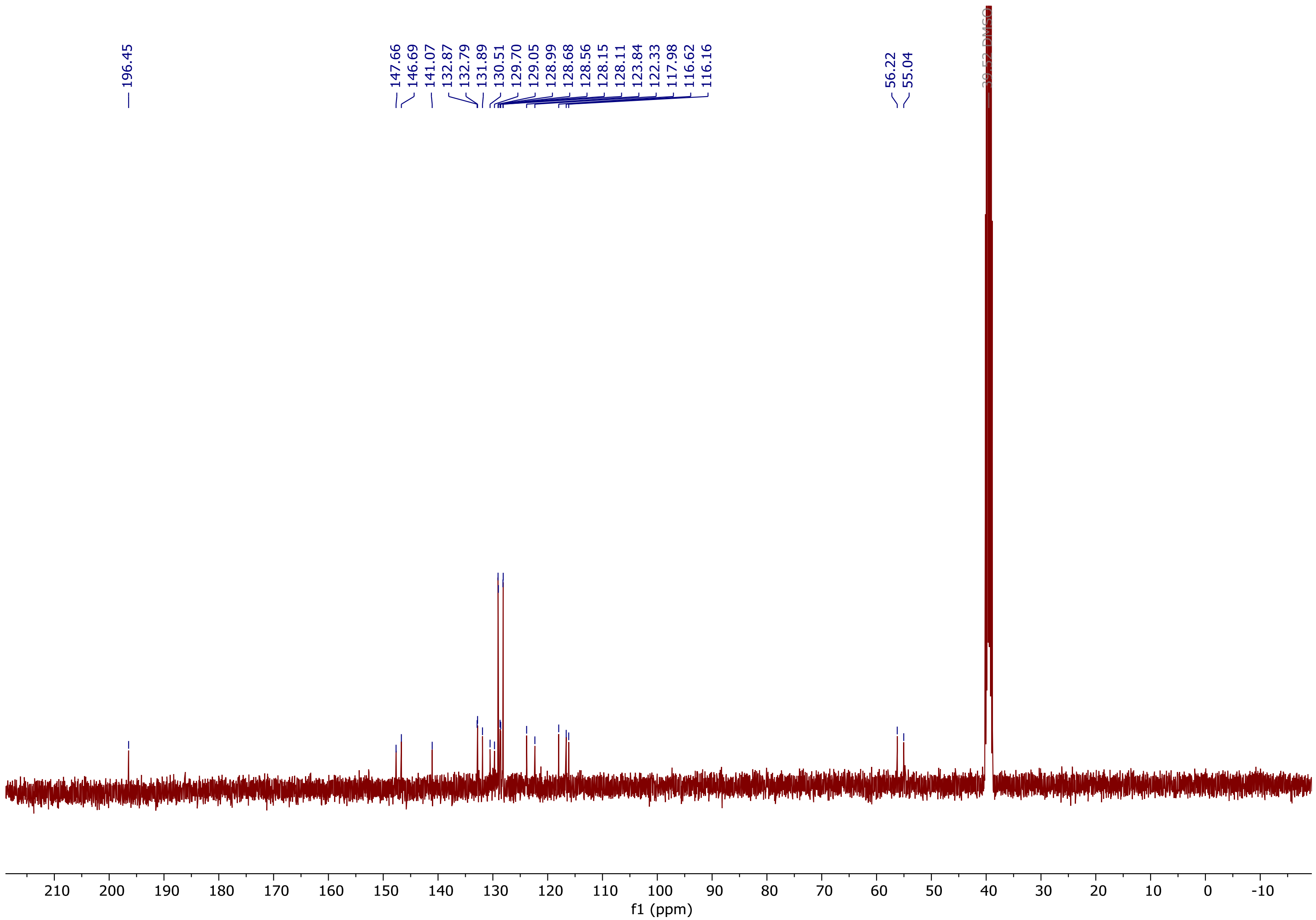


**Supplementary Figure 83.** $^{13}$C NMR of **5D** in DMSO-d$^{6}$.

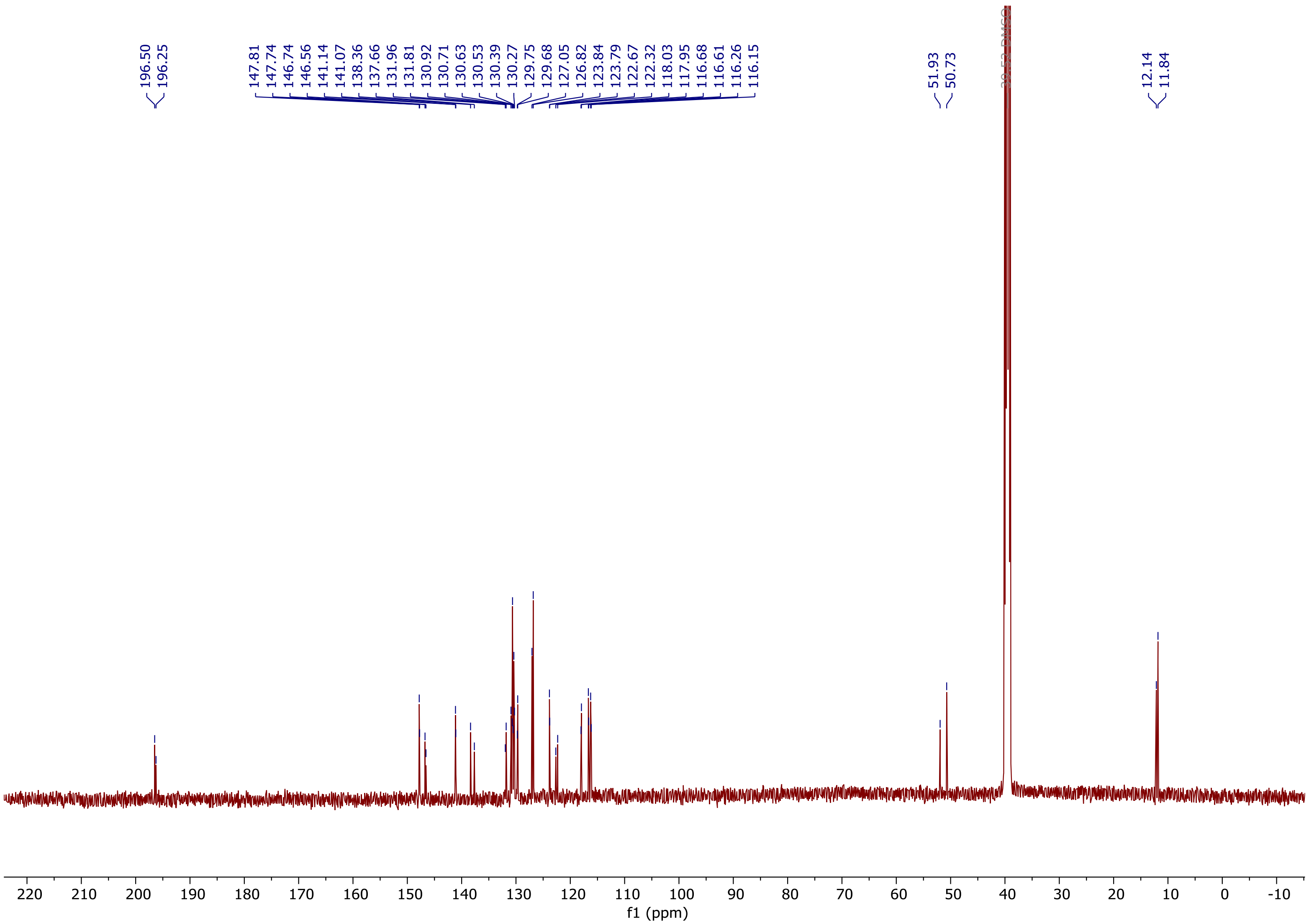


**Supplementary Figure 84.** $^{13}$C NMR of **5E** in DMSO-d$^{6}$.

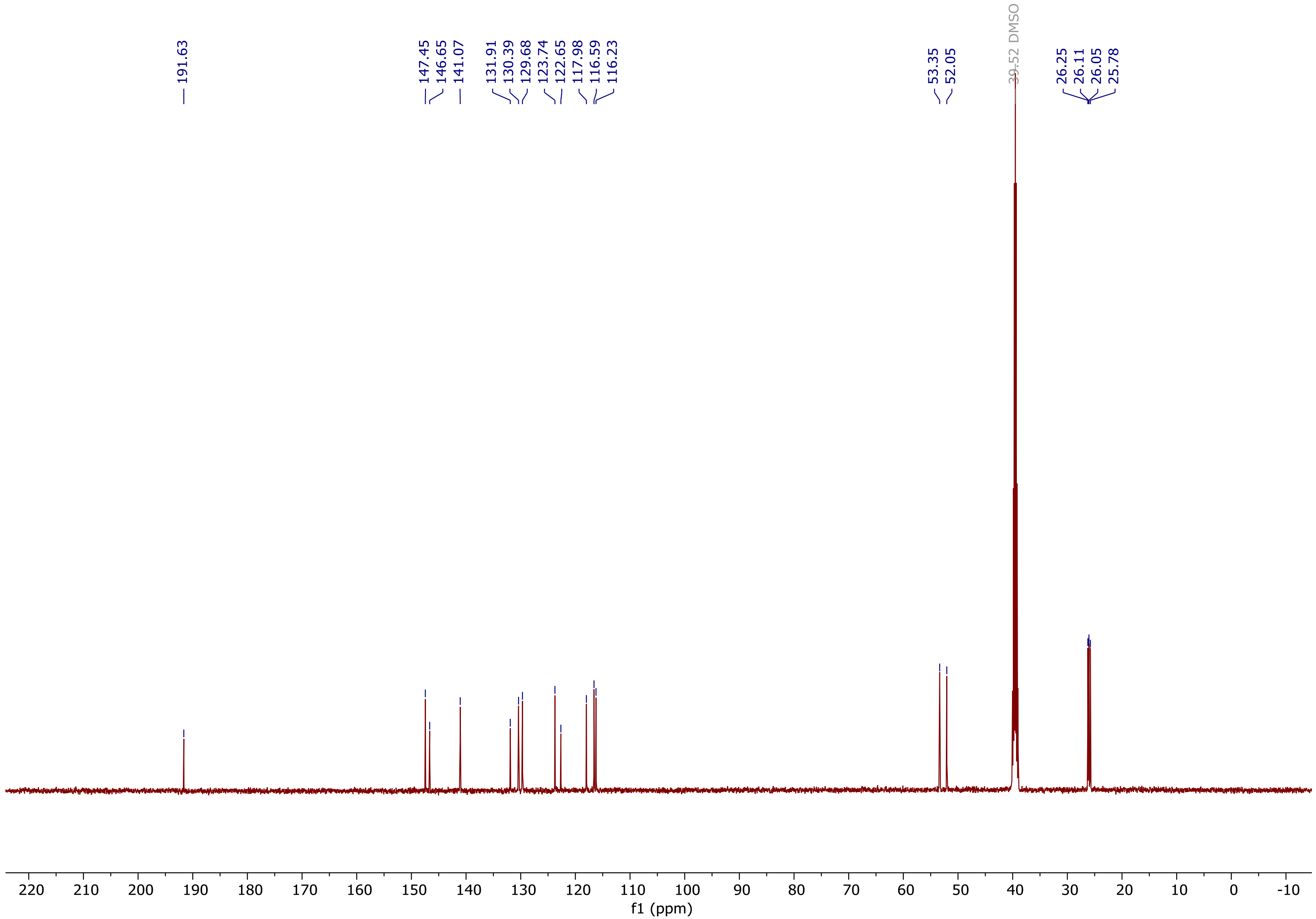


**Supplementary Figure 85.** $^{13}C$ NMR of **5G** in DMSO-$d^6$.

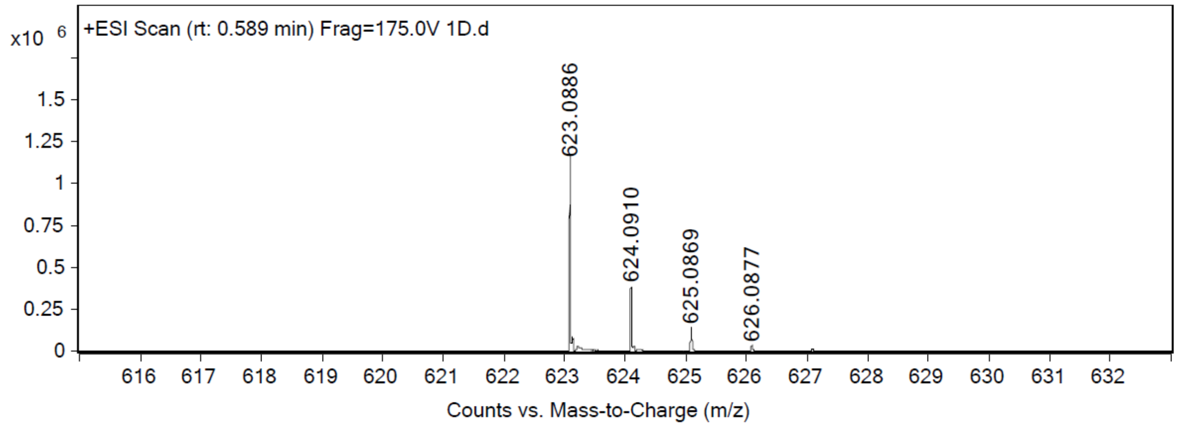


**Supplementary Figure 86.** High resolution ESI-MS spectrum of **1D**.

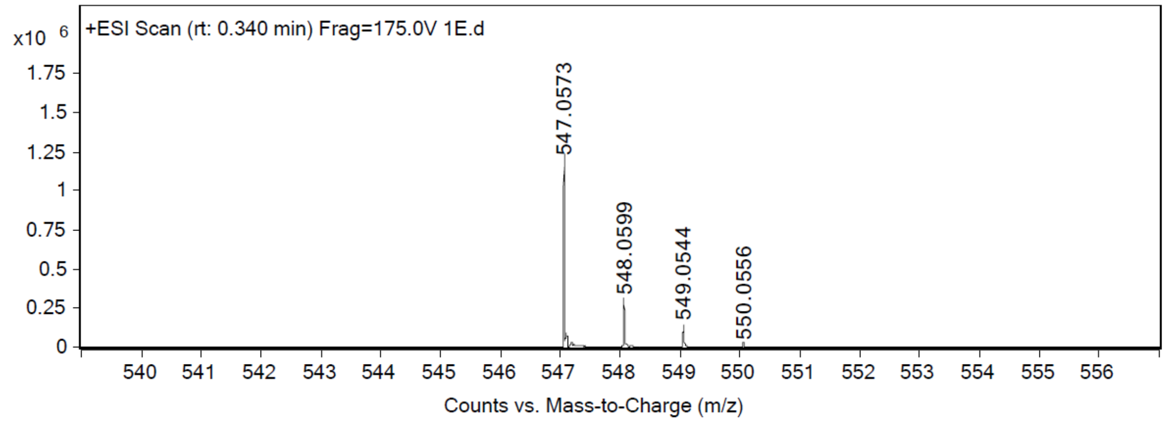


**Supplementary Figure 87.** High resolution ESI-MS spectrum of **1E**.

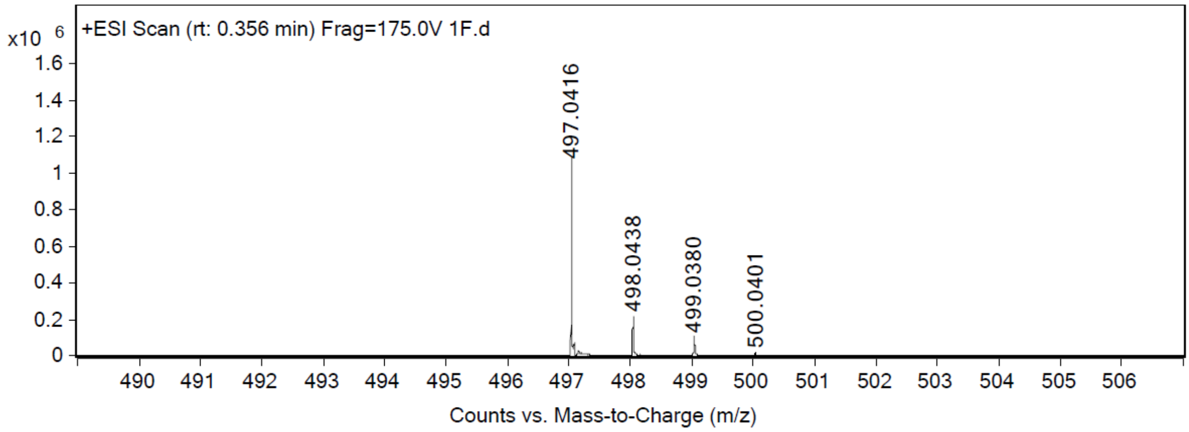


**Supplementary Figure 88.** High resolution ESI-MS spectrum of **1F**.

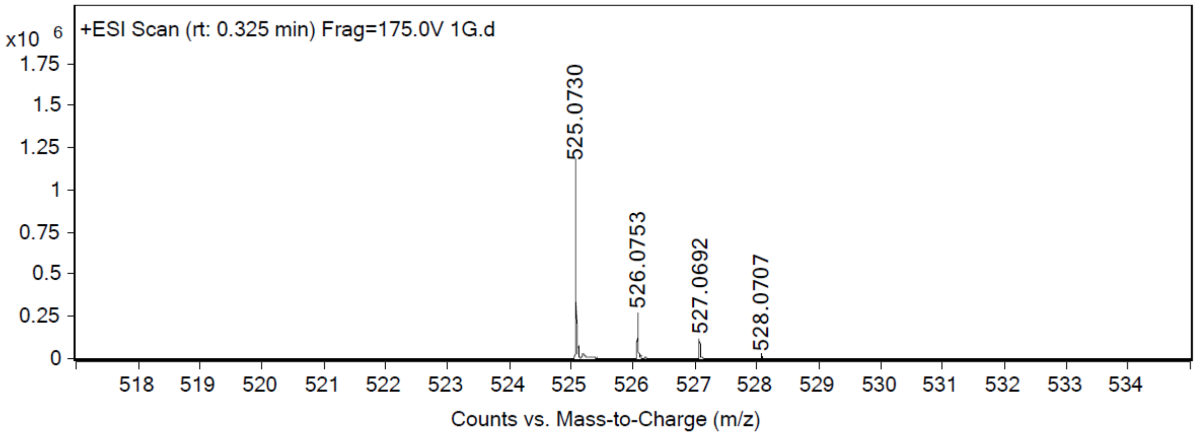


**Supplementary Figure 89.** High resolution ESI-MS spectrum of **1G**.

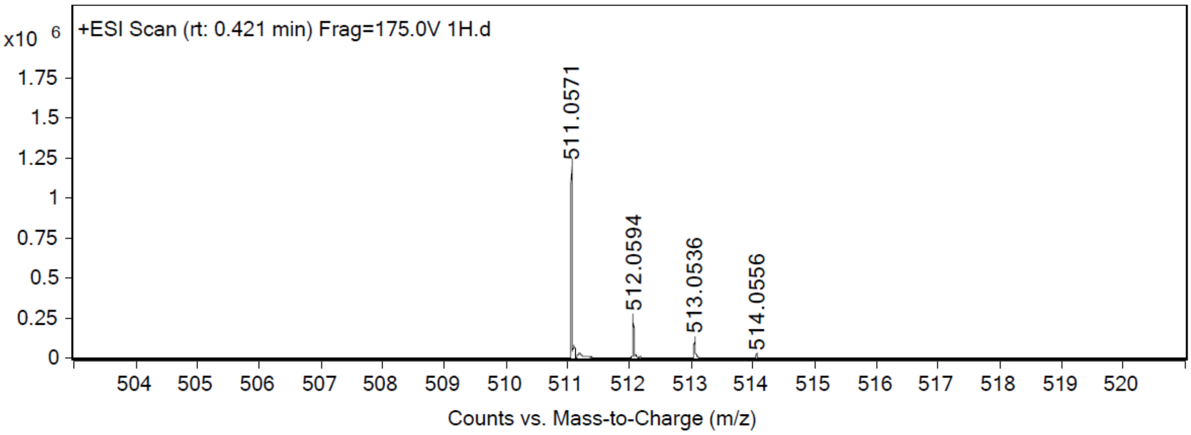


**Supplementary Figure 90.** High resolution ESI-MS spectrum of **1H**.

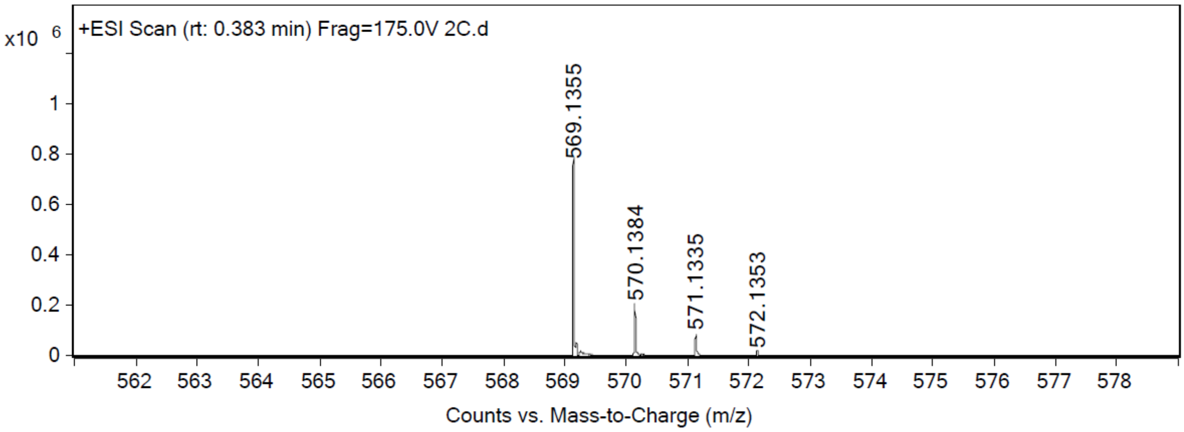


**Supplementary Figure 91.** High resolution ESI-MS spectrum of **2C**.

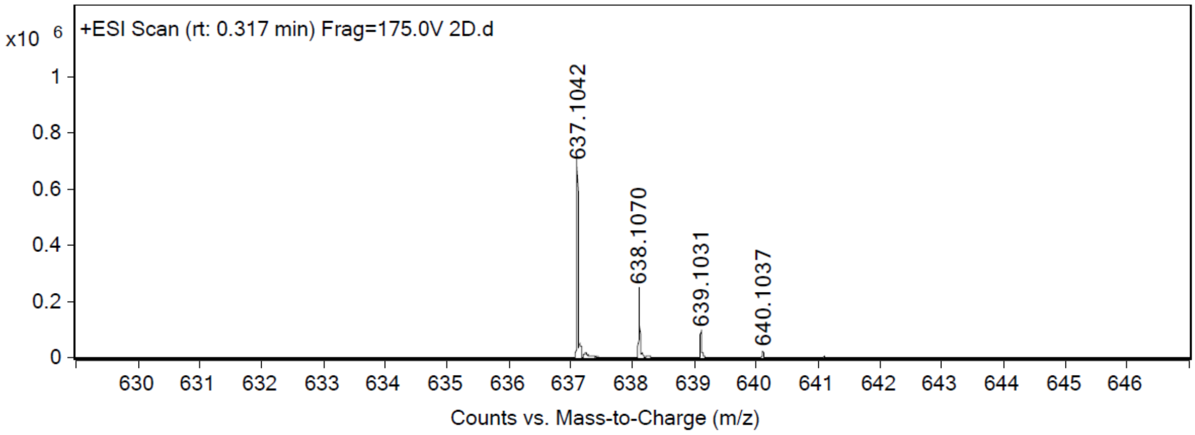


**Supplementary Figure 92.** High resolution ESI-MS spectrum of **2D**.

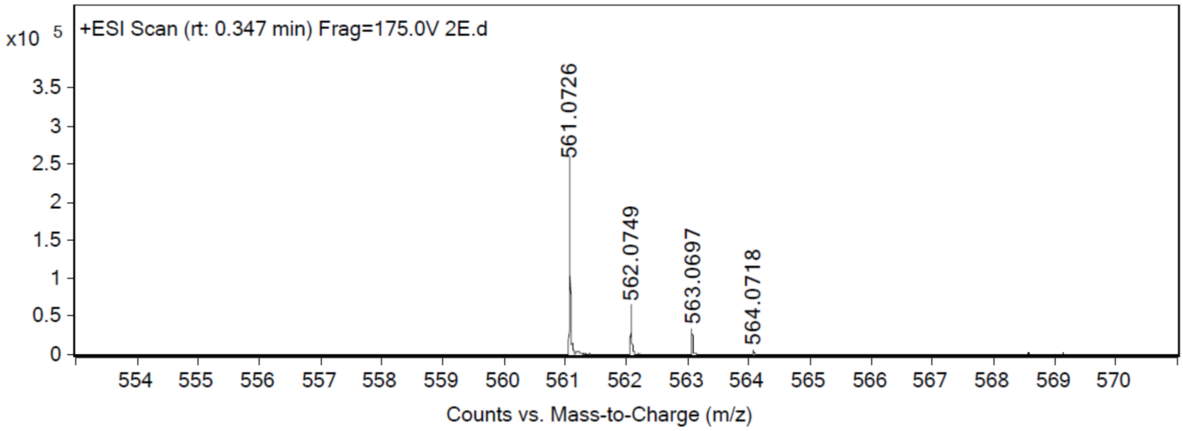


**Supplementary Figure 93.** High resolution ESI-MS spectrum of **2E**.

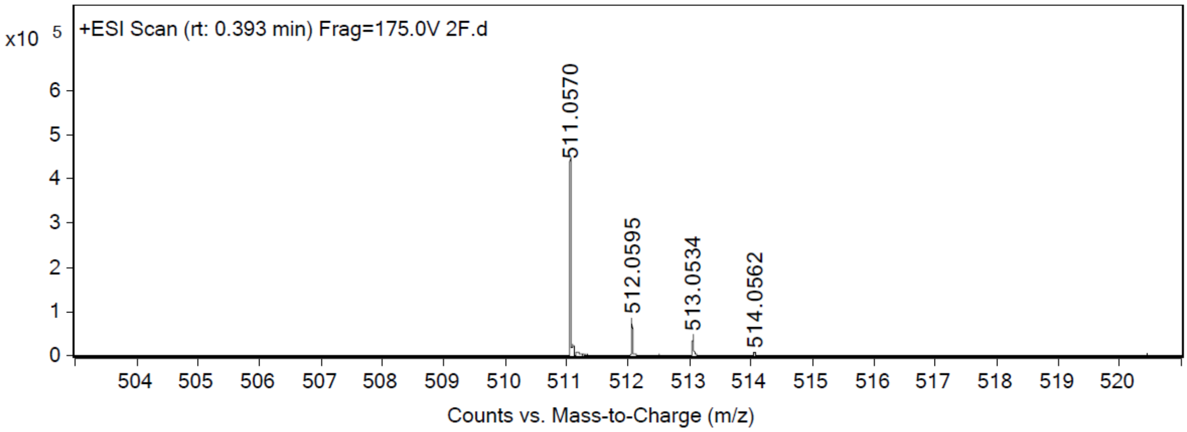


**Supplementary Figure 94.** High resolution ESI-MS spectrum of **2F**.

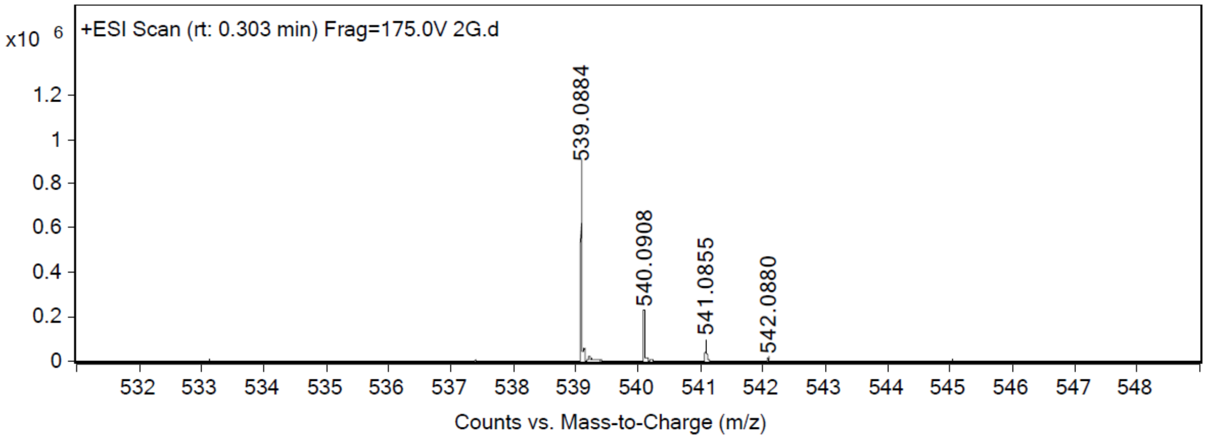


**Supplementary Figure 95.** High resolution ESI-MS spectrum of **2G**.

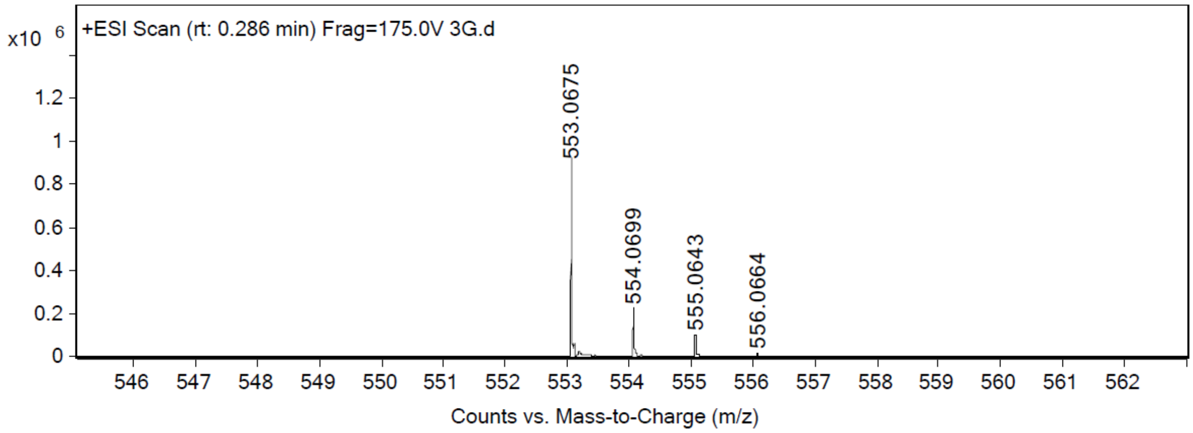


**Supplementary Figure 96.** High resolution ESI-MS spectrum of **3G**.

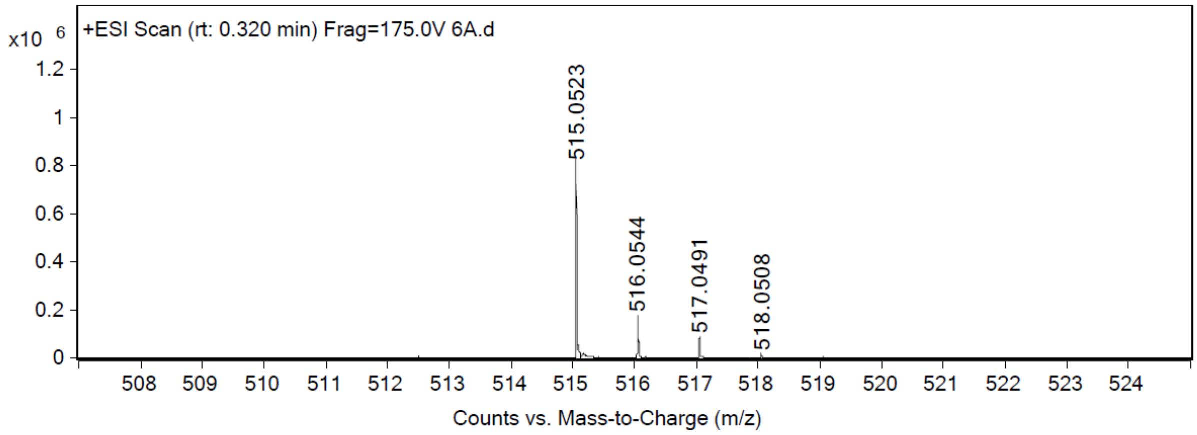


**Supplementary Figure 97.** High resolution ESI-MS spectrum of **4A**.

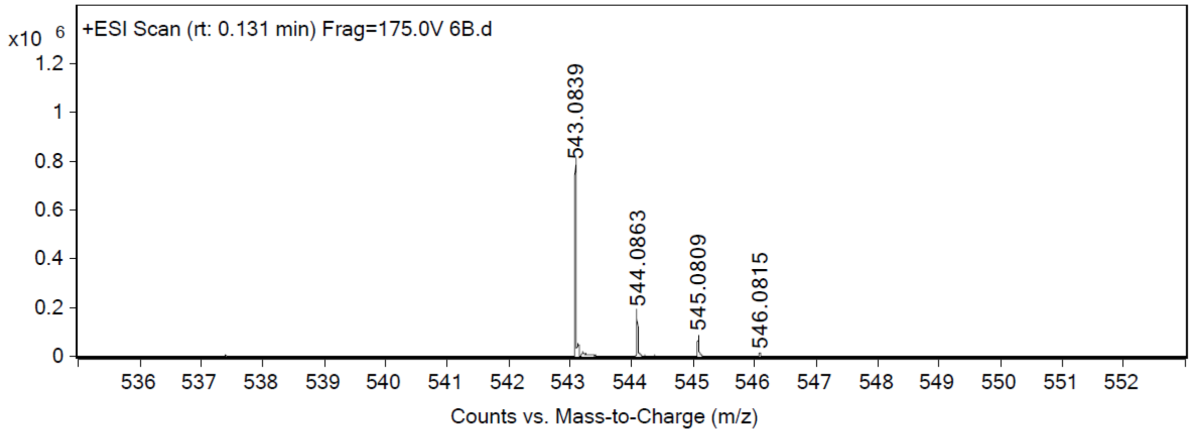


**Supplementary Figure 98.** High resolution ESI-MS spectrum of **4B**.

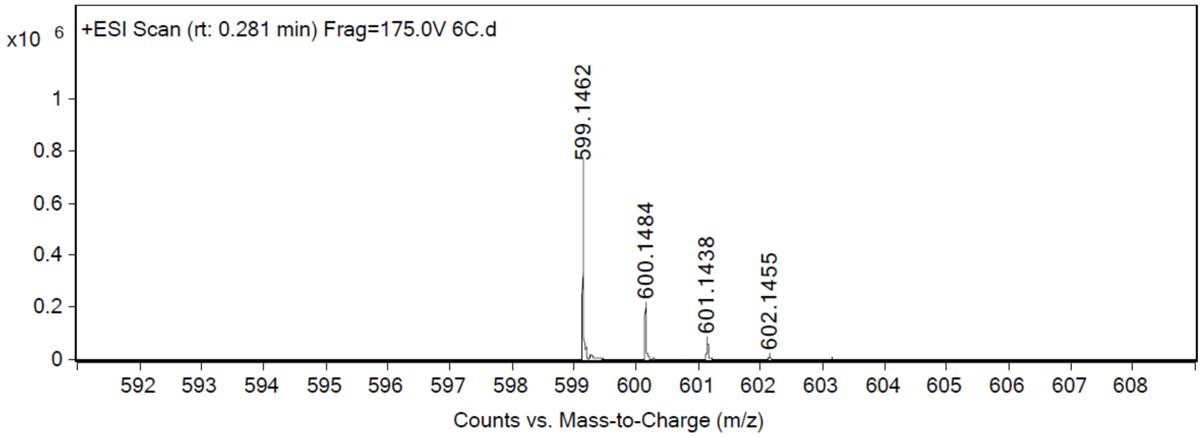


**Supplementary Figure 99.** High resolution ESI-MS spectrum of **4C**.

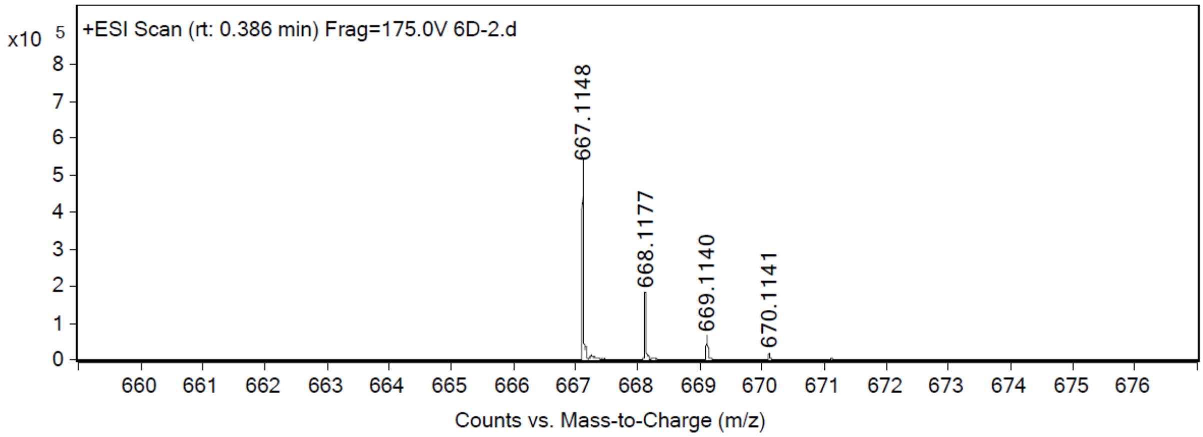


**Supplementary Figure 100.** High resolution ESI-MS spectrum of **4D**.

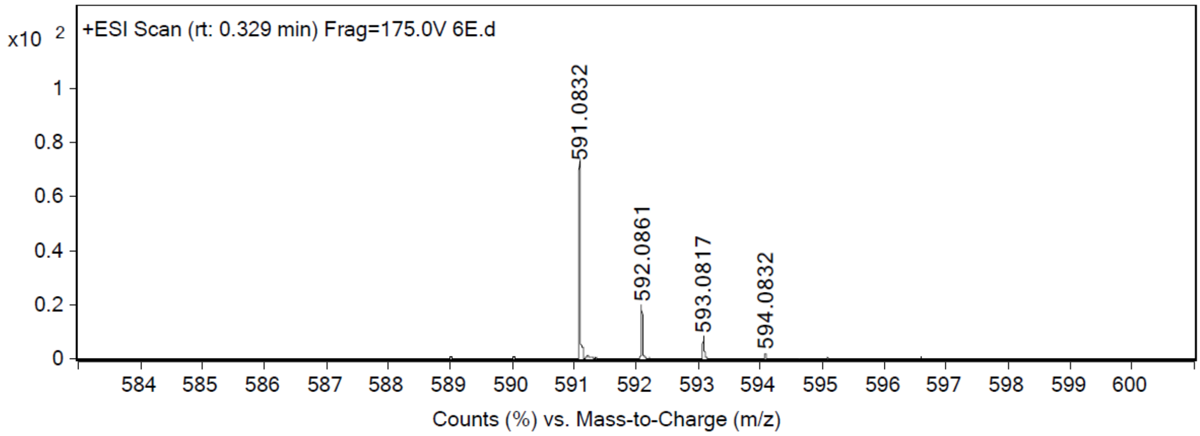


**Supplementary Figure 101.** High resolution ESI-MS spectrum of **4E**.

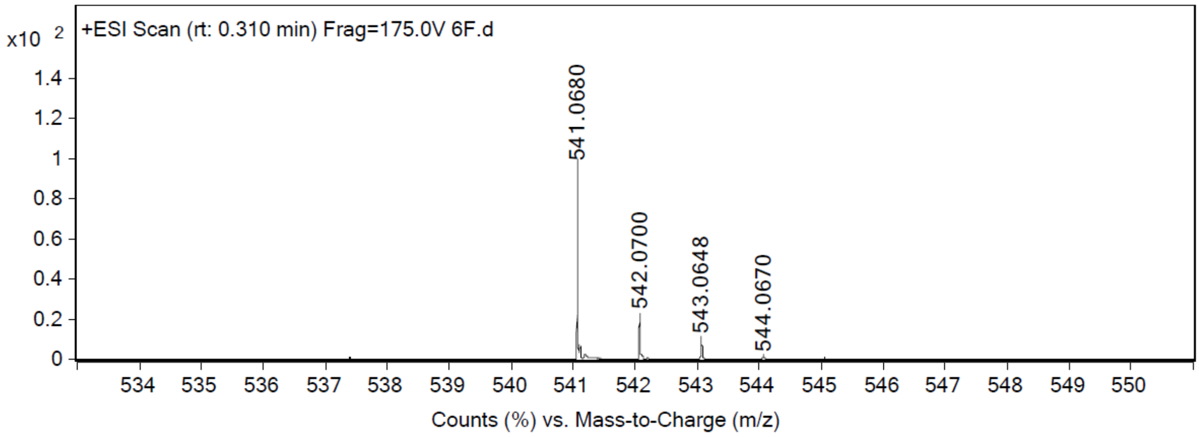


**Supplementary Figure 102.** High resolution ESI-MS spectrum of **4F**.

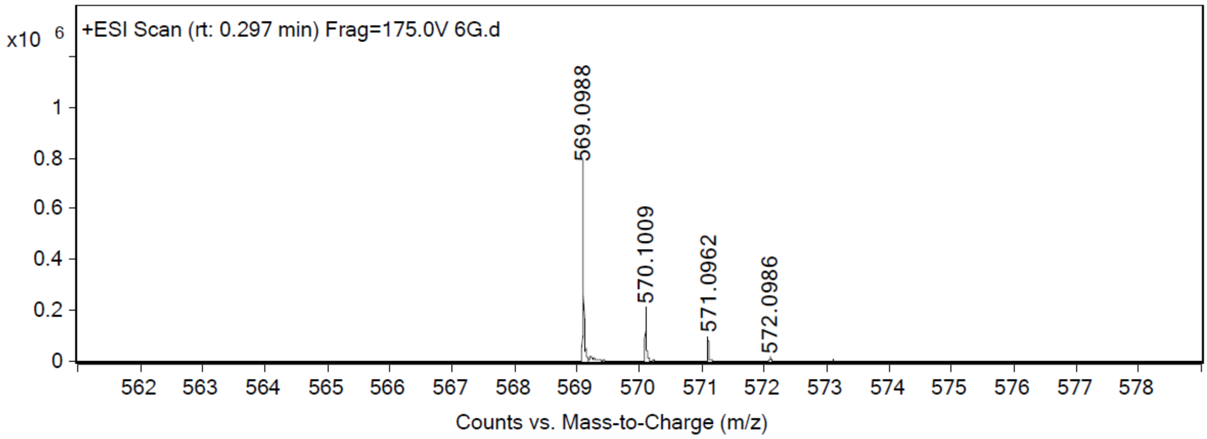


**Supplementary Figure 103.** High resolution ESI-MS spectrum of **4G**.

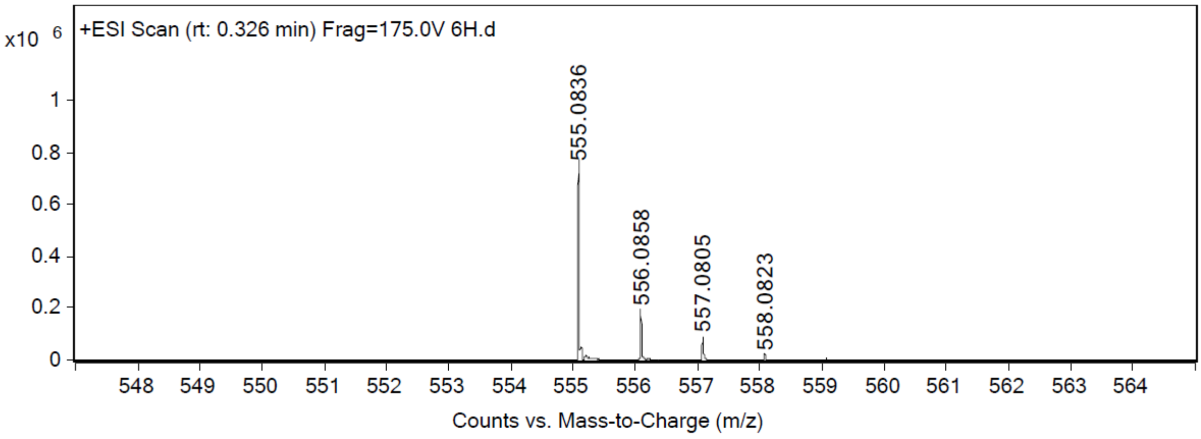


**Supplementary Figure 104.** High resolution ESI-MS spectrum of **4H**.

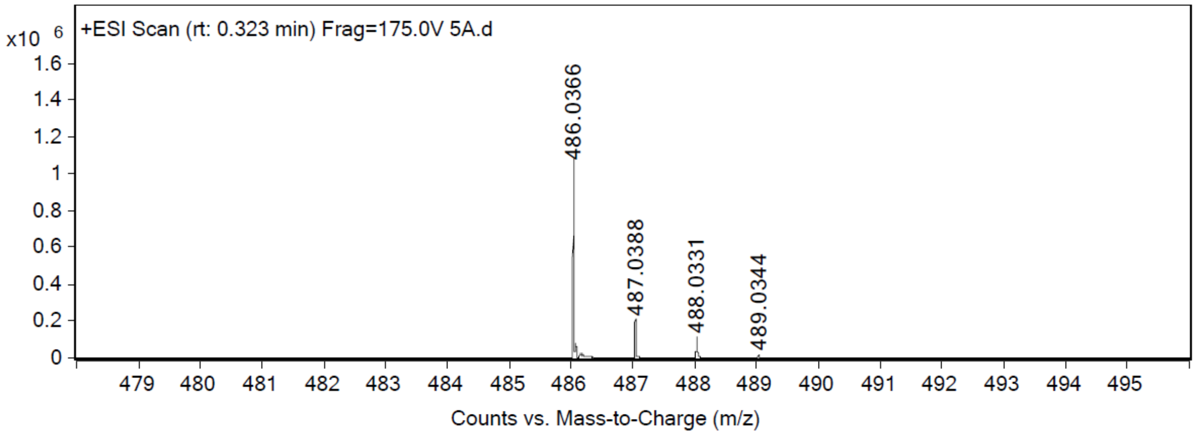


**Supplementary Figure 105.** High resolution ESI-MS spectrum of **5A**.

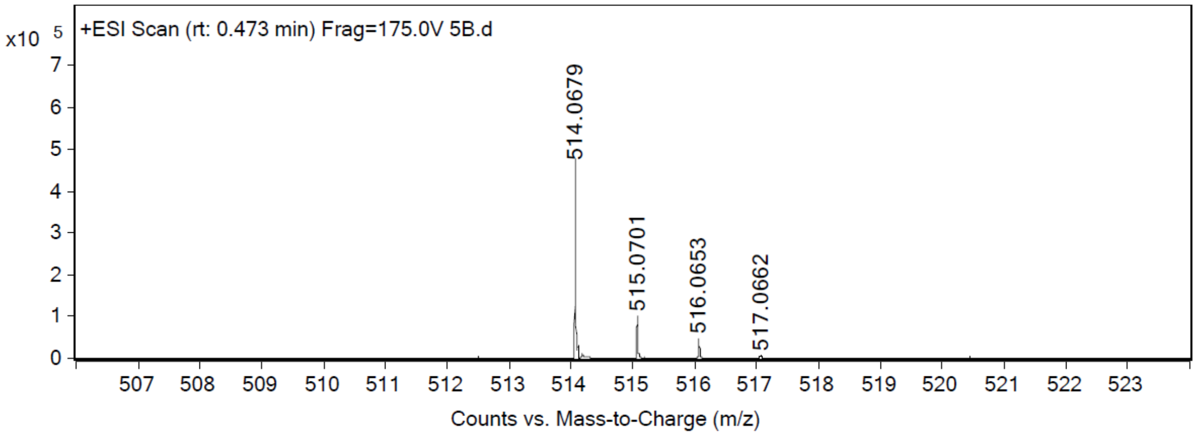


**Supplementary Figure 106.** High resolution ESI-MS spectrum of **5B**.

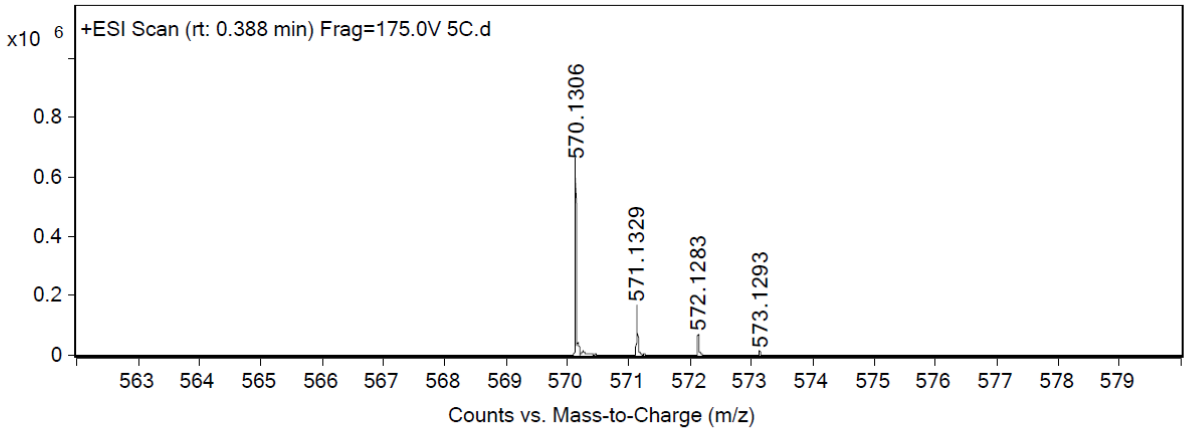


**Supplementary Figure 107.** High resolution ESI-MS spectrum of **5C**.

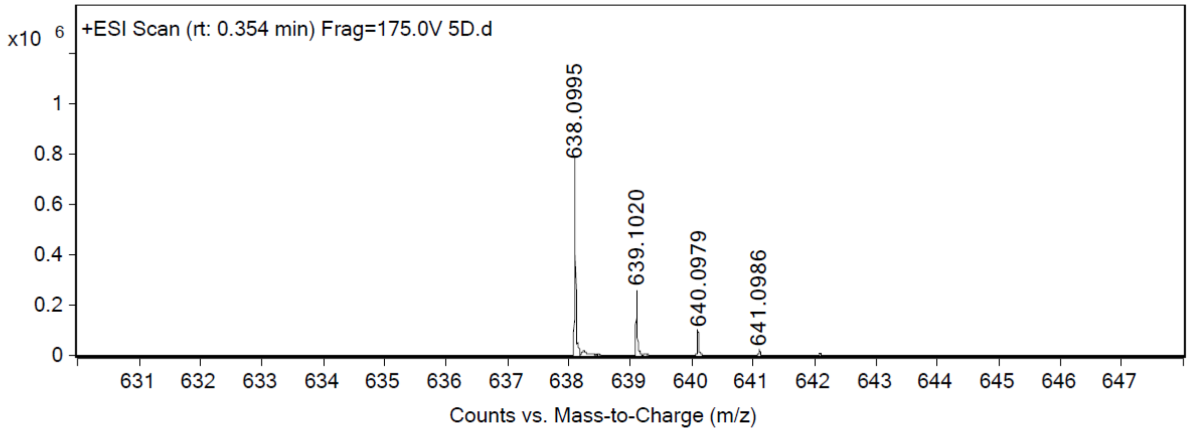


**Supplementary Figure 108.** High resolution ESI-MS spectrum of **5D**.

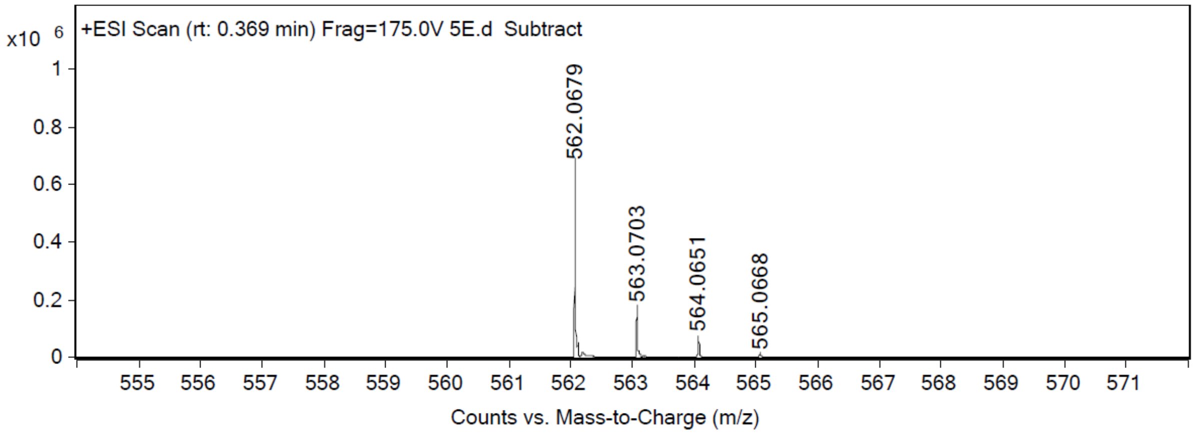


**Supplementary Figure 109.** High resolution ESI-MS spectrum of **5E**.

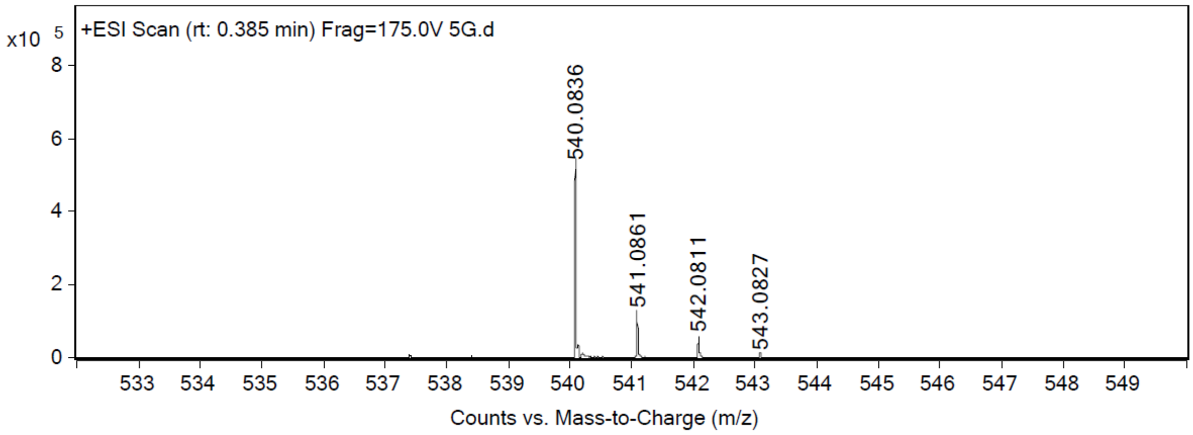


**Supplementary Figure 110.** High resolution ESI-MS spectrum of **5G**.

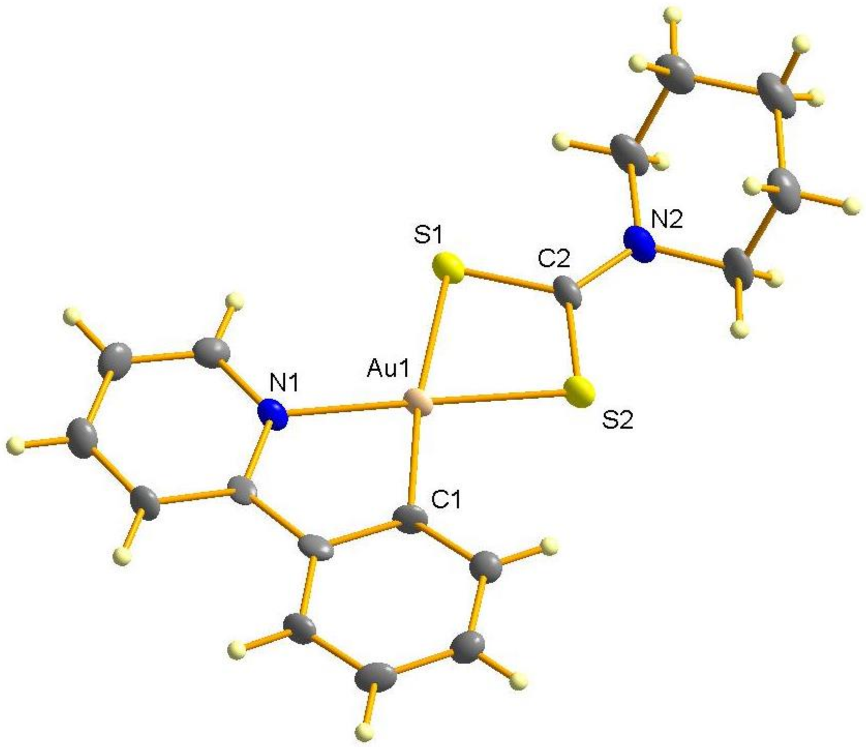


**Supplementary Figure 111.** Molecular structure of **1H**; $PF_6^-$ anion was omitted for clarity. Selected bond lengths (Å) and angles (°): Au1-N1 2.060(5), Au1-C1 2.040(6), Au1-S1 2.399(16), Au1-S2 2.273(16), C2-S1 1.733(7), C2-S2 1.731(7), C2-N2 1.312(8), N1-Au1-S1 106.15(15), N1-Au1-C1 81.0(2), C1-Au1-S2 97.55(19), S1-Au1-S2 75.28(6), S1-C2-S2 111.3(6).

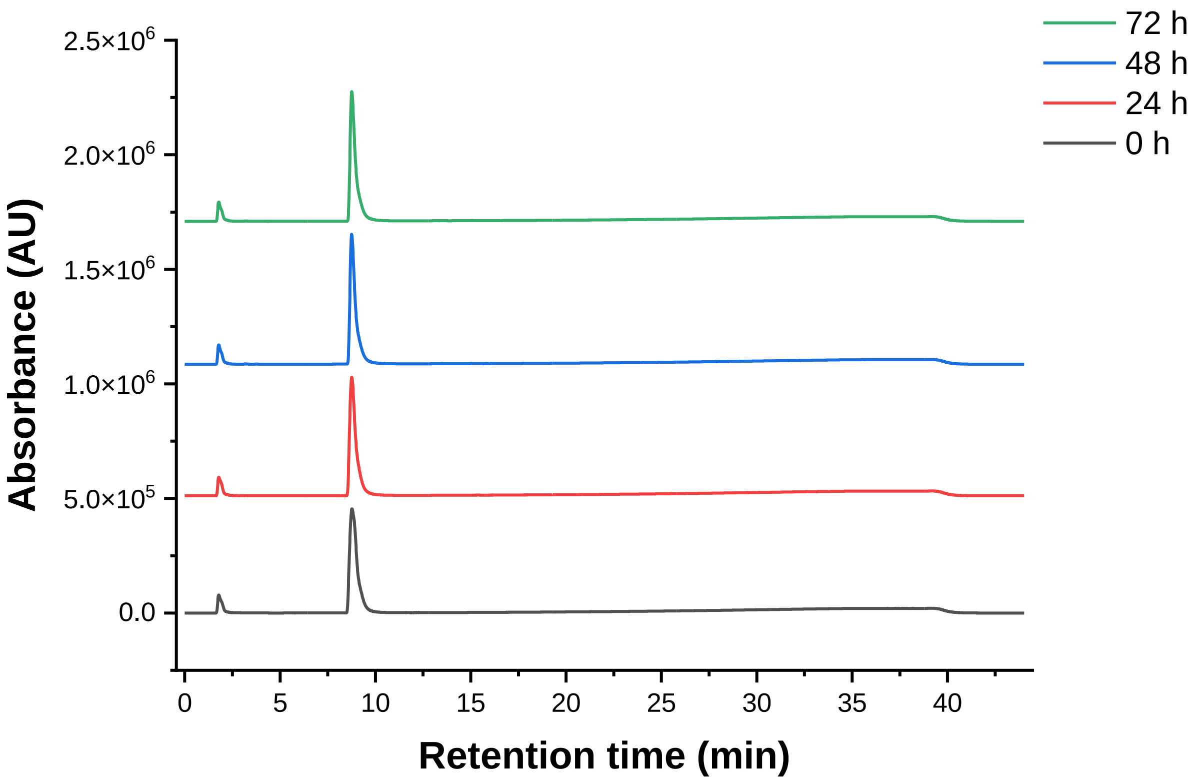


**Supplementary Figure 112**. Stability of **1A** in water/DMSO 1:1 using RP-HPLC.

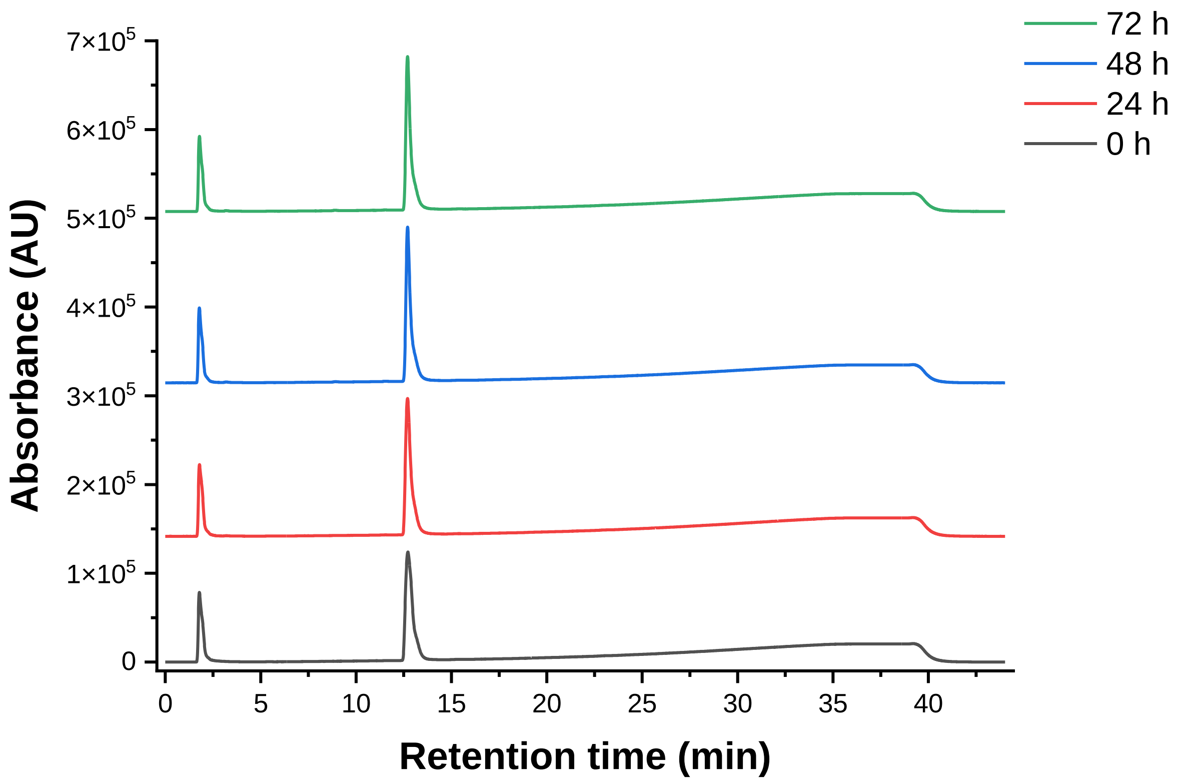


**Supplementary Figure 113**. Stability of **1B** in water/DMSO 1:1 using RP-HPLC.

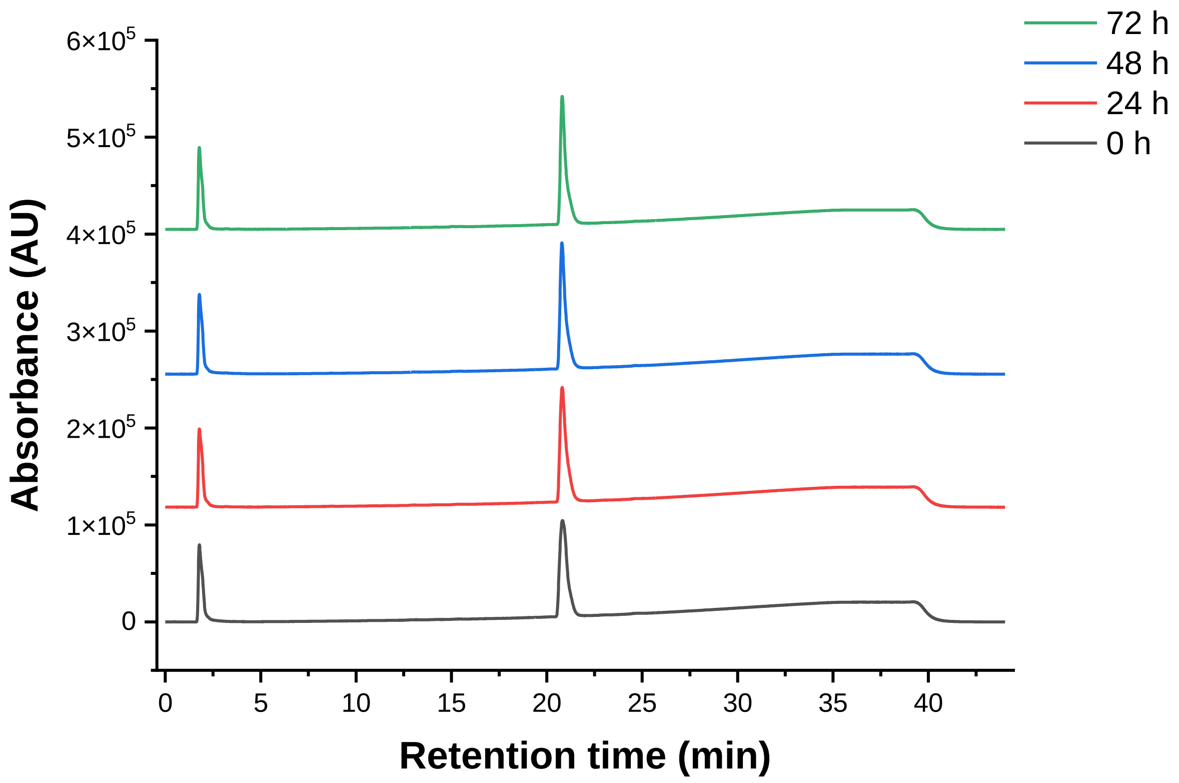


**Supplementary Figure 114**. Stability of **1C** in water/DMSO 1:1 using RP-HPLC.

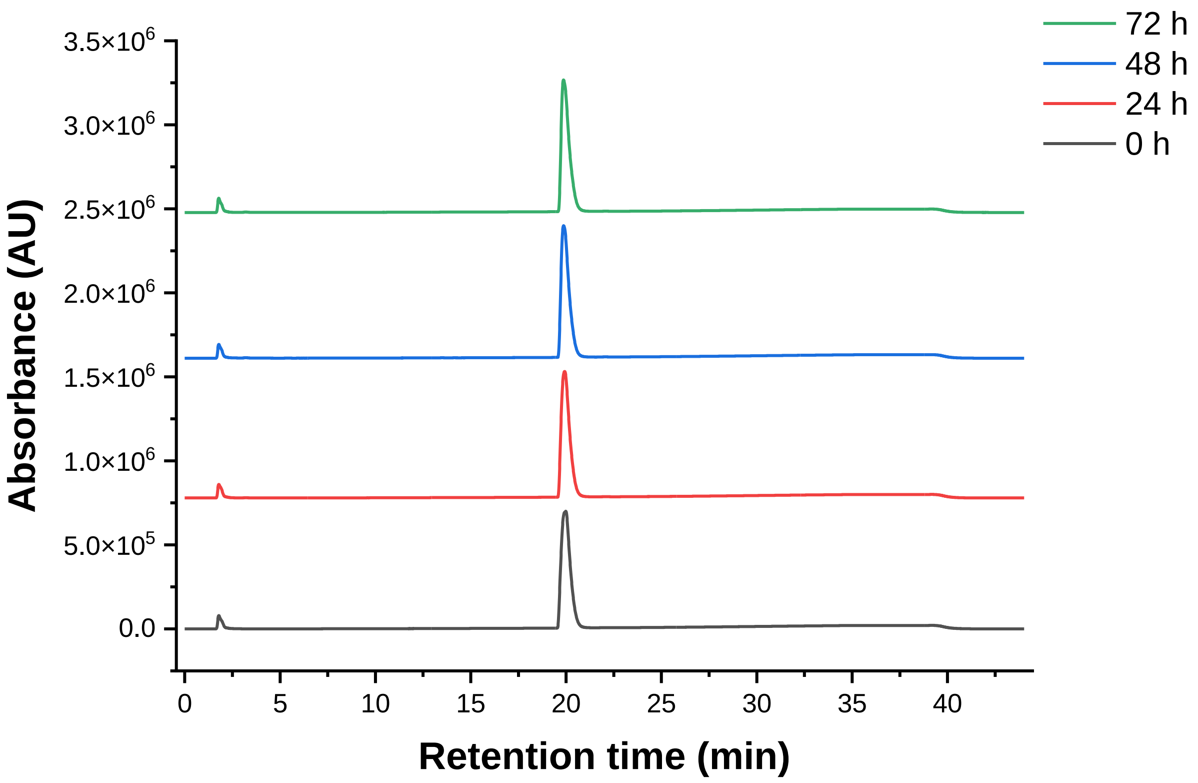


**Supplementary Figure 115**. Stability of **1D** in water/DMSO 1:1 using RP-HPLC.

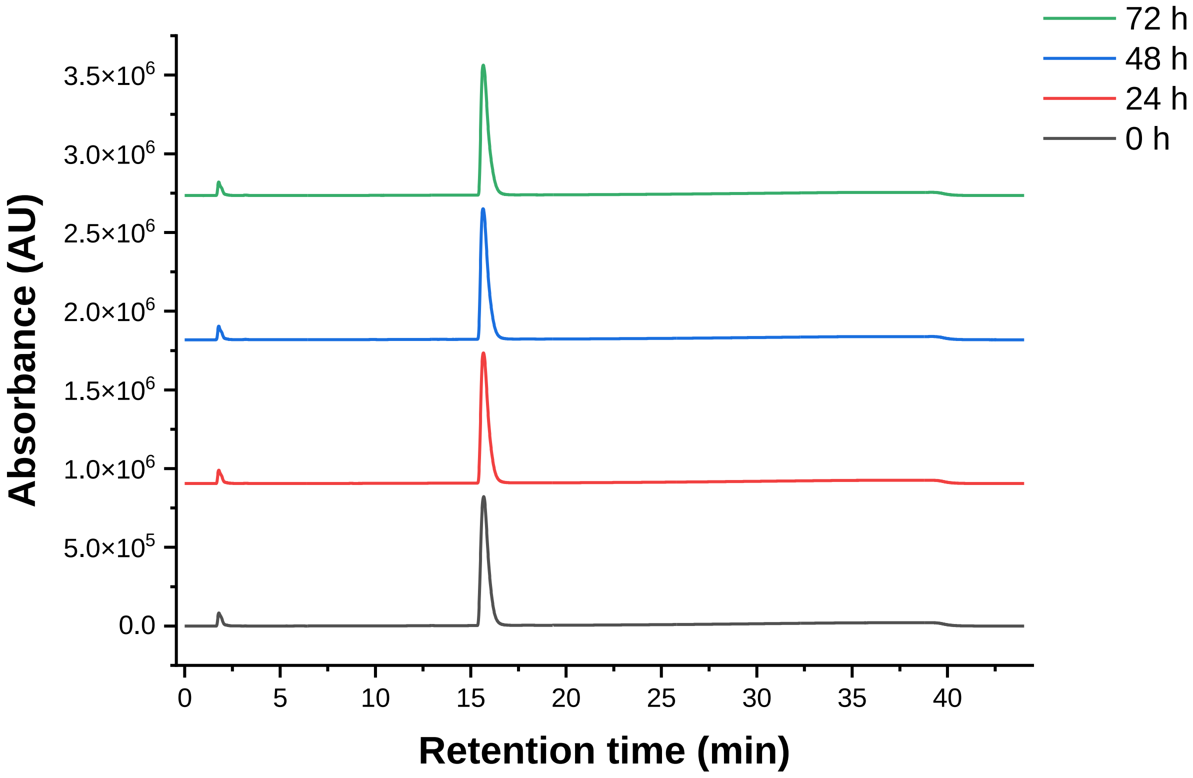


**Supplementary Figure 116**. Stability of **1E** in water/DMSO 1:1 using RP-HPLC.

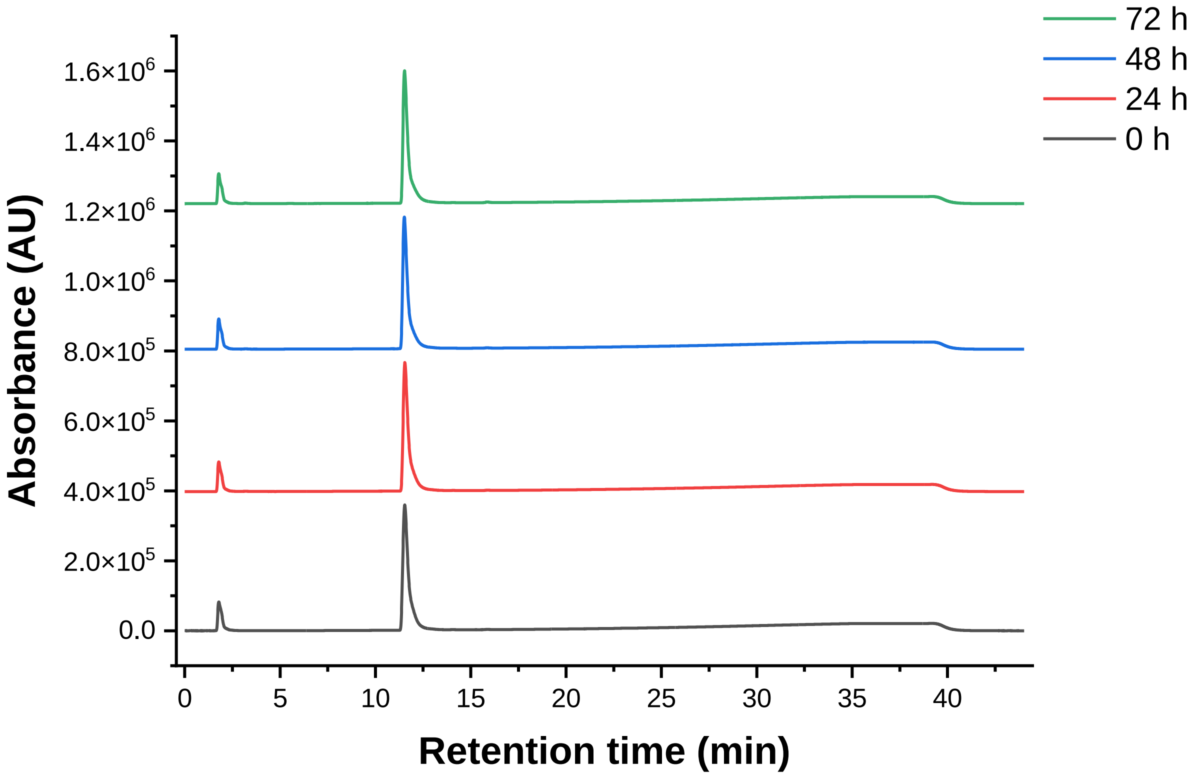


**Supplementary Figure 117.** Stability of **1F** in water/DMSO 1:1 using RP-HPLC.

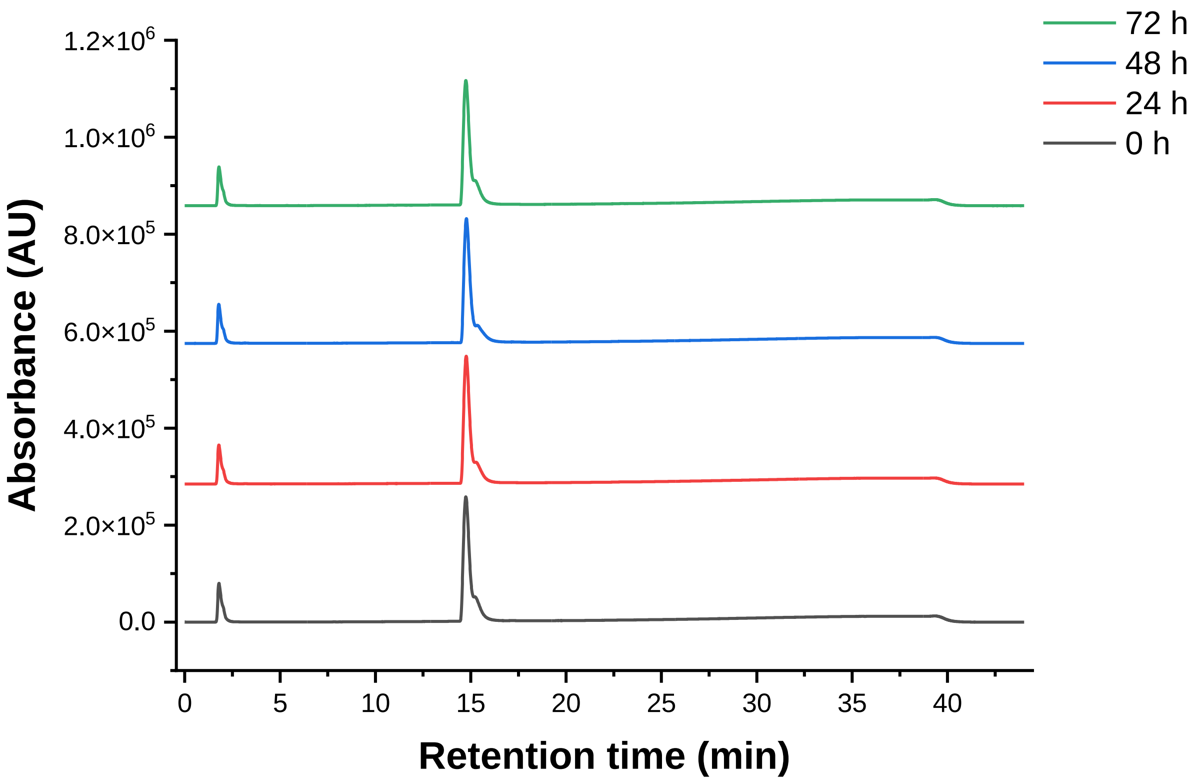


**Supplementary Figure 118**. Stability of **1G** in water/DMSO 1:1 using RP-HPLC.

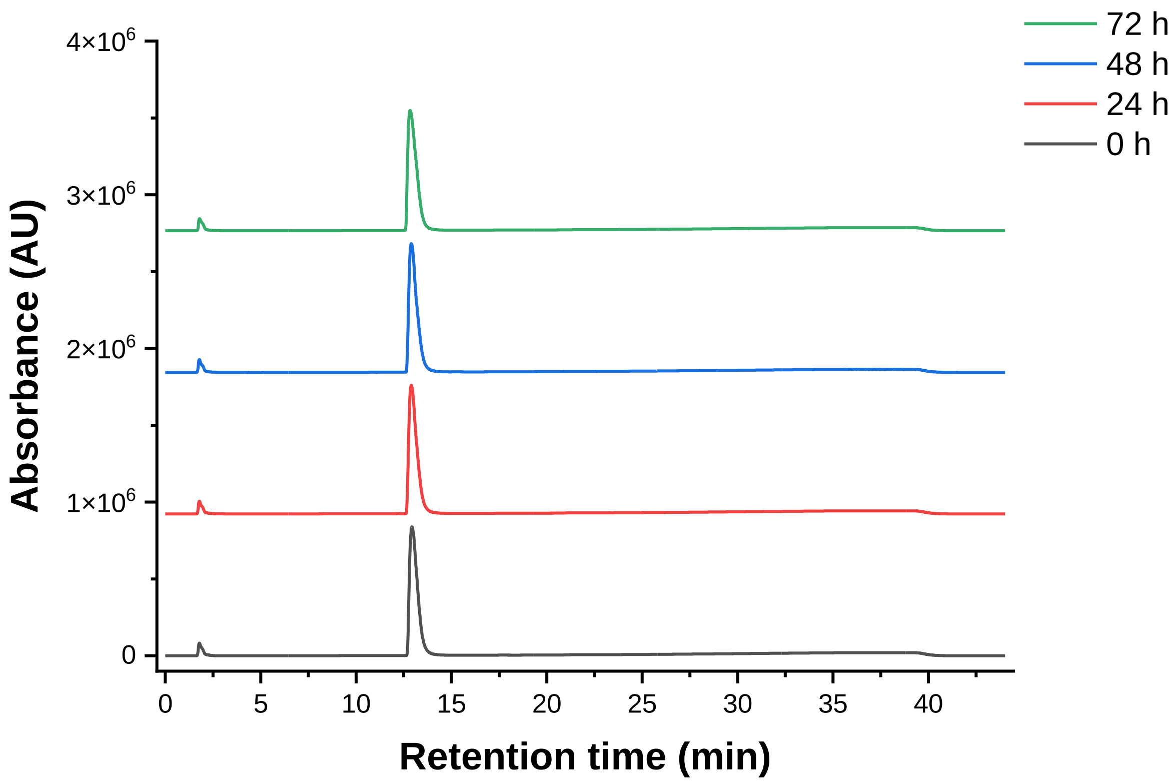


**Supplementary Figure 119**. Stability of **1H** in water/DMSO 1:1 using RP-HPLC.

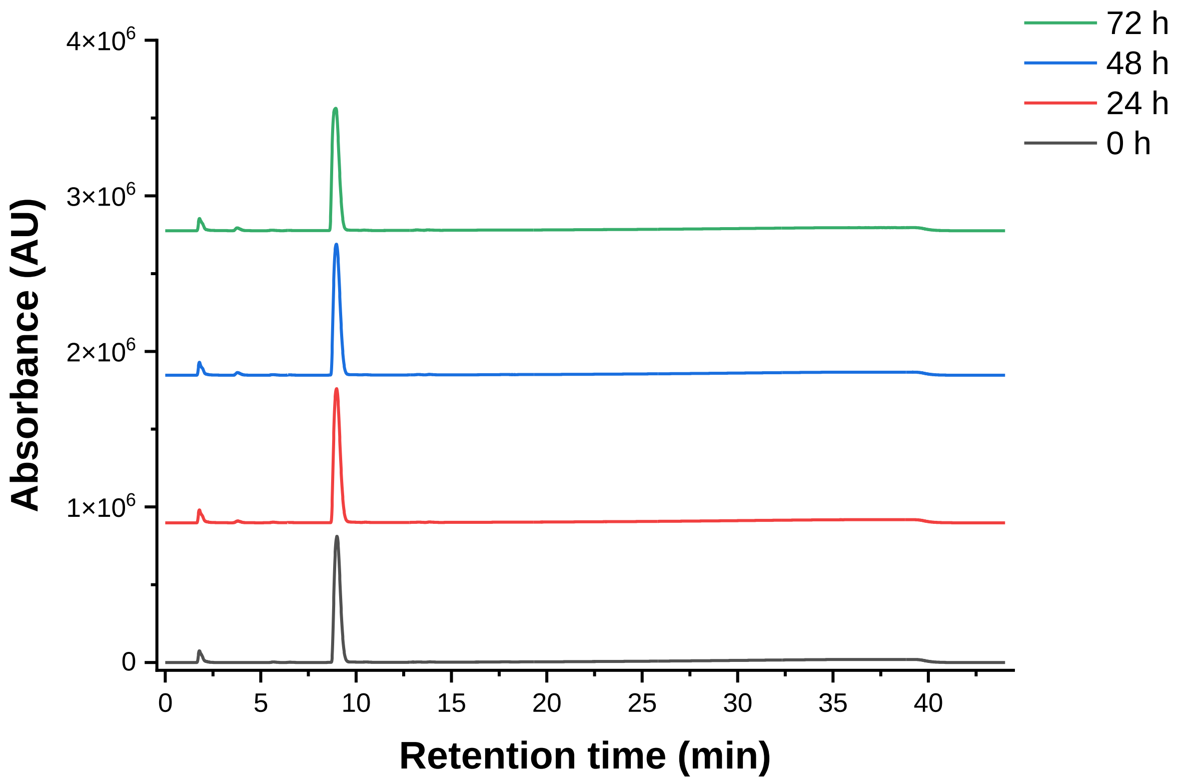


**Supplementary Figure 120**. Stability of **2A** in water/DMSO 1:1 using RP-HPLC.

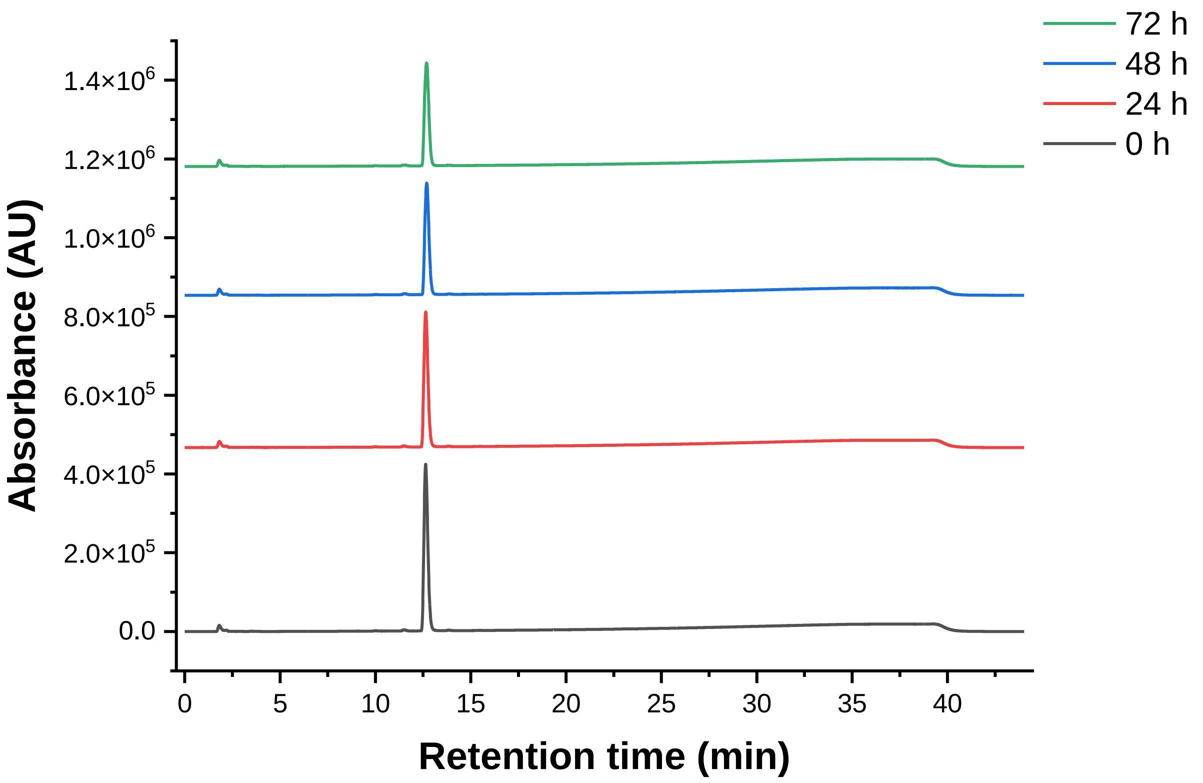


**Supplementary Figure 121**. Stability of **2B** in water/DMSO 1:1 using RP-HPLC.

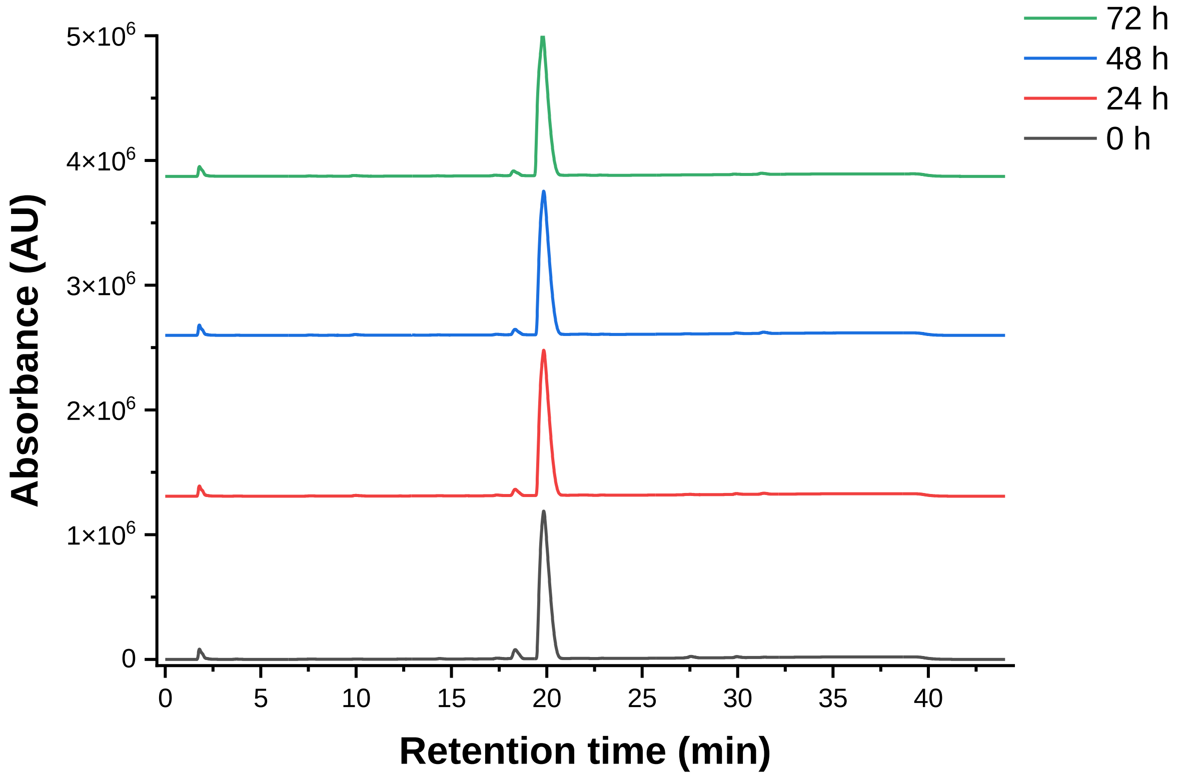


**Supplementary Figure 122**. Stability of **2C** in water/DMSO 1:1 using RP-HPLC.

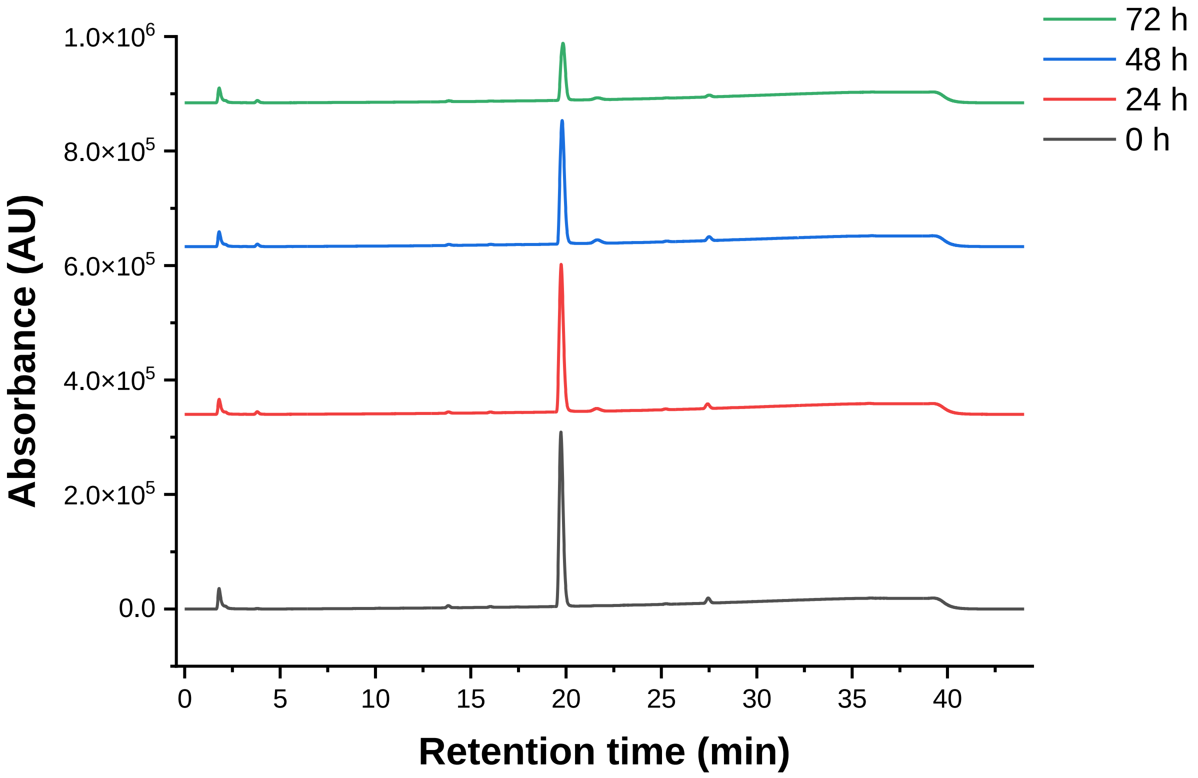


**Supplementary Figure 123.** Stability of **2D** in water/DMSO 1:1 using RP-HPLC.

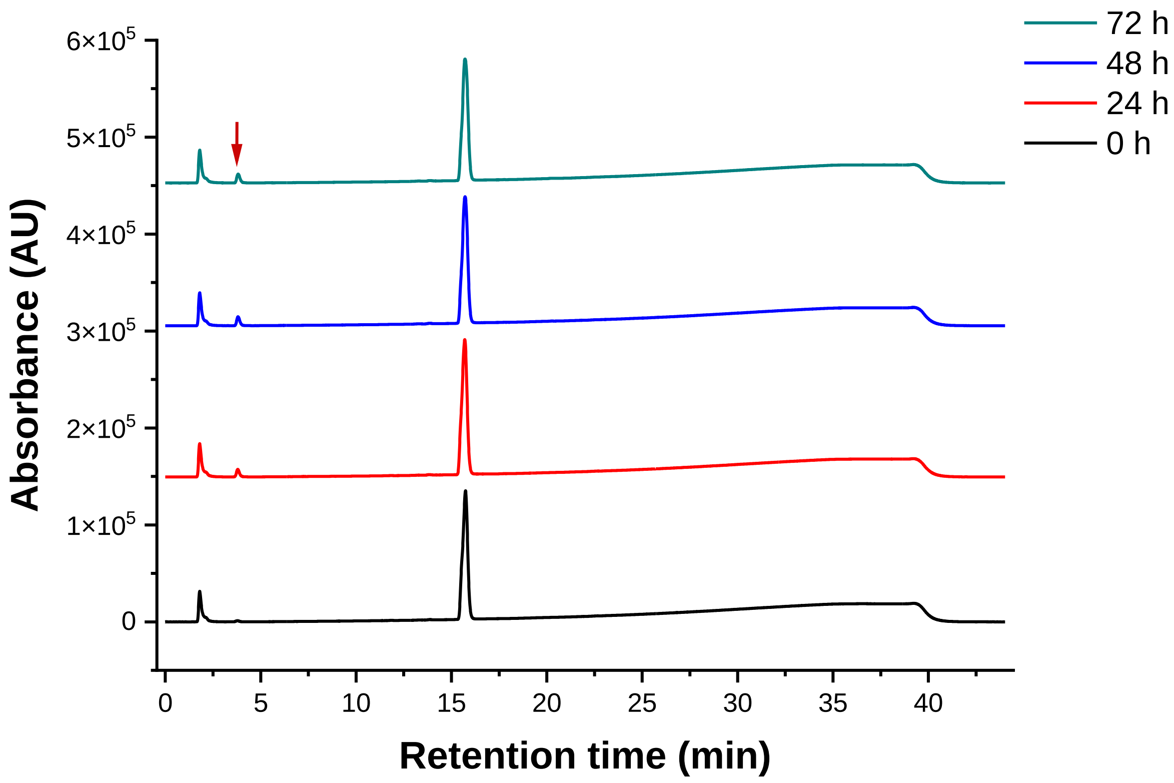


**Supplementary Figure 124**. Stability of **2E** in water/DMSO 1:1 using RP-HPLC.

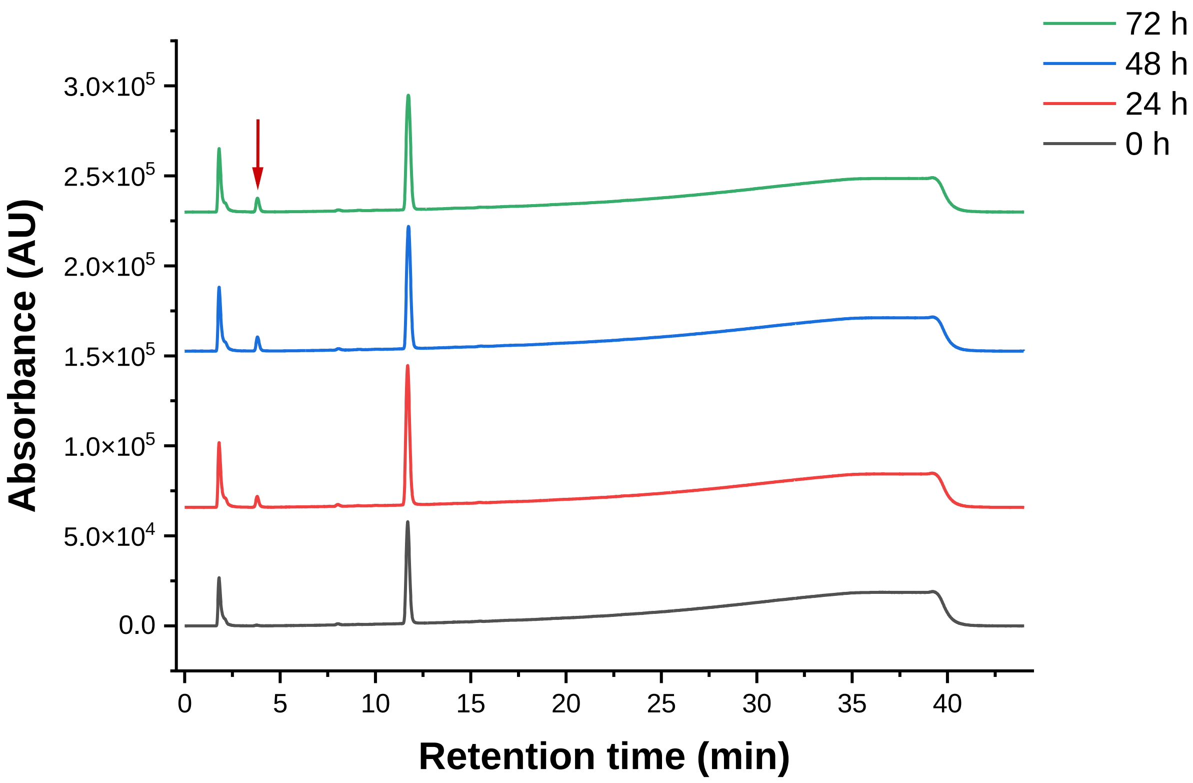


**Supplementary Figure 125**. Stability of **2F** in water/DMSO 1:1 using RP-HPLC.

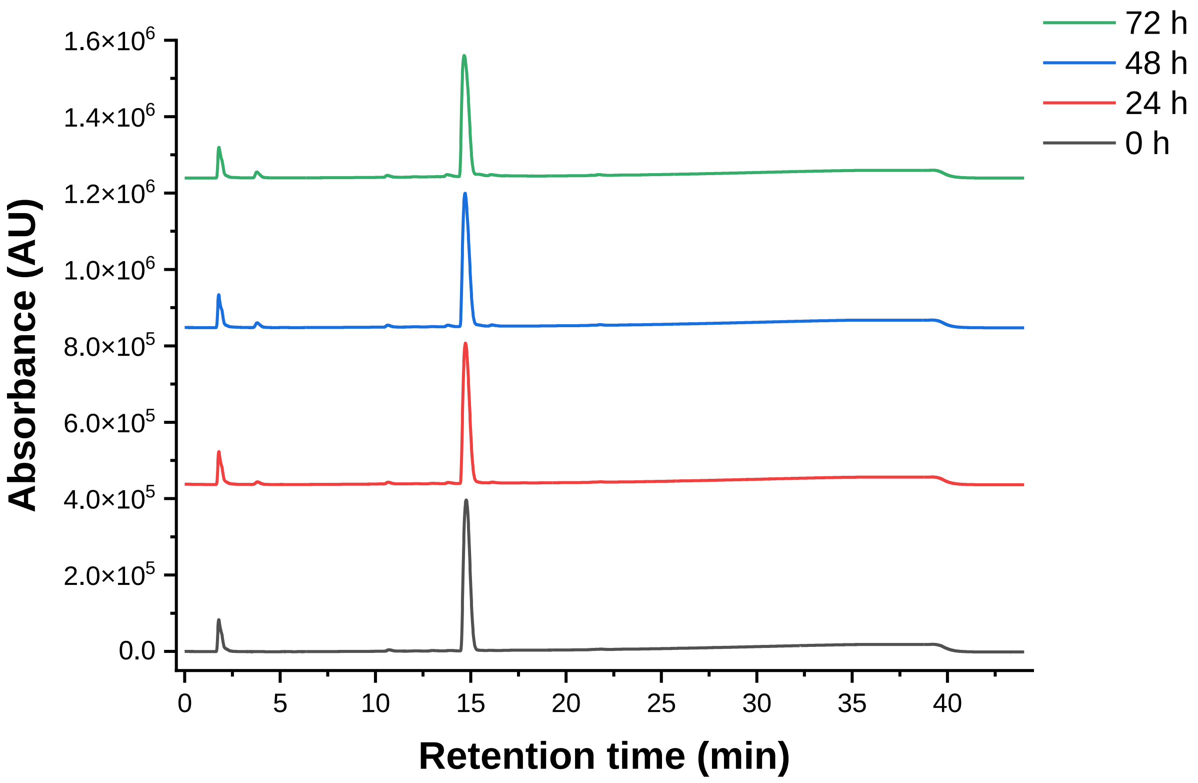


**Supplementary Figure 126**. Stability of **2G** in water/DMSO 1:1 using RP-HPLC.

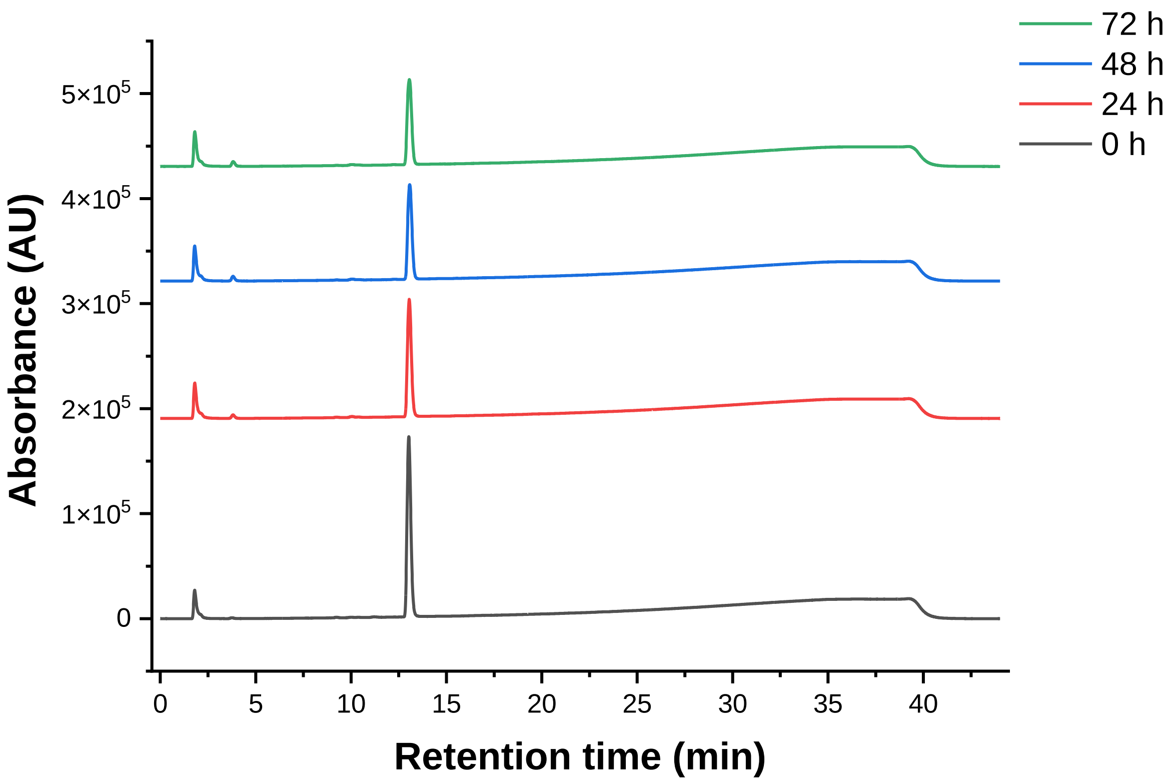


**Supplementary Figure 127**. Stability of **2H** in water/DMSO 1:1 using RP-HPLC.

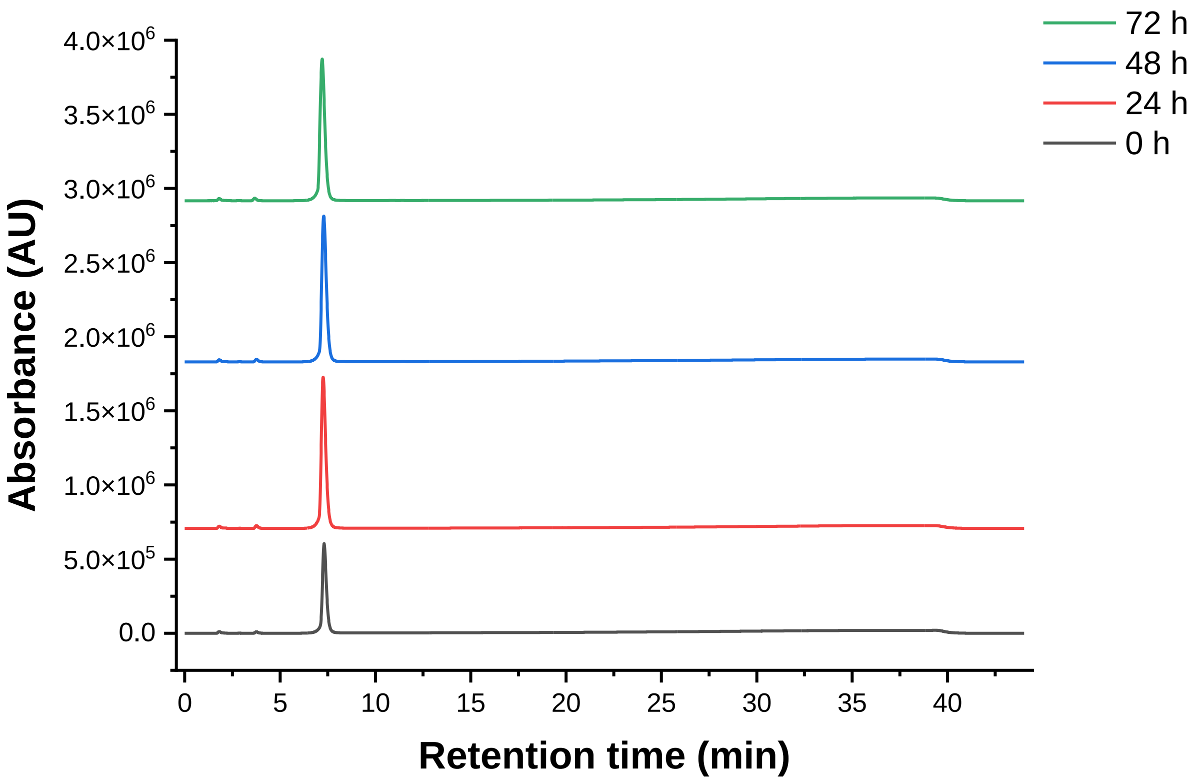


**Supplementary Figure 128**. Stability of **3A** in water/DMSO 1:1 using RP-HPLC.

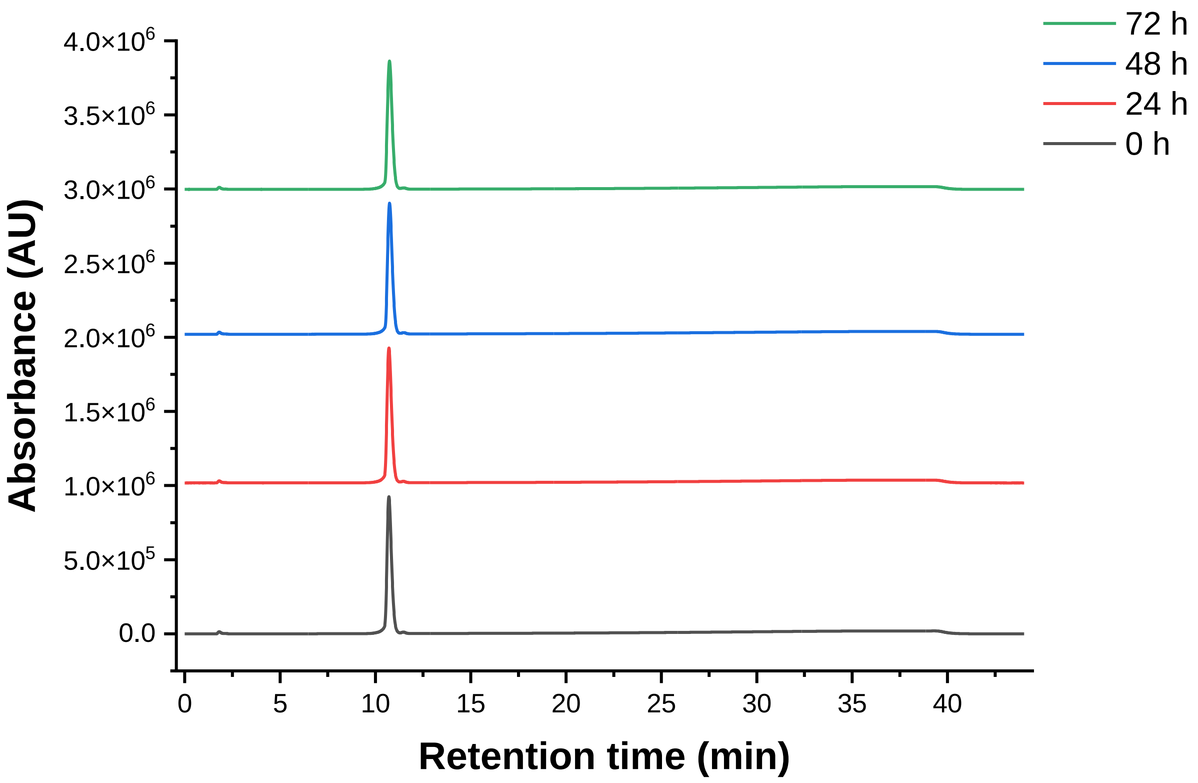


**Supplementary Figure 129**. Stability of **3B** in water/DMSO 1:1 using RP-HPLC.

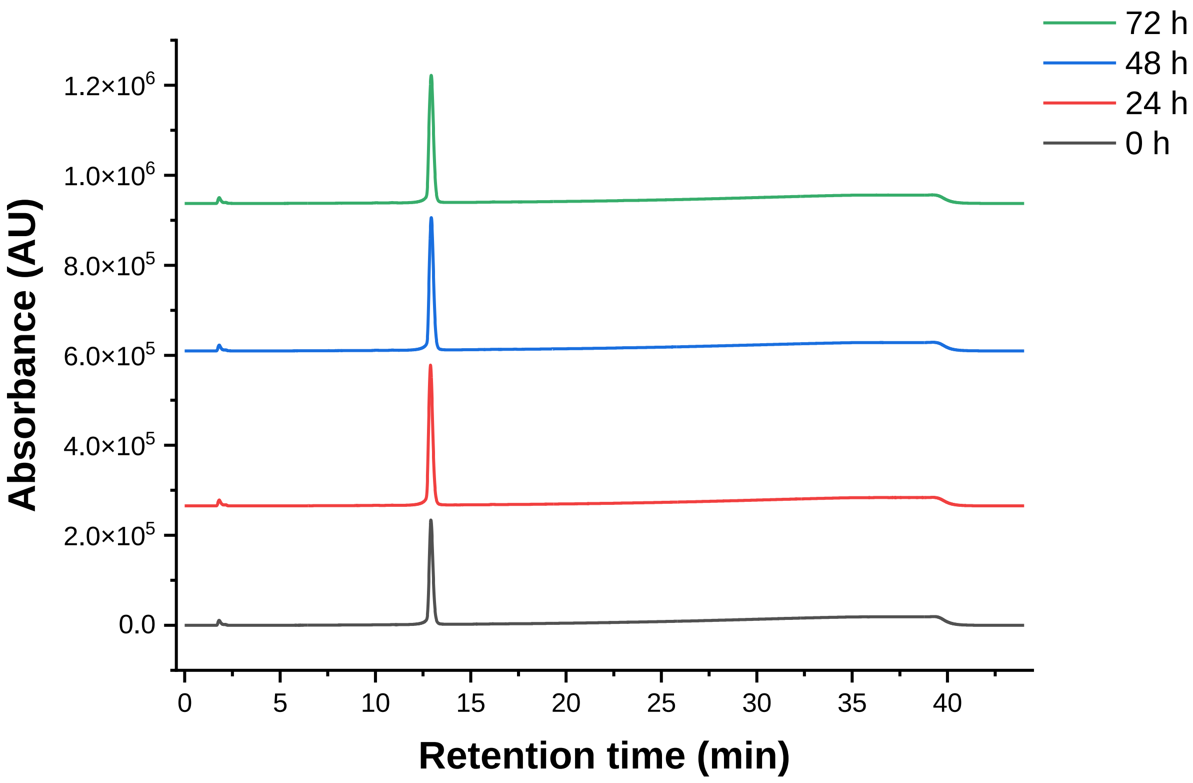


**Supplementary Figure 130.** Stability of **3G** in water/DMSO 1:1 using RP-HPLC.

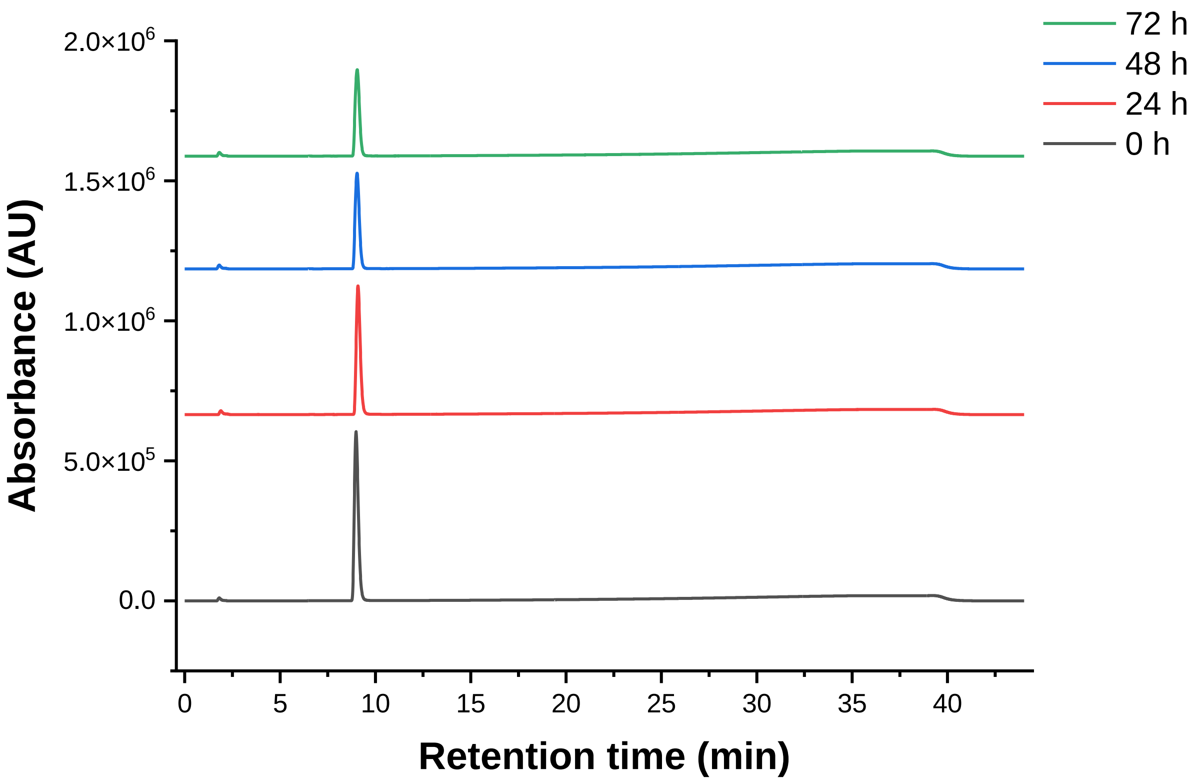


**Supplementary Figure 131**. Stability of **4A** in water/DMSO 1:1 using RP-HPLC.

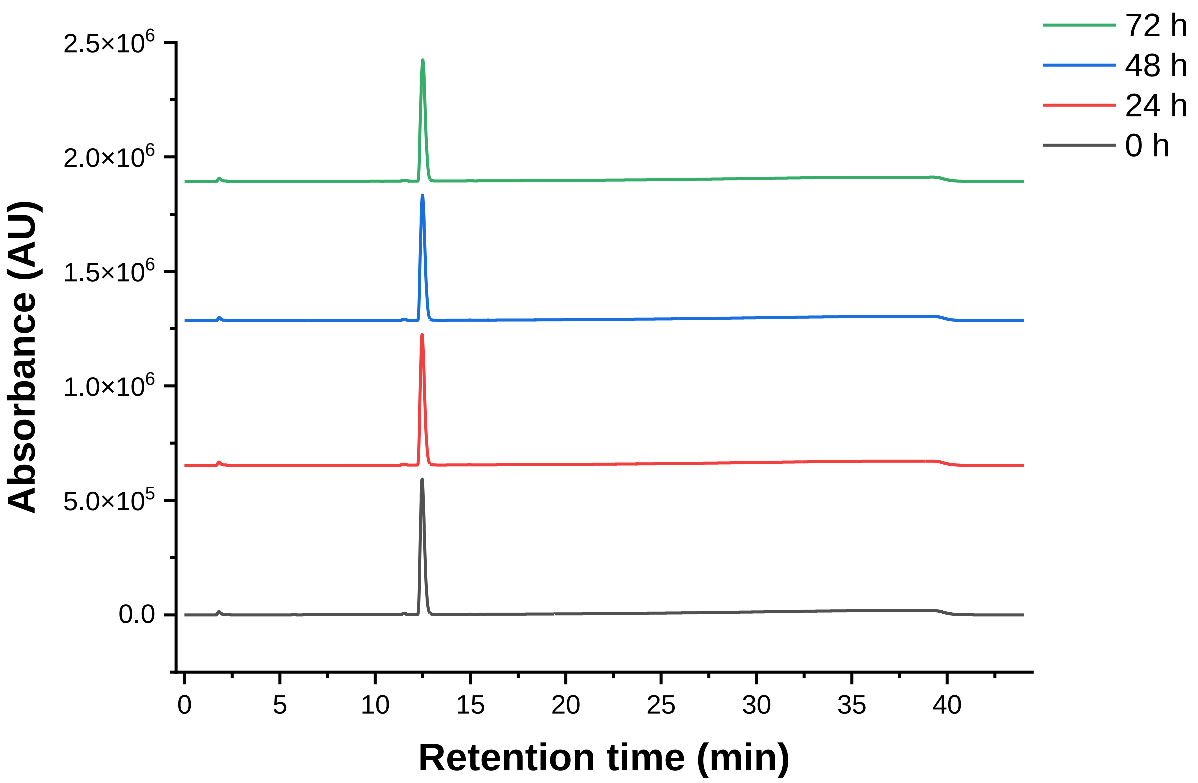


**Supplementary Figure 132**. Stability of **4B** in water/DMSO 1:1 using RP-HPLC.

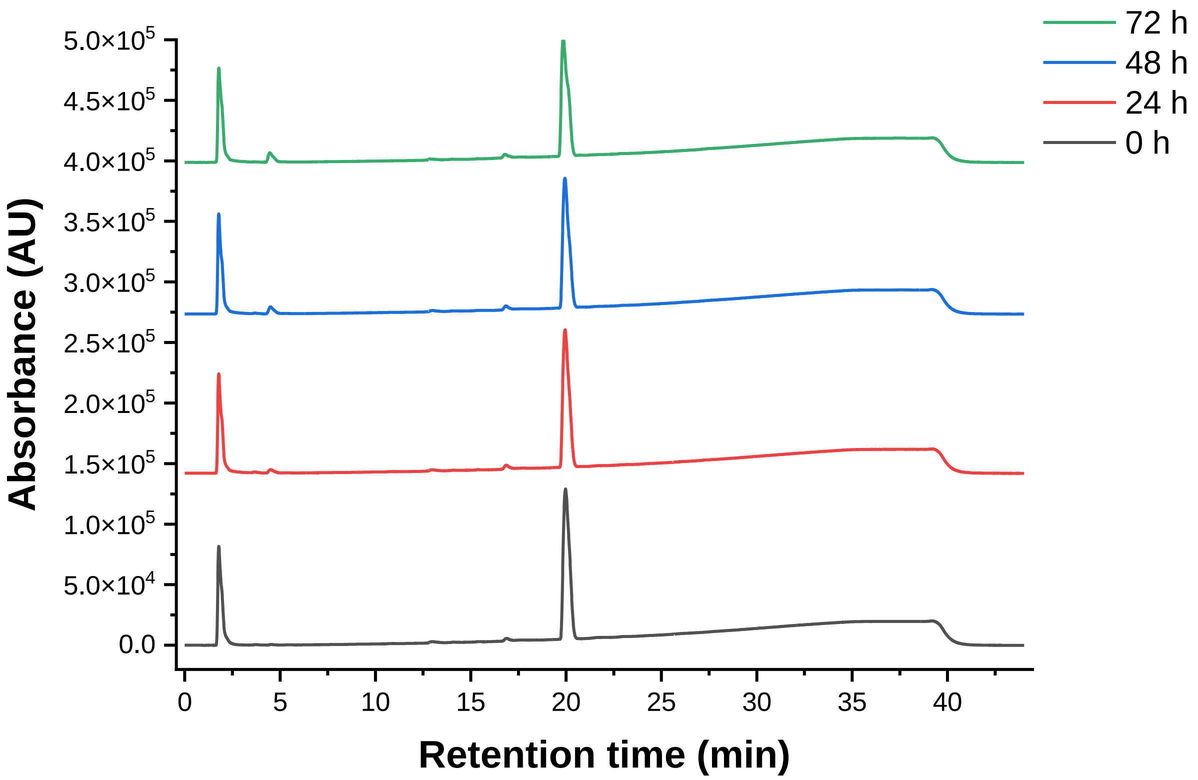


**Supplementary Figure 133**. Stability of **4C** in water/DMSO 1:1 using RP-HPLC.

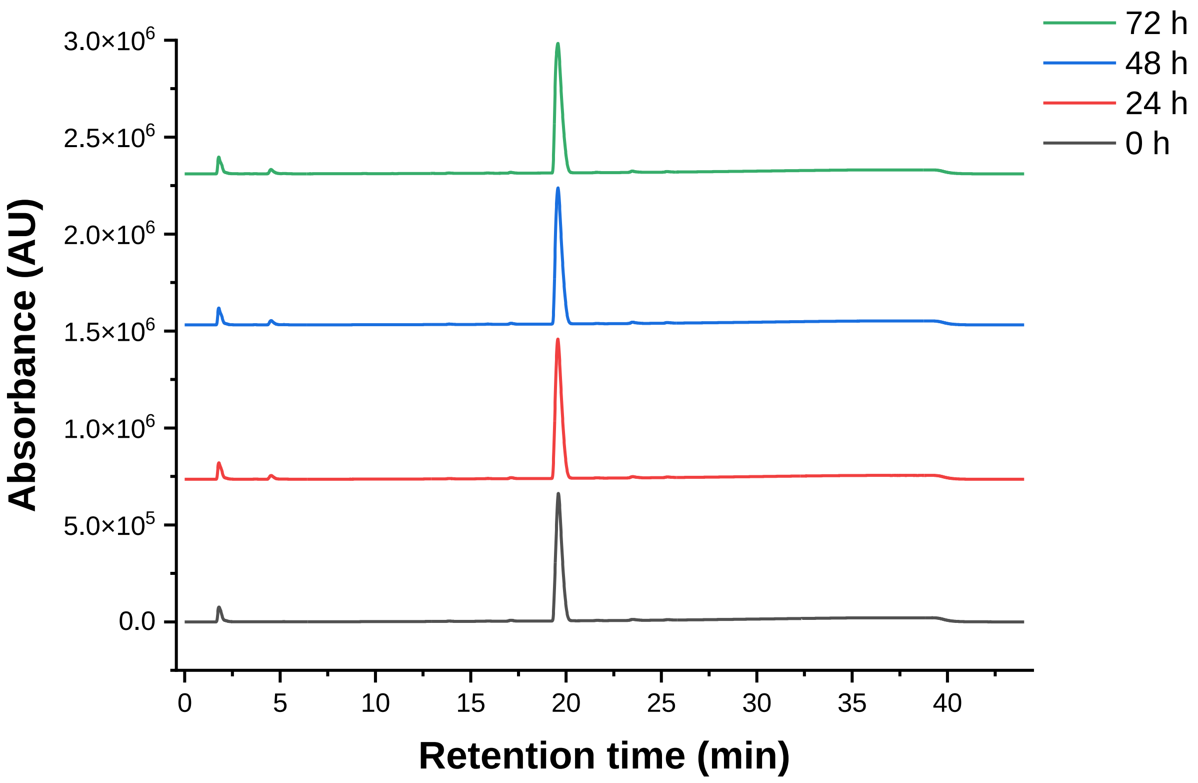


**Supplementary Figure 134**. Stability of **4D** in water/DMSO 1:1 using RP-HPLC.

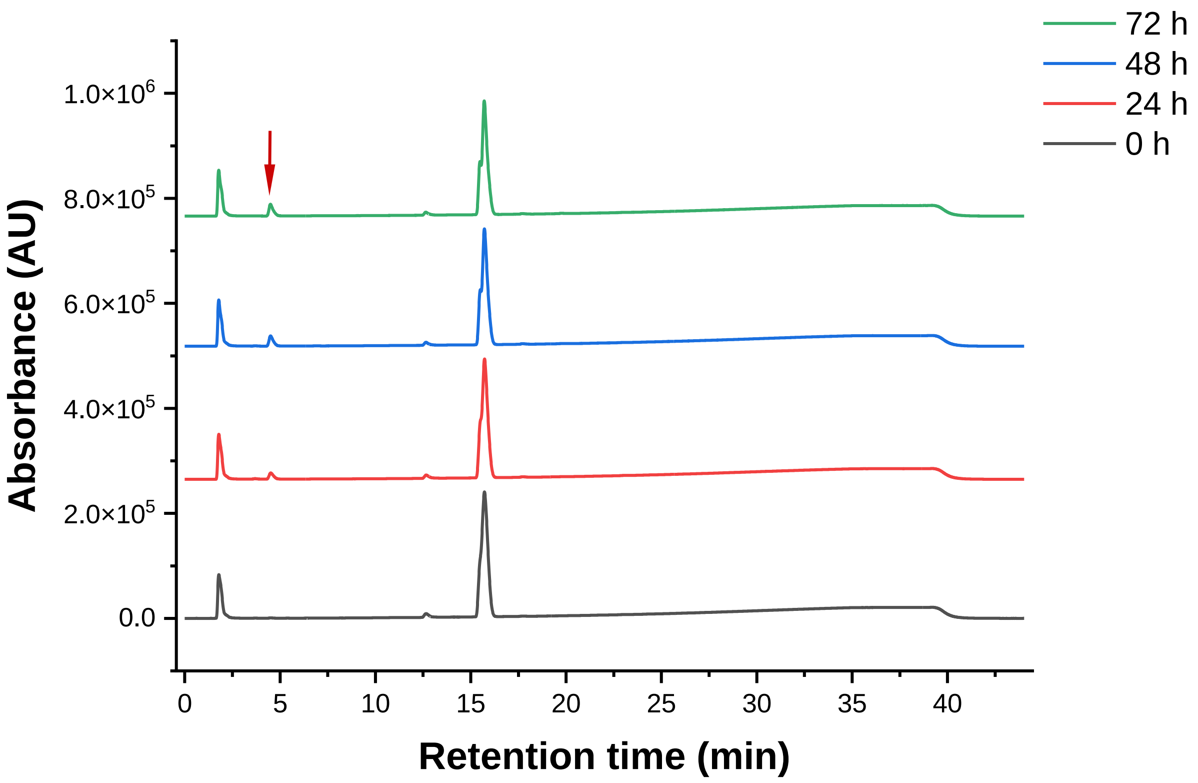


**Supplementary Figure 135**. Stability of **4E** in water/DMSO 1:1 using RP-HPLC.

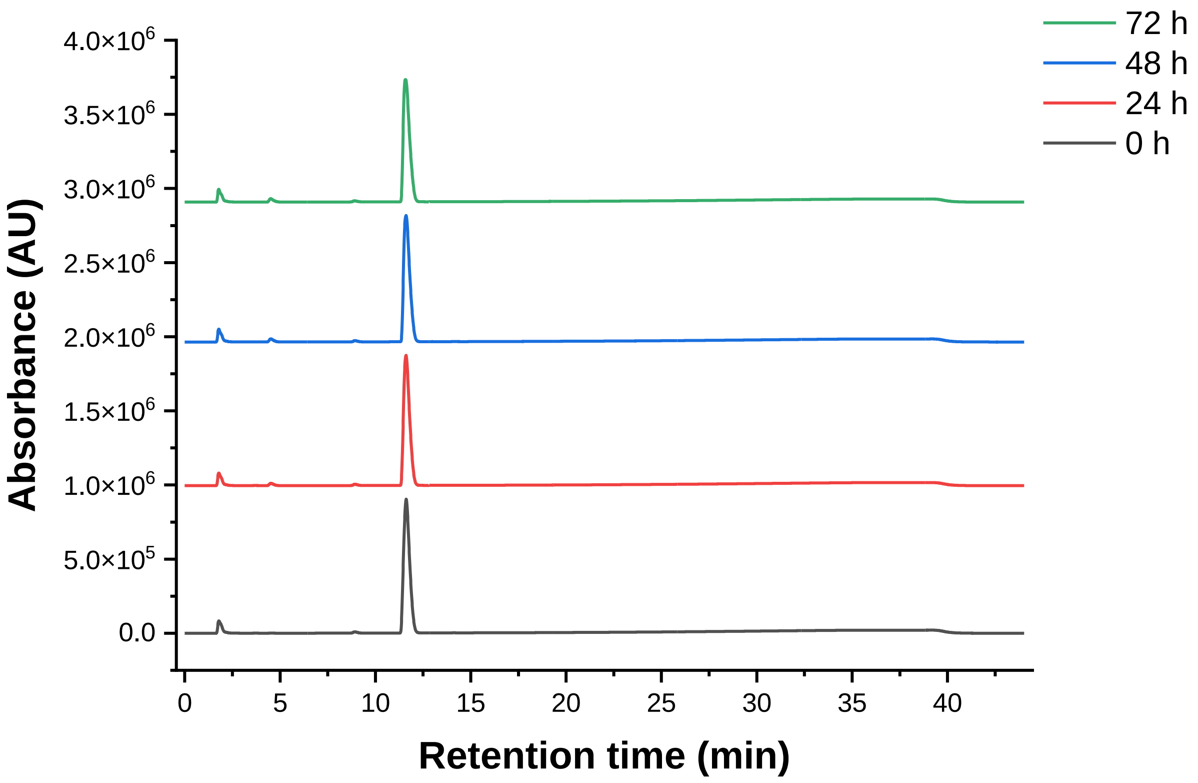


**Supplementary Figure 136**. Stability of **4F** in water/DMSO 1:1 using RP-HPLC.

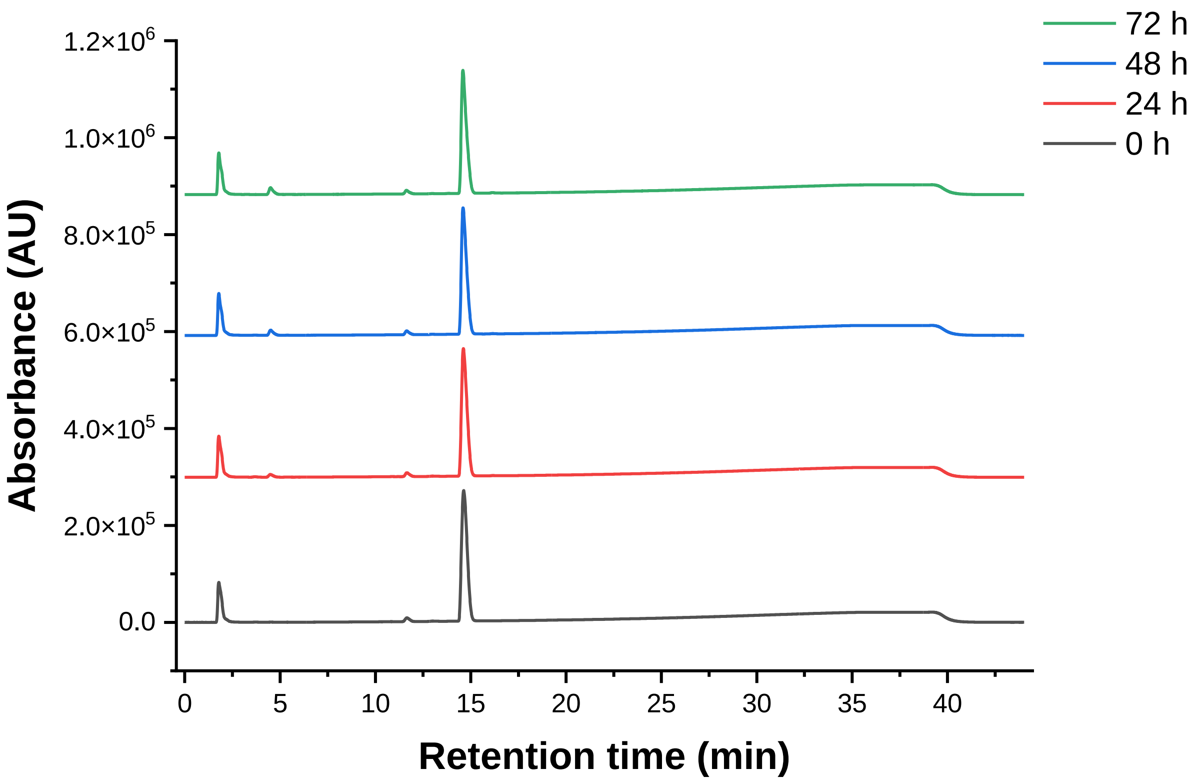


**Supplementary Figure 137**. Stability of **4G** in water/DMSO 1:1 using RP-HPLC.

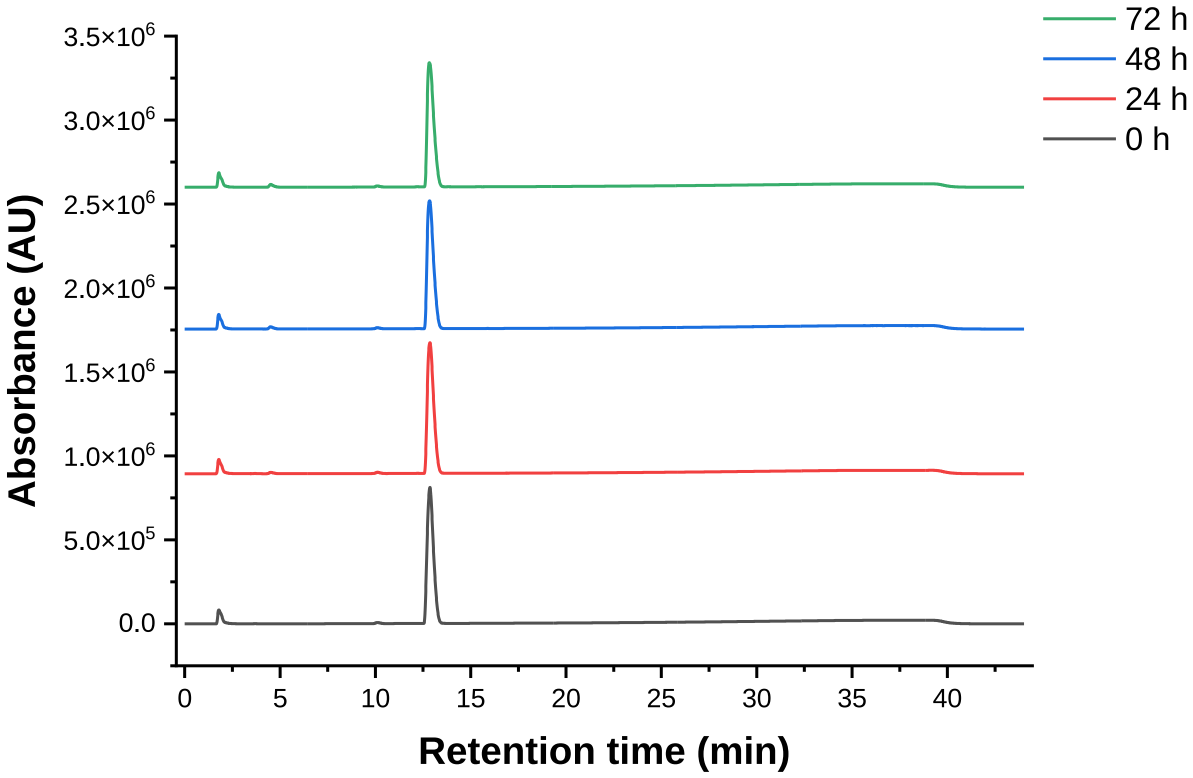


**Supplementary Figure 138**. Stability of **4H** in water/DMSO 1:1 using RP-HPLC.

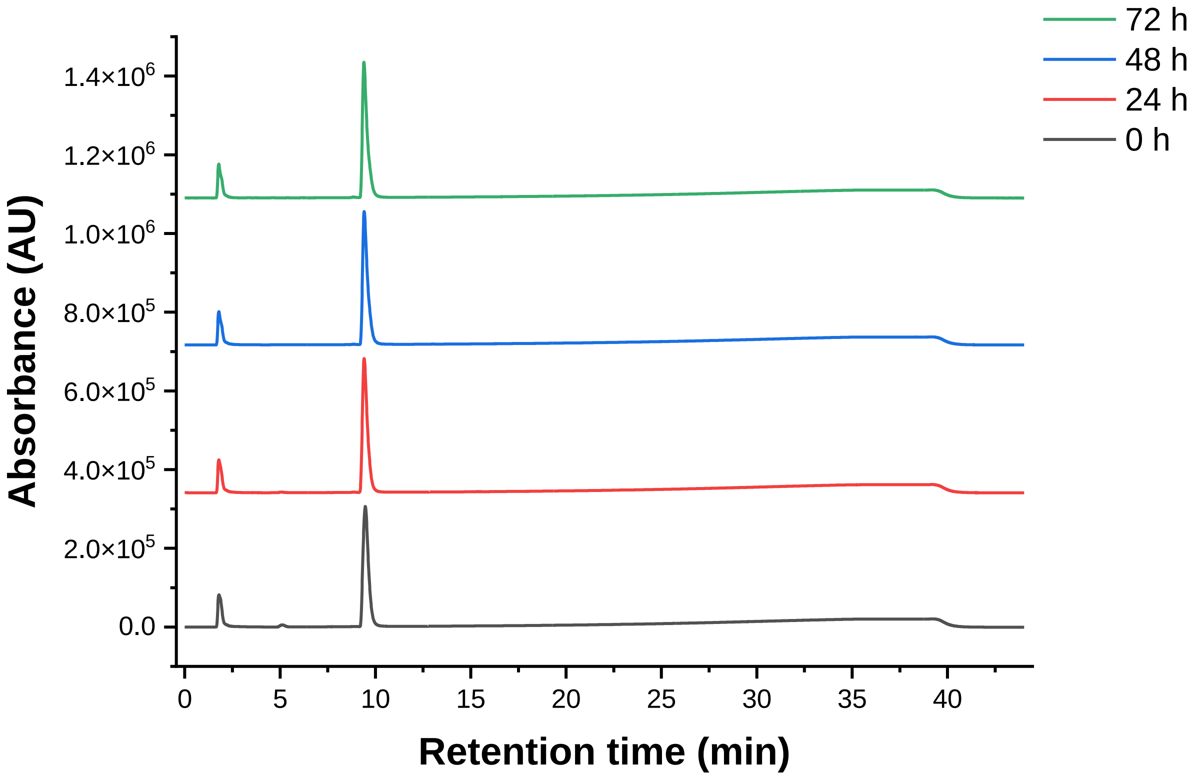


**Supplementary Figure 139**. Stability of **5A** in water/DMSO 1:1 using RP-HPLC.

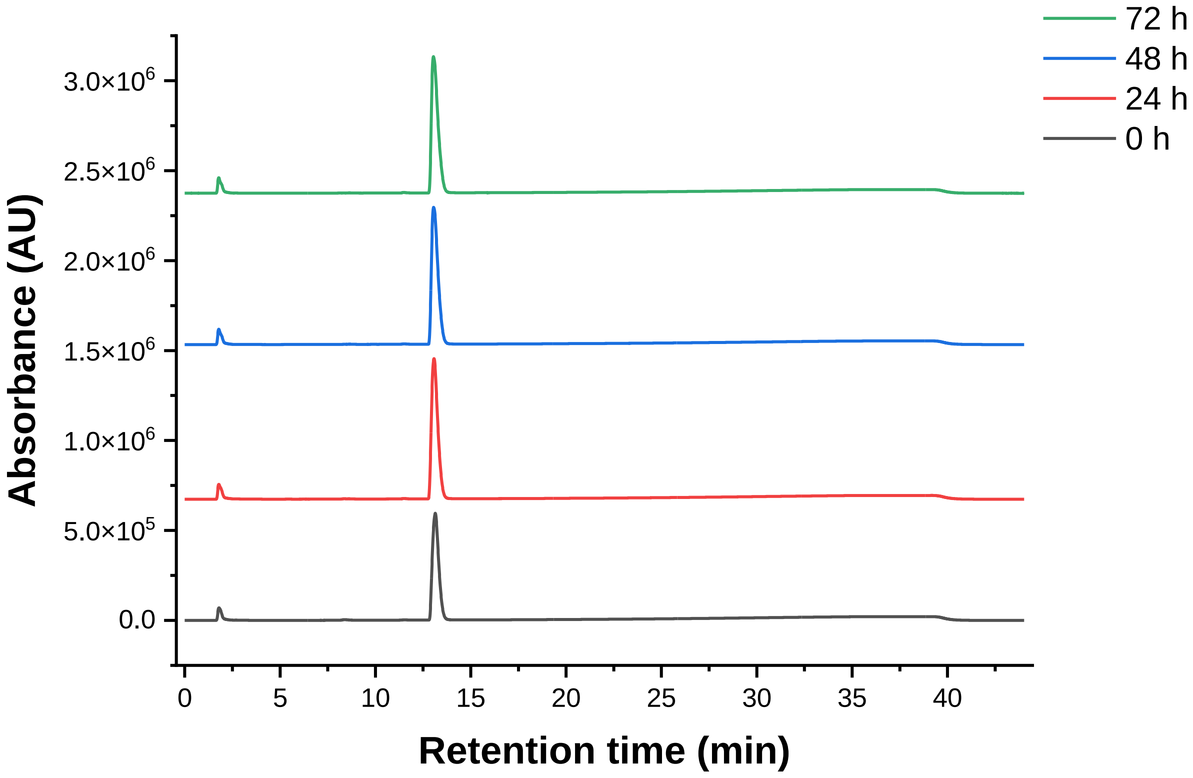


**Supplementary Figure 140**. Stability of **5B** in water/DMSO 1:1 using RP-HPLC.

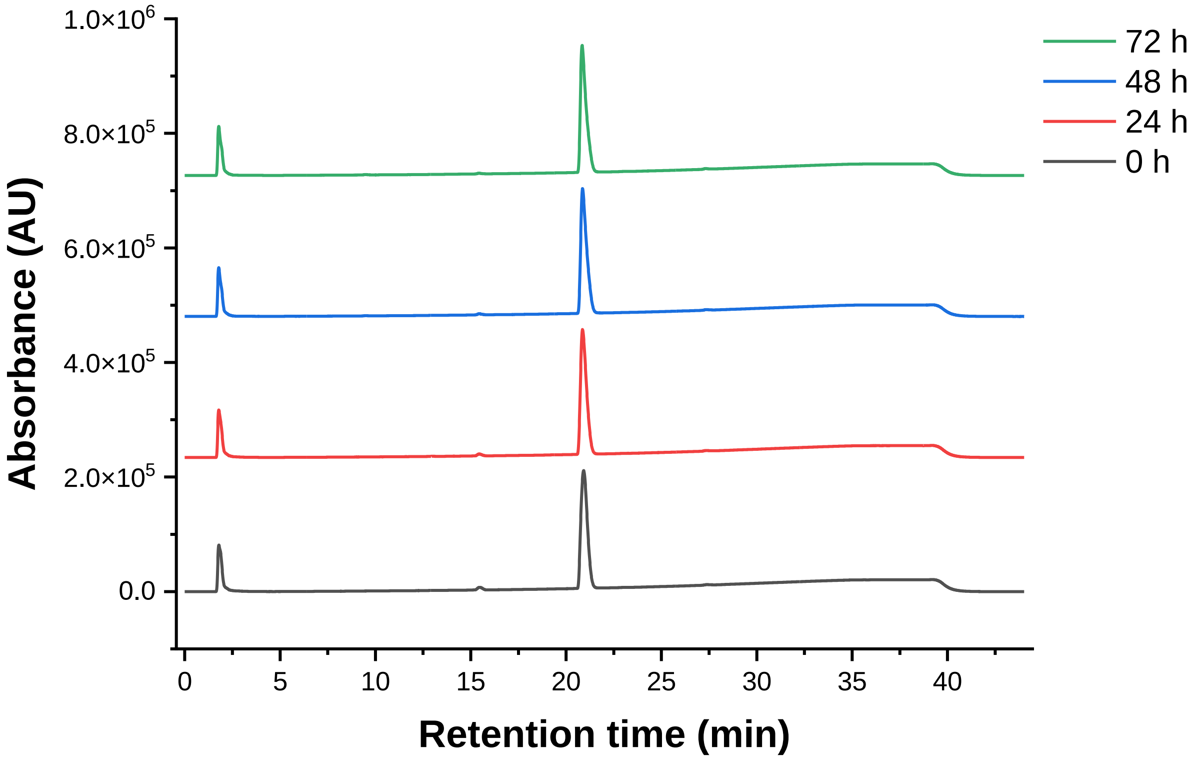


**Supplementary Figure 141.** Stability of **5C** in water/DMSO 1:1 using RP-HPLC.

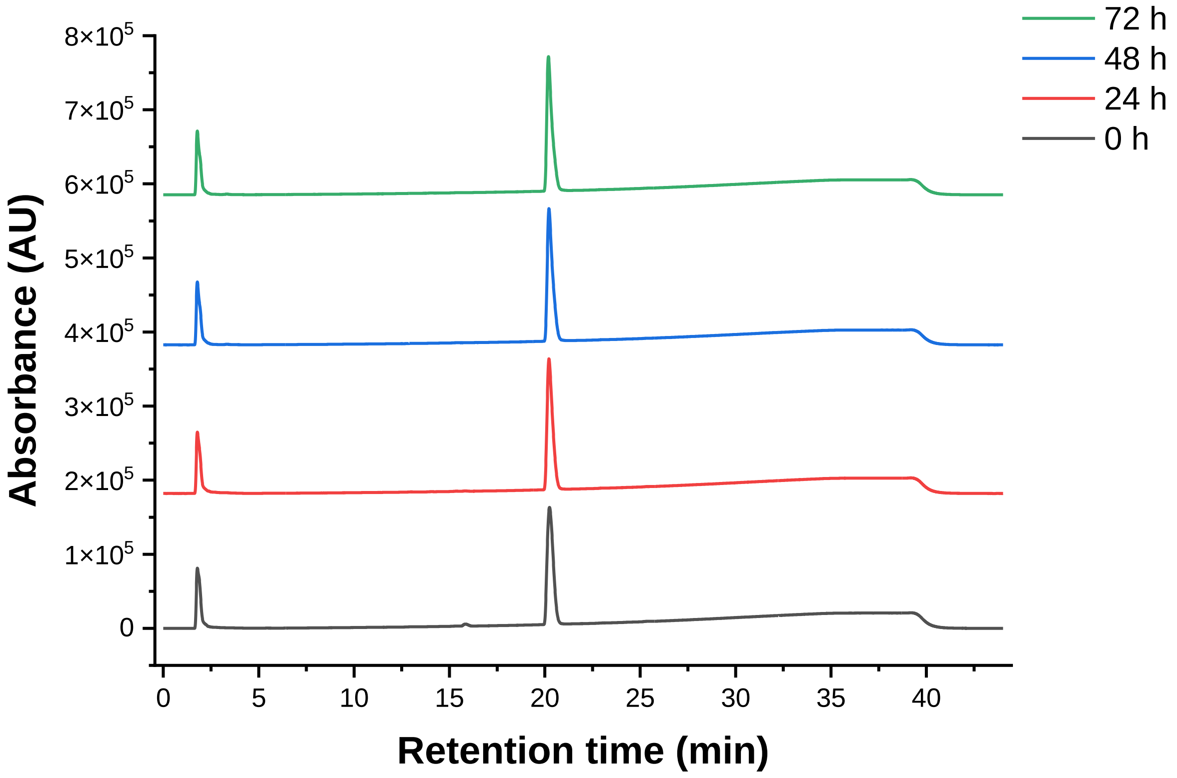


**Supplementary Figure 142**. Stability of **5D** in water/DMSO 1:1 using RP-HPLC.

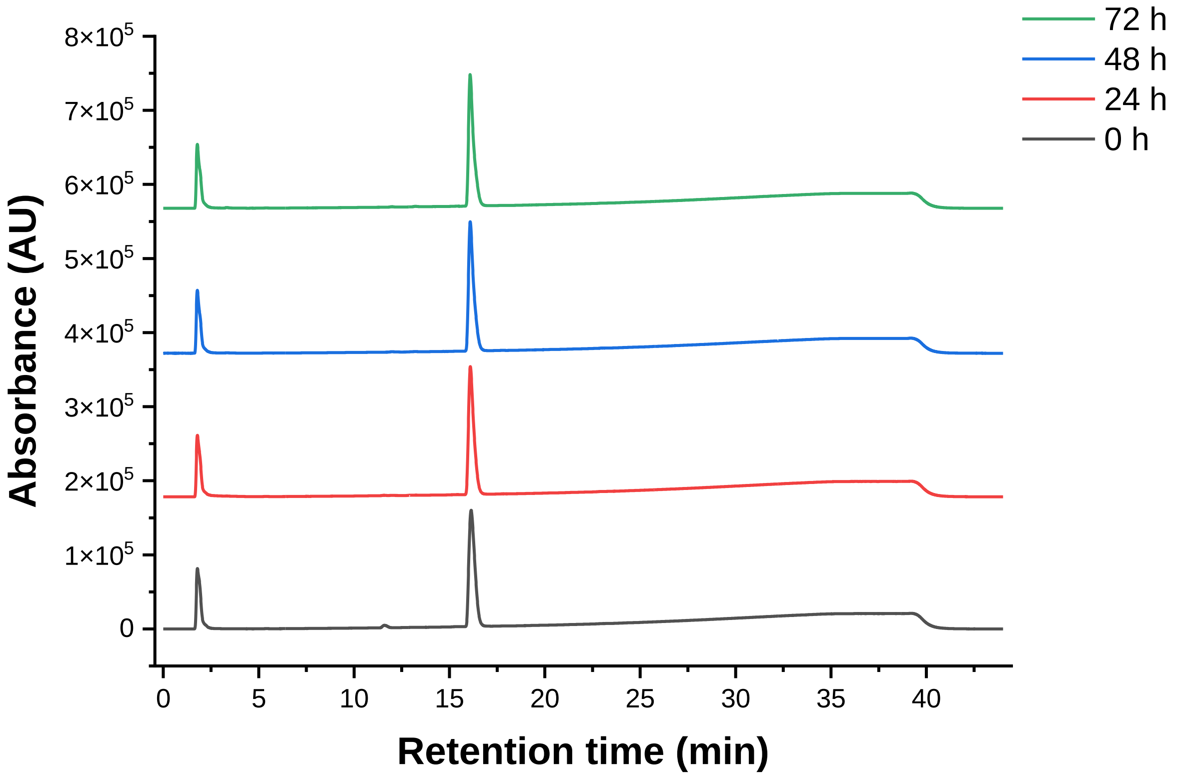


**Supplementary Figure 143**. Stability of **5E** in water/DMSO 1:1 using RP-HPLC.

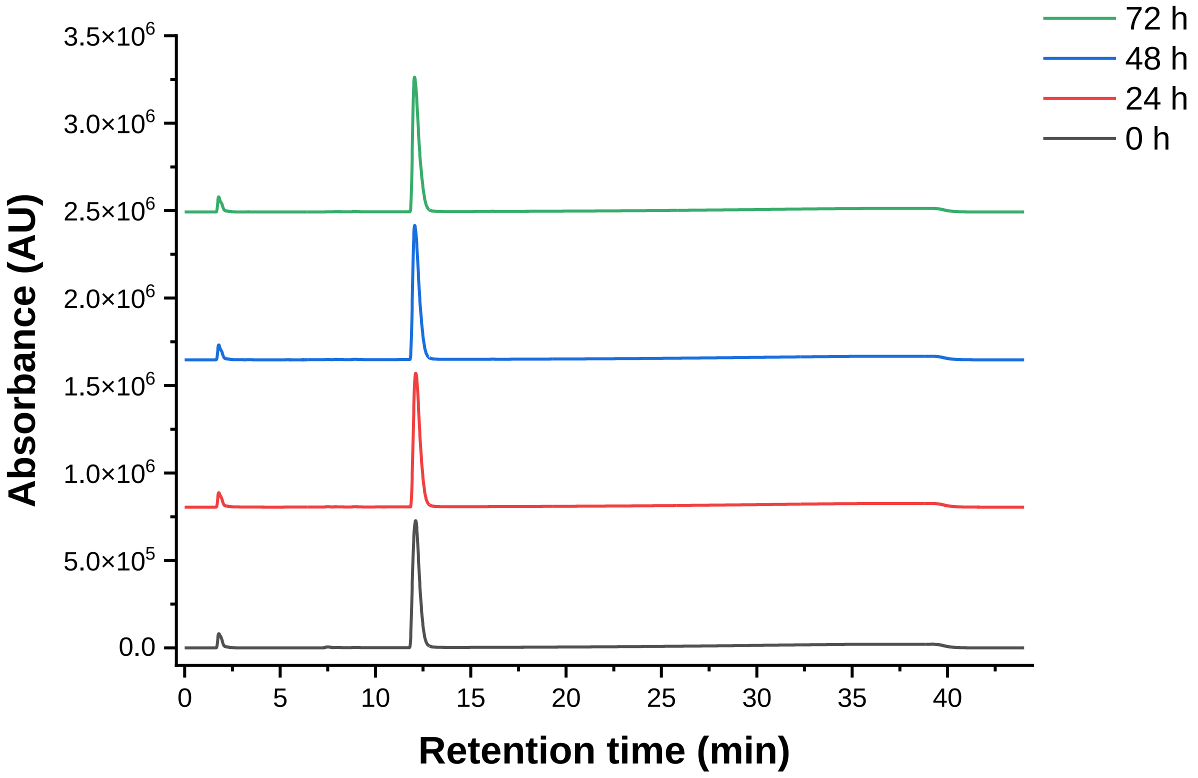


**Supplementary Figure 144**. Stability of **5F** in water/DMSO 1:1 using RP-HPLC.

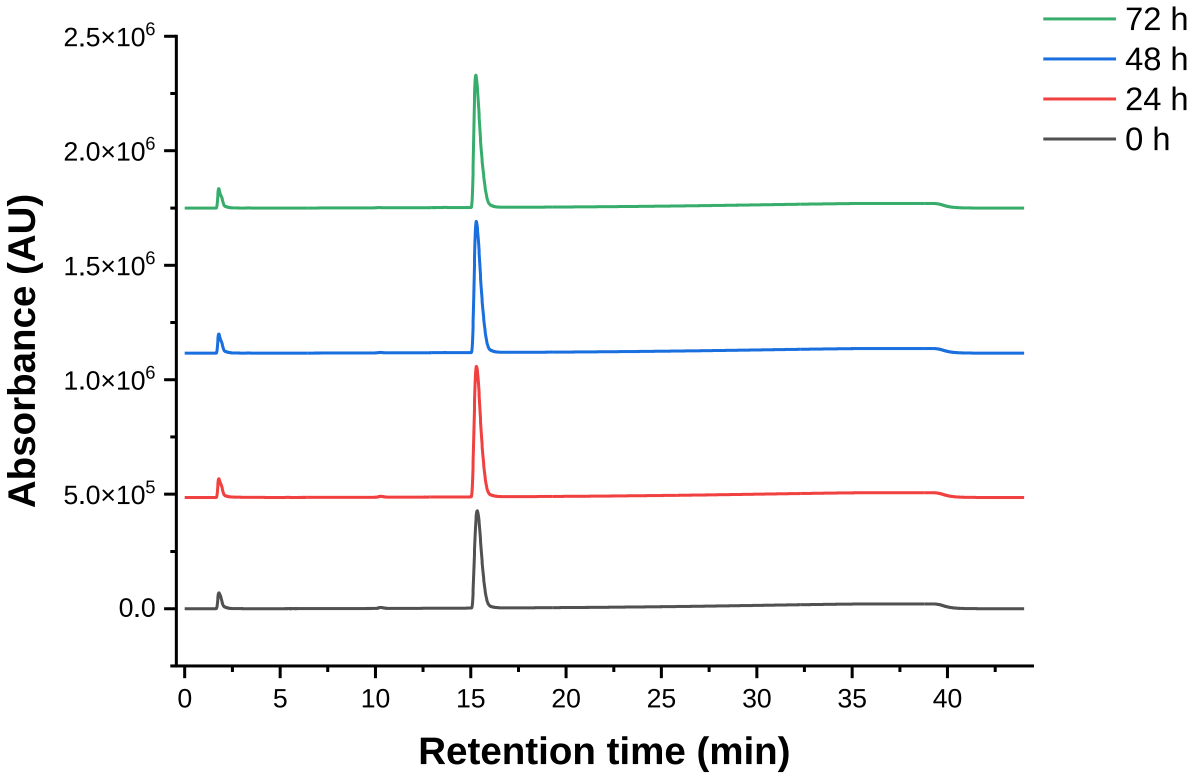


**Supplementary Figure 145**. Stability of **5G** in water/DMSO 1:1 using RP-HPLC.

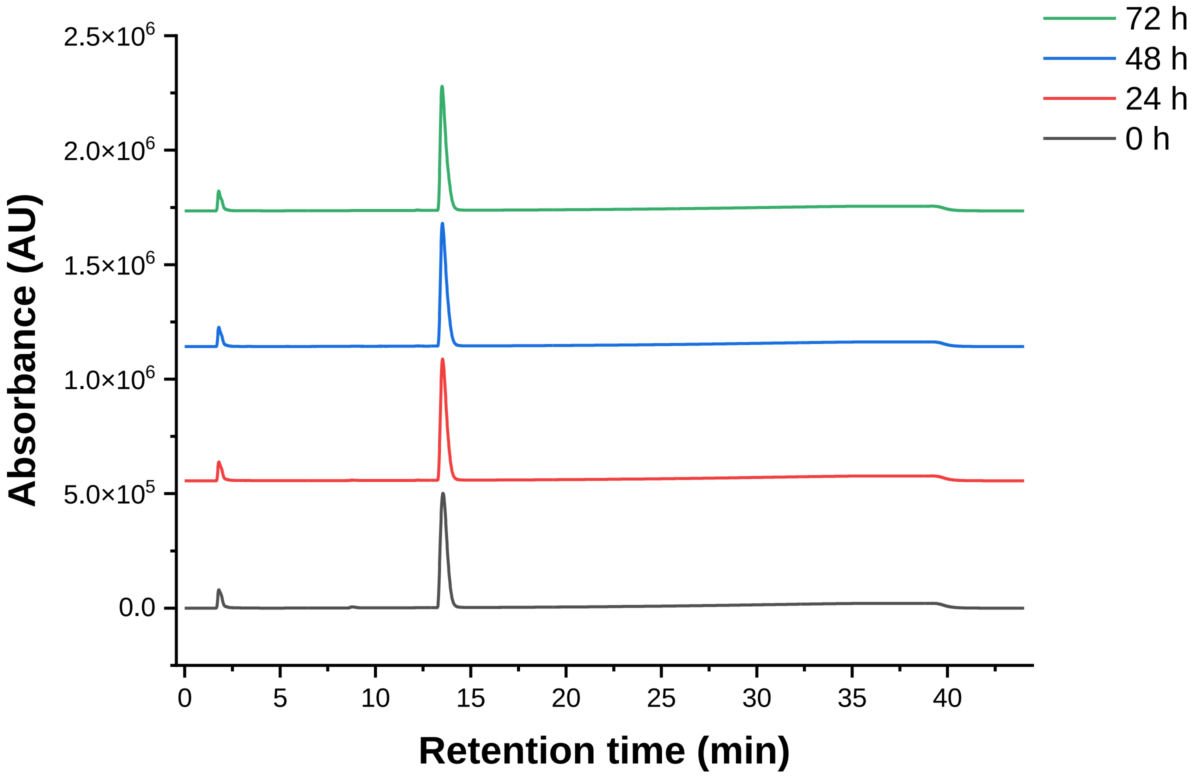


**Supplementary Figure 146**. Stability of **5H** in water/DMSO 1:1 using RP-HPLC.

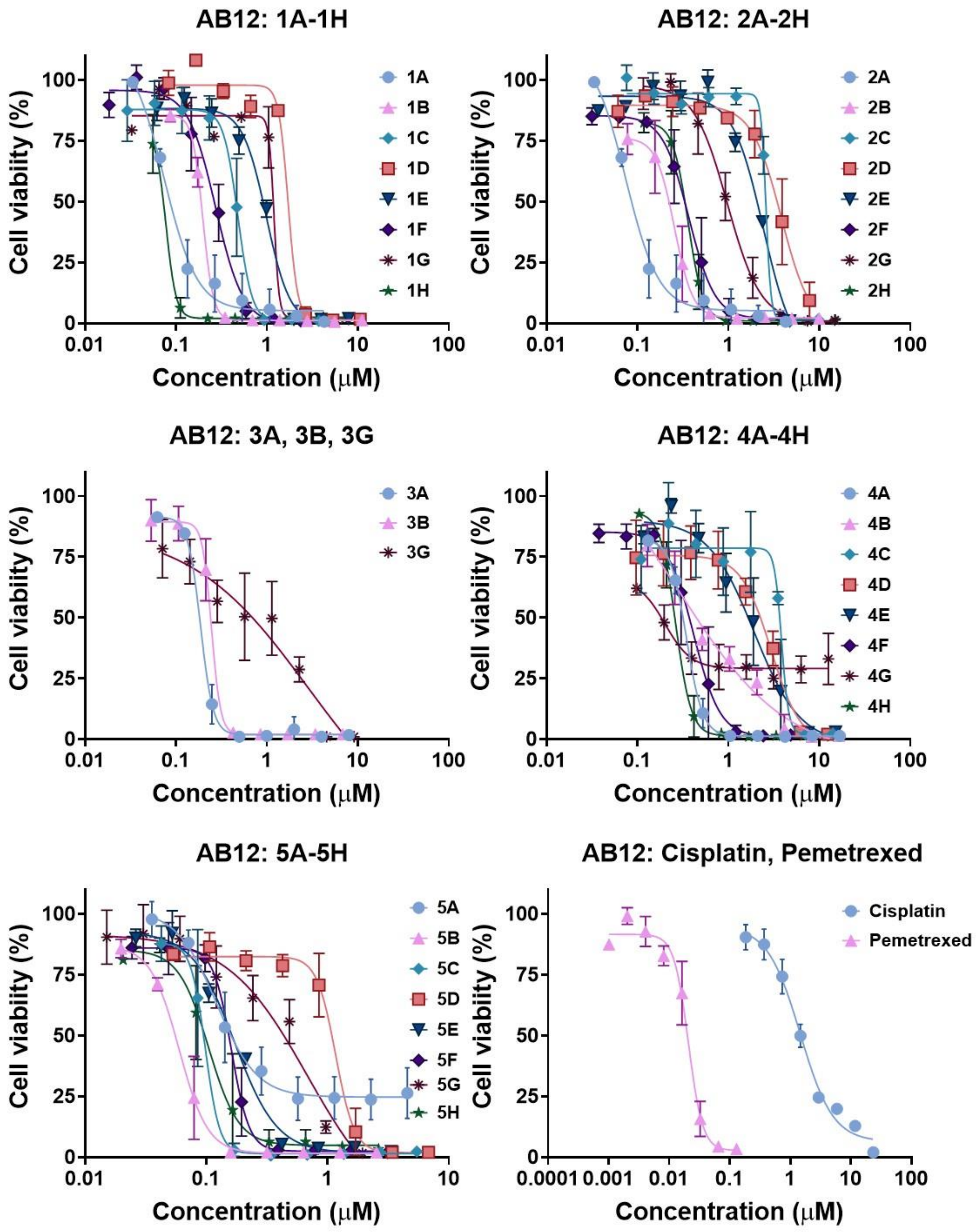


**Supplementary Figure 147.** Concentration-effect curves of 35 Au(III)-DTC complexes in a murine AB12 MPM cell line in comparison with cisplatin and pemetrexed. Values were obtained by the MTT assay and are means ± standard deviations from at least four independent experiments using exposure times of 72 h.

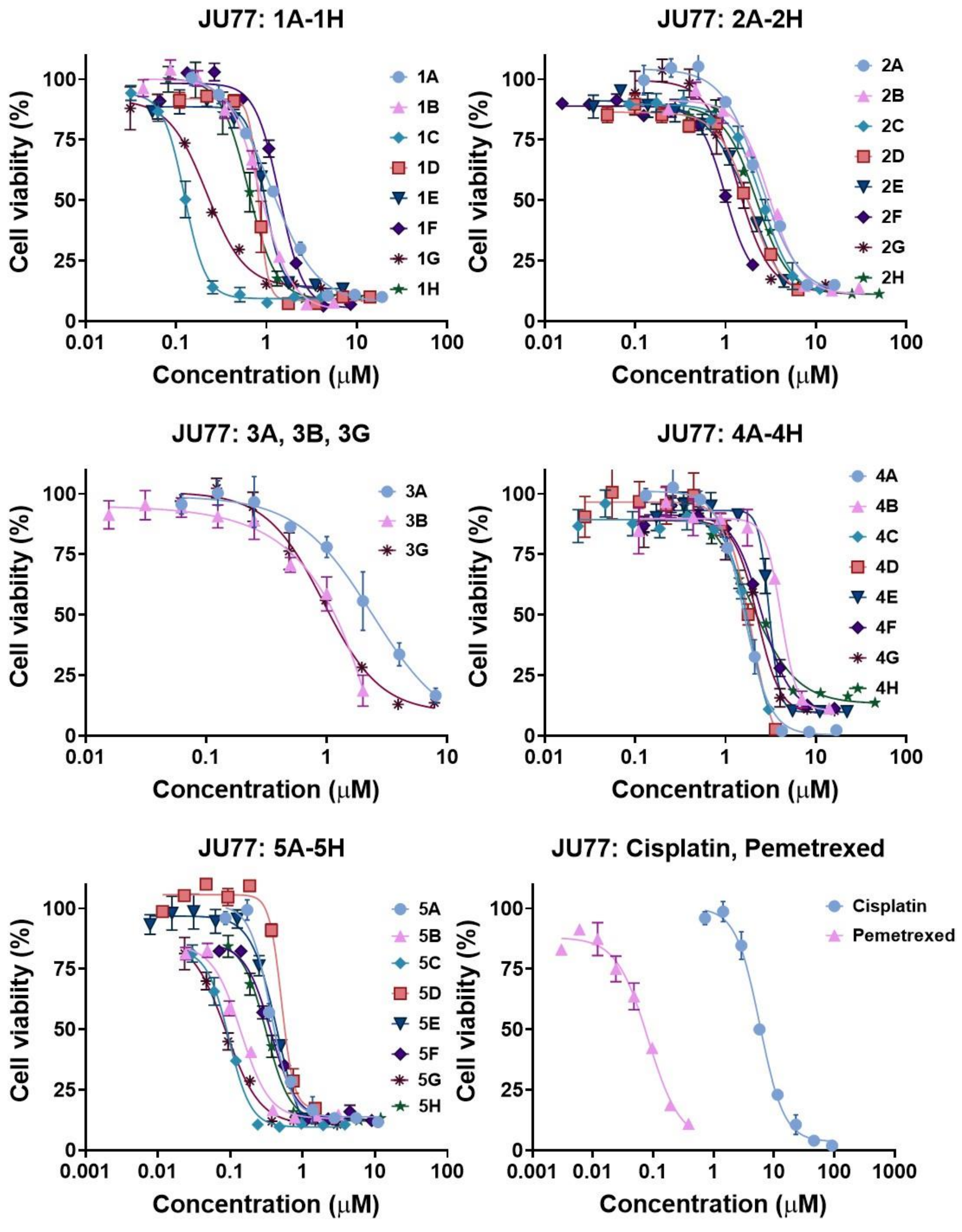


**Supplementary Figure 148.** Concentration-effect curves of 35 Au(III)-DTC complexes in a human JU77 MPM cell line in comparison with cisplatin and pemetrexed. Values were obtained by the MTT assay and are means ± standard deviations from at least four independent experiments using exposure times of 72 h.

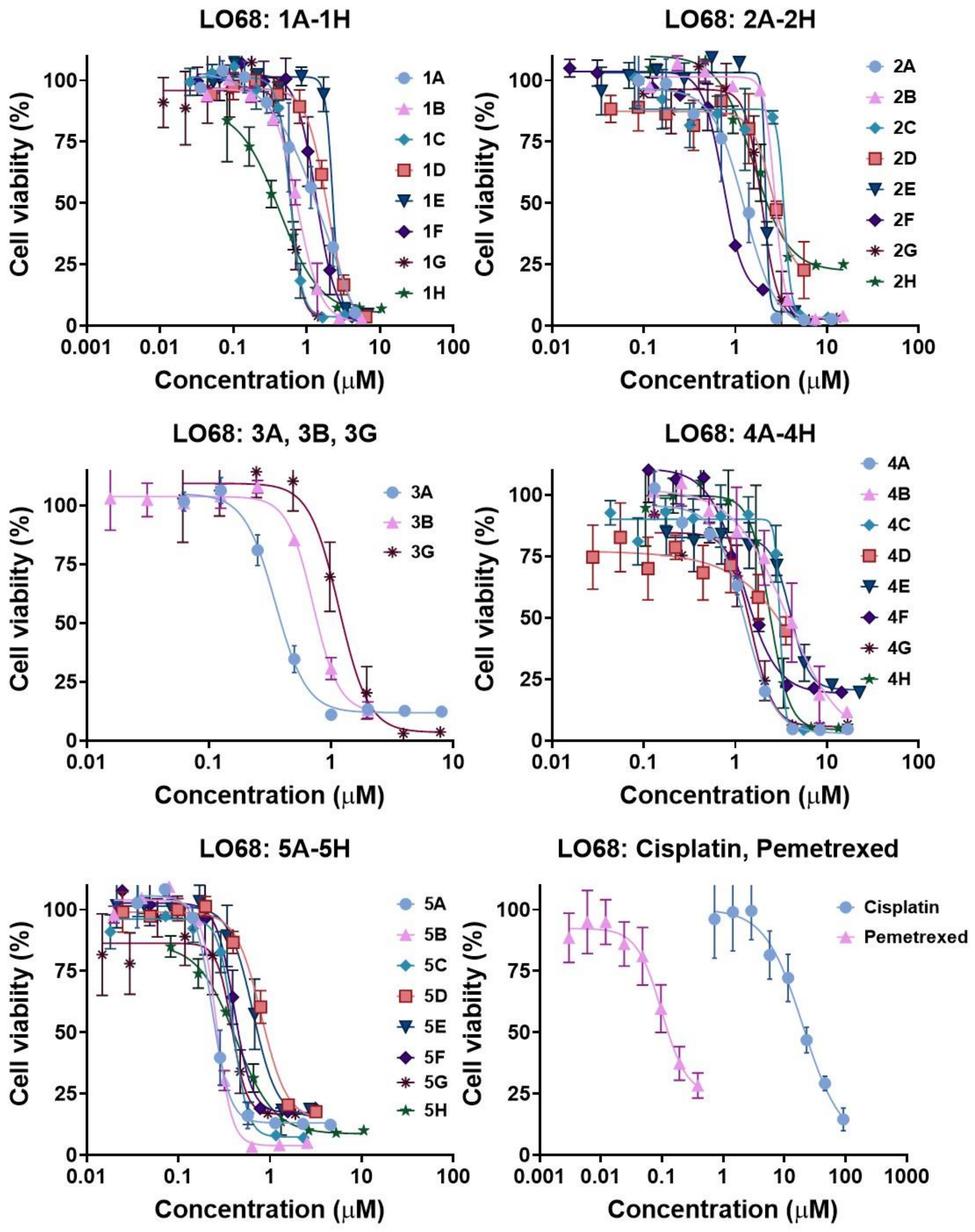


**Supplementary Figure 149.** Concentration-effect curves of 35 Au(III)-DTC complexes in a human LO68 MPM cell line in comparison with cisplatin and pemetrexed. Values were obtained by the MTT assay and are means ± standard deviations from at least four independent experiments using exposure times of 72 h.

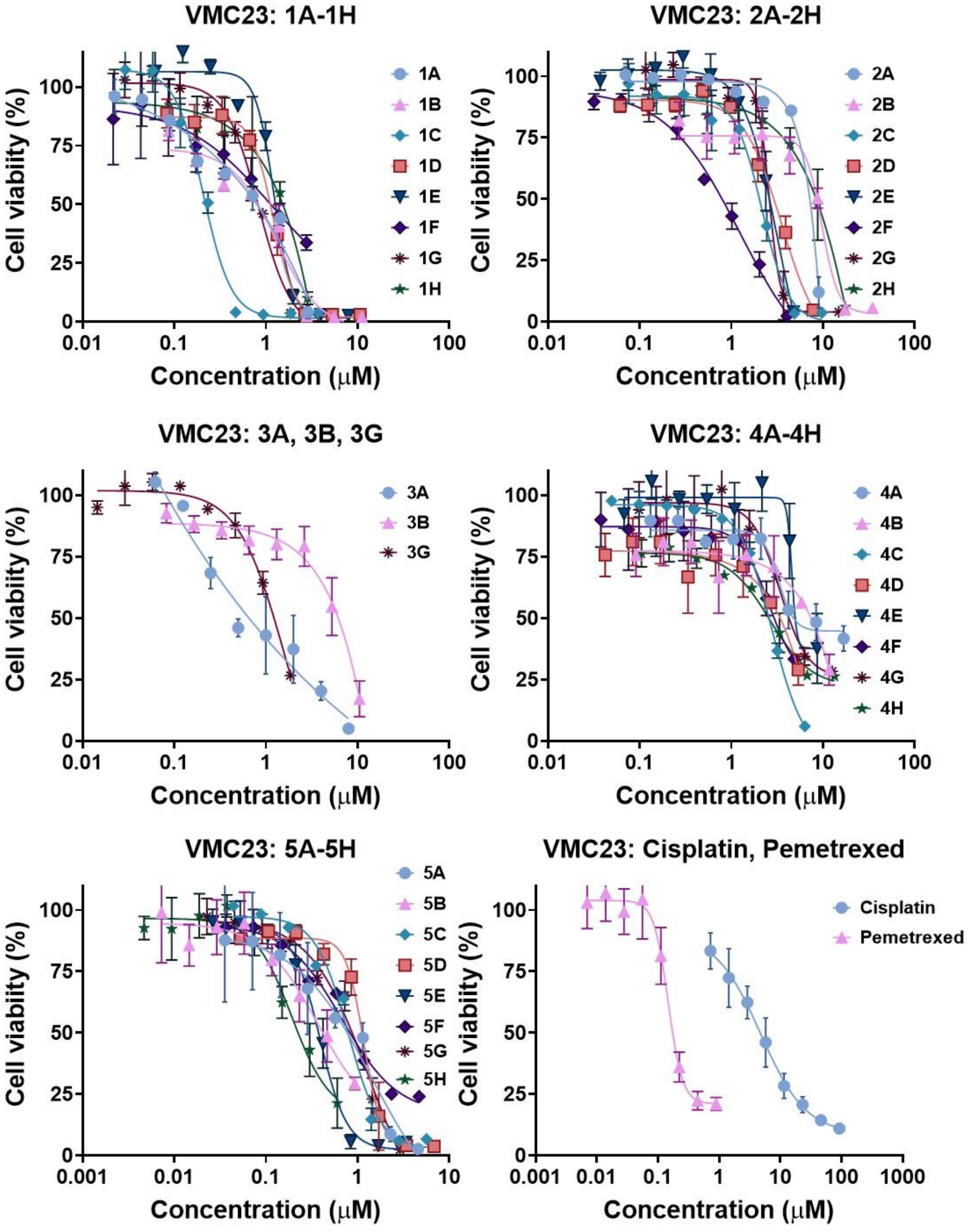


**Supplementary Figure 150.** Concentration-effect curves of 35 Au(III)-DTC complexes in a patient-derived VMC23 cell line in comparison with cisplatin and pemetrexed. Values were obtained by the MTT assay and are means ± standard deviations from at least four independent experiments using exposure times of 72 h.

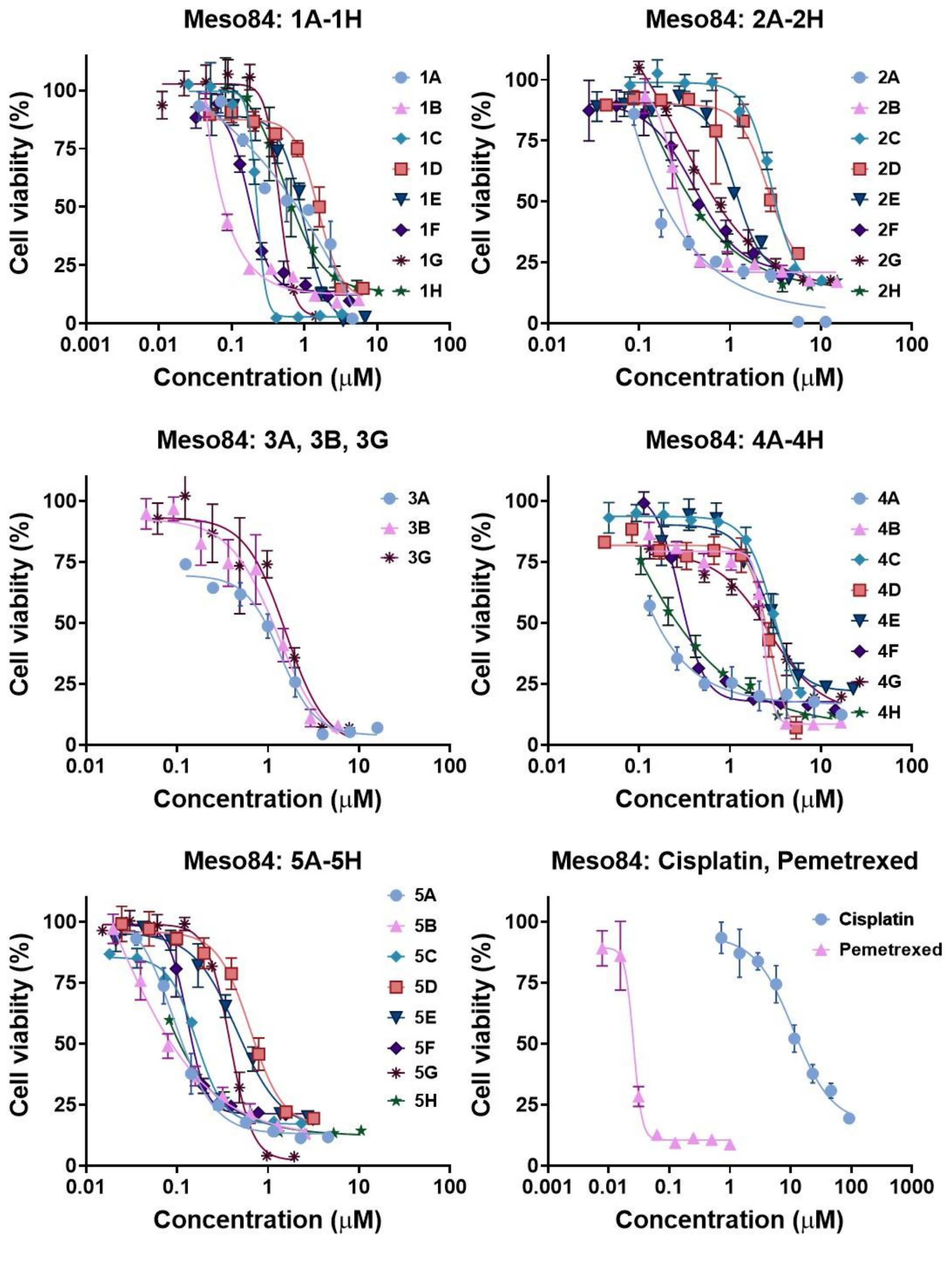


**Supplementary Figure 151.** Concentration-effect curves of 35 Au(III)-DTC complexes in a patient-derived Meso84 cell line in comparison with cisplatin and pemetrexed. Values were obtained by the MTT assay and are means ± standard deviations from at least four independent experiments using exposure times of 72 h.

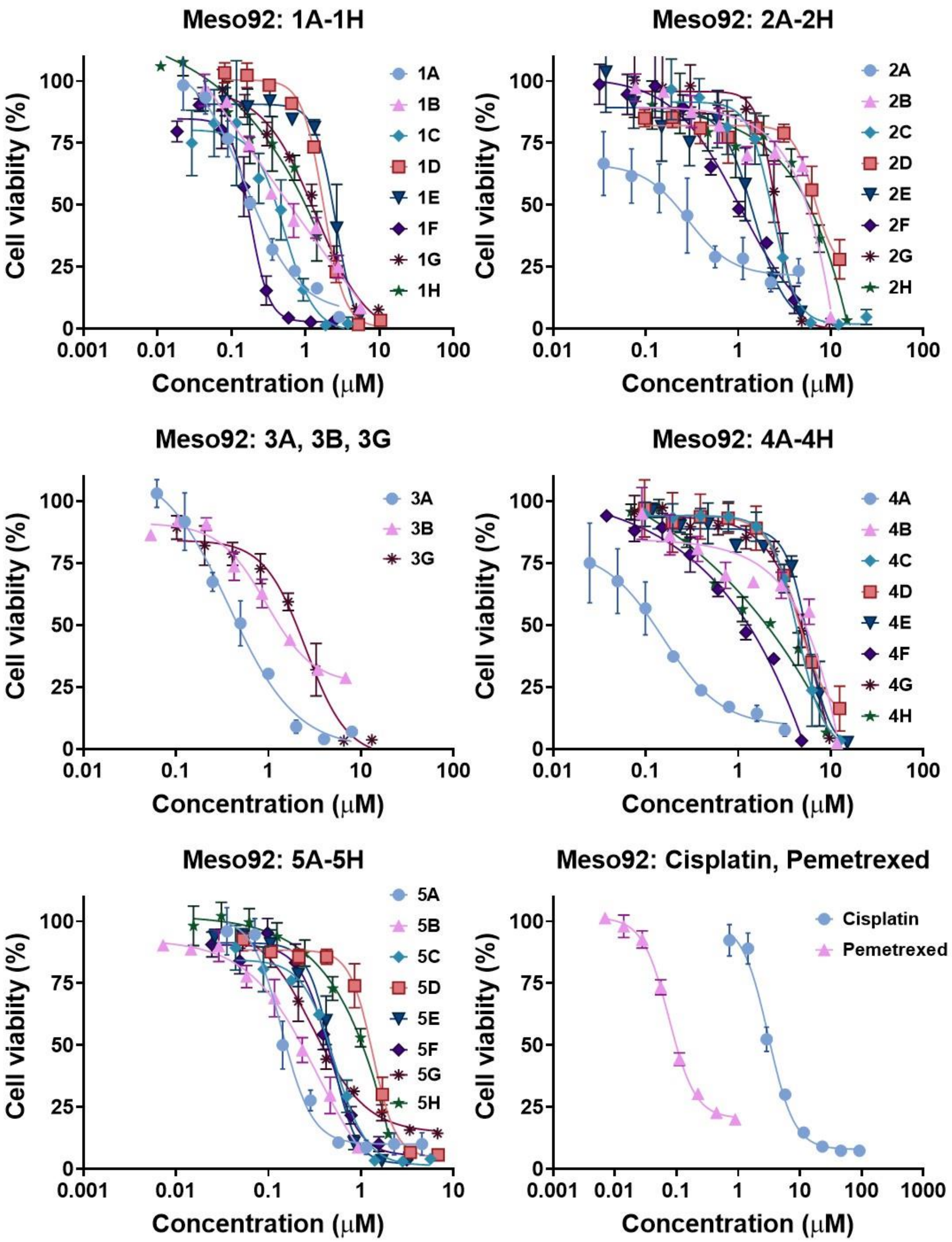


**Supplementary Figure 152.** Concentration-effect curves of 35 Au(III)-DTC complexes in a patient-derived Meso92 cell line in comparison with cisplatin and pemetrexed. Values were obtained by the MTT assay and are means ± standard deviations from at least four independent experiments using exposure times of 72 h.

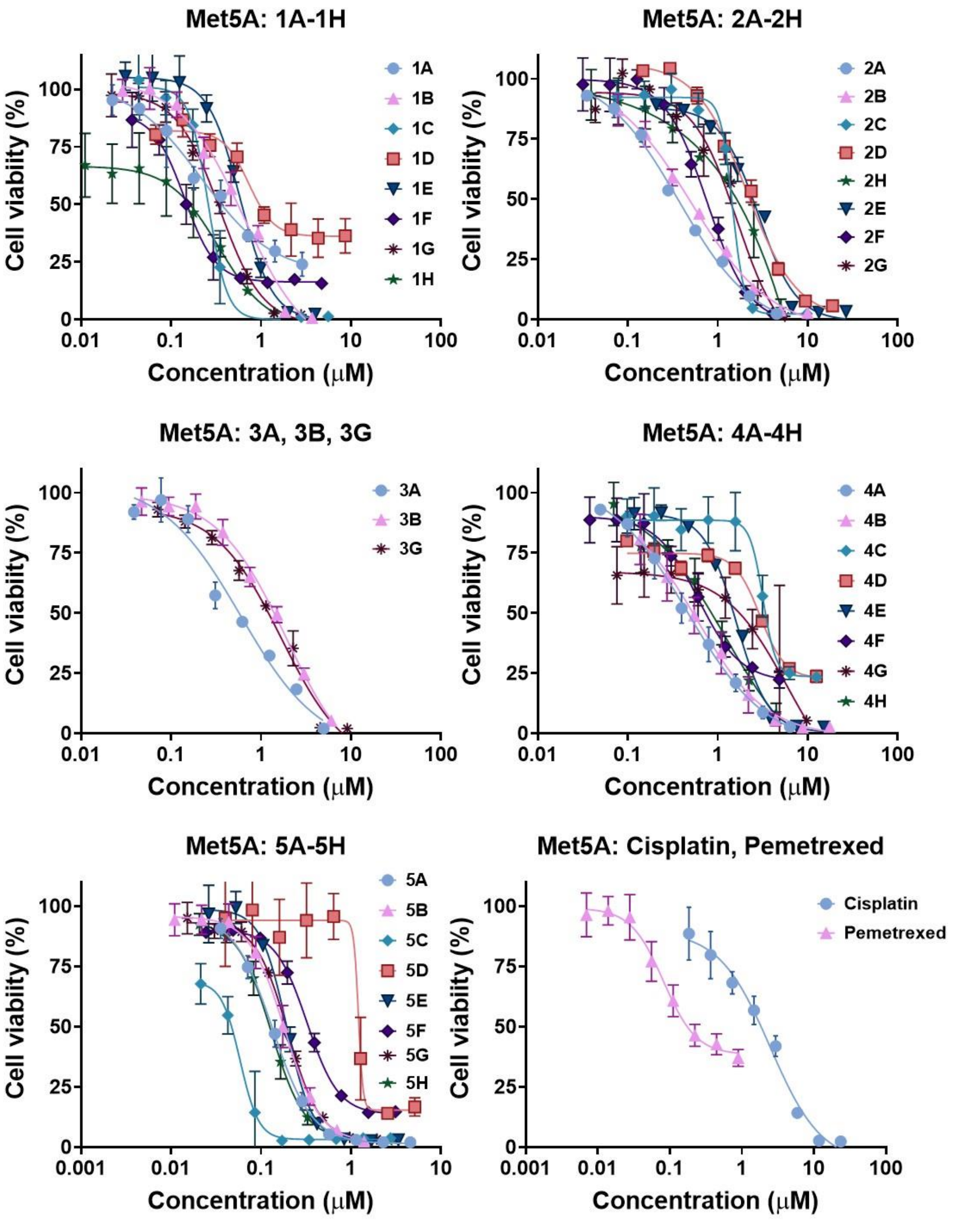


**Supplementary Figure 153.** Concentration-effect curves of 35 Au(III)-DTC complexes in a human Met5A non-malignant mesothelioma cell line in comparison with cisplatin and pemetrexed. Values were obtained by the MTT assay and are means ± standard deviations from at least four independent experiments using exposure times of 72 h.

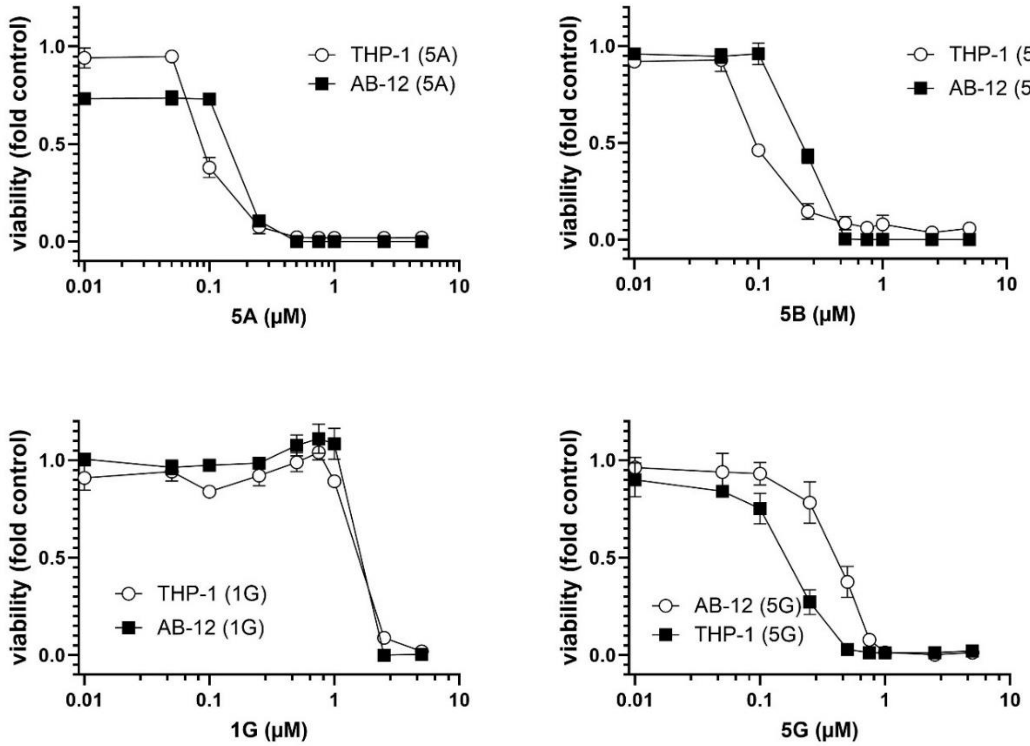


**Supplementary Figure 154.** Concentration-effect curves of complexes **1G**, **5A**, **5B** and **5G** toward M0-differentiated THP-1 cells and murine AB12 MPM cells

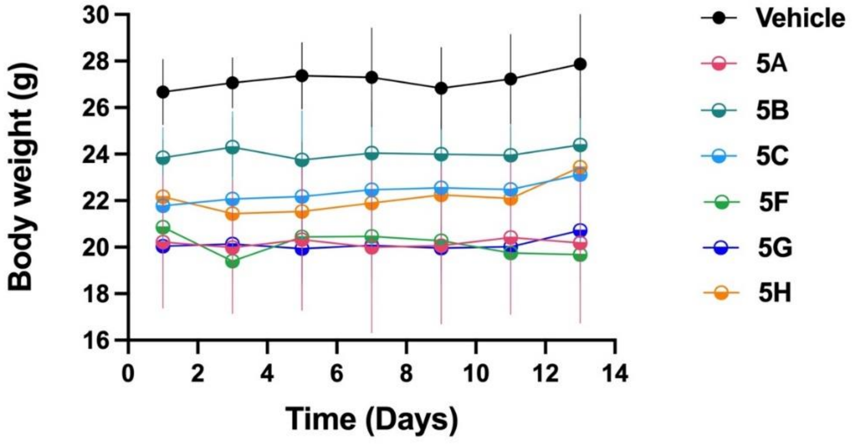


**Supplementary Figure 155.** Body weight changes of Balb/C mice during a toxicity study. Mice were treated with **5A** (4 mg/kg), **5B** (4 mg/kg), **5C** (4 mg/kg), **5F** (2.5 mg/kg), **5G** (8 mg/kg), **5H** (4 mg/kg) in 4% DMSO/Cremophore EL in saline and respective vehicle. Compounds **5A**, **5H**, and **5F** were injected daily (total of 12 injections), compounds **5B**, **5C** and **5G** were injected every other day (total of 6 injections) starting from Day 1 via i.p. route.

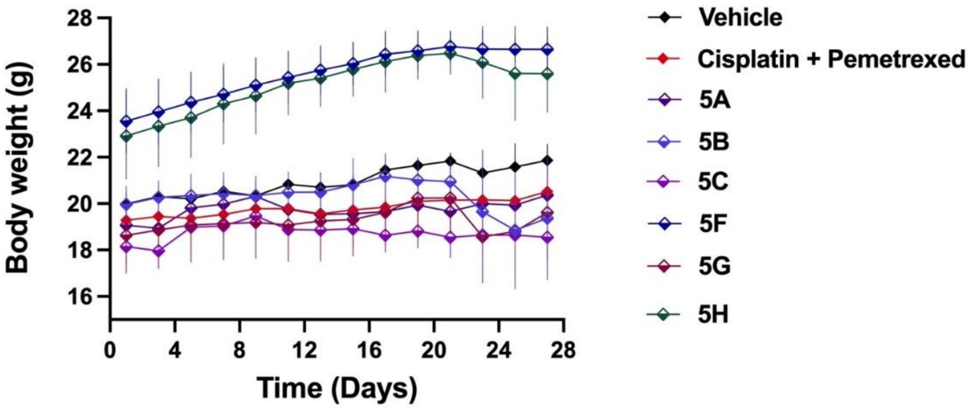


**Supplementary Figure 156.** Body weight changes of Balb/C mice (n=6) with subcutaneous AB12 MPM tumor allografts treated with **5A** (4 mg/kg), **5B** (4 mg/kg), **5C** (4 mg/kg), **5F** (2.5 mg/kg), **5G** (8 mg/kg), **5H** (4 mg/kg), cisplatin (1 mg/kg) + pemetrexed (5 mg/kg), and respective vehicle (4% DMSO/Cremophore EL in saline) on Days 19, 21, and 23 via i.p. route.

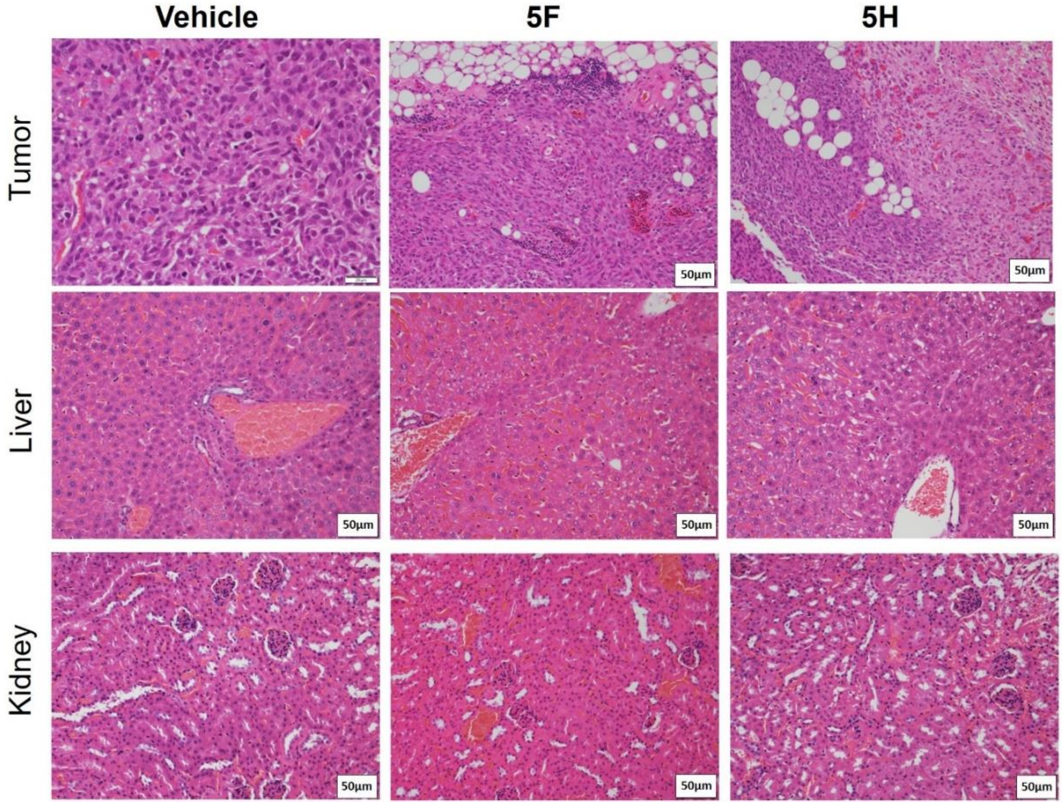


**Supplementary Figure 157.** Representative H&E-stained images of liver and kidney sections from mice treated with vehicle and complexes **5H** and **5F**. Scale bars represent 50 μm.

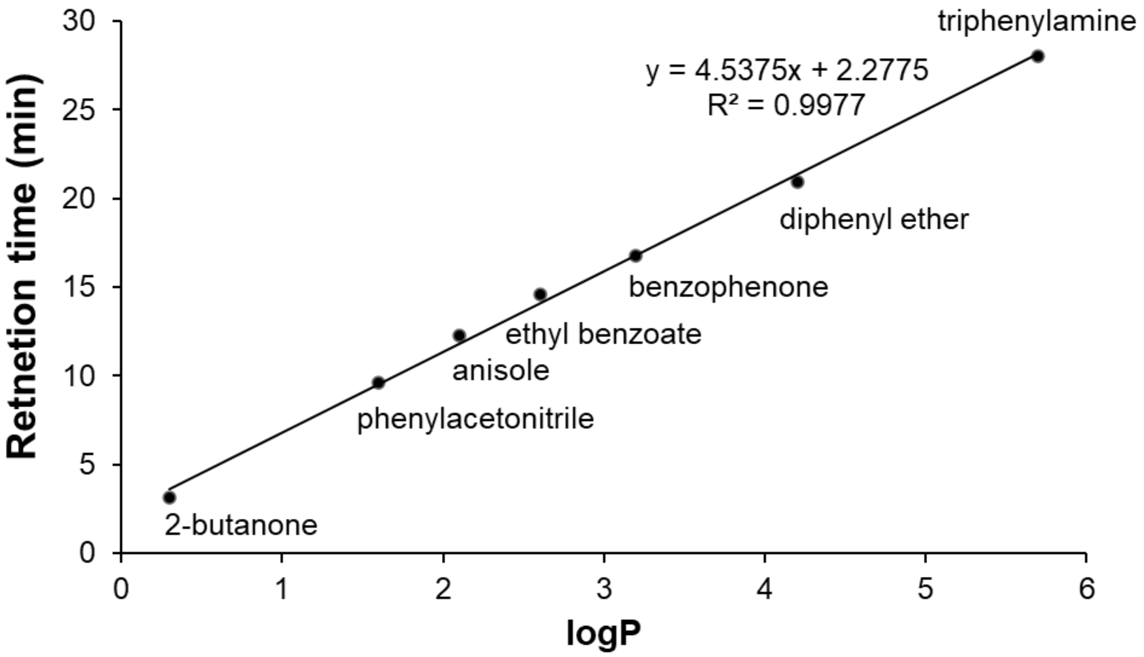


**Supplementary Figure 158**. LogP standard curve used to determine the logP of the complexes.

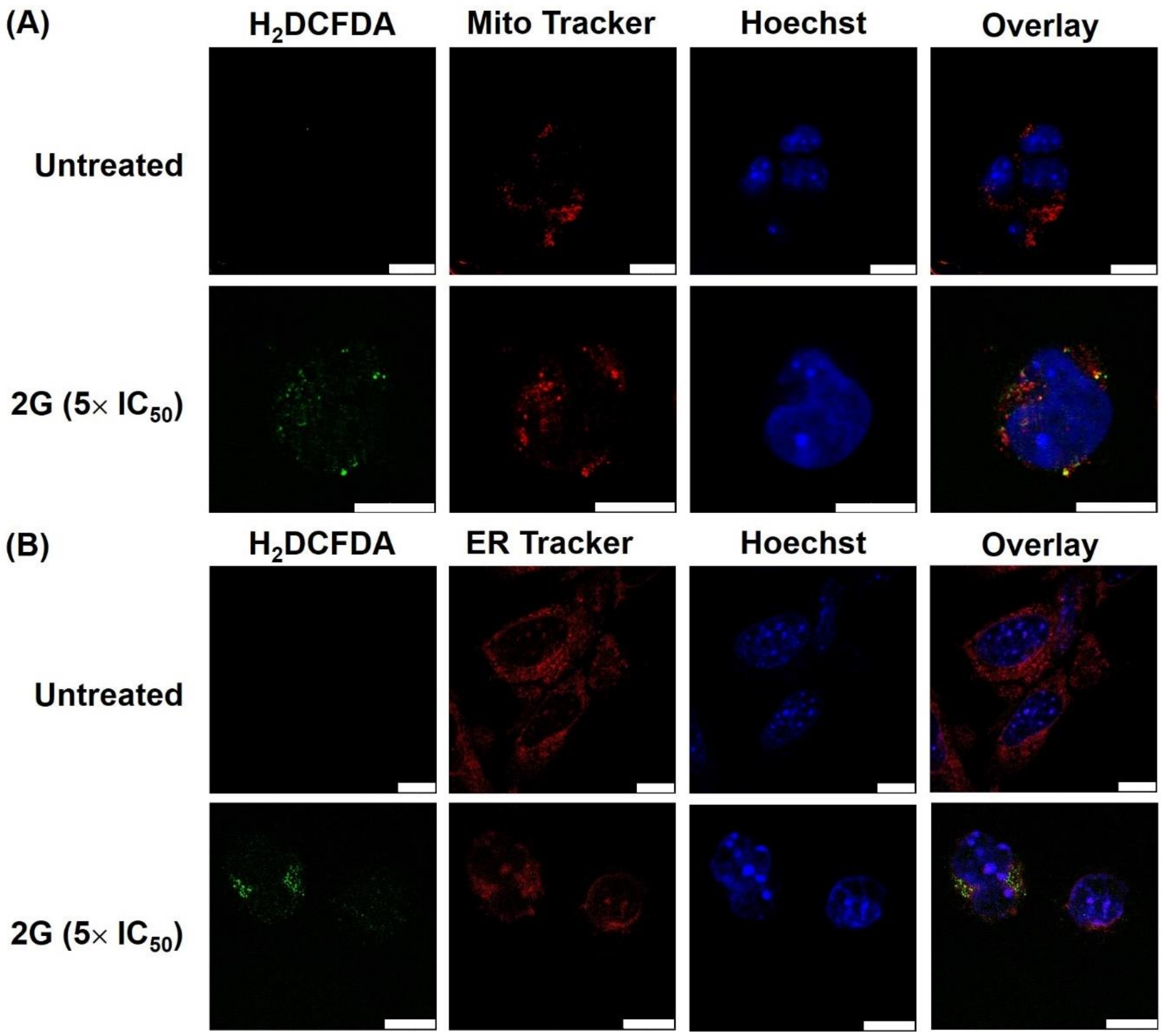


**Supplementary Figure 159.** ROS detection using live cell confocal microscopy imaging. AB12 cells were stained with $H_2$DCFDA (excitation/emission wavelengths of 504 nm/525 nm) for 15 min, incubated with complex **2G** (5× $IC_{50}$) for 4 h. Hoechst 33342 dye (excitation/emission wavelength of 350 nm/461 nm) was used for nuclear staining. (**A**) Mitotracker TM Red Deep Red dye (excitation/emission wavelengths of 644 nm/665 nm) or (**B**) ER Tracker™ Blue-White DPX (excitation/emission wavelengths of 374 nm/430-640 nm) were used for mitochondrial and ER staining, respectively. Scale bars represent 10 μm.

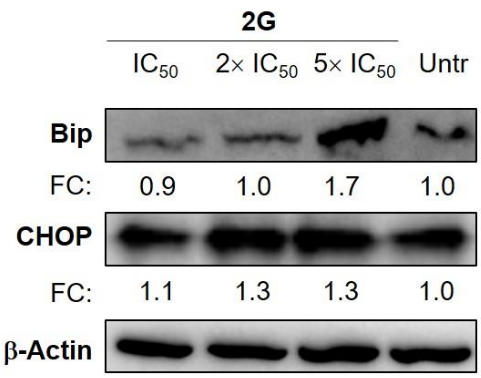


**Supplementary Figure 160.** Western blot analysis of Binding Immunoglobulin Protein (BiP) and C/EBP Homologous Protein (CHOP) involved in ER stress in AB12 cells treated with **2G** for 4 h at indicated concentrations. The fold change (FC) denotes the intensity change compared to untreated cells and is normalized to β-actin levels.

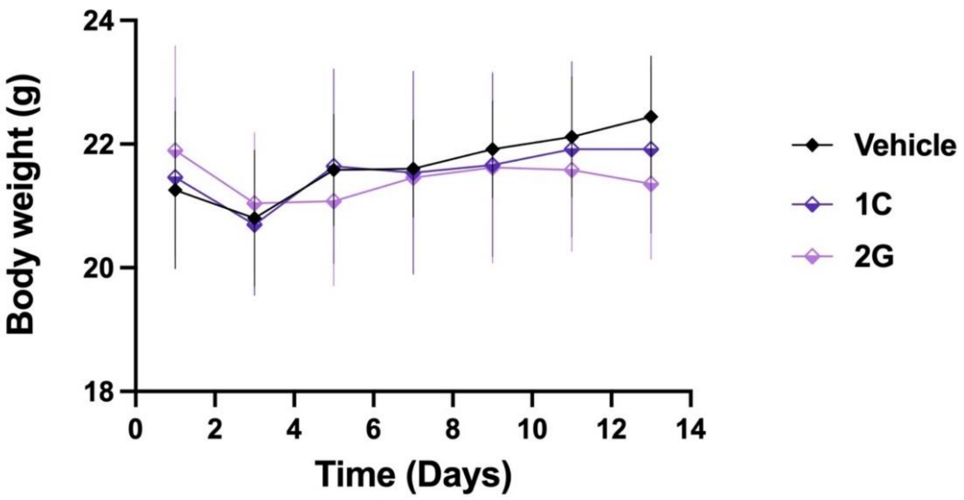


**Supplementary Figure 161.** Body weight changes of Balb/C mice in a toxicity study, with injections starting on Day 1. The maximum soluble doses were administered in 4% DMSO/4% Cremophore EL in PBS: **1C** (1.5 mg/kg), **2G** (3.5 mg/kg), and vehicle (4% DMSO/4% Cremophore EL in saline). Compounds were given every other day (total of 5 injections) starting from Day 1.

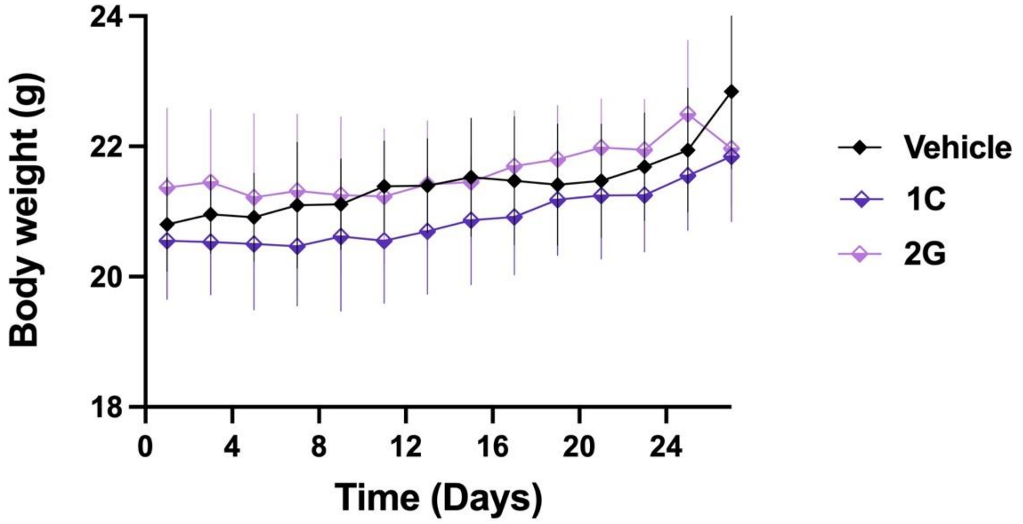


**Supplementary Figure 162.** Body weight changes of Balb/C mice (n=6) with subcutaneous AB12 MPM tumor allografts treated with **1C** (1.5 mg/kg), **2G** (3.5 mg/kg) and respective vehicle (4% DMSO/Cremophore EL in PBS) on Days 19, 21, and 23 via i.p. route.

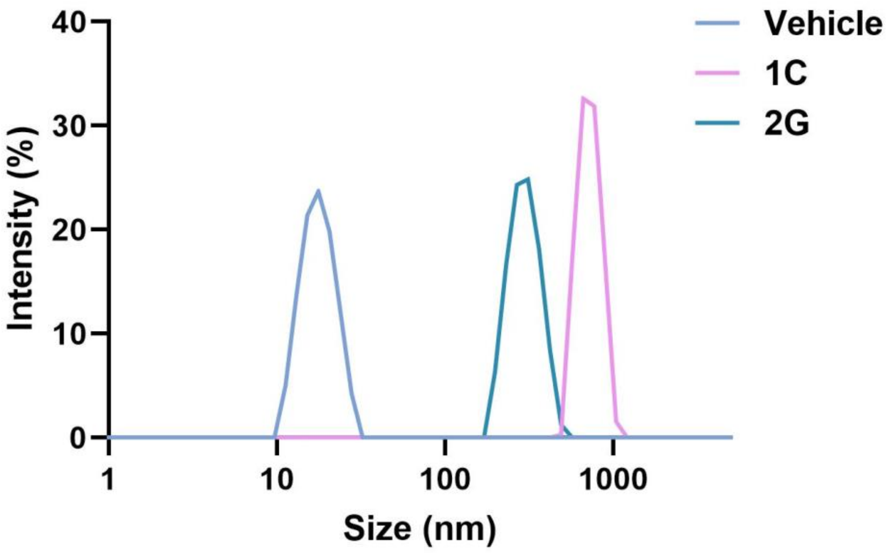


**Supplementary Figure 163**. Dynamic light scattering (DLS) spectra of the vehicle, **1C** (0.16 mg/ml) and **2G** (0.39 mg/ml) in PBS with 4-5% DMSO/Cremophor EL showing the particle size distribution of Au complexes administered to the mice for the *in vivo* experiment depicted in Figure 5C. Median size values for each peak are as follows: vehicle (17.66 nm), **1C** (714.7 nm) and **2G** (288.95 nm).

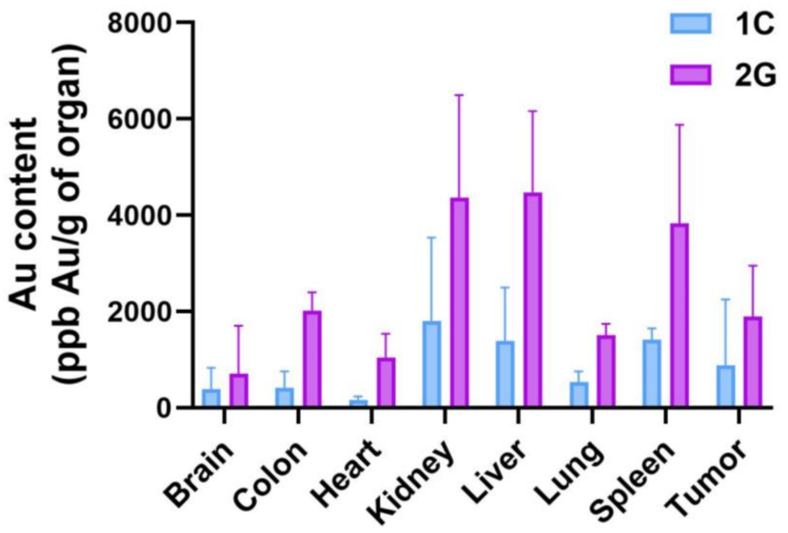


**Supplementary Figure 164**. Au accumulation in mouse organs and tumors obtained from Balb/C mice treated with **1C** (1.5 mg/kg) and **2G** (3.5 mg/kg) via i.p. route at the endpoint (Day 23) and quantified by ICP-MS.

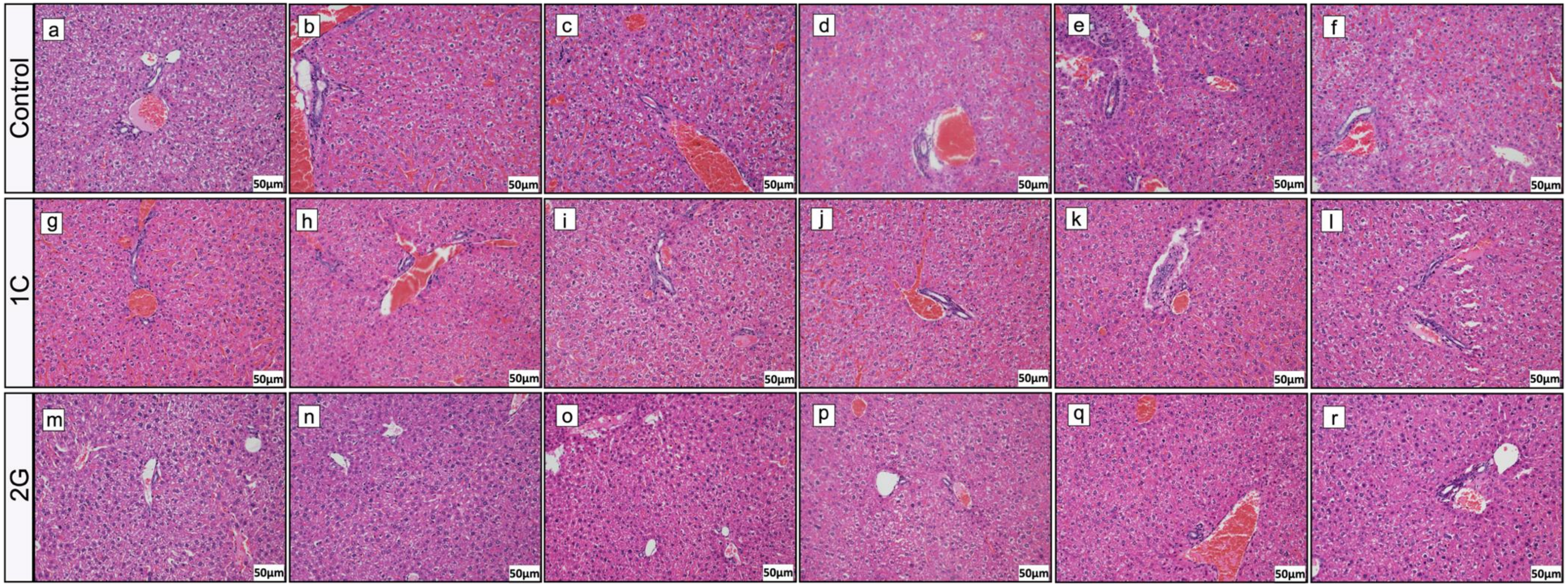


**Supplementary Figure 165.** Representative H&E stained images of liver sections from all mice (n=6) treated with **1C**, **2G** and respective vehicle. Scale bars represent 50 µm.

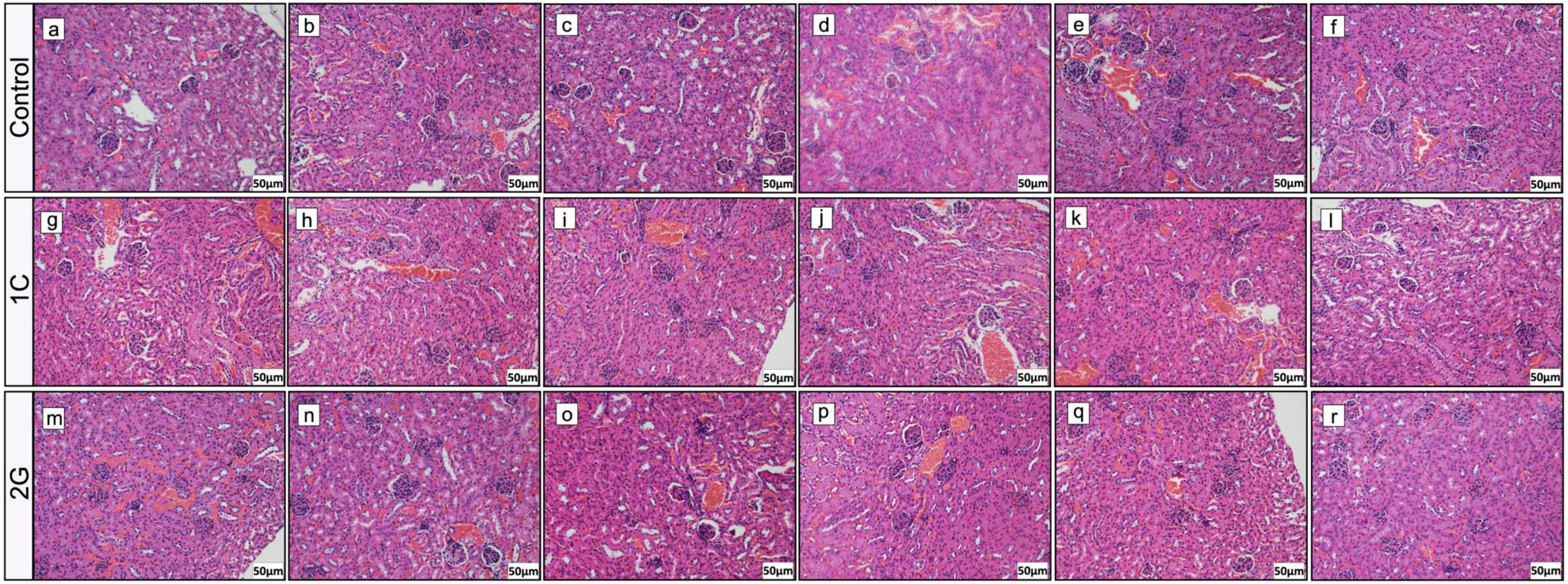


**Supplementary Figure 166.** Representative H&E stained images of kidney sections from all mice (n=6) treated with **1C**, **2G** and respective vehicle. Scale bars represent 50 µm.

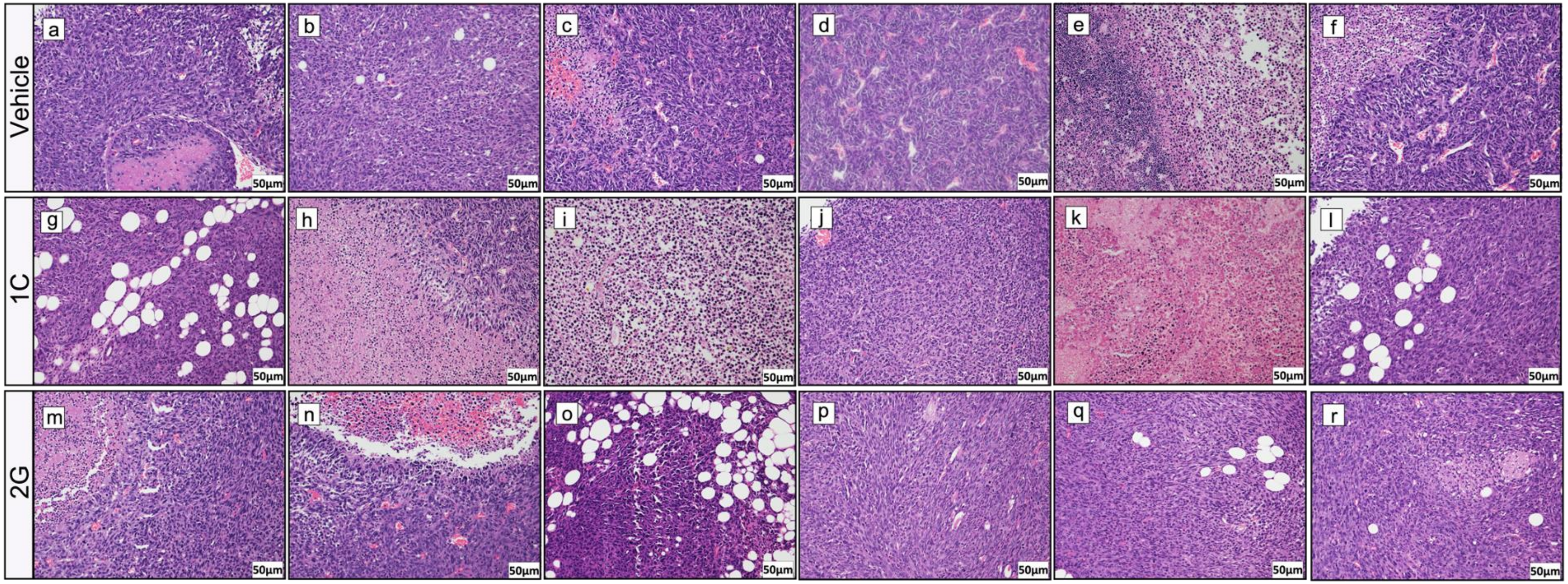


**Supplementary Figure 167.** Representative H&E stained images of tumor sections from all mice (n=6) treated with **1C**, **2G** and respective vehicle. Scale bars represent 50 µm.

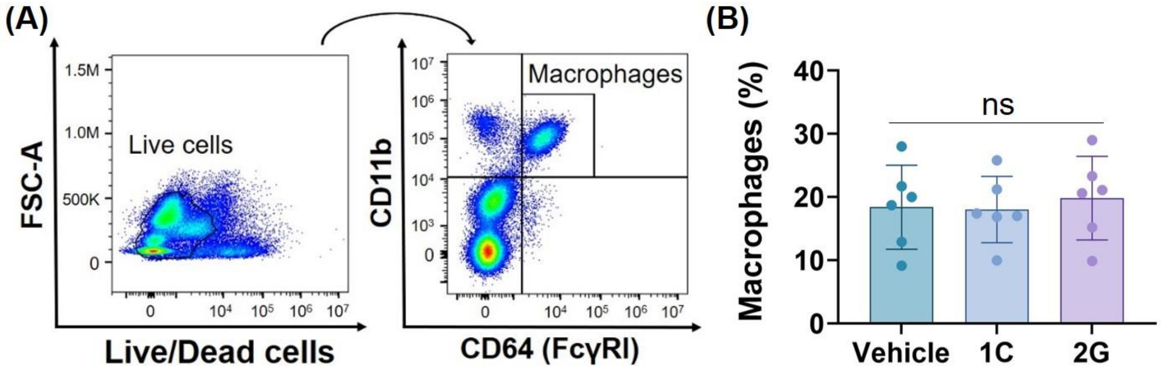


**Supplementary Figure 168.** (A) Gating strategy for Fluorescence-Activated Cell Sorting (FACS). Macrophages were identified as the CD64$^{+}$(FcγRI)/CD11b$^{+}$ double-positive cell subset. (B) Quantification of total percentage of macrophages in tumors freshly isolated from all mice (n=6) treated with **1C**, **2G** and respective vehicle.

## References


1. Ang W.H., Babak M. & Balyasnikova I.V. Gold (III)-biguanide prodrugs, methods of preparation and uses thereof. Patent No. WO2021194420A1 (2021).
2. Smith T.S., Henderson W. & Nicholson B.K. Cycloaurated gold(III) complexes with monoanionic thiourea ligands. *Inorg. Chim. Acta* **408**, 27-32 (2013).
3. Kung K.K.Y. *et al.*, Cyclometallated Gold(III) Complexes as Effective Catalysts for Synthesis of Propargylic Amines, Chiral Allenes and Isoxazoles. *Adv. Synth. Catal.* **355**, 2055-2070 (2013).
4. Bachmann M., Fessler R., Blacque O. & Venkatesan K. Towards blue emitting monocyclometalated gold(iii) complexes – synthesis, characterization and photophysical investigations. *Dalton Trans.* **48**, 7320-7330 (2019).
5. Arojojoye A.S. *et al.*, Circumventing Physicochemical Barriers of Cyclometalated Gold(III) Dithiocarbamate Complexes with Protein-Based Nanoparticle Delivery to Enhance Anticancer Activity. *ACS Appl. Mater. Inter.* **15**, 43607-43620 (2023).
6. Zhang J.J., Ng K.M., Lok C.N., Sun R.W.Y. & Che C.M. Deubiquitinases as Potential Anti-Cancer Targets for Gold(III) Complexes. *Chem. Commun.* **49**, 5153-5155 (2013).
7. Mertens R.T., Parkin S. & Awuah S.G. Cancer cell-selective modulation of mitochondrial respiration and metabolism by potent organogold(iii) dithiocarbamates. *Chem. Sci.* **11**, 10465-10482 (2020).
8. de Andrade Querino A. L. *et al.*, Organogold(III)-dithiocarbamate compounds and their coordination analogues as anti-tumor and anti-leishmanial metallodrugs. *J. Inorg. Biochem.* **247**, 112346 (2023).
9. Bankhead P. *et al.*, QuPath: Open source software for digital pathology image analysis. *Sci. Rep.* **7**, 16878 (2017).
